# Development of a Prognostic Model Based on Pyroptosis-Related Genes in Pancreatic Adenocarcinoma

**DOI:** 10.1155/2022/9141117

**Published:** 2022-05-29

**Authors:** Kaifeng Su, Yang Peng, Haochen Yu

**Affiliations:** ^1^Medical Faculty of Ludwig-Maximilians-University of Munich, University Hospital of LMU Munich, Munich, Germany; ^2^Department of Endocrine and Breast Surgery, The First Affiliated Hospital of Chongqing Medical University, Chongqing, China

## Abstract

**Background:**

The importance of pyroptosis in tumorigenesis and cancer progression is becoming increasingly apparent. However, the efficacy of using pyroptosis-related genes (PRGs) in predicting the prognosis of pancreatic adenocarcinoma (PAAD) patients is unknown.

**Methods:**

This investigation used two databases to obtain expression data for PAAD patients. Differentially expressed PRGs (DEPRGs) were identified between PAAD and control samples. Several bioinformatic approaches were used to analyze the biological functions of DEPRGs and to identify prognostic DERPGs. A miRNA-prognostic DEPRG-transcription factor (TF) regulatory network was created via the miRNet online tool. A risk score model was created after each patient's risk score was calculated. The microenvironments of the low- and high-risk groups were assessed using xCell, the expression of immune checkpoints was determined, and gene set variation analysis (GSVA) was performed. Finally, the efficacy of certain potential drugs was predicted using the pRRophetic algorithm, and the results in the high- and low-risk groups were compared.

**Results:**

A total of 13 DEPRGs were identified between PAAD and control samples. Functional enrichment analysis showed that the DEPRGs had a close relationship with inflammation. In univariate and multivariate Cox regression analyses, GSDMC, IRF1, and PLCG1 were identified as prognostic biomarkers in PAAD. The results of the miRNA-prognostic DEPRG-TF regulatory network showed that GSDMC, IRF1, and PLCG1 were regulated by both specific and common miRNAs and TFs. Based on the risk score and other independent prognostic indicators, a nomogram with a good ability to predict the survival of PAAD patients was developed. By evaluating the tumor microenvironment, we observed that the immune and metabolic microenvironments of the two groups were substantially different. In addition, individuals in the low-risk group were more susceptible to axitinib and camptothecin, whereas lapatinib might be preferred for patients in the high-risk group.

**Conclusion:**

Our study revealed the prognostic value of PRGs in PAAD and created a reliable model for predicting the prognosis of PAAD patients. Our findings will benefit the prognostication and treatment of PAAD patients.

## 1. Introduction

Pancreatic cancer is the fourth leading cause of cancer-related death in the USA and Europe [1], and it is expected to overtake lung carcinoma as the second leading cause by 2030 [2]. The most frequent type of pancreatic cancer is pancreatic ductal adenocarcinoma (PDAC), which accounts for up to 90% of pancreatic adenocarcinoma cases [3]. Despite the low incidence and relative rarity, with documented incidences in the United States and Europe ranging from 11.5 to 15.3 per 100,000 people [4], the five-year survival rate is dismal, typically falling below 10% [5]. This bleak outlook can be attributed to a number of factors. The insidious and nonspecific symptoms of the disease make it difficult to diagnose, and cases of pancreatic cancer that can be definitively diagnosed are usually already in advanced stages. In addition, a growing number of clinical cases have found that pancreatic cancer exhibits significant resistance to traditional treatment options, including chemotherapy, radiation, and immunotherapy [6, 7]. This resistance makes surgery the best option for pancreatic adenocarcinoma (PAAD) treatment, and for 10-20% of patients who undergo surgery, the 5-year survival rate is still only 15-25% [8]. Adjuvant therapy is also further evolving. Adjuvant treatment with a modified FOLFIRINOX regimen has recently demonstrated an extended median overall survival of 4.53 years and a 3-year survival rate of 63.4% in patients after surgical operation [9]. With regard to chemotherapy, for approximately 80% of patients with locally advanced or metastatic illness, gemcitabine or 5-fluorouracil chemotherapy provides a survival time of months, and nab-paclitaxel combined with gemcitabine or FOLFIRINOX provides slight improvements [10, 11]. Neoadjuvant chemotherapy has evolved rapidly in recent decades, giving patients with advanced disease more possibilities for surgical resection and radiation therapy [12]. However, there are still limitations in the treatment and diagnosis of PAAD, and further research to explore new mechanisms and discover new potential targets is still warranted. The importance of programmed cell death (PCD) is being increasingly recognized in cell biology, and cell apoptosis and necrosis are two of the most classical forms of PCD. In the last decade, ferroptosis, autophagy, and pyroptosis of cells have become particularly interesting as emerging modalities of PCD. Miao et al. described the mechanism of pyroptosis as a unique type of caspase-1-dependent PCD in 2001 [13]. Pyroptosis is distinguished by gasdermin family-mediated pore development and then cell lysis, which also releases some intracellular proinflammatory cytokines. Thus, pyroptosis is considered a novel proinflammatory cell death program [14]. Based on the prevailing academic view, pyroptosis can be divided into two pathways. In the first pathway, pyroptosis is accompanied by the activation of caspase-1, which is referred to as the canonical pathway. The noncanonical pathway, in contrast, mainly involves the activation of atypical caspases 4/5 and 11 (human caspases 4 and 5 and mouse caspase 11) [15]. Pyroptosis research in solid tumors is also emerging. There is growing evidence that pyroptosis can be a potential target for tumor therapy [16]. However, there are few basic experimental studies on pyroptosis in pancreatic cancer. In 2019, Cui et al. found that MST1 inhibits pancreatic cancer growth through the ROS-related pathway in the pyroptosis process [17]. Unfortunately, few pyroptosis-related medicines (inhibitors or activators) have been confirmed for clinical use; additionally, no detailed studies or data have been published to elucidate the relationship between pyroptosis and pancreatic cancer outcomes, and for pancreatic cancer, there are few prediction models available. Due to the lack of basic experimental studies and the inaccessibility of pyroptosis clinical data, the use of big data and bioinformatics to try to investigate the connections between pyroptosis and pancreatic cancer, as well as the connections between relatively well-studied subject areas (e.g., the immune microenvironment and tumor metabolism) and pyroptosis in PAAD is intriguing.

With the rapid advancement of gene sequencing technology, there is undeniably an increase in the number of reports on the gene signature of pancreatic cancer. For example, a 9-gene signature [18], a 15-gene signature [19], a tumor stem cell-associated signature [20], and a miRNA-associated signature [21] have been reported.

However, studies on pyroptosis-related signatures have not been reported in pancreatic cancer. Our research did not aim to find direct evidence for pyroptosis-related causes and treatment of pancreatic cancer. Because the regulation of tumor growth is not determined by a few genes alone, we focused on the impact of pyroptosis genes on the overall regulation of pancreatic cancer and aimed to identify new targets and treatment options. Finally, we provide a reference for the precise management of individuals with pancreatic cancer.

## 2. Materials and Procedures

### 2.1. Information Source

The PAAD patients' transcriptome profile (RNA-seq FPKM) and clinical characteristics were obtained from The Cancer Genome Atlas (TCGA) database (https://portal.gdc.cancer.gov/) and the International Cancer Genome Consortium (ICGC) database (https://dcc.icgc.org/). The distribution of the clinical features can be found in Table 1. TCGA-PAAD cohort was used as the training set to construct the risk score model. To test this model, data from the ICGC database were utilized as an external dataset. Fifty-seven pyroptosis-related genes (PRGs) were collected from the Reactome database (https://reactome.org/content/detail/R-HSA-5620971), MSigDB database (http://www.gsea-msigdb.org/gsea/msigdb/cards/GOBP_PYROPTOSIS.html), and previous literature [22–25].

### 2.2. Identification and Functional Analysis of DEPRGs in PAAD

DEGs between 179 tumors and 4 control samples were identified through the limma R package [26] using the criterion of |log2*FC*| > 1 and adjusted *p* value <0.05. The differentially expressed PRGs (DEPRGs) were obtained by overlapping the DEGs with the 57 PRGs. The clusterProfiler R package [27] was used to screen significantly enriched biological process (BP), cellular component (CC), molecular function (MF), and Kyoto Encyclopedia of Genes and Genomes (KEGG) pathway terms of the DEPRGs with *p* value <0.05 as a threshold.

### 2.3. Identification of Robust Prognostic DEPRGs in PAAD

Based on the expression profiles and clinical information of the 179 patients in the TCGA-PAAD cohort, DEPRGs significantly associated with survival (*p* value <0.05) were identified using univariate Cox regression. Thereafter, the genes obtained from the univariate Cox regression were input into a multivariate Cox regression model to acquire robust prognostic DEPRGs (*p* value <0.05). To further explore the transcriptional and posttranscriptional regulation of the prognostic DEPRGs, the miRNet online tool (https://www.mirnet.ca/) was applied to predict the miRNAs and TFs targeting prognostic DEPRGs. Thereafter, a miRNA-prognostic DEPRG-TF regulatory network was constructed and visualized using Cytoscape software [28].

### 2.4. Development and Validation of a Risk Score Model for Predicting PAAD Prognosis

Depending on the expression of prognostic DEPRGs and the coefficients, the risk score of every patient in the TCGA-PAAD cohort was calculated using the following formula:

(1) $$\begin{array}{c} \text{ExpGene}1*\text{Coef}1+\text{ExpGene}2*\text{Coef}2+\text{ExpGene}3*\text{Coef}3\cdots \end{array}$$

Every signature gene's normalized expression value is Exp, and the gene's regression coefficient in multivariate Cox regression analysis is Coef. Thereafter, based on the median risk score, PAAD patients in the TCGA training set were divided into low- and high-risk groups. Kaplan–Meier analysis was used to examine the patients' overall survival in the low- and high-risk categories. ROC curves were used to evaluate the performance of the risk score model through the “survivalROC” R package [29]. 169 patients in the aggregate with whole clinical characteristics in the ICGC-PACA-CA cohort were performed to validate this risk model.

### 2.5. Development of a Nomogram to Predict Prognosis in PAAD

To determine independent prognostic factors for PAAD patients, clinical variables (age, sex, grade, and TNM stage) and the risk score were input into a multivariate Cox regression model. Then, independent prognostic markers determined by multivariate Cox regression analysis (*p* value <0.05) were incorporated to establish a nomogram for predicting the 1-, 3-, and 5-year survival of PAAD patients. Calibration curves were plotted to assess the nomogram's performance.

### 2.6. Characterization of PAAD Patients in the High- and Low-Risk Score Groups

The PAAD patients in the high- and low-risk score groups were characterized in three ways. (1) The reference gene sets “c2.cp.kegg.v6.2.symbols” were retrieved from the MSigDB database and utilized for GSVA enrichment analysis and in heatmap by the “GSVA” R package [30]. Significantly enriched KEGG pathways were found between the high- and low-risk score groups with a *p* value <0.05. (2) The xCell algorithm [31] was used to evaluate the microenvironment of patients in the high- and low-risk score groups. (3) The expression of 35 immune checkpoints was compared between patients in the high- and low-risk score groups. Furthermore, to investigate the sensitivity of patients in high- and low-risk categories to common anticancer drugs, IC_50_ values were analyzed using the pRRophetic algorithm [32].

## 3. Results

### 3.1. Thirteen DEPRGs Associated with Immunity Were Identified between PAAD and Control Samples

**Figure 1:**
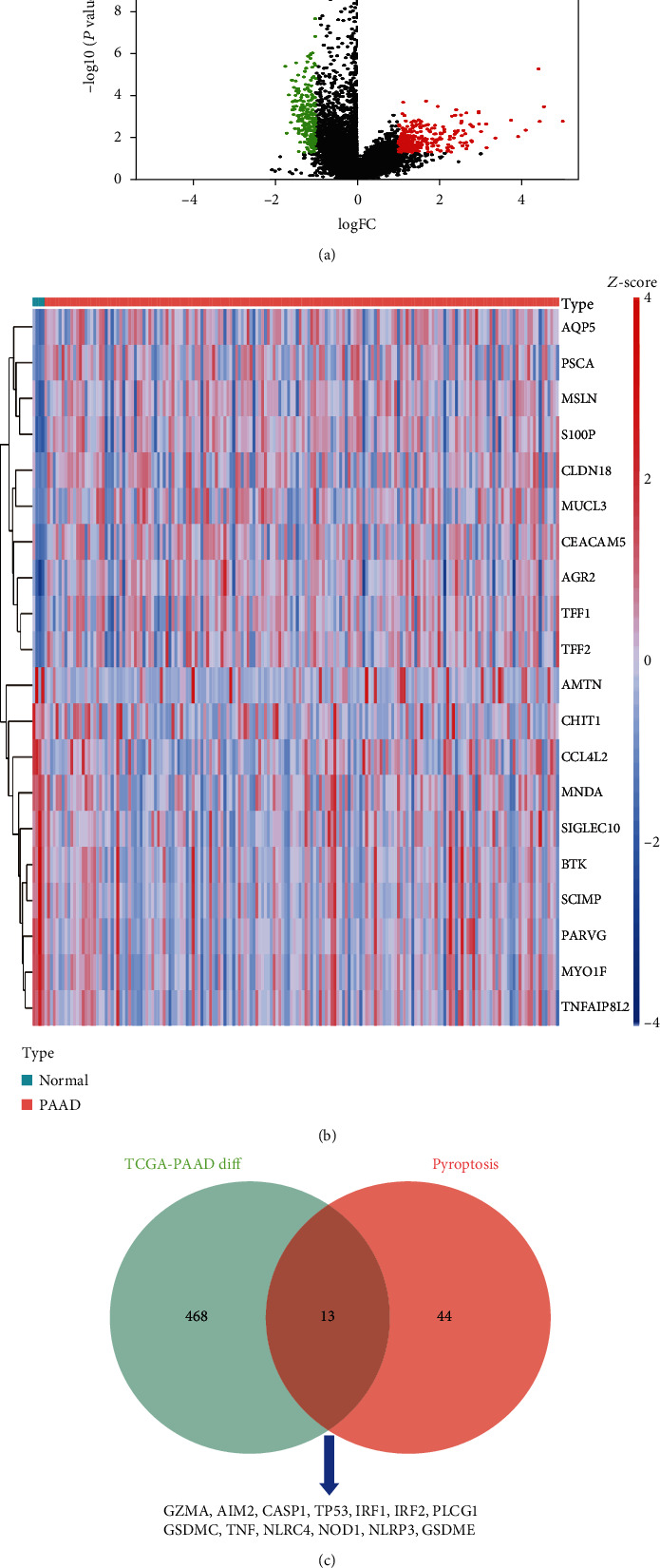
Identification of the expression of 13 different PRGs. (a) Volcano plot presenting differentially expressed genes between normal and tumor tissues from the TCGA dataset. Red plot: upregulated genes in tumor samples. Green plot: downregulated genes in tumor samples. (b) Heatmap (green: low expression level, red: high expression level) of differentially expressed genes between the normal (blue) and PAAD (red) samples. (c) Venn diagram: the green circle on the left includes the 481 TCGA-PAAD cohort differentially expressed genes, the red circle on the right includes the 57 pyroptosis-related genes, and the intersection of the two circles includes the 13 differentially expressed pyroptosis-related genes.

**Figure 2:**
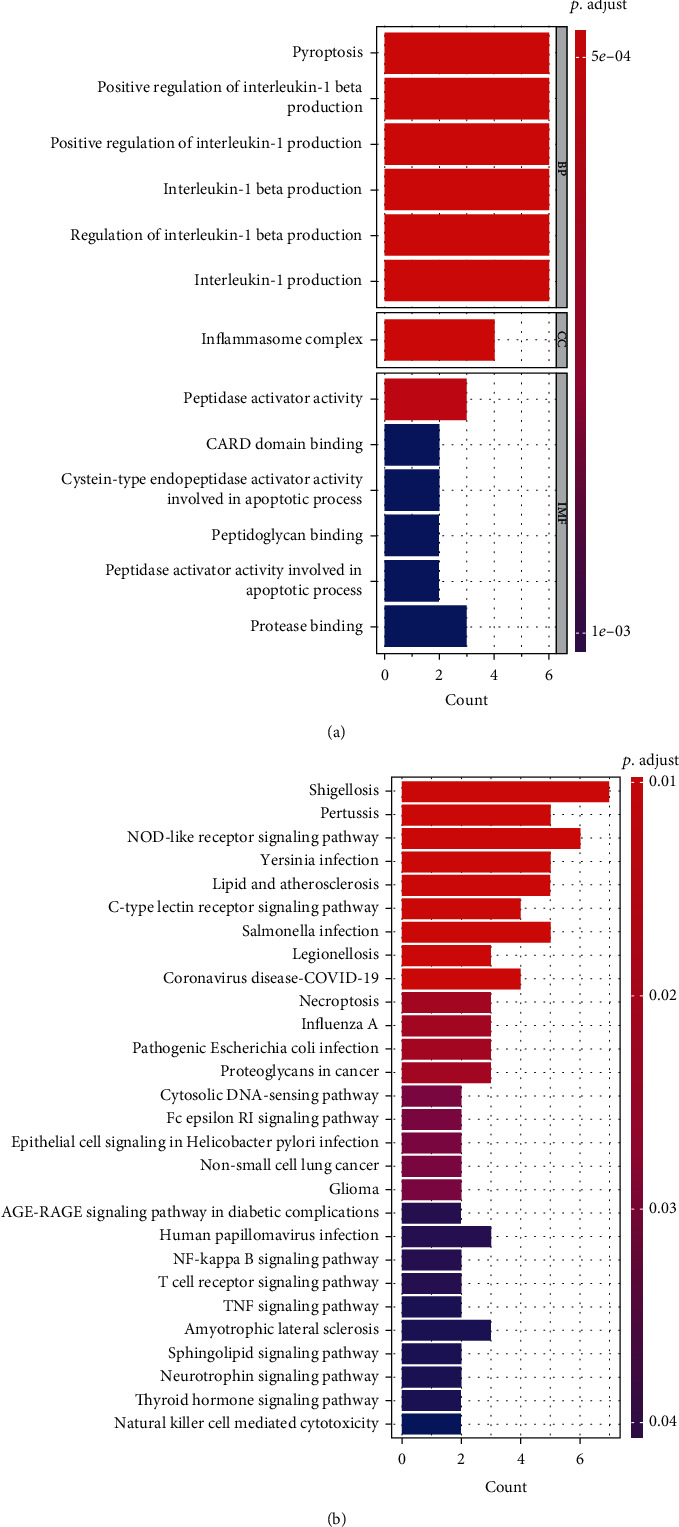
Functional enrichment analysis of DEPRGs in the TCGA cohort. (a) Bar graph for GO analysis category. (b) Bar graph for the KEGG analysis. A longer bar means that more genes were enriched, and an increasing depth of red means that the differences were more obvious. BP: Biological process; CC: Cellular component: MF: Molecular function. DEPRGs differentially expressed pyroptosis-related genes.

A total of 481 DEGs were detected between PAAD and control samples (Figure 1(a) and Table S1), including 257 upregulated and 224 downregulated genes in tumor samples relative to control samples. The expression of the top 20 DEGs is shown in a heatmap (Figure 1(b)). After overlapping the DEGs with PRGs, GZMA, AIM2, CASP1, TP53, IRF1, IRF2, PLCG1, GSDMC, TNF, NLRC4, NOD1, NLRP3, and GSDME were identified as DEPRGs (Figure 1(c)). Functional analysis results revealed that the DEPRGs were remarkably enriched in 148 BP, one CC, 28 MF, and 28 KEGG pathway terms (Table S2). We found that the top Gene Ontology (GO) and KEGG pathway terms were related to inflammation (Figures 2(a) and 2(b)), such as positive regulation of interleukin-1 beta production, inflammasome complex, NOD-like receptor signaling pathway, and C-type lectin receptor signaling pathway, indicating that the DEPRGs may participate in PAAD via immune regulation.

### 3.2. Three DEPRGs Were Identified as Prognostic Biomarkers in PAAD

**Figure 3:**
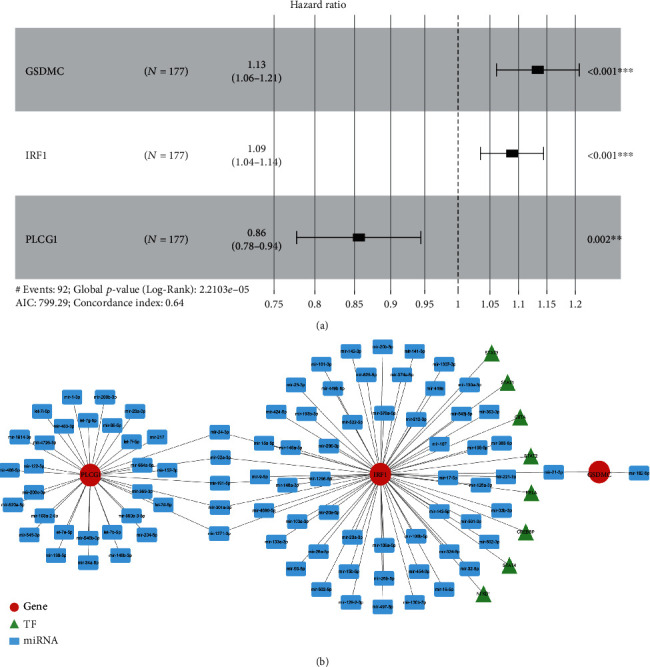
Multivariate Cox regression analysis of the three DEPRGs and construction of a miRNA–mRNA–TF regulatory network. (a) Forest plot presenting the HRs for the three DEPRG prognostic models. (b) The miRNA–mRNA–TF regulatory network including the three DEPRGs, 89 miRNAs, and eight TFs.

Thereafter, we assessed the prognostic value of the 13 DEPRGs in PAAD through univariate and multivariate Cox regression analyses. GSDMC, IRF1, and PLCG1 were found to be significantly correlated with the survival of PAAD patients (Table 2) by univariate Cox regression analysis. These three DEPRGs were input into a multivariate Cox regression model to gain more robust prognostic biomarkers. GSDMC, IRF1, and PLCG1 remained remarkably linked to the prognosis of PAAD patients (Figure 3(a)) and were identified as prognostic biomarkers. Moreover, using the miRNet online tool, we constructed a miRNA–mRNA–TF regulatory network composed of three prognostic biomarkers, 89 miRNAs, and eight TFs. In the network, we found that GSDMC, IRF1, and PLCG1 were regulated by both specific and common miRNAs, whereas only IRF1 was predicted to be regulated by TFs, including STAT1/2/3/4, RELA, NFKB1, CREBBP, and CIITA (Figure 3(b)).

### 3.3. A PRG-Related Risk Score Model Was Constructed and Validated in PAAD

**Figure 4:**
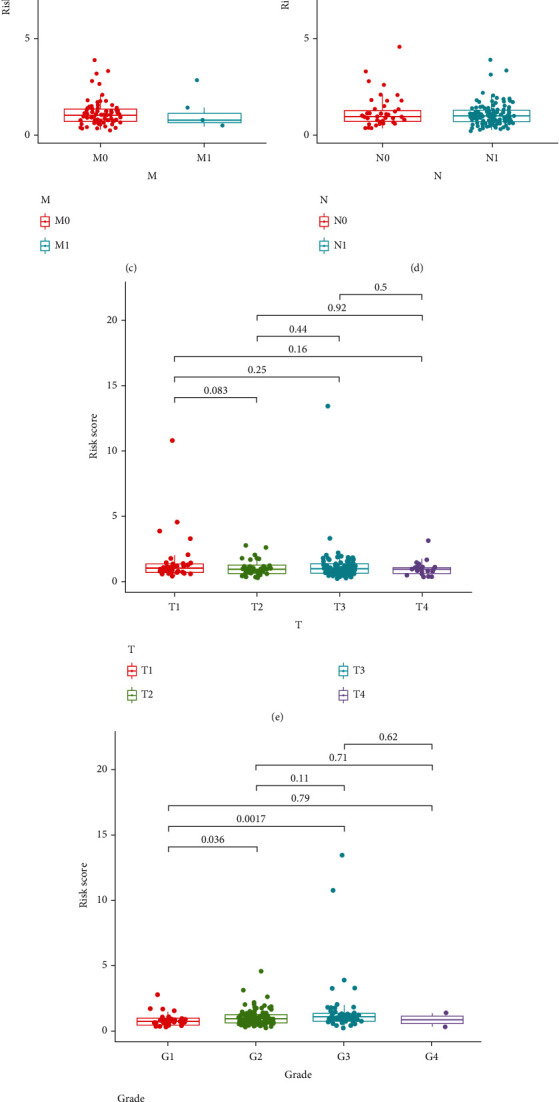
Clinical evaluation based on the risk score. Scatter diagram showing (a) age, (b) sex, (c) M stage, (d) N stage, (e) T stage, (f) tumor grade, and (g) clinical stage based on the risk score. The statistical test used by nonparametric tests.

**Figure 5:**
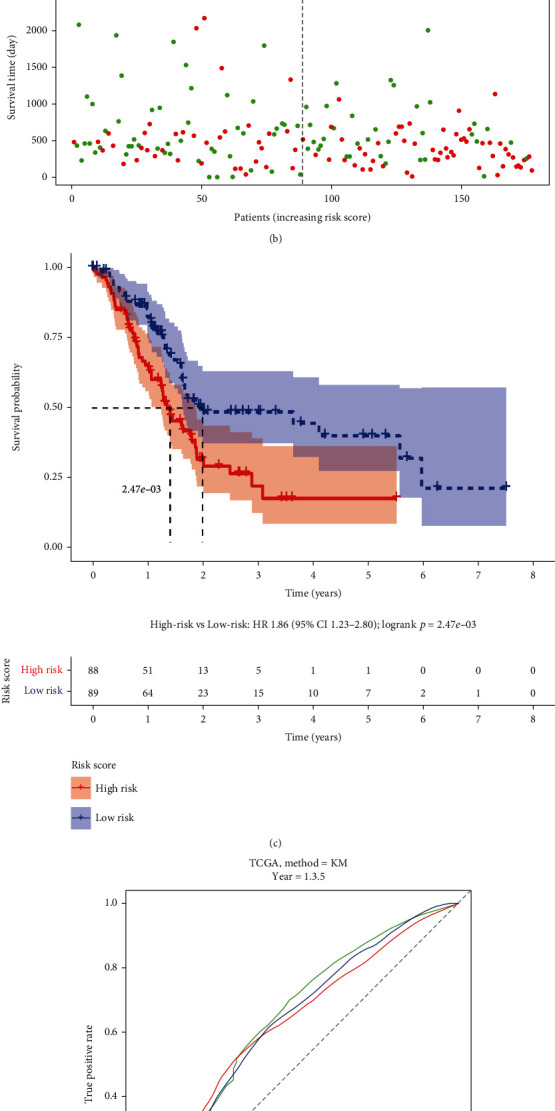
Prognostic analysis of the risk signature model in the TCGA cohort. (a) Distribution of patients based on the risk score. (b) Survival status for each patient (low-risk population: on the left side of the dotted line; high-risk population: on the right side of the dotted line; green plot: alive; red plot: dead). (c) Kaplan–Meier curves for the OS of patients in the high- and low-risk groups. (d) ROC curves showing the predictive efficiency of the risk score.

**Figure 6:**
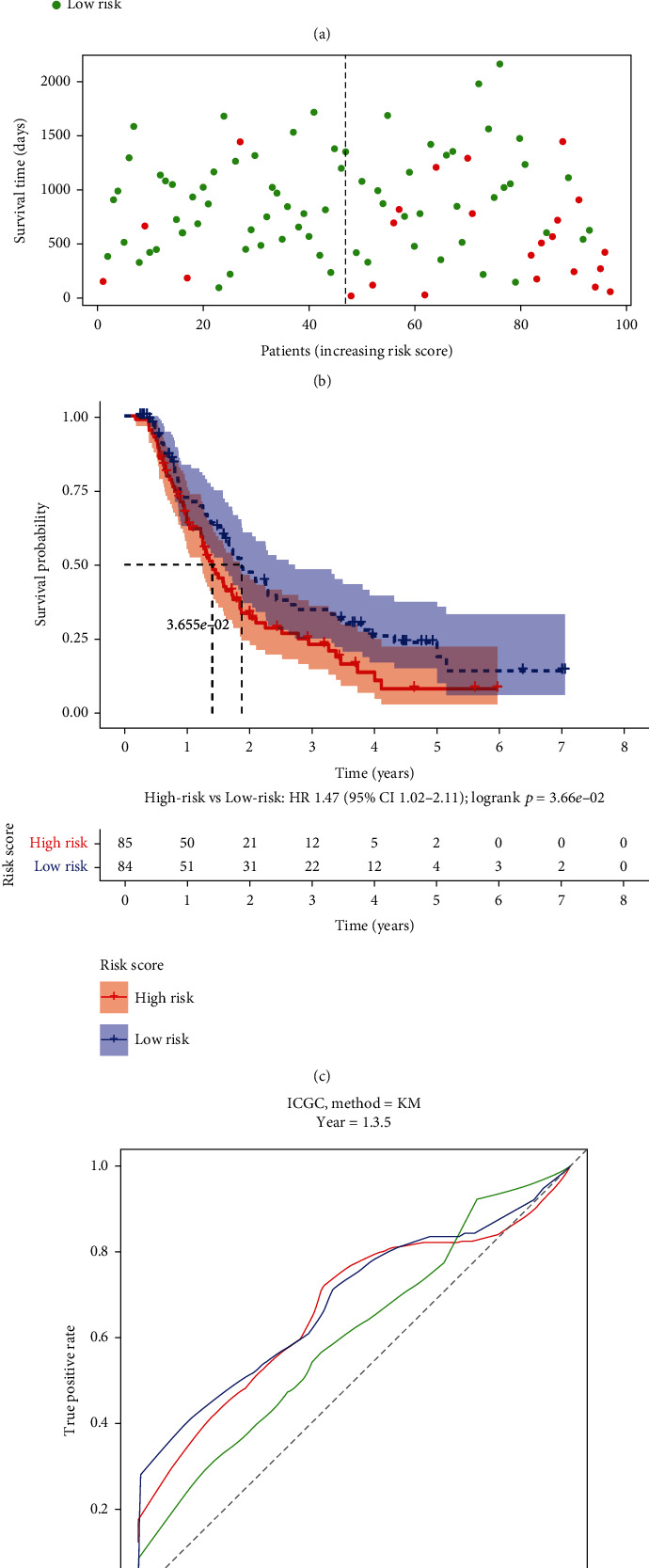
Validation of the risk model in the ICGC cohort. (a) Distribution of the ICGC cohort based on the risk score. (b) Survival status for each patient. (c) Kaplan–Meier curves for the OS of patients in the high- and low-risk groups. (d) ROC curves for PAAD.

Depending on the expression levels and coefficients of GSDMC, IRF1, and PLCG1, we calculated every patient's risk score as follows: risk score = (0.124∗GSDMC exp.) + (0.084∗IRF1 exp.) + (−0.155∗PLCG1 exp.). We found that the risk score differed considerably between groups stratified by tumor grade, but no significant difference in risk score was observed between groups classified by age, sex, TNM stage, or tumor stage (Figure 4). The PAAD patients in the TCGA training set were divided into high- and low-risk groups according to the median risk score (Figures 5(a) and 5(b)). The high-risk group showed worse survival than the low-risk group (*p* value <0.05, hazard ratio = 1.86, confidence interval = 1.23 − 2.80, Figure 5(c)). The ROC curves and area under the ROC curve (AUC) values revealed that this risk score model performed well in predicting the 1-, 3- and 5-year survival of PAAD patients, with AUC values of 0.63, 0.619, and 0.639, respectively (Figure 5(d)). Furthermore, the risk score model was also evaluated in the ICGC testing set and detected similar results (Figures 6(a)–6(d)), indicating that it was reliable for predicting PAAD patient survival.

### 3.4. A PRG-Related Nomogram Was Developed in PAAD

**Figure 7:**
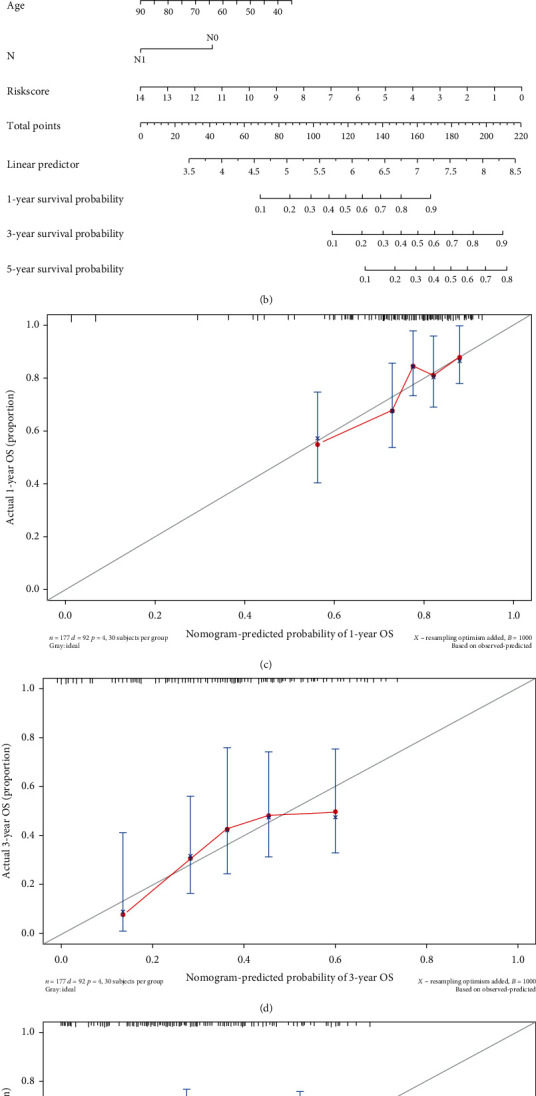
Combination of the risk model and clinical characteristics for predicting PAAD prognosis. (a) Multivariate Cox regression analysis of independent prognostic factors for the risk model. (b) Nomogram constructed to predict the probability of OS in PAAD patients at 1, 3, and 5 years. (c)–(e) Calibration curves for 1-, 3-, and 5-year survival probabilities in the TCGA cohort.

Next, we detected independent prognostic factors in PAAD by performing multivariate analyses and found that the risk score, age, and N stage were remarkably correlated with prognosis (Figure 7(a)), indicating that they were independent prognostic factors for PAAD. Then, a nomogram for predicting the 1-, 3- and 5-year survival of PAAD patients was established (Figure 7(b)) using those independent prognostic factors. Calibration curves revealed that the predicted probability of overall survival was close to the actual overall survival, indicating good performance of the nomogram (Figures 7(c)–7(e)).

### 3.5. The Microenvironments of the High- and Low-Risk Groups Were Different

**Figure 8:**
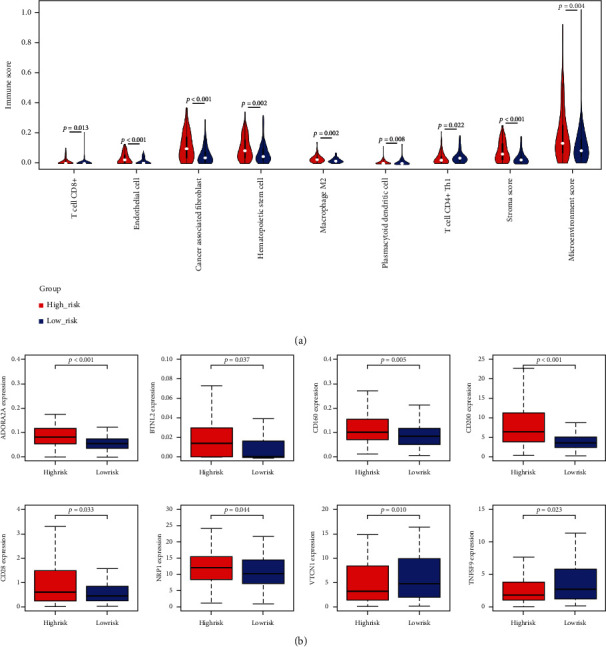
Association of the risk score with the microenvironment and immune checkpoints. (a) Comparison of immune cells and the stroma between the low- and high-risk groups in the TCGA cohort. (b) Comparison of immune checkpoints between the low- and high-risk groups in the TCGA cohort. The statistical test used by nonparametric tests.

**Figure 9:**
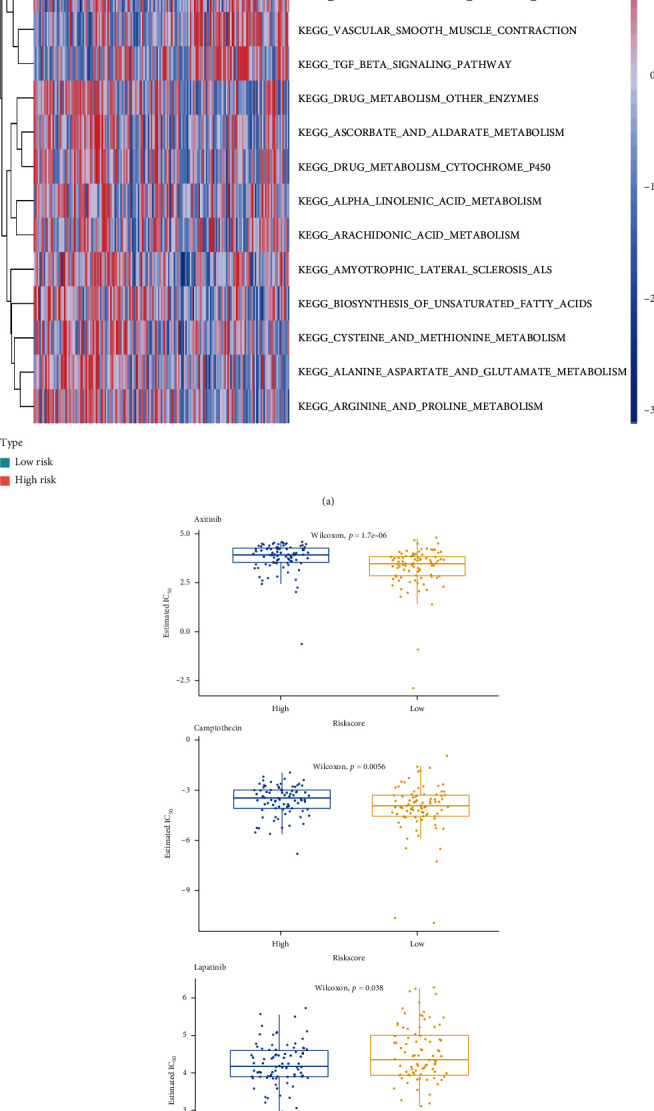
GSVA and determination of response to chemotherapy. (a) GSVA showing the activation states of biological pathways between the high- and low-risk groups. A heatmap was used to visualize the enriched biological processes. Red represents activated pathways, and blue represents inhibited pathways. (b) Correlation analysis between chemotherapeutic drug sensitivity and the model: the low-risk group had lower IC_50_ values for axitinib and camptothecin and a higher IC_50_ value for lapatinib.

The microenvironment of PAAD patients in the high- and low-risk groups was then investigated. With xCell, we discovered that there was a significant difference in the infiltration of CD8+ T cells, endothelial cells, cancer-associated fibroblasts, hematopoietic stem cells, M2 macrophages, plasmacytoid dendritic cells, and CD4+ Th1 T cells between the high- and low-risk groups, and the high-risk groups had higher stromal and microenvironment scores (Figure 8(a)). Furthermore, among the 35 immune checkpoint molecules, patients in the high-risk group had significantly higher expressions of ADORA2A, BTNL1, CD160, CD200, CD28, and NRP1 expression levels and lower expression of VTCN1 and TNFSF9, and no significant expression difference of other 27 immune checkpoints was found between low- and high-risk group (Figure 8(b), Figure S1). In addition, GSVA identified 184 KEGG pathway terms that were significantly differentially enriched between the high- and low-risk groups (Table S3). Interestingly, we observed that the top 20 differentially enriched KEGG pathway terms between the high- and low-risk groups were related to metabolism, such as inositol phosphate metabolism, ascorbate and aldarate metabolism, alphalinolenic acid metabolism, and cysteine and methionine metabolism (Figure 9(a)). These findings indicate that pyroptosis might disturb the immune and metabolic microenvironment, thus affecting the prognosis of PAAD patients. Increasing evidence has reported that the tumor microenvironment influences the treatment response of cancer patients [33, 34]. Therefore, we compared the IC_50_ values of common anticancer drugs between the high- and low-risk groups. We found that patients in the low-risk group were more sensitive to axitinib and camptothecin but more resistant to lapatinib (Figure 9(b)).

## 4. Discussion

**Figure 10:**
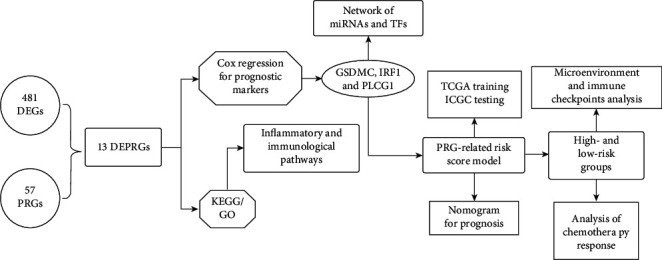
Workflow diagram. Specific workflow diagram for data analysis.

Various forms of tumor death have been discovered. From apoptosis (proposed in 1972) [35] to ferroptosis (which has been increasingly researched in the last decade) [36] to alkaliptosis (proposed in 2018) [37], researchers are exploring the mechanisms of cell death. In 2001, pyroptosis was also a focus of research because of its characteristic inflammatory response. Basic experimental studies related to pyroptosis and tumors are also abundant, and pyroptosis has been found in tumor cells in digestive cancers [38–40], breast cancer [41], and lung cancer [42]. Unfortunately, studies of pyroptosis in PAAD are very rare. Since 2019, only Cui Jet al. have clearly suggested that MST1 is able to induce pyroptosis via ROS [17]. Few studies have reported whether studies of pyroptosis can provide therapeutic insight for clinicians. In addition, there are few studies in the field of PAAD. As a result, we aimed to investigate the association between pyroptosis and PAAD using clinical data to develop more innovative methods for clinical diagnosis and therapy. The entire experimental design flow of the study is shown in Figure 10.

We first investigated the intersection of the DEGs in PAAD and the PRGs. After overlapping the DEGs with the PRGs, GZMA, AIM2, CASP1, TP53, IRF1, IRF2, PLCG1, GSDMC, TNF, NLRC4, NOD1, NLRP3, and GSDME were identified as DEPRGs. The KEGG and GO studies revealed that these 13 genes are linked to inflammatory and immunological pathways, especially IL-1-related pathways. In 2020, Das et al. reported a novel mode of immune evasion in PAAD that is dependent on tumor cell IL-1 production via TLR4-NLRP3 inflammasome activation [43]. Similarly, regarding IL-1 and NLRP3, Zhang et al. discovered that fatty acid oxidation was responsible for increased IL-1 secretion and the resultant promigratory impact on M2 phenotype monocyte-derived macrophages. Furthermore, the researchers demonstrated that IL-1 induction was mediated by reactive oxygen species and was NLRP3 dependent. Their studies showed that fatty acid oxidation plays an important role in functioning human M2 macrophages by increasing IL-1 production, which promotes hepatocellular carcinoma cell motility [44]. Interleukins and TNF-*α* are also thought to play an important role in gastroenteropancreatic neuroendocrine neoplasms. In a 2020 article, it was reported that TNF-*α* can be used as a prognostic indicator, while various ILs (IL-2, IL-1*β*, and IL-6) were also discovered to be linked to tumor prognosis [45]. Excitingly, we found a recent report that more completely elaborates the role of NLRP3, CASP1, and IL-*β* in pancreatic cancer. According to a previous study, LPS-induced inflammation in the presence of ATP activates NLRP3, which enhances pancreatic cancer cell proliferation by boosting caspase-1 activity, resulting in total IL-1*β* production [46]. Although the term “pyroptosis” is not explicitly stated in the article, we believe that the mechanism detailed in the study is most likely the canonical CASP1-dependent pyroptosis pathway. In fact, interleukin-based inflammatory agents have been shown to act as prognostic indicators in PAAD [47]. Combined with what has been described previously, we suggest that these genes associated with pyroptosis regulate the development of PAAD through multiple pathways that affect immunity or inflammation.

To identify more precise prognostic markers for PAAD, we employed Cox regression (univariate and multivariate) to examine the 13 genes, and ultimately, we obtained three genes of interest: GSDMC, IRF1, and PLCG1. We analyzed the regulatory network of miRNAs and TFs linked with these three genes. This network contained several experimentally confirmed regulatory pathways. One study showed that miR-21 promotes the activation of inflammasomes to induce pyrolysis [48]. Wang et al. discovered in 2019 that metformin causes pyroptosis in human esophageal cancer cells by targeting the miR-497/PELP1 axis [49]. Similarly, miR-133a [50], miR-148a [51], and the TF STAT [52–54] were found to be involved in pyroptosis progression in our network. This research suggests that a significant number of miRNAs have a role in pyroptosis control, although more basic studies are needed to corroborate this concept.

The GSDM family includes six genes in humans, namely, GSDMA, GSDMB, GSDMC, GSDMD, GSDME, and DFNB59. These genes may be linked to cell proliferation and differentiation, as well as cancer in several organs [55]. Two mechanisms of GSDM activation have been confirmed: the first pathway is protease-related cleavage of the linkage region to release the N-terminal active domain, and the second pathway involves mutations in the C-terminal structural domain that disrupt the interaction between the N-terminal and C-terminal structural domains and reduce the ability of the C-terminal domain to inhibit N-terminal domain pore formation. Specifically, different GSDMs have different activation methods. GSDMD was found to be activated by caspases-1/4/5/11, GSDME was found to be activated by caspase-3, and GSDMB was found to be activated by caspases-3/6/7. GSDMA was the first identified GSDM family member, and the expression of GSDMA3 upregulates caspase-3 expression, implying that GSDMA3 may be associated with apoptosis. GSDMB promotes caspase-4 activity by binding to the CARD structural domain of caspase-4, which may be another pathway for cell pyroptosis. However, the related functions of GSDMC are poorly studied [56]. When GSDMC was discovered in metastatic melanoma cells, it was named melanoma-derived leucine zipper extranuclear factor (MLZE) due to a suspected leucine zipper in its C-terminal domain [57]. Overexpression of the N-terminal domain of GSDMC in human 292 T cells can yield pyroptotic characteristics comparable to those induced by other GSDM family members [58]. Furthermore, Hou et al. [59] found that caspase-8 can specifically cleave GSDMC and generate an NT domain that forms pores on the cell membrane and induces pyroptosis of MAD-MB-231 cells. Furthermore, GSDMC has been discovered to have a crucial function in colorectal cancer [60], lung cancer [61], and other solid tumors. In our study, GSDMC seemed to be a oncogene, since it was expressed nearly twice as strongly in tumor tissues as in normal tissues, contributing to a decrease in patient survival.

Phospholipase C grammar 1 (PLCG1) is ubiquitously expressed in various tissues and is reported to be associated with the receptor tyrosine kinase- (RTK-) related signaling pathway, influencing multiple biological functions of cells [62, 63]. Some studies have revealed that PLCG1 can drive the progression of cancers, such as breast carcinoma [64], colorectal carcinoma [65], and small-cell lung cancer [66]. PLCG1 has not been reported in pancreatic cancer, and we only found that phosphorylation and translocation of PLCG1 were associated with PAAD invasion and metastasis [67]. It is worth noting that we analyzed PLCG1 expression and its correlation with prognosis. PLCG1 was highly expressed in tumor tissue, but patients with high expression had better survival. Based on our Cox analysis findings, PLCG1 also plays a protective role in PAAD. This relationship between expression and prognosis seems to be contradictory. However, our careful analysis revealed an interesting phenomenon in which PLCG1 expression in pancreatic cancer was mainly found in mesenchymal cells rather than tumor cells. In contrast, PLCG1 expression was not high in normal pancreatic mesenchymal cells (the expression location data can be found at https://www.proteinatlas.org/ENSG00000124181-PLCG1). Tumor mesenchymal cells mainly regulate the tumor microenvironment, and alterations in mesenchymal cells change tumor immunity. Therefore, we hypothesize that the main reason for the contradictory results is the feedback regulation of pancreatic cancer. It is possible that the increased expression of PLCG1 is due to the presence of tumors and that this increase in PLCG1 is intended to negatively regulate tumor growth. Although we did not find other relevant studies in solid tumors, we confirmed in basic studies of other diseases that PLCG1 may be a protective factor. The article “Reduced PLCG1 expression is associated with inferior survival for myelodysplastic syndromes” published in 2020 by Masayuki Shiseki et al. provides good supporting evidence. The association of PLCG1 with pyroptosis has also been demonstrated, with the main mechanism being involvement in the GSDMD-mediated pyroptosis pathway [68]. In 2018, Kang et al. found that GSDMD-N-induced cell death can be inhibited by the knockdown of PLCG1 and indicated that PLCG1 could be involved in GSDMD-dependent pyroptosis [69]. This paper supports our previous speculation that PLCG1 is a protective factor, suggesting that this protective mechanism is likely to promote cellular pyroptosis.

The IRF-1 gene encodes a protein that acts as a transcriptional regulator and tumor suppressor, activating genes involved in both innate and acquired immune responses. The encoded protein promotes the transcription of genes involved in the body's response to viruses and bacteria, as well as cell proliferation, apoptosis, the immunological response, and the DNA damage response [70]. Some studies have revealed that IRF-1 has antitumor effects via apoptosis and the cell cycle in human cancer [71]. In addition to apoptosis, IRF-1 has been linked to the control of cellular autophagy in breast cancer [72]. Although reports on IRF-1 regulation of pyroptosis are uncommon in tumors, we found that IRF-1 is involved in the pyroptosis pathway in other diseases. IRF-1 can induce macrophages in acute coronary syndrome [73, 74]. The same pyroptosis of macrophages is also induced by IRF-1 in acute lung injury [75]. These results suggest that IRF-1 plays an important role in tumor immunity and pyroptosis. In a mouse model, Shao et al. discovered that the lack of IRF-1 can result in the loss of PD-L1 and tumor cell killing via CD8+ T cells [76]. Similarly, Yan et al. also found that IRF-1 can upregulate PD-L1 and result in tumor cell evasion of antitumor immunity via T cell interactions in human hepatocellular carcinoma [77]. However, the relationship between tumor immunity and pyroptosis remains unknown. In our study, using the expression of the three genes to analyze and predict the prognosis of patients was found to be a feasible approach with good statistical significance.

We are also interested in the predictive power of these three genes in other cancers. Therefore, we performed a prognostic pancancer analysis and found that these three genes alone did not show good predictive power as prognostic factors for other tumor prognoses (Figure S2).

Meanwhile, we analyzed the relationship between clinical features and risk scores. Based on the clinical data available in the TCGA database, we did find that risk scores were associated with tumor grade. However, other clinical characteristics did not show a strong correlation with the risk score and were not statistically significant. This may be caused by insufficient samples in the subgroups divided by clinical characteristics. The grading of tumors is based on the appearance of tumor cells under the microscope: low-grade tumor cells are closer to normal cells than to high-grade tumor cells. The grading provides advice to physicians about the degree of infiltration of individual tumors and their rate of growth and spread. Overall, low-grade tumors grow more slowly, while high-grade tumors grow more rapidly. The rate of tumor infiltration and growth also has a great impact on the prognosis of patients, and our risk score was established based on the prognosis of patients too, so the relationship between tumor grade and risk score is within the understandable range.

New signaling pathways of IRF-1 have been found within tumor microenvironments and in metastatic sites [78]. PLCG1 overexpression is associated with tumor growth and poor survival in gliomas in adult patients [79]. The colony formation assay showed that GSDMC and GSDMD had cell growth inhibitory activity, which affected the growth rate of the cells [55]. The above literature suggests that the genes contained in our signature all affect tumor infiltration and growth. Therefore, we can speculate that it is because the altered expression and activity of these genes affect the rate of tumor cell infiltration and growth, ultimately leading to different tumor grades.

As researchers in clinical medicine, we were most interested in what benefits the above results can bring to patients and what type of treatment references can be provided for clinicians. As a result, we classified patients as high-risk or low-risk based on the expression of the three genes. The immune infiltration of patients in various risk categories was compared, and we investigated whether immunotherapy works differently in patients in various risk groups. We were surprised to note that tumor infiltration differed between risk categories of patients. Tumor cell invasion was more noticeable in the high-risk patients. The expression of immunological checkpoints is a crucial marker utilized in tumor immunotherapy. As a result, we investigated the expression of immunological checkpoints in patients from various risk categories. Immune checkpoint expression was substantially correlated with the degree of tumor cell infiltration and was higher in patients in the high-risk group than in those in the low-risk group. This finding suggests that the prognostic PRGs interfere with the immune response of the tumor. A study published in 2020 discovered that CD8+ T cells and natural killer (NK) cells boosted tumor clearance via the GSDMB–granzyme A axis. These data suggest that NK cells can trigger pyroptosis but have different axes in different cells [80]. Separate studies published the same year discovered that CD8+ T cells and NK cells both induce pyroptosis in tumor cells via granzyme B (an enzyme capable of cleaving GSDME) [81]. The GSDM family is still the focus of research on the link between CD8+ T cells and pyroptosis. An in vitro study found that GSDMD is required for CD8+ T cell antitumor activity [82]. In our study, CD8+ T cells varied across risk groups; the scores were lower in the high-risk group. This finding is also in accordance with our expectations.

However, few targetable immune checkpoints have been identified for PAAD compared to other tumors. Therefore, few immune checkpoint inhibitors are available. Fortunately, we still identified eight immune checkpoints with differential expression. Furthermore, our immune checkpoint gene study revealed that the low-risk group had greater levels of immune checkpoint gene expression (VTCN1 and TNFSF9). VTCN1 is a transmembrane protein expressed on the surface of some tumors and is considered to negatively regulate T cells, significantly inhibiting CD4+ T cell differentiation [83]. TNFSF9 (also known as CD137 or 4-1BB) is an inducible costimulatory receptor expressed on activated T cells and natural killer cells that can enhance effective function; combining PD-1/PD-L1 blockade with a CD137 agonist synergistically increased antitumor responses [84]. We hypothesize that a combination therapy targeting immune checkpoints and pyroptosis might be a novel therapeutic strategy. A review corroborated this idea, reporting that immune checkpoint inhibitors effectively killed immune-cold tumor cells only when pyroptosis was also induced. Similarly, pyroptosis induction alone was ineffective in inhibiting tumor growth, emphasizing the need to treat immune-cold tumors with a combination of pyroptosis activator and immune checkpoint inhibitors [85].

In addition, the GSVA results suggested that our prognostic PRGs may influence the metabolism of tumor cells. Glucose metabolism plays a crucial role in the body's environmental homeostasis. A variety of enzymes related to gluconeogenic intermediates have been shown to be closely related to the activation of NLRP3 [86]. Gao et al. found that hyperglycemia activates the NADPH-oxidase system, leading to the upregulation of ROS production and NLRP3 inflammatory vesicle activity [87]. ROS have been shown to induce NLRP3 inflammatory vesicle formation, triggering cellular pyroptotic death. Bacterial infections may cause cellular pyroptosis by affecting glucose metabolism. Salmonella can disrupt metabolism by taking up glucose from the host cell, reducing the level of reduced nicotinamide adenine dinucleotide (NADH) and leading to mitochondrial ROS production, which in turn triggers caspase-1-dependent cellular pyroptosis. It has been shown that restoration of NADH can save cells from pyroptosis [88]. The absence of NLRP3 inflammatory vesicles or treatment with the antioxidant N-acetylcysteine ameliorated islet cell damage caused by advanced glycation end products (AGEs) after NLRP3 activation [89]. We also found reports on pyroptosis and lipid metabolism. High homocysteine (HCY) is a risk factor for obesity, elevated serum cysteine levels have been found in obese patients [90], and high levels of HCY can cause pyroptosis in the presence and absence of LPS [90]. Researchers found that NLRP3-related inflammation was activated in the fat pads of leptin-deficient mice and high-fat diet-fed obese mice and that NLRP3-dependent caspase-1 activation in hypertrophic adipocytes may induce obesity and adipocyte death through pyroptosis [91]. A high-fat diet alters lipid metabolism and raises free fatty acid levels, which activates the NLRP3 inflammasome and causes caspase-1-dependent pyroptosis. Glucose metabolism disruptions generate additional ROS, which activate the NLRP3 inflammasome and caspase-1-dependent pyroptosis. HCY levels can also cause NLRP3 inflammasome activation and caspase-1-dependent pyroptosis.

Finally, in light of the importance of the tumor microenvironment in cancer treatment, we examined the sensitivity of the two groups of patients to routinely used anticancer drugs, and the results showed that the drug sensitivity differed between the two groups, with axitinib and camptothecin being more suitable for patients in the low-risk group and lapatinib being more suitable for patients in the high-risk group. We analyzed the origin and mechanism of the three drugs in DrugBank (https://go.drugbank.com/). Axitinib is a tyrosine kinase inhibitor of the second generation that operates by specifically blocking vascular endothelial growth factor receptors (VEGFR-1, VEGFR-2, and VEGFR-3) [92]. Camptothecin is an alkaloid extracted from the stem wood of Camptotheca acuminata, a Chinese tree. This chemical inhibits the nuclear enzyme DNA topoisomerase (type I) in a specific manner [93]. Lapatinib is a tyrosine kinase inhibitor of human epidermal growth factor receptor type 2 (HER2/ERBB2) and epidermal growth factor receptor (HER1/EGFR/ERBB1) [94]. A phase II clinical trial assessed lapatinib combined with capecitabine as a new choice for metastatic PAAD [95, 96]. Unfortunately, we did not find a clear pathway associated with pyroptosis, and thus, more exploration is needed. However, we found that the targets of both lapatinib and axitinib are tyrosine kinases. Therefore, our subsequent research will focus on the relationship between tyrosine kinases and pyroptosis.

We were interested in comparing our prediction model to existing pyroptosis-related models. In fact, no similar pyroptosis signature study has been reported in pancreatic cancer. We could only compare pyroptosis-related signatures in other solid tumors. In lung adenocarcinoma, a pyroptosis signature has been reported to be able to predict prognosis. However, the values of their AUC curves were small, and the AUC values in the first and second years were surprisingly less than 0.6, which indicates that the predictive power of this prediction model is not sufficient [97]. In hepatocellular carcinoma, some reports indicate that a pyroptosis signature has good predictive power with relatively high AUC values. However, the signature includes a total of 9 related genes. Theoretically, this signature may be more accurate, but the cost of detection in clinical practice will be much higher. In contrast, our signature has only three genes, which reduces the cost of testing in practical application in the clinic [98].

Nonetheless, there are certain general limits to our research. The major source of our data, as with other predictive model publications, was publicly available databases; hence, our work lacks validation using laboratory data and genuine clinical data. Although we validated our model with another dataset, this strategy cannot substitute for validation using real clinical data. As a result, further prospective studies are needed to evaluate the feasibility and real predictive value of the pyroptosis-related gene signature in clinical applications.

## 5. Conclusion

Our research described that pyroptosis is closely related to pancreatic cancer and established a predictive model consisting of three signature PRGs. The score from the model could predict overall survival in TCGA and ICGC cohorts. This score could be used to determine whether patients were sensitive to immunotherapy and which drug was most suitable. In addition, a nomogram was generated to predict patient survival. We hope this model can contribute to the precision treatment of pancreatic cancer in the future.

## Figures and Tables

**Table 1:** Distribution of characteristics of clinical samples in TCGA and IGCG databases.

	TCGA	ICGC
OS event		
Dead	58	116
Alive	119	53
Age		
<=65	93	63
>65	84	87
Unknown		19
Gender		
Female	80	76
Male	97	92
Unknown		1
Grade		
G1	31	15
G2	94	38
G3	48	23
G4	2	6
Unknown	2	87
Stage		
I	21	6
II	146	74
III	3	1
IV	4	1
Unknown	3	87
T stage		NA
T1	7	
T2	24	
T3	141	
T4	3	
Unknown	2	
N stage		NA
N0	49	
N1	123	
Unknown	5	
M stage		NA
M0	79	
M1	4	
Unknown	94	
Total	177	169

**Table 2:** Univariate Cox regression analysis of OS.

Gene	95% CI	95% CI lower	95% CI high	*p* value
GSDMC	1.132550775	1.061848796	1.207960363	0.000153929
IRF1	1.088101442	1.035478699	1.14339846	0.0008425
PLCG1	0.85612537	0.777047892	0.943250289	0.00168086

## Data Availability

All data generated or analyzed during this study are included in this published article and its supplementary information files.

## Supplementary Materials

Supplementary 1 Table S1: 481 DEGs between normal and tumor tissues.

Supplementary 2 Table S2: results of GO and KEGG analysis.

Supplementary 3 Table S3: the result of GSVA analysis.

Supplementary 4 Figure S1: expression difference of other 27 immune checkpoints between high- and low-risk group.

Supplementary 5 Figure S2: the predictive power of these three genes in other cancers.
