# Supplementary material for: Development of a Prognostic Model Based on Pyroptosis-Related Genes in Pancreatic Adenocarcinoma
Source: Dis Markers. 2022 May 29;2022:9141117. doi: 10.1155/2022/9141117 (PMC9169203; doi:10.1155/2022/9141117)
Supplement: Supplementary 1 — Table S1: 481 DEGs between normal and tumor tissues. [file 9141117.f1.pdf]

Table S1

id	logFC	AveExpr	t	adj.P.Val	P.Value	B
SIGLEC11	-1.18033	0.358111	-7.01368	4.48E-11	5.78E-08	14.69692
STAB2	-1.01441	0.188531	-5.85623	2.19E-08	1.11E-05	8.900629
CD33	-1.01299	0.691405	-5.46193	1.54E-07	5.61E-05	7.081999
HBA1	-1.08005	0.238181	-5.08332	9.21E-07	0.000246	5.420568
SOWAHD	-1.11713	0.869964	-5.0617	1.02E-06	0.000269	5.328318
RASGRP3	-1.13792	1.308507	-5.03187	1.17E-06	0.000292	5.201526
TFEC	-1.18031	0.734619	-5.00419	1.32E-06	0.000317	5.084374
CD68	-1.1302	0.973534	-4.95219	1.68E-06	0.000376	4.865576
CLECL1	-1.20748	0.51323	-4.87033	2.42E-06	0.000524	4.524639
GZMA	-1.47823	1.081629	-4.83925	2.78E-06	0.000579	4.396315
KEL	-1.03204	0.290412	-4.82632	2.95E-06	0.000604	4.343137
KLRF1	-1.01212	0.569993	-4.77549	3.69E-06	0.000733	4.135048
PARVG	-1.73874	1.573965	-4.75416	4.06E-06	0.000789	4.048228
GDPD5	-1.08617	1.555631	-4.73609	4.39E-06	0.000838	3.974935
MATK	-1.23222	0.91888	-4.70682	4.99E-06	0.000925	3.856628
AGR2	4.39935	6.893834	4.690099	5.37E-06	0.000958	3.789298
SIGLEC7	-1.25931	1.002212	-4.63139	6.92E-06	0.001213	3.554405
CX3CR1	-1.42765	0.892325	-4.53525	1.05E-05	0.001665	3.174683
GNGT2	-1.05051	1.02428	-4.50244	1.20E-05	0.001852	3.046537
ADAP2	-1.29034	1.875658	-4.49016	1.27E-05	0.001905	2.998778
P2RX7	-1.07935	1.041922	-4.46819	1.39E-05	0.002058	2.913567
BAALC	-1.00186	0.791721	-4.44638	1.52E-05	0.002179	2.829285
GAB3	-1.11803	1.108956	-4.40814	1.78E-05	0.002495	2.68234
MYO1F	-1.57978	2.033575	-4.40328	1.82E-05	0.002525	2.663752
DOK3	-1.48542	1.692232	-4.3798	2.01E-05	0.002741	2.574091
LILRA4	-1.24515	0.529409	-4.29072	2.90E-05	0.003556	2.237474
FADS3	-1.17681	2.077556	-4.28034	3.02E-05	0.003663	2.198632
AIM2	-1.11037	1.191384	-4.25977	3.29E-05	0.003934	2.12184
SCIMP	-1.52884	1.18793	-4.21112	4.00E-05	0.00451	1.941418
SIGLEC9	-1.20319	1.294977	-4.19724	4.23E-05	0.004687	1.890242
GAPT	-1.45139	0.934427	-4.18055	4.53E-05	0.004955	1.828917
TMEM131I	-1.42492	1.923222	-4.17604	4.61E-05	0.005017	1.812357
NPL	-1.26306	1.933729	-4.15238	5.06E-05	0.005392	1.72584
PLD4	-1.16546	0.916632	-4.13494	5.43E-05	0.005707	1.662292
P2RY13	-1.47319	1.14221	-4.11435	5.89E-05	0.006009	1.587591
LILRB2	-1.40812	1.421726	-4.10825	6.04E-05	0.006089	1.565512
TLR7	-1.15712	0.858733	-4.10422	6.13E-05	0.006124	1.550942
CEACAM21	-1.13031	0.938907	-4.09621	6.33E-05	0.006223	1.522022
CASP1	-1.42702	1.870362	-4.05065	7.57E-05	0.007187	1.358364
1-Mar	-1.09197	1.08626	-4.00999	8.87E-05	0.00799	1.213579
DPEP2	-1.32822	1.311917	-4.00111	9.19E-05	0.008194	1.182125
BTK	-1.65389	1.578263	-3.99945	9.24E-05	0.008209	1.176254
FGD2	-1.22557	1.243879	-3.98936	9.61E-05	0.008443	1.140602
CIITA	-1.49369	1.764208	-3.98866	9.64E-05	0.008443	1.138129
PRAM1	-1.21196	1.263649	-3.96065	0.000107	0.009015	1.039559
RUBCNL	-1.19743	0.934822	-3.95841	0.000108	0.009015	1.031676
RNF166	-1.05922	2.411873	-3.95073	0.000112	0.009228	1.004768
GPR65	-1.07736	0.996651	-3.93488	0.000119	0.00968	0.949343
FAM49A	-1.35087	1.907901	-3.93383	0.000119	0.00968	0.94571

EOMES	-1.02337	0.550118	-3.92998	0.000121	0.009783	0.932255
C19orf38	-1.07853	1.222025	-3.9122	0.000129	0.010341	0.870405
TLR1	-1.11146	1.512327	-3.86146	0.000157	0.012053	0.695118
CD180	-1.37059	1.093215	-3.85731	0.000159	0.012165	0.680859
CSF3R	-1.50373	1.525931	-3.84775	0.000165	0.012431	0.648095
TRAF1	-1.16675	1.972102	-3.84048	0.00017	0.012657	0.623192
LRRC25	-1.45172	2.012722	-3.83416	0.000174	0.012822	0.601592
WDFY4	-1.48629	1.063431	-3.83338	0.000174	0.012822	0.598943
PERP	1.673863	6.10352	3.829411	0.000177	0.012916	0.585391
NFAM1	-1.30003	1.738313	-3.81765	0.000185	0.013243	0.545314
TNFAIP8L2	-1.55995	2.199493	-3.81071	0.00019	0.013496	0.52172
PLXNA2	1.117304	2.737373	3.79245	0.000203	0.014219	0.459811
BCL11A	-1.01907	0.726188	-3.77322	0.000218	0.015076	0.394893
CR1	-1.03003	0.495877	-3.76027	0.000229	0.015595	0.351313
PSTPIP2	-1.19712	1.692729	-3.73655	0.00025	0.016845	0.271854
ADAMTS1(	-1.32335	1.972843	-3.7228	0.000263	0.017643	0.225971
STX11	-1.13779	1.573246	-3.72209	0.000264	0.017643	0.223626
TP53	-1.20605	1.173448	-3.7138	0.000272	0.01794	0.196022
MCOLN2	-1.08447	0.824295	-3.70576	0.00028	0.018166	0.16932
IL12RB1	-1.01859	1.094602	-3.67975	0.000308	0.019648	0.083283
PIK3R5	-1.30725	1.416306	-3.67717	0.000311	0.019758	0.074776
DOCK2	-1.46722	1.531073	-3.67639	0.000312	0.019758	0.072232
LAIR1	-1.51936	2.160242	-3.67193	0.000317	0.019867	0.057536
SMIM22	1.957261	4.727291	3.671301	0.000318	0.019867	0.055458
ATG16L2	-1.12311	1.897917	-3.67002	0.000319	0.019877	0.05125
S100P	4.532104	6.526922	3.66181	0.000329	0.020197	0.024253
CCL4L2	-1.53545	1.539281	-3.66078	0.00033	0.020197	0.020884
HVCN1	-1.27166	1.617675	-3.64876	0.000345	0.020949	-0.01854
APOBR	-1.35819	2.485205	-3.64322	0.000352	0.02124	-0.03668
FAM78A	-1.28984	1.48049	-3.62867	0.000371	0.021675	-0.08415
PLCB2	-1.51326	2.133986	-3.58667	0.000431	0.024469	-0.22034
DNASE1L3	-1.19217	0.574344	-3.58615	0.000432	0.024469	-0.22201
CD84	-1.36781	1.422977	-3.58231	0.000438	0.024591	-0.23441
PALD1	-1.21078	2.266572	-3.57924	0.000443	0.024735	-0.24429
CHI3L2	-1.35008	0.966065	-3.57112	0.000456	0.02501	-0.27043
FAM83E	2.406399	3.922469	3.568252	0.00046	0.025109	-0.27963
DOCK8	-1.33026	1.722185	-3.56762	0.000461	0.025109	-0.28165
AMTN	-1.5686	0.580396	-3.56422	0.000467	0.025201	-0.29256
RNF144B	-1.0779	2.241565	-3.56095	0.000472	0.025354	-0.30305
RENBP	-1.36819	2.670714	-3.54903	0.000493	0.026088	-0.34125
PIK3CD	-1.30891	2.095466	-3.54386	0.000502	0.026354	-0.35776
CAPN8	2.947615	4.049022	3.516112	0.000554	0.028678	-0.44605
NCF1	-1.2156	0.984382	-3.5098	0.000566	0.029166	-0.46606
MYO1G	-1.13298	1.26331	-3.49397	0.000598	0.030347	-0.51608
MAP3K8	-1.13298	2.227833	-3.49115	0.000604	0.030554	-0.52496
CAMK1D	-1.19789	1.984038	-3.49053	0.000606	0.030554	-0.52691
IRF1	2.65291	3.403888	3.465187	0.000662	0.032952	-0.60653
MUC13	2.951292	5.151257	3.463748	0.000665	0.033032	-0.61103
RAB42	-1.06933	1.466679	-3.45897	0.000676	0.033372	-0.62597
PSTPIP1	-1.09589	1.292481	-3.44681	0.000705	0.034503	-0.66395

STEAP2	1.101423	2.576224	3.443053	0.000715	0.034867	-0.67564
SPN	-1.29989	1.506878	-3.43763	0.000728	0.035349	-0.69252
TLR10	-1.24859	0.591108	-3.43587	0.000733	0.03546	-0.69798
KCNAB2	-1.11811	2.041968	-3.43528	0.000734	0.03546	-0.69984
BIN2	-1.32112	1.726731	-3.42682	0.000756	0.036331	-0.7261
IFFO1	-1.01726	2.256532	-3.42096	0.000771	0.036981	-0.74424
SIGLEC10	-1.53414	1.912802	-3.41875	0.000777	0.037079	-0.75108
POU2F2	-1.07744	0.94003	-3.41279	0.000794	0.037478	-0.7695
NRROS	-1.08395	1.42659	-3.40862	0.000805	0.037742	-0.78239
SERPINB5	2.520371	3.434883	3.402756	0.000821	0.038233	-0.80049
LCP2	-1.34266	2.170879	-3.3985	0.000833	0.038684	-0.81358
BAIAP2L2	2.382142	3.895569	3.396693	0.000839	0.038757	-0.81916
BCL2	-1.18758	1.446044	-3.3905	0.000857	0.0394	-0.8382
PDE7A	-1.1766	2.268587	-3.38701	0.000867	0.039685	-0.84893
SCNN1G	-1.41262	0.739288	-3.38538	0.000872	0.039728	-0.85393
TLCD1	1.151904	2.890138	3.383985	0.000876	0.039728	-0.8582
IGSF6	-1.43799	2.175704	-3.3761	0.0009	0.040514	-0.88237
SDR16C5	2.303876	3.061898	3.363417	0.00094	0.041631	-0.92112
CD300LF	-1.41804	1.891238	-3.35453	0.000969	0.042449	-0.94821
LAT2	-1.24123	2.168763	-3.34372	0.001005	0.043364	-0.98105
IRF2	-1.24336	1.8576	-3.34119	0.001014	0.043364	-0.98873
NPY1R	-1.21467	0.971785	-3.34079	0.001015	0.043364	-0.98994
KLHL6	-1.24112	1.178263	-3.33133	0.001048	0.044578	-1.0186
DOCK10	-1.15718	1.443065	-3.3216	0.001083	0.045867	-1.04801
SHANK3	-1.05855	2.23681	-3.31158	0.00112	0.047131	-1.0782
VAV3	-1.1412	1.271273	-3.30236	0.001155	0.048092	-1.10592
RASGRP1	-1.21295	1.425864	-3.29976	0.001166	0.048268	-1.11371
TLR8	-1.01169	0.835723	-3.29934	0.001167	0.048268	-1.11496
LILRB4	-1.32439	1.743429	-3.28337	0.001231	0.050418	-1.16278
FLI1	-1.17165	1.706667	-3.28316	0.001232	0.050418	-1.16341
RASAL3	-1.39291	1.79006	-3.27599	0.001262	0.051099	-1.18481
EPHB3	1.493113	2.877361	3.244436	0.001402	0.054799	-1.27845
CXorf21	-1.07371	1.212769	-3.24349	0.001406	0.05486	-1.28124
MSLN	3.733287	5.896286	3.236816	0.001438	0.055435	-1.30095
CD300C	-1.00101	1.356099	-3.23052	0.001468	0.056242	-1.3195
CELF2	-1.37511	1.85905	-3.22115	0.001514	0.057326	-1.34706
STYK1	1.379954	1.949745	3.218561	0.001527	0.057704	-1.35465
DOK2	-1.32978	2.336376	-3.21254	0.001558	0.058377	-1.37231
CDS1	1.181389	3.183767	3.211943	0.001561	0.058377	-1.37406
AOAH	-1.4334	1.779706	-3.20168	0.001614	0.059519	-1.40409
MYEOV	2.539241	3.323241	3.199384	0.001627	0.059663	-1.41078
TFF1	4.988691	7.529882	3.197445	0.001637	0.059663	-1.41644
CETP	-1.01002	0.87353	-3.19298	0.001661	0.060247	-1.42945
TFF2	4.431589	6.058127	3.191438	0.00167	0.060247	-1.43395
STX1A	1.552546	3.277815	3.191438	0.00167	0.060247	-1.43395
ARHGEF16	1.422491	3.402142	3.183333	0.001714	0.061292	-1.45753
TMEM273	-1.08302	1.750032	-3.17884	0.00174	0.061516	-1.47058
TRAF3IP3	-1.14116	0.96654	-3.17576	0.001757	0.061793	-1.4795
FCGR2B	-1.02228	1.260825	-3.17152	0.001782	0.062313	-1.49179
C1orf210	1.32334	3.494263	3.166507	0.001811	0.06311	-1.50631

MNDA	-1.60172	2.446067	-3.15992	0.00185	0.064075	-1.52535
CCL3L1	-1.07563	1.00609	-3.15963	0.001852	0.064075	-1.52619
PILRA	-1.27432	2.287261	-3.14781	0.001925	0.065755	-1.56026
EPS8L3	2.523512	4.250237	3.14627	0.001934	0.06585	-1.56468
GIMAP1	-1.03134	1.368052	-3.14548	0.001939	0.065902	-1.56695
PAPLN	-1.25245	2.083255	-3.13307	0.002019	0.068359	-1.60257
CLEC7A	-1.21896	1.788413	-3.12092	0.002099	0.070383	-1.63733
ZNF486	-1.0546	1.336133	-3.11707	0.002125	0.070981	-1.64832
ETV4	1.529234	3.102408	3.115193	0.002138	0.070985	-1.65366
C19orf33	2.956421	6.719931	3.109839	0.002175	0.071467	-1.66891
AQP5	3.13276	4.181649	3.107863	0.002189	0.071744	-1.67454
SLC15A3	-1.18256	2.966613	-3.09352	0.002292	0.07411	-1.71525
GPRC5A	2.751295	5.598289	3.089776	0.00232	0.074502	-1.72585
PLCG1	1.721044	3.820182	3.086516	0.002344	0.074615	-1.73507
IL27RA	-1.00244	2.61824	-3.08622	0.002346	0.074615	-1.7359
CD1D	-1.17092	1.539477	-3.08618	0.002346	0.074615	-1.73601
STEAP1	1.445222	3.705888	3.08154	0.002382	0.075433	-1.74913
GIMAP8	-1.21779	1.994401	-3.07904	0.002401	0.075711	-1.75618
MANSC1	1.018486	3.273489	3.077065	0.002416	0.07594	-1.76175
TOX3	1.339434	2.180353	3.070688	0.002465	0.077245	-1.77971
CCR1	-1.30634	2.027739	-3.06478	0.002512	0.078206	-1.79633
GALNT5	1.894807	2.779412	3.064759	0.002512	0.078206	-1.79638
CCL4	-1.3012	1.793344	-3.06422	0.002516	0.078214	-1.7979
FERMT1	1.664923	3.395695	3.061041	0.002542	0.078406	-1.80682
SLC2A6	-1.03857	2.122444	-3.05876	0.00256	0.078691	-1.81321
LRRN2	-1.04716	1.333644	-3.05423	0.002597	0.079324	-1.82591
CD86	-1.28074	2.196421	-3.04742	0.002654	0.08029	-1.84497
PPL	1.601911	4.185422	3.03791	0.002735	0.082094	-1.87151
BAIAP2	1.024611	2.702987	3.037293	0.00274	0.082126	-1.87323
IQANK1	1.413324	2.48084	3.03339	0.002774	0.082756	-1.8841
SFN	2.793027	6.19994	3.032575	0.002781	0.082841	-1.88637
PLEKHA6	1.218906	3.802962	3.025326	0.002846	0.083919	-1.90652
ITGA4	-1.11266	1.339849	-3.02057	0.002888	0.08459	-1.9197
PPP1R14D	2.086011	2.552903	3.019011	0.002903	0.084878	-1.92404
FCRL2	-1.07104	0.394005	-3.01812	0.002911	0.084985	-1.92649
VSIG2	2.810892	5.190837	3.01735	0.002918	0.085063	-1.92864
ZAP70	-1.272	1.167883	-3.01678	0.002923	0.085088	-1.93023
HIST2H2BE	1.527689	3.49877	3.010237	0.002984	0.086071	-1.94832
PRKAR2B	-1.17376	1.91674	-2.98743	0.003204	0.091199	-2.01115
TMC4	1.354045	5.111578	2.98072	0.003272	0.092359	-2.02955
JAK3	-1.16148	2.177506	-2.97819	0.003297	0.092627	-2.03647
MAB21L4	1.540494	2.013164	2.973382	0.003347	0.093885	-2.04963
OVOL2	1.105518	2.308261	2.969316	0.00339	0.094801	-2.06074
CKMT1A	1.10017	1.458783	2.964774	0.003438	0.095866	-2.07313
GSDMC	1.826796	3.929475	2.962457	0.003462	0.096139	-2.07945
LAMA3	1.876476	3.885905	2.961821	0.003469	0.09619	-2.08118
FPR1	-1.5078	2.556967	-2.94897	0.00361	0.099372	-2.11612
SH2D3C	-1.01073	2.146078	-2.94358	0.00367	0.100136	-2.13072
FYB1	-1.42648	2.147446	-2.93954	0.003716	0.10072	-2.14168
PRKCB	-1.23991	1.012945	-2.93833	0.00373	0.100952	-2.14493

TUFT1	1.155307	3.57482	2.932319	0.0038	0.102119	-2.16118
CBLC	1.704237	4.435955	2.930581	0.00382	0.102237	-2.16588
SAMSN1	-1.27368	2.014304	-2.92957	0.003832	0.102271	-2.1686
CYFIP2	-1.1628	2.026328	-2.92681	0.003864	0.102857	-2.17605
AREG	2.115515	3.769233	2.924916	0.003887	0.10317	-2.18115
SH3RF2	1.296353	2.408859	2.923887	0.003899	0.103354	-2.18392
TMEM125	1.171083	3.590633	2.917615	0.003975	0.104782	-2.20079
EBI3	-1.18825	1.51157	-2.90024	0.004191	0.108914	-2.24736
PSCA	4.090589	4.952495	2.89428	0.004268	0.1104	-2.26327
GZMH	-1.32893	1.630726	-2.89155	0.004304	0.111171	-2.27055
ARHGEF6	-1.0885	2.037992	-2.89086	0.004313	0.111255	-2.27238
TRPV2	-1.06295	2.944888	-2.88997	0.004324	0.111408	-2.27476
SYT13	1.758583	3.910811	2.88875	0.004341	0.111671	-2.27801
CKMT1B	1.120157	1.525855	2.887585	0.004356	0.111769	-2.28111
HSPB8	-1.36295	2.889229	-2.88484	0.004392	0.112159	-2.28843
CCR5	-1.21141	1.536554	-2.88476	0.004393	0.112159	-2.28862
TNFSF8	-1.07436	0.932272	-2.88375	0.004407	0.112182	-2.29132
AGR3	2.450536	4.396762	2.881638	0.004435	0.112615	-2.29693
TNS4	2.580521	3.529673	2.88047	0.004451	0.112707	-2.30003
SLC37A2	-1.08549	1.679495	-2.87509	0.004524	0.113516	-2.31431
KLRB1	-1.4047	1.835549	-2.87416	0.004537	0.113685	-2.31676
GLIPR1	-1.11694	2.338643	-2.8734	0.004547	0.113801	-2.3188
GNG7	-1.20735	1.884914	-2.8628	0.004695	0.116007	-2.34682
TNF	-1.03215	1.970844	-2.86234	0.004702	0.116007	-2.34805
GOLT1A	1.307242	2.704399	2.859886	0.004737	0.116127	-2.35453
BAIAP2L1	1.377532	4.47948	2.857721	0.004768	0.116731	-2.36024
SDC1	1.762254	6.479449	2.853803	0.004824	0.11764	-2.37056
SLPI	2.373393	7.756588	2.853483	0.004829	0.11764	-2.37141
HAVCR2	-1.22527	2.431438	-2.84324	0.00498	0.119879	-2.39833
KRT8	1.546078	8.282877	2.842202	0.004995	0.119879	-2.40106
COL17A1	3.005202	4.345785	2.841083	0.005012	0.119879	-2.404
PYGB	1.232825	5.954027	2.835996	0.005089	0.120561	-2.41733
NMU	2.116493	2.617234	2.835767	0.005093	0.120561	-2.41793
TRIM15	1.774713	2.554413	2.832402	0.005144	0.121484	-2.42674
ATP1B1	1.319412	7.241751	2.825994	0.005244	0.122632	-2.44348
SEZ6L2	1.160552	4.637596	2.815253	0.005415	0.125558	-2.47147
SLC4A11	1.457142	2.230501	2.808932	0.005517	0.127568	-2.4879
TM4SF5	2.407407	3.54129	2.80453	0.00559	0.128391	-2.49932
IKZF1	-1.23636	1.332332	-2.79139	0.005812	0.131765	-2.5333
ZG16B	2.021161	3.191195	2.789329	0.005848	0.132106	-2.53862
CHIT1	-1.70105	1.339481	-2.78256	0.005966	0.134145	-2.55606
PLA2G7	-1.41438	2.324357	-2.78083	0.005996	0.134363	-2.56052
TMEM54	1.154321	5.64707	2.77768	0.006052	0.13499	-2.56862
CLRN3	2.053469	2.738093	2.767315	0.00624	0.138055	-2.59521
ESRP2	1.089543	2.944604	2.765967	0.006265	0.138286	-2.59866
RASL11B	-1.05294	1.724215	-2.76091	0.006359	0.13908	-2.61158
NLRC4	2.626986	4.076057	2.758711	0.0064	0.139192	-2.61721
FMNL1	-1.05272	2.558248	-2.75521	0.006466	0.139838	-2.62613
PITX1	1.904083	2.724011	2.753284	0.006502	0.140473	-2.63105
CDHR5	2.027152	2.956126	2.751175	0.006543	0.141027	-2.63643

BCAS1	1.879451	2.861526	2.748191	0.0066	0.142105	-2.64402
SP140	-1.03033	0.957736	-2.74466	0.006669	0.142625	-2.65301
TRIM29	2.366632	3.585312	2.739793	0.006764	0.143629	-2.66536
AKAP7	-1.31285	1.945418	-2.73961	0.006768	0.143629	-2.66584
CCL3	-1.11105	1.623692	-2.73319	0.006896	0.145382	-2.6821
SMAGP	1.137472	3.181393	2.725406	0.007054	0.148098	-2.70178
GPR34	-1.29232	1.993125	-2.72533	0.007055	0.148098	-2.70198
TMEM30B	1.088929	3.995396	2.722599	0.007111	0.148614	-2.70887
SIGLEC14	-1.22674	1.354209	-2.71289	0.007315	0.150192	-2.73333
IRF6	1.166223	4.446178	2.702541	0.007537	0.152879	-2.7593
CDCP1	1.233773	4.053172	2.70083	0.007574	0.153476	-2.76359
IQGAP3	1.232842	2.112166	2.696899	0.007661	0.154577	-2.77342
MUC5B	2.544291	3.210792	2.691402	0.007783	0.15652	-2.78716
CYSTM1	1.375992	6.391773	2.691398	0.007783	0.15652	-2.78717
ASPHD1	1.290041	3.396751	2.690721	0.007798	0.156534	-2.78886
SPINT2	1.120525	6.751568	2.687463	0.007872	0.157353	-2.79698
HIST1H1C	1.572258	5.772911	2.671809	0.008234	0.162377	-2.8359
SLAMF8	-1.2573	2.661274	-2.67149	0.008241	0.162377	-2.83669
ARL14	2.255795	3.939378	2.664239	0.008414	0.164604	-2.85465
TAGAP	-1.12946	1.564343	-2.66286	0.008447	0.164921	-2.85805
KDELR3	1.165025	4.435358	2.661589	0.008478	0.165351	-2.8612
JAML	-1.09722	1.711874	-2.66037	0.008507	0.165426	-2.86422
MYH14	1.411382	4.968232	2.65654	0.008601	0.166739	-2.87367
ECT2	1.052246	2.707372	2.650229	0.008757	0.168918	-2.88921
CEACAM5	3.904918	5.917084	2.649376	0.008778	0.169157	-2.89131
AK4	1.195213	2.209801	2.643647	0.008922	0.170108	-2.90539
IL2RB	-1.0571	1.676988	-2.64356	0.008925	0.170108	-2.90562
HIST1H2BC	1.277395	2.037065	2.641825	0.008969	0.170734	-2.90987
ACSM3	-1.06319	1.609952	-2.6319	0.009225	0.173796	-2.93418
SUSD3	-1.08169	1.753501	-2.6314	0.009238	0.173843	-2.93541
CORO2A	1.34971	3.386274	2.625637	0.00939	0.176013	-2.94948
CD247	-1.02996	1.288699	-2.6245	0.00942	0.176067	-2.95226
TSTA3	1.134247	5.343461	2.615958	0.00965	0.179154	-2.97306
ANKS4B	1.324711	1.667027	2.613562	0.009715	0.179771	-2.97888
PHLDA2	2.012052	4.862767	2.613217	0.009725	0.179771	-2.97972
NOD1	1.49288	3.723495	2.608652	0.009851	0.180095	-2.9908
TSPAN8	2.347181	6.55077	2.602412	0.010025	0.182749	-3.00592
ID1	1.571531	5.863948	2.601764	0.010043	0.182749	-3.00749
MUCL3	3.355299	4.056973	2.596332	0.010197	0.185206	-3.02061
ADGRF1	1.256589	1.676143	2.593611	0.010275	0.18625	-3.02718
TPSAB1	1.351121	3.177492	2.591952	0.010323	0.186264	-3.03118
SLC6A8	1.497744	3.807262	2.586131	0.010493	0.18809	-3.04521
RAPGEFL1	1.65316	3.213684	2.582837	0.01059	0.188351	-3.05313
HNF4A	1.190828	3.086836	2.582706	0.010594	0.188351	-3.05344
STARD10	1.042007	4.744593	2.581992	0.010615	0.188351	-3.05516
ADCY5	-1.00154	1.550345	-2.57717	0.010759	0.189852	-3.06672
SLC39A4	1.504142	3.176612	2.574384	0.010843	0.190709	-3.07341
FCGR3B	-1.07118	0.792297	-2.57424	0.010847	0.190709	-3.07374
GZMM	-1.10869	1.311501	-2.57307	0.010882	0.190809	-3.07655
HRASLS2	1.827494	2.247299	2.569133	0.011002	0.192054	-3.08598

EVPL	1.616394	3.193904	2.567871	0.011041	0.192541	-3.089
GCNT3	2.043299	4.204002	2.566856	0.011072	0.192737	-3.09142
EPHA2	1.691633	5.035453	2.559048	0.011315	0.194009	-3.11005
EVI2A	-1.19837	2.384982	-2.559	0.011316	0.194009	-3.11017
CYP2C18	1.639669	2.296978	2.553588	0.011487	0.195204	-3.12305
AOC1	1.984035	4.021138	2.550472	0.011587	0.196203	-3.13046
SEMA4G	1.213442	2.906316	2.547707	0.011676	0.197189	-3.13702
GPX2	2.24774	6.923819	2.546007	0.011731	0.197771	-3.14105
HTR1D	1.094691	1.457604	2.543156	0.011824	0.198466	-3.14781
ITGA3	1.384617	5.304145	2.541937	0.011863	0.198657	-3.15069
KRT18	1.351603	7.846318	2.541548	0.011876	0.198657	-3.15162
ATP2C2	1.077761	1.768296	2.5382	0.011986	0.199693	-3.15954
OSCAR	-1.10664	2.240498	-2.53784	0.011998	0.199693	-3.16038
C6orf132	1.125902	2.8337	2.53491	0.012095	0.20024	-3.16731
TRIM31	1.922296	2.912943	2.526812	0.012368	0.20249	-3.18641
SPIRE2	1.172926	1.997319	2.525952	0.012397	0.202758	-3.18844
TLR2	-1.00768	2.549985	-2.51323	0.012837	0.206741	-3.21831
PLCD3	1.247272	3.284774	2.512034	0.012879	0.207167	-3.22112
NGEF	1.092006	2.263525	2.511224	0.012908	0.207323	-3.22301
KLK11	1.92882	3.531978	2.500086	0.013307	0.212267	-3.24904
DMBT1	2.774281	3.54291	2.496004	0.013455	0.213756	-3.25855
TMC7	1.018567	1.941467	2.495018	0.013492	0.21388	-3.26084
IL16	-1.00178	1.46709	-2.48803	0.01375	0.215596	-3.27708
FA2H	1.595493	3.75155	2.48706	0.013787	0.215816	-3.27934
CEACAM1	1.294036	3.599048	2.482218	0.013969	0.217258	-3.29056
AMN	1.662884	2.377541	2.478897	0.014095	0.218166	-3.29825
KCNN4	1.889996	4.55898	2.476663	0.014181	0.218612	-3.30341
CRTAC1	-1.03582	1.267412	-2.47071	0.014411	0.22055	-3.31717
AADAC	1.634972	2.136183	2.467514	0.014536	0.221956	-3.32452
FAM129C	-1.09026	0.47697	-2.46437	0.01466	0.222512	-3.33176
SLCO2B1	-1.16352	2.832056	-2.46087	0.014799	0.223763	-3.3398
NLRP3	2.784323	4.635135	2.451079	0.015194	0.227128	-3.36226
OVOL1	1.077584	1.52023	2.447703	0.015332	0.228184	-3.36999
LSR	1.183113	6.562062	2.447646	0.015335	0.228184	-3.37012
ITGAM	-1.03296	2.413058	-2.44662	0.015377	0.228634	-3.37245
SIGLEC1	-1.04523	1.584516	-2.4462	0.015394	0.228717	-3.37342
TMPRSS3	1.348987	3.089397	2.443736	0.015496	0.229702	-3.37905
ITIH5	-1.10481	2.14831	-2.44291	0.015531	0.229966	-3.38094
PLA2G10	1.466295	2.044724	2.442698	0.015539	0.229966	-3.38142
CMKLR1	-1.00873	1.840774	-2.44094	0.015613	0.230364	-3.38542
MBOAT2	1.013546	3.029598	2.438992	0.015694	0.231394	-3.38987
C3AR1	-1.26405	2.98097	-2.43765	0.015751	0.231997	-3.39292
EFNA1	1.271386	5.331603	2.435459	0.015843	0.231997	-3.39791
RUNX3	-1.15552	2.084979	-2.43545	0.015844	0.231997	-3.39792
SPIB	-1.37635	1.467879	-2.4277	0.016175	0.235314	-3.41555
GIMAP6	-1.10921	2.33749	-2.4273	0.016192	0.235314	-3.41644
KRT15	1.196103	1.678607	2.425433	0.016273	0.235957	-3.42068
MUC5AC	2.649871	3.195506	2.417481	0.016622	0.238316	-3.43868
PI3	2.502529	4.730764	2.415726	0.016699	0.238544	-3.44264
P2RY8	-1.04372	1.546545	-2.4153	0.016718	0.23864	-3.44361

FGFBP1	1.905455	2.339972	2.413087	0.016817	0.239335	-3.4486
AKR7A3	1.69571	3.522874	2.412306	0.016852	0.239396	-3.45036
ANLN	1.129465	2.222164	2.412158	0.016858	0.239396	-3.45069
CEMIP	1.539322	2.928196	2.407747	0.017057	0.241148	-3.46063
MROH6	1.472933	2.948907	2.405418	0.017162	0.242112	-3.46587
KCNJ5	-1.05156	1.078706	-2.40536	0.017165	0.242112	-3.46601
MS4A6A	-1.20314	2.953383	-2.40339	0.017255	0.243059	-3.47044
KLK6	2.608809	3.833298	2.400357	0.017394	0.244271	-3.47724
FOSL1	1.544576	3.405555	2.399561	0.017431	0.244271	-3.47903
FOXA1	1.071886	1.603711	2.399513	0.017433	0.244271	-3.47914
TMEM92	1.260573	2.802108	2.399303	0.017442	0.244271	-3.47961
EPS8L2	1.060106	4.505732	2.398175	0.017495	0.244725	-3.48214
CDHR2	1.80481	2.892204	2.397829	0.017511	0.244725	-3.48292
IHH	1.830813	2.390936	2.394907	0.017646	0.245875	-3.48947
XDH	1.237595	2.006858	2.393255	0.017723	0.246415	-3.49316
CRYBG2	1.236753	2.031705	2.392344	0.017766	0.246653	-3.4952
F2RL1	1.208401	4.478909	2.390669	0.017845	0.247388	-3.49895
GALNT12	1.120978	3.498099	2.390051	0.017874	0.24748	-3.50033
CLDN7	1.199736	5.591151	2.385808	0.018075	0.248611	-3.50981
HIST1H4I	1.002677	2.631677	2.385407	0.018094	0.248696	-3.51071
LMO7	1.026678	3.760727	2.379851	0.018361	0.251202	-3.52309
MMP28	1.478476	3.759104	2.371269	0.018779	0.254289	-3.54217
HIST1H2Bk	1.237727	5.358914	2.364638	0.019108	0.256592	-3.55687
SIT1	-1.18279	1.393868	-2.35998	0.019342	0.258654	-3.56717
ALG1L	1.20567	1.600266	2.356984	0.019494	0.259971	-3.57378
PDLIM3	-1.12934	2.793494	-2.35034	0.019834	0.262573	-3.58842
SLC1A3	-1.02707	1.778049	-2.34263	0.020237	0.266367	-3.60537
TRNP1	1.319497	4.812088	2.342068	0.020266	0.26639	-3.6066
GJB5	1.707743	2.123112	2.341154	0.020314	0.26669	-3.6086
SCEL	1.575443	2.190472	2.339564	0.020398	0.267035	-3.61209
GSDME	1.040202	6.208546	2.339185	0.020418	0.267117	-3.61292
LRRC66	1.17617	1.502901	2.332823	0.020758	0.269547	-3.62684
S100A14	2.210407	6.4625	2.332051	0.0208	0.269603	-3.62853
ITGA6	1.077943	5.254581	2.324076	0.021233	0.27205	-3.64593
CREB3L1	1.393182	4.947726	2.319293	0.021497	0.274341	-3.65633
ELF3	1.511303	5.834647	2.319098	0.021508	0.274341	-3.65676
S1PR4	-1.06424	1.848913	-2.31368	0.02181	0.276543	-3.66852
F12	1.04272	1.879898	2.310104	0.022012	0.278144	-3.67627
CSF2RB	-1.12286	2.134882	-2.29567	0.022843	0.282078	-3.70744
S100A10	1.182722	8.171254	2.293893	0.022947	0.282642	-3.71126
ABO	1.341263	2.422669	2.290938	0.023121	0.283241	-3.71761
INAVA	1.351225	3.575303	2.289805	0.023188	0.283882	-3.72004
CLDN4	1.55861	6.178514	2.288019	0.023294	0.284785	-3.72388
LPCAT4	1.227567	4.543175	2.286247	0.0234	0.285332	-3.72768
SLC6A14	1.849393	3.058517	2.283996	0.023534	0.285765	-3.73251
CTSE	2.677685	7.182188	2.283392	0.023571	0.285844	-3.7338
MIA	1.965957	2.651838	2.281923	0.023659	0.286377	-3.73694
CA9	2.558027	4.005191	2.279364	0.023814	0.287347	-3.74242
GPR35	1.239291	2.627151	2.271684	0.024284	0.289751	-3.75881
CYTIP	-1.11343	2.362492	-2.26909	0.024444	0.290406	-3.76434

SDCBP2	1.452614	4.747241	2.264672	0.02472	0.2926	-3.77374
FAM83A	1.829871	2.129048	2.258901	0.025084	0.295637	-3.78599
CRACR2B	1.347856	3.388587	2.258305	0.025122	0.295902	-3.78725
HIST1H2A(	1.10412	4.506357	2.256752	0.025221	0.296082	-3.79054
FAM3D	2.284956	3.605048	2.254478	0.025366	0.296871	-3.79536
RNF39	1.157903	2.194566	2.250399	0.025629	0.298949	-3.80399
ENTPD8	1.290208	1.78448	2.249346	0.025697	0.299486	-3.80621
ALDH3A1	1.450366	1.763722	2.2492	0.025706	0.299486	-3.80652
RASAL1	1.085903	2.436648	2.24771	0.025803	0.300193	-3.80966
UGT1A10	1.527844	1.949856	2.231883	0.026851	0.307803	-3.84295
LYVE1	-1.05815	1.362249	-2.23042	0.02695	0.30857	-3.84602
RFLNA	1.439718	3.228775	2.227823	0.027126	0.309846	-3.85146
ST6GALNA	1.698747	3.523518	2.224941	0.027322	0.311721	-3.85748
LYPD1	-1.10075	1.711399	-2.22271	0.027475	0.312658	-3.86214
LEMD1	1.327157	2.020189	2.214451	0.028048	0.315844	-3.87936
PRSS3	2.200226	5.027826	2.205978	0.028646	0.319255	-3.89696
ALKAL2	-1.21605	1.351751	-2.20242	0.0289	0.320431	-3.90433
DDC	1.475118	1.750692	2.20085	0.029013	0.321424	-3.90758
VILL	1.517724	3.949155	2.197837	0.029231	0.322246	-3.91381
CLDN18	3.140873	4.827072	2.195684	0.029388	0.32314	-3.91826
CAVIN2	-1.08182	2.521697	-2.19557	0.029396	0.32314	-3.9185
AKR1B10	2.187531	3.640307	2.194634	0.029464	0.323704	-3.92042
GDA	1.168233	2.527319	2.190721	0.029751	0.325077	-3.92849
MS4A8	1.543328	2.184849	2.189655	0.02983	0.325596	-3.93069
MSR1	-1.08234	2.369415	-2.18254	0.030359	0.327318	-3.94531
SLCO4A1	1.29894	2.686097	2.182424	0.030368	0.327318	-3.94555
PLA2G16	1.051453	4.904272	2.18033	0.030525	0.328062	-3.94984
LGALS4	2.321921	6.766794	2.179841	0.030562	0.328112	-3.95085
SLC11A1	-1.00407	2.046973	-2.16973	0.031332	0.33138	-3.97154
B4GALNT3	1.049165	3.574495	2.165514	0.031658	0.33294	-3.98013
NPC1L1	1.371843	2.644674	2.16124	0.031992	0.335046	-3.98883
CCDC69	-1.03479	2.665343	-2.15941	0.032136	0.335529	-3.99255
MET	1.247284	4.156663	2.156498	0.032366	0.337188	-3.99846
C4BPB	1.252371	1.960204	2.154012	0.032563	0.337722	-4.0035
TFF3	2.648285	5.981215	2.14845	0.033009	0.339965	-4.01476
CAPN9	1.708454	2.217362	2.146262	0.033185	0.341056	-4.01918
SPINK5	1.005719	1.280839	2.134012	0.03419	0.345846	-4.04387
CYP4F3	1.123151	1.78755	2.132948	0.034278	0.346194	-4.046
GALE	1.028311	4.045716	2.13134	0.034412	0.347184	-4.04923
MGST1	1.007998	3.013896	2.127136	0.034765	0.349096	-4.05766
SHH	1.161245	1.957059	2.124936	0.03495	0.350049	-4.06206
CD1C	-1.18907	1.94333	-2.12341	0.03508	0.350616	-4.06512
FOXL1	1.32315	2.709618	2.120571	0.035322	0.351392	-4.07079
EFNA3	1.13162	2.156216	2.11947	0.035416	0.351803	-4.07299
NCAM1	-1.03256	1.096501	-2.11639	0.03568	0.353323	-4.07913
EHF	1.198039	3.826009	2.116033	0.035711	0.353441	-4.07984
FAM83D	1.115318	2.159229	2.111773	0.03608	0.356017	-4.08833
SYT8	1.950121	3.418448	2.110155	0.036221	0.356081	-4.09154
MPZL2	1.172565	4.014916	2.104865	0.036685	0.358858	-4.10205
F5	1.445703	2.703796	2.104387	0.036727	0.358997	-4.103

KRT16	2.207135	3.836958	2.095291	0.037538	0.362782	-4.12099
CPA3	1.302266	3.198045	2.086235	0.038361	0.367091	-4.13884
FUT3	1.418958	4.02293	2.085556	0.038423	0.36745	-4.14017
C9orf152	1.335785	2.729067	2.078662	0.03906	0.369672	-4.1537
CYP3A5	1.448802	3.768728	2.07331	0.039561	0.371735	-4.16418
MYRF	1.048532	3.967406	2.068321	0.040033	0.373332	-4.17392
UBE2C	1.21948	3.699031	2.065275	0.040324	0.374298	-4.17986
NAPRT	1.107564	4.417809	2.052803	0.041532	0.380284	-4.20408
TMEM238	1.103042	2.715998	2.049666	0.041841	0.381581	-4.21015
VSIG1	2.035395	2.947758	2.049001	0.041907	0.381733	-4.21143
TMC5	1.386727	4.254849	2.046076	0.042197	0.382653	-4.21708
APOBEC1	1.483655	1.839146	2.036756	0.043133	0.385652	-4.23504
PADI1	2.016432	2.584981	2.036484	0.04316	0.385652	-4.23556
SLC2A1	1.501424	5.347178	2.032945	0.04352	0.385652	-4.24236
KLK7	2.195014	3.382465	2.031949	0.043622	0.385981	-4.24427
GZMK	-1.14685	1.675011	-2.02803	0.044025	0.388796	-4.25177
FCN1	-1.09591	1.272393	-2.02701	0.044131	0.38902	-4.25374
SLC5A1	1.309972	3.089471	2.025227	0.044315	0.389751	-4.25714
HSPA1B	1.164059	4.896876	2.024426	0.044398	0.390304	-4.25867
HLA-DRB5	-1.41097	6.556056	-2.0215	0.044703	0.390847	-4.26426
WNT7B	1.018572	2.240428	2.020996	0.044755	0.391127	-4.26522
TPSB2	1.228141	3.21238	2.01501	0.045385	0.39305	-4.27662
AHNAK2	1.257772	2.944915	2.014448	0.045445	0.39305	-4.27769
CPVL	-1.03918	3.217338	-2.00594	0.046354	0.396806	-4.29384
VSTM2L	1.724216	4.093742	2.002448	0.046731	0.397189	-4.30045
MMP1	2.28372	5.433185	1.997067	0.047318	0.399219	-4.31061
SFTA2	1.799108	4.316565	1.995428	0.047498	0.399907	-4.3137
GJC2	1.020088	1.763996	1.991075	0.047979	0.402329	-4.3219
GJB4	1.07428	1.900193	1.980639	0.049148	0.408466	-4.34148
CCR7	-1.27951	1.726426	-1.98057	0.049156	0.408466	-4.3416
FPR3	-1.11367	2.786241	-1.9792	0.049311	0.409404	-4.34416
MYO1A	1.334634	1.809085	1.97556	0.049726	0.411433	-4.35097
