# Supplementary material for: Development of a Prognostic Model Based on Pyroptosis-Related Genes in Pancreatic Adenocarcinoma
Source: Dis Markers. 2022 May 29;2022:9141117. doi: 10.1155/2022/9141117 (PMC9169203; doi:10.1155/2022/9141117)
Supplement: Supplementary 2 — Table S2: results of GO and KEGG analysis. [file 9141117.f2.pdf]

Table S2

ONTOLOG	ID	Description	BgRatio
BP	GO:00327	positive regulation of interleukin-1 beta production	53/18866
BP	GO:00327	positive regulation of interleukin-1 production	60/18866
BP	GO:00326	regulation of interleukin-1 beta production	97/18866
BP	GO:00326	interleukin-1 beta production	106/18866
BP	GO:00326	regulation of interleukin-1 production	111/18866
BP	GO:00702	pyroptosis	12/18866
BP	GO:00326	interleukin-1 production	121/18866
BP	GO:20010	positive regulation of cysteine-type endopeptidase activity	151/18866
BP	GO:00109	positive regulation of endopeptidase activity	181/18866
BP	GO:00109	positive regulation of peptidase activity	201/18866
BP	GO:00018	positive regulation of cytokine production	447/18866
BP	GO:20001	regulation of cysteine-type endopeptidase activity	239/18866
BP	GO:00432	positive regulation of cysteine-type endopeptidase activity involved in a	134/18866
BP	GO:00510	positive regulation of NF-kappaB transcription factor activity	159/18866
BP	GO:00096	response to virus	349/18866
BP	GO:00458	positive regulation of proteolysis	370/18866
BP	GO:00525	regulation of endopeptidase activity	434/18866
BP	GO:00432	regulation of cysteine-type endopeptidase activity involved in apoptotic	215/18866
BP	GO:00069	activation of cysteine-type endopeptidase activity involved in apoptotic	86/18866
BP	GO:00525	regulation of peptidase activity	466/18866
BP	GO:00510	positive regulation of DNA-binding transcription factor activity	270/18866
BP	GO:00713	cellular response to tumor necrosis factor	297/18866
BP	GO:00346	response to tumor necrosis factor	320/18866
BP	GO:00095	detection of biotic stimulus	36/18866
BP	GO:00427	defense response to bacterium	348/18866
BP	GO:00332	tumor necrosis factor-mediated signaling pathway	173/18866
BP	GO:00713	cellular response to interferon-gamma	182/18866
BP	GO:00343	response to interferon-gamma	202/18866
BP	GO:00510	regulation of DNA-binding transcription factor activity	455/18866
BP	GO:00702	necrotic cell death	64/18866
BP	GO:00712	cellular response to biotic stimulus	246/18866
BP	GO:00516	defense response to virus	258/18866
BP	GO:00603	interferon-gamma-mediated signaling pathway	91/18866
BP	GO:00508	defense response to Gram-positive bacterium	99/18866
BP	GO:19037	negative regulation of production of miRNAs involved in gene silencing	14/18866
BP	GO:00160	detection of bacterium	17/18866
BP	GO:00445	NLRP3 inflammasome complex assembly	17/18866
BP	GO:00513	regulation of oxidoreductase activity	117/18866
BP	GO:19012	regulation of NIK/NF-kappaB signaling	119/18866
BP	GO:00985	detection of other organism	19/18866
BP	GO:00609	negative regulation of gene silencing by miRNA	20/18866
BP	GO:00510	positive regulation of nitric-oxide synthase activity	22/18866
BP	GO:00313	positive regulation of defense response	385/18866
BP	GO:00354	cellular response to interferon-beta	23/18866
BP	GO:00601	negative regulation of posttranscriptional gene silencing	23/18866
BP	GO:00609	negative regulation of gene silencing by RNA	23/18866
BP	GO:19037	regulation of production of miRNAs involved in gene silencing by miRN	23/18866
BP	GO:00985	detection of external biotic stimulus	24/18866
BP	GO:00709	regulation of production of small RNA involved in gene silencing by RN	25/18866
BP	GO:00028	regulation of response to biotic stimulus	409/18866

BP	GO:00096!	response to salt stress	27/18866
BP	GO:00028:	regulation of adaptive immune response	168/1886€
BP	GO:00327:	positive regulation of monooxygenase activity	31/18866
BP	GO:00354!	response to interferon-beta	32/18866
BP	GO:20012:	positive regulation of apoptotic signaling pathway	183/1886€
BP	GO:00380(	NIK/NF-kappaB signaling	184/1886€
BP	GO:00431:	positive regulation of I-kappaB kinase/NF-kappaB signaling	188/1886€
BP	GO:00609(	negative regulation of gene silencing	37/18866
BP	GO:19028!	positive regulation of pri-miRNA transcription by RNA polymerase II	40/18866
BP	GO:00712:	cellular response to lipopolysaccharide	208/1886€
BP	GO:00712:	cellular response to molecule of bacterial origin	222/1886€
BP	GO:00702(	necroptotic process	45/18866
BP	GO:00026:	regulation of acute inflammatory response	49/18866
BP	GO:00351!	production of miRNAs involved in gene silencing by miRNA	49/18866
BP	GO:00973(	programmed necrotic cell death	50/18866
BP	GO:19028!	regulation of pri-miRNA transcription by RNA polymerase II	51/18866
BP	GO:00509!	regulation of nitric-oxide synthase activity	52/18866
BP	GO:00616:	pri-miRNA transcription by RNA polymerase II	53/18866
BP	GO:00028:	positive regulation of response to biotic stimulus	251/1886€
BP	GO:00431:	regulation of I-kappaB kinase/NF-kappaB signaling	252/1886€
BP	GO:00310!	dsRNA processing	54/18866
BP	GO:00709:	production of small RNA involved in gene silencing by RNA	54/18866
BP	GO:00302:	T cell differentiation	253/1886€
BP	GO:00513!	positive regulation of oxidoreductase activity	55/18866
BP	GO:00435:	positive regulation of neuron apoptotic process	59/18866
BP	GO:00619:	selective autophagy	61/18866
BP	GO:00108(	regulation of tumor necrosis factor-mediated signaling pathway	62/18866
BP	GO:20003!	regulation of endothelial cell apoptotic process	62/18866
BP	GO:20012‹	positive regulation of intrinsic apoptotic signaling pathway	62/18866
BP	GO:00072‹	I-kappaB kinase/NF-kappaB signaling	286/1886€
BP	GO:00971!	intrinsic apoptotic signaling pathway	290/1886€
BP	GO:19021(	regulation of leukocyte differentiation	290/1886€
BP	GO:00327(	regulation of monooxygenase activity	66/18866
BP	GO:00725:	endothelial cell apoptotic process	68/18866
BP	GO:00356!	cellular response to drug	71/18866
BP	GO:00450{	regulation of innate immune response	307/1886€
BP	GO:00506(	cytokine secretion	72/18866
BP	GO:00714!	cellular response to external stimulus	326/1886€
BP	GO:00712(	cellular response to mechanical stimulus	79/18866
BP	GO:19030:	regulation of leukocyte cell-cell adhesion	329/1886€
BP	GO:00712:	cellular response to abiotic stimulus	331/1886€
BP	GO:01040(	cellular response to environmental stimulus	331/1886€
BP	GO:19012:	positive regulation of NIK/NF-kappaB signaling	80/18866
BP	GO:00324!	response to lipopolysaccharide	334/1886€
BP	GO:00027:	regulation of cytokine production involved in immune response	83/18866
BP	GO:00069:	response to osmotic stress	85/18866
BP	GO:00327!	positive regulation of interleukin-6 production	88/18866
BP	GO:00022:	response to molecule of bacterial origin	356/1886€
BP	GO:00071!	leukocyte cell-cell adhesion	364/1886€
BP	GO:00320{	negative regulation of NF-kappaB transcription factor activity	93/18866
BP	GO:00300!	lymphocyte differentiation	368/1886€

BP	GO:19040:	regulation of epithelial cell apoptotic process	94/18866
BP	GO:00603:	type I interferon signaling pathway	95/18866
BP	GO:00713!	cellular response to type I interferon	95/18866
BP	GO:00971!	execution phase of apoptosis	95/18866
BP	GO:00466:	regulation of alpha-beta T cell activation	97/18866
BP	GO:19012:	positive regulation of neuron death	97/18866
BP	GO:00343‹	response to type I interferon	99/18866
BP	GO:00326:	interleukin-8 production	101/1886€
BP	GO:00086:	intrinsic apoptotic signaling pathway in response to DNA damage	103/1886€
BP	GO:00023(	cytokine production involved in immune response	104/1886€
BP	GO:00028:	positive regulation of adaptive immune response based on somatic reco	105/1886€
BP	GO:00027(	positive regulation of lymphocyte mediated immunity	106/1886€
BP	GO:00466:	alpha-beta T cell differentiation	106/1886€
BP	GO:20003:	positive regulation of reactive oxygen species metabolic process	106/1886€
BP	GO:00022:	T cell activation involved in immune response	109/1886€
BP	GO:00028:	positive regulation of adaptive immune response	110/1886€
BP	GO:20012:	regulation of apoptotic signaling pathway	413/1886€
BP	GO:00025:	acute inflammatory response	111/1886€
BP	GO:00507:	regulation of inflammatory response	425/1886€
BP	GO:00609(	regulation of gene silencing by miRNA	116/1886€
BP	GO:19040:	epithelial cell apoptotic process	117/1886€
BP	GO:00601‹	regulation of posttranscriptional gene silencing	120/1886€
BP	GO:00609(	regulation of gene silencing by RNA	120/1886€
BP	GO:00224(	regulation of cell-cell adhesion	439/1886€
BP	GO:00510!	negative regulation of DNA metabolic process	127/1886€
BP	GO:19054:	positive regulation of protein localization to membrane	127/1886€
BP	GO:00026!	regulation of immune effector process	470/1886€
BP	GO:00513‹	negative regulation of hydrolase activity	473/1886€
BP	GO:00027(	positive regulation of leukocyte mediated immunity	138/1886€
BP	GO:00463:	positive regulation of JNK cascade	138/1886€
BP	GO:00421:	T cell activation	483/1886€
BP	GO:00609(	regulation of gene silencing	140/1886€
BP	GO:00022:	activation of innate immune response	144/1886€
BP	GO:00027(	regulation of production of molecular mediator of immune response	144/1886€
BP	GO:19037(	regulation of hemopoiesis	498/1886€
BP	GO:00466:	alpha-beta T cell activation	146/1886€
BP	GO:00455:	regulation of T cell differentiation	150/1886€
BP	GO:00028:	regulation of adaptive immune response based on somatic recombinati	153/1886€
BP	GO:00027(	regulation of lymphocyte mediated immunity	154/1886€
BP	GO:00001:	activation of MAPK activity	156/1886€
BP	GO:00326:	regulation of interleukin-6 production	159/1886€
BP	GO:19021(	positive regulation of leukocyte differentiation	159/1886€
BP	GO:00435:	regulation of blood vessel endothelial cell migration	164/1886€
BP	GO:20012‹	regulation of intrinsic apoptotic signaling pathway	166/1886€
BP	GO:00326:	interleukin-6 production	170/1886€
BP	GO:00328:	positive regulation of stress-activated MAPK cascade	170/1886€
BP	GO:00703(	positive regulation of stress-activated protein kinase signaling cascade	172/1886€
CC	GO:00617(	inflammasome complex	17/19559
MF	GO:00165(	peptidase activator activity	40/18352
MF	GO:00507(	CARD domain binding	16/18352
MF	GO:00086!	cysteine-type endopeptidase activator activity involved in apoptotic pro	17/18352

MF	GO:00428:	peptidoglycan binding	18/18352
MF	GO:00165(	peptidase activator activity involved in apoptotic process	20/18352
MF	GO:00020:	protease binding	137/18352
MF	GO:00430:	cysteine-type endopeptidase regulator activity involved in apoptotic pr	43/18352
MF	GO:00611:	peptidase regulator activity	229/18352
MF	GO:00309:	receptor tyrosine kinase binding	72/18352
MF	GO:00055:	phosphatidylinositol-4,5-bisphosphate binding	82/18352
MF	GO:19907:	protein tyrosine kinase binding	98/18352
MF	GO:19029:	phosphatidylinositol bisphosphate binding	119/18352
MF	GO:19019:	phosphatidylinositol phosphate binding	179/18352
MF	GO:00010:	TFIID-class transcription factor complex binding	10/18352
MF	GO:00971:	cysteine-type endopeptidase activity involved in apoptotic signaling pat	10/18352
MF	GO:19016:	cardiolipin binding	11/18352
MF	GO:00051(	neurotrophin receptor binding	12/18352
MF	GO:00897:	caspase binding	12/18352
MF	GO:00971:	cysteine-type endopeptidase activity involved in apoptotic process	15/18352
MF	GO:19016:	phosphatidylglycerol binding	15/18352
MF	GO:00055:	glycosaminoglycan binding	232/18352
MF	GO:00010:	RNA polymerase II general transcription initiation factor binding	19/18352
MF	GO:00350:	phosphatidylinositol binding	262/18352
MF	GO:00051:	cytokine receptor binding	271/18352
MF	GO:00381:	pattern recognition receptor activity	23/18352
MF	GO:00044:	phosphatidylinositol phospholipase C activity	26/18352
MF	GO:00046:	phospholipase C activity	28/18352
MF	GO:00350:	histone acetyltransferase binding	28/18352

pvalue	p.adjust	qvalue	geneID	Count
6.20E-13	7.52E-10	3.41E-10	NLRC4/AIN	6
1.35E-12	8.18E-10	3.71E-10	NLRC4/AIN	6
2.63E-11	1.06E-08	4.83E-09	NLRC4/AIN	6
4.53E-11	1.35E-08	6.14E-09	NLRC4/AIN	6
6.00E-11	1.35E-08	6.14E-09	NLRC4/AIN	6
6.69E-11	1.35E-08	6.14E-09	NLRC4/AIN	4
1.02E-10	1.76E-08	7.99E-09	NLRC4/AIN	6
3.90E-10	5.91E-08	2.68E-08	NLRC4/AIN	6
1.16E-09	1.57E-07	7.12E-08	NLRC4/AIN	6
2.19E-09	2.65E-07	1.20E-07	NLRC4/AIN	6
6.07E-09	6.25E-07	2.84E-07	NLRC4/AIN	7
6.19E-09	6.25E-07	2.84E-07	NLRC4/AIN	6
2.06E-08	1.92E-06	8.73E-07	NLRC4/TNI	5
4.87E-08	4.21E-06	1.91E-06	NLRC4/AIN	5
5.91E-08	4.77E-06	2.17E-06	AIM2/TNF/	6
8.35E-08	6.32E-06	2.87E-06	NLRC4/AIN	6
2.14E-07	1.48E-05	6.71E-06	NLRC4/AIN	6
2.19E-07	1.48E-05	6.71E-06	NLRC4/TNI	5
2.79E-07	1.78E-05	8.08E-06	NLRC4/TNI	4
3.26E-07	1.97E-05	8.97E-06	NLRC4/AIN	6
6.78E-07	3.91E-05	1.78E-05	NLRC4/AIN	5
1.09E-06	5.98E-05	2.72E-05	AIM2/TNF/	5
1.57E-06	8.25E-05	3.75E-05	AIM2/TNF/	5
1.80E-06	9.10E-05	4.13E-05	NLRC4/NO	3
2.36E-06	0.000115	5.21E-05	NLRC4/TNI	5
4.58E-06	0.000213	9.69E-05	AIM2/TNF/	4
5.60E-06	0.000251	0.000114	CASP1/IRF	4
8.46E-06	0.000366	0.000166	CASP1/IRF	4
8.75E-06	0.000366	0.000166	NLRC4/AIN	5
1.04E-05	0.00042	0.000191	TNF/GSDN	3
1.84E-05	0.000719	0.000327	TNF/CASP1	4
2.22E-05	0.00084	0.000381	AIM2/NLRI	4
3.00E-05	0.001101	0.0005	IRF1/IRF2/	3
3.86E-05	0.001375	0.000624	TNF/NOD1	3
3.97E-05	0.001375	0.000624	TNF/TP53	2
5.93E-05	0.001941	0.000882	NLRC4/NO	2
5.93E-05	0.001941	0.000882	AIM2/NLRI	2
6.35E-05	0.002027	0.000921	TNF/NOD1	3
6.68E-05	0.002077	0.000943	TNF/NOD1	3
7.45E-05	0.002256	0.001025	NLRC4/NO	2
8.27E-05	0.002445	0.00111	TNF/TP53	2
0.0001	0.002836	0.001288	TNF/NOD1	2
0.000106	0.002836	0.001288	NLRC4/AIN	4
0.00011	0.002836	0.001288	AIM2/IRF1	2
0.00011	0.002836	0.001288	TNF/TP53	2
0.00011	0.002836	0.001288	TNF/TP53	2
0.00011	0.002836	0.001288	TNF/TP53	2
0.00012	0.003029	0.001376	NLRC4/NO	2
0.00013	0.003224	0.001464	TNF/TP53	2
0.000133	0.00323	0.001467	NLRC4/AIN	4

0.000152	0.003621	0.001645	TNF/TP53	2
0.000186	0.00433	0.001967	TNF/NLRP3	3
0.000202	0.004609	0.002093	TNF/NOD1	2
0.000215	0.004823	0.002191	AIM2/IRF1	2
0.000239	0.005257	0.002388	TNF/GSDM	3
0.000243	0.005257	0.002388	TNF/NOD1	3
0.000259	0.005502	0.002499	TNF/CASP1	3
0.000288	0.006018	0.002733	TNF/TP53	2
0.000337	0.00692	0.003143	TNF/TP53	2
0.000348	0.007034	0.003195	TNF/CASP1	3
0.000421	0.008342	0.003789	TNF/CASP1	3
0.000427	0.008342	0.003789	TNF/TP53	2
0.000506	0.009585	0.004354	TNF/NLRP3	2
0.000506	0.009585	0.004354	TNF/TP53	2
0.000527	0.009827	0.004464	TNF/TP53	2
0.000548	0.010069	0.004574	TNF/TP53	2
0.00057	0.010245	0.004654	TNF/NOD1	2
0.000592	0.010245	0.004654	TNF/TP53	2
0.000603	0.010245	0.004654	NLRC4/AIM	3
0.00061	0.010245	0.004654	TNF/CASP1	3
0.000615	0.010245	0.004654	TNF/TP53	2
0.000615	0.010245	0.004654	TNF/TP53	2
0.000617	0.010245	0.004654	NLRP3/IRF	3
0.000638	0.010443	0.004744	TNF/NOD1	2
0.000734	0.011854	0.005384	TNF/TP53	2
0.000784	0.012423	0.005643	NOD1/TP5	2
0.00081	0.012423	0.005643	TNF/CASP1	2
0.00081	0.012423	0.005643	TNF/PLCG1	2
0.00081	0.012423	0.005643	GSDME/TP	2
0.000881	0.013346	0.006062	TNF/CASP1	3
0.000917	0.013392	0.006083	TNF/GSDM	3
0.000917	0.013392	0.006083	TNF/NLRP3	3
0.000917	0.013392	0.006083	TNF/NOD1	2
0.000973	0.014042	0.006378	TNF/PLCG1	2
0.00106	0.01512	0.006868	AIM2/TP53	2
0.001081	0.015189	0.006899	NLRC4/AIM	3
0.00109	0.015189	0.006899	TNF/NLRP3	2
0.001286	0.017512	0.007954	CASP1/IRF	3
0.001311	0.017512	0.007954	CASP1/IRF	2
0.00132	0.017512	0.007954	TNF/NLRP3	3
0.001343	0.017512	0.007954	CASP1/IRF	3
0.001343	0.017512	0.007954	CASP1/IRF	3
0.001344	0.017512	0.007954	TNF/NOD1	2
0.001379	0.017774	0.008073	TNF/CASP1	3
0.001445	0.01844	0.008376	TNF/NLRP3	2
0.001515	0.019128	0.008689	TNF/TP53	2
0.001623	0.020276	0.00921	TNF/NOD1	2
0.001656	0.020475	0.0093	TNF/CASP1	3
0.001764	0.0216	0.009811	TNF/NLRP3	3
0.00181	0.021788	0.009897	AIM2/NLR	2
0.00182	0.021788	0.009897	NLRP3/IRF	3

0.001849	0.021788	0.009897	TNF/PLCG1	2
0.001888	0.021788	0.009897	IRF1/IRF2	2
0.001888	0.021788	0.009897	IRF1/IRF2	2
0.001888	0.021788	0.009897	CASP1/TP5	2
0.001967	0.022278	0.01012	NLRP3/IRF	2
0.001967	0.022278	0.01012	TNF/TP53	2
0.002048	0.022979	0.010438	IRF1/IRF2	2
0.00213	0.023683	0.010758	TNF/NOD1	2
0.002214	0.024393	0.01108	TNF/TP53	2
0.002256	0.024637	0.011191	TNF/NLRP3	2
0.002299	0.024689	0.011214	TNF/NLRP3	2
0.002343	0.024689	0.011214	TNF/NLRP3	2
0.002343	0.024689	0.011214	NLRP3/IRF	2
0.002343	0.024689	0.011214	TNF/TP53	2
0.002475	0.025858	0.011745	NLRP3/TP5	2
0.00252	0.025982	0.011802	TNF/NLRP3	2
0.00253	0.025982	0.011802	TNF/GSDM	3
0.002565	0.026124	0.011866	TNF/NLRP3	2
0.002744	0.027714	0.012588	TNF/CASP1	3
0.002797	0.028015	0.012725	TNF/TP53	2
0.002844	0.028258	0.012835	TNF/PLCG1	2
0.002989	0.029165	0.013247	TNF/TP53	2
0.002989	0.029165	0.013247	TNF/TP53	2
0.003008	0.029165	0.013247	TNF/NLRP3	3
0.003341	0.031881	0.014481	GZMA/TP5	2
0.003341	0.031881	0.014481	TNF/TP53	2
0.003647	0.034535	0.015687	AIM2/TNF/	3
0.003713	0.034887	0.015847	TNF/GZMA	3
0.00393	0.036163	0.016426	TNF/NLRP3	2
0.00393	0.036163	0.016426	TNF/NOD1	2
0.003939	0.036163	0.016426	NLRP3/IRF	3
0.004042	0.036835	0.016732	TNF/TP53	2
0.004271	0.038248	0.017374	NLRC4/AIM	2
0.004271	0.038248	0.017374	TNF/NLRP3	2
0.004292	0.038248	0.017374	TNF/NLRP3	3
0.004387	0.038812	0.017629	NLRP3/IRF	2
0.004624	0.040615	0.018448	NLRP3/IRF	2
0.004806	0.041908	0.019036	TNF/NLRP3	2
0.004868	0.04214	0.019141	TNF/NLRP3	2
0.004991	0.042905	0.019489	TNF/NOD1	2
0.00518	0.043902	0.019942	TNF/NOD1	2
0.00518	0.043902	0.019942	TNF/NLRP3	2
0.005501	0.046301	0.021031	TNF/PLCG1	2
0.005632	0.047077	0.021384	GSDME/TP	2
0.005899	0.048633	0.022091	TNF/NOD1	2
0.005899	0.048633	0.022091	TNF/NOD1	2
0.006034	0.049413	0.022445	TNF/NOD1	2
2.78E-10	1.11E-08	9.36E-09	NLRC4/AIM	4
2.70E-06	0.000189	6.83E-05	NLRC4/CAS	3
5.53E-05	0.001223	0.000441	CASP1/NO	2
6.26E-05	0.001223	0.000441	CASP1/NO	2

7.04E-05	0.001223	0.000441	NOD1/NLR	2
8.74E-05	0.001223	0.000441	CASP1/NO	2
0.00011	0.001286	0.000464	NLRC4/TNI	3
0.000411	0.004115	0.001485	CASP1/NO	2
0.0005	0.004375	0.001579	NLRC4/CAS	3
0.001151	0.008955	0.003232	PLCG1/TP5	2
0.00149	0.01043	0.003764	GSDMC/GS	2
0.002119	0.013483	0.004866	PLCG1/TP5	2
0.003104	0.018105	0.006534	GSDMC/GS	2
0.006876	0.032939	0.011888	GSDMC/GS	2
0.007063	0.032939	0.011888	TP53	1
0.007063	0.032939	0.011888	CASP1	1
0.007767	0.032939	0.011888	GSDME	1
0.00847	0.032939	0.011888	PLCG1	1
0.00847	0.032939	0.011888	NLRC4	1
0.010577	0.03702	0.01336	CASP1	1
0.010577	0.03702	0.01336	GSDME	1
0.011323	0.037744	0.013622	NOD1/NLR	2
0.01338	0.042573	0.015365	TP53	1
0.014276	0.04345	0.015681	GSDMC/GS	2
0.015221	0.044395	0.016022	TNF/PLCG1	2
0.016176	0.045292	0.016346	NOD1	1
0.018268	0.04915	0.017738	PLCG1	1
0.01966	0.04915	0.017738	PLCG1	1
0.01966	0.04915	0.017738	TP53	1

ID	Description	BgRatio	pvalue	p.adjust	qvalue
hsa05131	Shigellosis	247/8111	2.48E-09	3.15E-07	2.06E-07
hsa05133	Pertussis	76/8111	1.54E-08	9.75E-07	6.39E-07
hsa04621	NOD-like receptor signaling pathway	185/8111	2.53E-08	1.07E-06	7.01E-07
hsa05135	Yersinia infection	137/8111	3.01E-07	9.55E-06	6.26E-06
hsa05417	Lipid and atherosclerosis	215/8111	2.83E-06	7.18E-05	4.70E-05
hsa04625	C-type lectin receptor signaling pathway	104/8111	5.05E-06	0.000106	6.91E-05
hsa05132	Salmonella infection	249/8111	5.82E-06	0.000106	6.91E-05
hsa05134	Legionellosis	57/8111	3.81E-05	0.000605	0.000396
hsa05171	Coronavirus disease - COVID-19	232/8111	0.00012	0.001688	0.001105
hsa04217	Necroptosis	159/8111	0.000802	0.010181	0.006667
hsa05164	Influenza A	172/8111	0.001008	0.011634	0.007618
hsa05130	Pathogenic Escherichia coli infection	197/8111	0.001493	0.015799	0.010345
hsa05205	Proteoglycans in cancer	205/8111	0.001674	0.016358	0.010711
hsa04623	Cytosolic DNA-sensing pathway	63/8111	0.002567	0.023286	0.015247
hsa04664	Fc epsilon RI signaling pathway	68/8111	0.002984	0.024949	0.016336
hsa05120	Epithelial cell signaling in Helicobacter pylori	70/8111	0.00316	0.024949	0.016336
hsa05223	Non-small cell lung cancer	72/8111	0.00334	0.024949	0.016336
hsa05214	Glioma	75/8111	0.003619	0.025531	0.016718
hsa04933	AGE-RAGE signaling pathway in diabetic com	100/8111	0.006349	0.039556	0.025901
hsa05165	Human papillomavirus infection	331/8111	0.006525	0.039556	0.025901
hsa04064	NF-kappa B signaling pathway	104/8111	0.006852	0.039556	0.025901
hsa04660	T cell receptor signaling pathway	104/8111	0.006852	0.039556	0.025901
hsa04668	TNF signaling pathway	112/8111	0.007911	0.043202	0.028288
hsa05014	Amyotrophic lateral sclerosis	365/8111	0.008562	0.043202	0.028288
hsa04071	Sphingolipid signaling pathway	119/8111	0.008894	0.043202	0.028288
hsa04722	Neurotrophin signaling pathway	119/8111	0.008894	0.043202	0.028288
hsa04919	Thyroid hormone signaling pathway	121/8111	0.009185	0.043202	0.028288
hsa04650	Natural killer cell mediated cytotoxicity	131/8111	0.010702	0.04854	0.031783

geneID	Count
58484/712	7
7124/834/	5
58484/944	6
58484/712	5
7124/834/	5
7124/834/	4
58484/712	5
58484/712	3
7124/834/	4
7124/834/	3
7124/834/	3
7124/834/	3
7124/5335	3
9447/834	2
7124/5335	2
10392/533	2
5335/7157	2
5335/7157	2
7124/5335	2
7124/3659	3
7124/5335	2
7124/5335	2
7124/3659	2
7124/834/	3
7124/7157	2
5335/7157	2
5335/7157	2
7124/5335	2
