# Supplementary material for: Development of a Prognostic Model Based on Pyroptosis-Related Genes in Pancreatic Adenocarcinoma
Source: Dis Markers. 2022 May 29;2022:9141117. doi: 10.1155/2022/9141117 (PMC9169203; doi:10.1155/2022/9141117)
Supplement: Supplementary 3 — Table S3: the result of GSVA analysis. [file 9141117.f3.pdf]

id	TCGA-2J-A	TCGA-2J-A	TCGA-2J-A	TCGA-2J-A	TCGA-2J-A	TCGA-2J-A	TCGA-2J-A	TCGA-2J-A
KEGG_N_G	-0.41825	0.066991	-0.05807	0.044076	-0.21309	-0.3658	0.04171	0.166934
KEGG_OTH	-0.50097	0.512682	0.458201	0.288151	-0.17694	-0.4569	0.414413	-0.48744
KEGG_O_G	0.228532	0.387884	-0.15809	0.142684	-0.25665	-0.24674	-0.00374	0.111466
KEGG_GLY	0.024687	0.280094	0.118737	0.351831	-0.01911	-0.25275	0.412447	0.112179
KEGG_GLY	-0.02715	0.213182	-0.41159	0.467832	-0.233	-0.03896	-0.01181	-0.12398
KEGG_GLY	0.074131	-0.04523	0.141832	0.267566	-0.23885	0.043897	0.087282	-0.08279
KEGG_GLY	0.190907	0.185122	-0.52041	0.224557	0.003006	0.12297	-0.17346	0.398017
KEGG_GLY	0.10159	0.084679	0.055615	0.243305	0.059791	0.173034	-0.0146	0.069393
KEGG_ETH	0.148081	0.330013	-0.02406	0.123966	-0.03483	0.301525	0.138972	0.170214
KEGG_ARA	0.287546	0.217429	0.150702	-0.11617	0.004927	0.190375	0.281168	-0.14315
KEGG_LIN	0.130591	0.366047	0.081216	0.065041	-0.05373	0.333293	-0.04442	-0.01373
KEGG_ALP	-0.18018	0.410083	-0.0393	0.242592	0.087919	0.134458	-0.07189	0.140841
KEGG_SPH	0.208532	0.316892	0.335973	0.205313	-0.45059	-0.2003	0.014225	-0.20892
KEGG_GLY	0.29341	0.347163	-0.05663	0.1004	-0.09971	0.278517	-0.03401	-0.14667
KEGG_GLY	0.223796	0.38775	-0.21037	-0.00309	-0.45606	-0.44694	0.437836	0.130238
KEGG_GLY	-0.38254	-0.16024	-0.47359	0.040036	-0.07122	-0.4699	-0.03783	-0.36627
KEGG_RIB	-0.45972	-0.43783	0.14364	0.275215	-0.18851	0.063923	-0.17844	-0.04853
KEGG_NIC	-0.21682	0.217811	0.044992	0.17175	-0.26481	-0.24065	-0.03115	0.116895
KEGG_PAN	-0.07864	0.027855	0.381037	-0.40194	-0.2697	-0.07595	-0.04799	-0.17183
KEGG_AMI	0.341658	-0.51466	-0.00473	0.478942	0.255934	0.016666	-0.55912	0.136012
KEGG_BAS	-0.18459	-0.41581	-0.10441	-0.09481	0.006632	-0.17783	-0.52184	0.129659
KEGG_NON	-0.21896	0.002962	0.333844	-0.11149	0.345522	-0.39022	-0.14091	-0.01663
KEGG_SNA	0.121481	0.098962	-0.08621	0.293295	-0.03809	0.040361	-0.35609	0.008213
KEGG_LYS	-0.03042	0.179398	0.290814	0.133574	-0.3283	-0.31415	0.135144	-0.28527
KEGG_CAR	-0.00183	0.109816	0.12031	0.016873	-0.32822	0.337207	-0.38782	0.02838
KEGG_REN	0.099416	-0.23258	-0.16737	-0.23574	-0.36106	-0.28846	0.0877	-0.09152
KEGG_CIR	-0.21183	0.187498	-0.32518	-0.06462	0.1177	0.184333	0.441118	0.474234
KEGG_TAS	0.16926	-0.00904	0.401801	-0.07192	0.157257	0.215264	0.250733	-0.38932
KEGG_PRO	0.20575	0.351109	-0.04824	0.417107	-0.57114	0.449002	0.446611	-0.41275
KEGG_PAT	-0.06167	0.030585	0.425583	-0.04132	-0.17844	-0.24406	0.19879	-0.01666
KEGG_SYS	0.063668	0.349497	0.5759	-0.33737	-0.23296	-0.42108	0.467276	-0.34269
KEGG_PRI	0.188498	0.218798	0.416194	-0.21048	0.275571	-0.39103	0.443292	-0.04318
KEGG_HYP	-0.05687	-0.12084	0.086649	0.069494	-0.1981	-0.29318	0.046016	0.140869
KEGG_ARR	-0.07883	-0.1244	0.091607	-0.02052	-0.10831	-0.42784	0.151585	0.192721
KEGG_DIL	-0.06037	-0.05383	0.107842	0.042364	-0.23784	-0.327	0.211507	0.091524
KEGG_GLY	0.072288	0.513576	0.079119	0.097064	0.00895	0.171221	-0.06975	0.230904
KEGG_CIT	-0.40437	0.191026	0.057283	-0.40968	-0.52359	0.140989	0.014579	-0.08116
KEGG_PEN	-0.03605	0.222059	-0.03147	0.272859	0.165461	-0.29945	-0.06993	0.069244
KEGG_PEN	0.4222	0.173631	-0.28835	0.027994	-0.25157	-0.12443	-0.02663	0.032852
KEGG_FRU	0.041143	0.417786	-0.08866	0.238748	-0.03452	0.178922	-0.20457	0.025966
KEGG_GAL	-0.03738	0.460453	-0.32193	0.370872	-0.0871	-0.20117	0.219795	0.216549
KEGG_ASC	0.431533	0.31211	-0.1172	0.170229	-0.2452	0.20742	0.282316	-0.03019
KEGG_FAT	0.388969	0.140446	0.097069	0.098005	-0.45574	0.265654	0.333772	-0.26656
KEGG_STE	0.360517	0.686752	0.682824	0.645561	-0.42871	0.387066	0.325128	-0.59445
KEGG_PRI	-0.04249	0.061569	-0.1257	0.077517	-0.46029	0.23735	0.394605	-0.35699
KEGG_STE	0.418103	0.166733	0.140877	0.185459	-0.35065	0.185497	0.349002	0.138468
KEGG_OXI	-0.02219	-0.16335	-0.01312	0.063074	-0.12191	0.516671	-0.44829	-0.22076
KEGG_PUR	0.030817	-0.05265	-0.05892	-0.03451	-0.10433	0.174969	-0.13116	0.002167
KEGG_PYR	-0.08554	-0.253	-0.01986	-0.09739	0.012828	0.203688	-0.27889	0.147499

KEGG_ALA	-0.14069	0.120813	-0.00916	0.04372	-0.26631	0.212822	0.165408	-0.01413
KEGG_GLY	0.119408	0.216356	-0.11392	-0.07711	-0.22069	-0.00235	0.366273	-0.47826
KEGG_CYS	-0.21916	0.234927	0.265067	0.143234	-0.33544	0.03693	0.401935	-0.20143
KEGG_VAL	0.181498	0.094109	-0.19806	0.009964	-0.37494	0.316192	0.19202	-0.27347
KEGG_LYSI	-0.15264	-0.25392	0.21323	0.127204	-0.0387	0.082528	0.143094	-0.26189
KEGG_ARG	0.213883	0.142079	-0.12644	0.19505	-0.32315	0.332257	-0.05095	-0.255
KEGG_HIST	0.483392	0.095231	0.115738	-0.03576	-0.06515	0.325321	-0.05202	0.292219
KEGG_TYR	0.383079	0.254506	0.021766	-0.14651	0.024242	0.176755	0.209241	0.19269
KEGG_PHE	0.32421	0.35811	0.138058	0.024815	0.016854	0.369997	0.300335	0.11003
KEGG_TRY	0.178132	0.37535	0.221686	-0.03586	-0.24687	0.029854	0.235022	-0.29621
KEGG_BET	0.223575	0.006617	-0.18527	-0.12948	-0.31268	0.305666	0.213561	-0.09786
KEGG_TAU	0.21926	0.474294	0.230223	-0.13686	-0.2498	0.328285	0.583175	-0.47684
KEGG_SELI	0.062247	0.225617	0.281663	0.074843	-0.11919	0.223644	0.146878	-0.32572
KEGG_GLU	0.227677	0.306547	0.420625	-0.10884	-0.29355	0.374814	0.382491	0.109829
KEGG_STA	0.075663	0.29815	-0.18987	0.060906	-0.23348	-0.20547	0.254409	0.209422
KEGG_AMI	-0.38599	0.250132	-0.27689	0.072233	-0.27705	0.008814	0.074945	0.024209
KEGG_GLY	-0.35605	-0.09037	-0.54792	-0.15284	0.494157	-0.6784	0.241596	0.287804
KEGG_GLY	-0.19021	0.258404	-0.38185	-0.14587	-0.09903	-0.4953	0.138923	0.2859
KEGG_INO	0.189901	-0.0258	0.057127	0.013398	-0.33916	-0.20239	0.178433	-0.24206
KEGG_PYR	0.004884	0.347562	-0.20919	0.110329	-0.37229	0.316128	0.12482	-0.21972
KEGG_GLY	0.065394	0.012005	-0.42519	-0.43395	-0.36018	0.376581	0.290896	-0.27523
KEGG_PRC	0.181727	0.103702	-0.17858	0.164756	-0.39782	0.214401	0.310463	-0.43355
KEGG_BUT	0.262422	0.370452	-0.06113	0.202344	-0.31699	0.391549	0.136449	-0.26791
KEGG_ONE	0.170648	0.301952	-0.17308	0.013706	0.394178	0.305165	0.017945	0.01197
KEGG_FOL	-0.01863	0.260433	0.166481	0.269435	0.030022	0.435557	-0.2525	-0.03093
KEGG_RET	0.449824	0.234052	0.014899	-0.00683	-0.27396	0.017542	0.292132	-0.153
KEGG_POF	0.454568	-0.00355	-0.07313	0.080455	-0.05587	0.043502	0.057932	-0.01224
KEGG_TER	0.178796	0.503027	0.504485	0.673671	-0.49541	0.22046	-0.16703	-0.47125
KEGG_NITI	0.310478	0.152681	-0.04735	0.265542	-0.15598	0.412162	0.040022	-0.02423
KEGG_SUL	0.456154	-0.27576	0.269517	-0.36275	-0.16361	-0.07789	-0.16687	-0.06687
KEGG_MET	0.451767	0.318966	0.280721	0.036461	-0.27944	0.29883	0.209438	0.206534
KEGG_DRU	0.483754	0.336467	0.282203	0.033616	-0.27267	0.280579	0.220028	0.175676
KEGG_DRU	0.340783	0.220677	-0.07004	0.076159	0.00035	0.162387	0.005292	0.203996
KEGG_BIO	0.235829	0.403676	-0.07156	0.465597	-0.49968	-0.05217	-0.00541	-0.08547
KEGG_ABC	0.035452	-0.11414	0.292946	-0.22	-0.3402	-0.04142	0.123411	0.20958
KEGG_RIB	-0.19106	-0.61313	-0.68257	-0.5238	0.475545	0.635216	-0.5547	-0.34573
KEGG_RNA	-0.30998	-0.35962	0.080392	-0.14797	0.336355	0.310257	-0.29152	0.153643
KEGG_RNA	-0.31272	-0.58652	-0.07953	-0.02624	0.090673	0.460316	-0.47031	-0.27318
KEGG_DNA	0.061404	-0.07653	0.262261	-0.38537	0.433805	-0.17716	-0.04796	0.498402
KEGG_SPLI	-0.34104	-0.48735	0.172983	-0.28705	0.067398	0.448565	-0.32946	0.123718
KEGG_PRC	-0.28593	-0.33094	0.220163	-0.17653	0.213692	0.25245	-0.17756	0.494699
KEGG_PRC	-0.71939	-0.35793	-0.60074	-0.54892	-0.53123	-0.02673	-0.32506	0.182848
KEGG_PPA	-0.03435	0.285641	0.06437	0.243922	-0.43113	0.216265	0.106021	-0.24298
KEGG_BAS	0.186734	-0.12002	0.320648	0.036094	0.227054	0.272163	-0.37861	0.344197
KEGG_NUC	-0.01413	-0.22374	-0.05763	-0.05293	0.43726	-0.00131	-0.34518	0.318375
KEGG_MIS	0.008373	0.062562	0.327556	-0.28443	0.365126	-0.30384	-0.17328	0.353025
KEGG_HOI	0.208663	0.176985	0.043963	-0.1221	0.601482	-0.31651	-0.5523	0.227747
KEGG_MAI	-0.05529	0.040879	-0.00101	-0.07478	-0.04743	-0.10381	0.248042	0.020463
KEGG_ERB	-0.09792	0.014914	-0.05465	0.079288	0.012173	-0.22966	0.365479	0.020502
KEGG_CAL	0.066142	-0.12252	-0.08747	-0.19344	-0.25441	-0.25999	0.176068	0.008029

KEGG_CYT	0.095622	0.258037	0.347892	-0.07913	0.049396	-0.29406	0.504471	-0.16116
KEGG_CHE	0.090835	0.121675	0.400132	-0.11615	-0.21773	-0.30075	0.438516	-0.23352
KEGG_PHO	0.198999	-0.06225	0.114383	0.103276	-0.2909	-0.13395	0.183685	-0.1692
KEGG_NEU	0.157645	-0.00268	-0.09015	-0.00652	-0.16707	-0.08715	0.335501	-0.10513
KEGG_CELI	-0.08946	0.102828	0.183184	-0.33211	0.315312	-0.03913	0.007805	0.139045
KEGG_OOC	-0.11242	0.20373	0.099045	-0.111	-0.14399	0.123823	0.058613	0.101595
KEGG_P53	0.103711	0.208436	-0.03172	-0.29602	0.000969	0.121249	0.196431	0.030851
KEGG_UBI	-0.2581	-0.23353	0.097679	-0.14077	0.079444	-0.07997	-0.14948	0.269239
KEGG_REG	-0.26448	-0.12676	-0.02666	-0.0468	-0.34086	0.003096	0.014089	-0.28416
KEGG_END	0.250712	0.113571	0.095811	0.135361	-0.06738	-0.20495	0.213831	0.007916
KEGG_PER	0.278475	0.065112	-0.2351	0.097163	-0.3158	0.201186	-0.15344	-0.04751
KEGG_MT(	-0.35111	-0.15049	-0.01843	-0.23028	0.062961	-0.15901	0.253102	0.143787
KEGG_APC	0.14713	0.280963	0.303453	0.003652	0.039524	-0.02316	0.402825	-0.03802
KEGG_VAS	-0.12278	-0.08551	0.101255	-0.10689	-0.36176	-0.08018	0.251402	-0.09883
KEGG_WN	0.071542	-0.18804	-0.14378	-0.19962	-0.0862	-0.28405	0.107029	0.081225
KEGG_DOI	0.055714	0.286514	0.257176	0.099764	0.037571	-0.0203	0.406602	-0.12672
KEGG_NOT	0.024574	-0.01508	0.279749	-0.0906	-0.02461	-0.2585	0.172623	0.23411
KEGG_HED	-0.0575	-0.27555	-0.27388	-0.19285	0.241047	-0.43022	0.179788	0.080425
KEGG_TGF	-0.08906	-0.30551	-0.05122	-0.17532	-0.30544	-0.41179	0.361778	-0.04147
KEGG_AXO	-0.0806	0.105982	0.104576	0.024982	-0.01501	-0.30053	0.248341	0.246407
KEGG_VEG	-0.19015	0.104627	0.100287	-0.05316	-0.0886	-0.21285	0.21574	0.122787
KEGG_FOC	-0.16785	-0.11456	0.223481	-0.00557	-0.12933	-0.48287	0.327201	0.285925
KEGG_ECM	-0.2385	-0.11007	0.303136	-0.02011	-0.12598	-0.56749	0.355425	0.383529
KEGG_CELI	0.032301	0.348064	0.448957	-0.27116	-0.25202	-0.36523	0.543792	-0.11511
KEGG_ADH	0.162692	-0.02762	0.166725	0.033713	-0.10759	-0.3738	0.318675	0.261842
KEGG_TIGI	0.149796	0.228344	0.234749	0.191466	-0.23958	-0.11676	0.225501	0.012588
KEGG_GAF	-0.03839	0.080376	0.143056	-0.11918	-0.3185	-0.19675	0.367368	-0.04733
KEGG_CON	0.07348	0.458994	0.39163	0.028043	-0.33105	-0.19031	0.644042	-0.23131
KEGG_ANT	0.047735	0.411153	0.514386	-0.15176	-0.19334	-0.1512	0.384406	-0.07498
KEGG_TOL	-0.08704	0.331368	0.489642	-0.01269	0.090503	-0.32328	0.397175	-0.36117
KEGG_NOI	-0.28071	0.437099	0.589222	0.099622	0.192417	-0.15106	0.382362	-0.19806
KEGG_RIG	-0.11974	0.34195	0.471368	0.214511	0.193272	0.007877	0.289042	-0.16995
KEGG_CYT	0.045176	0.029927	0.510137	0.156965	0.143804	0.268187	0.257497	-0.22965
KEGG_JAK	-0.01924	0.234778	0.23302	-0.04296	-0.03322	-0.26468	0.44354	-0.20499
KEGG_HEM	0.190069	0.318065	0.338775	-0.09167	0.182424	-0.3844	0.52504	-0.03858
KEGG_NAT	0.076042	0.306188	0.304839	-0.15585	0.04857	-0.30132	0.44042	0.108955
KEGG_T_C	0.003007	0.212661	0.235837	-0.10475	-0.03275	-0.33853	0.406015	-0.06123
KEGG_B_C	-0.02952	0.250061	0.305052	-0.09028	-0.08689	-0.29897	0.38097	-0.07736
KEGG_FC_	-0.133	0.200425	0.121579	-0.06745	-0.09286	-0.23647	0.278183	-0.08503
KEGG_FC_	0.009273	0.211763	0.310149	0.008192	-0.24964	-0.22814	0.265057	-0.101
KEGG_LEU	0.088154	0.252272	0.370718	-0.02654	-0.28442	-0.34867	0.398561	-0.13374
KEGG_INTI	0.187574	0.232538	0.394829	-0.36974	-0.00353	-0.38532	0.606839	-0.19635
KEGG_LON	-0.01833	-0.0715	0.018244	-0.29072	-0.41758	-0.00911	0.181894	0.048355
KEGG_NEU	-0.06667	-0.00242	0.099669	-0.25651	-0.17613	-0.26251	0.308049	0.010108
KEGG_LON	-0.23909	0.194921	0.050113	-0.02536	-0.20715	0.084706	0.253786	0.007011
KEGG_OLF	0.043179	0.224426	0.313896	0.15588	0.111536	0.167312	0.101012	0.090826
KEGG_REG	-0.02255	-0.08052	0.162685	-0.00938	-0.1651	-0.34125	0.281934	0.150587
KEGG_INSU	-0.15195	-0.14515	0.041718	-0.00774	-0.04995	-0.12138	0.143505	0.114021
KEGG_GNF	-0.07593	0.100021	0.09922	0.033214	-0.21032	-0.01086	0.214687	0.005181
KEGG_PRC	-0.20371	0.216415	0.147683	-0.19075	0.042128	-0.13131	0.191327	-0.00413

KEGG_MEI	0.057128	-0.17824	-0.05305	-0.31422	-0.21143	-0.24633	0.155966	-0.01005
KEGG_ADI	-0.11364	0.20319	0.223569	0.280391	-0.05221	-0.02445	0.138892	-0.08684
KEGG_TYP	0.079342	0.253523	0.071087	0.218928	-0.2443	-0.00206	0.383994	-0.08725
KEGG_TYP	0.331221	0.525275	0.562079	-0.27409	-0.0402	0.005086	0.462301	-0.26626
KEGG_MAT	0.441551	0.619449	0.585363	0.462635	-0.54947	0.844235	0.588877	-0.62388
KEGG_ALD	0.239129	-0.02055	0.025221	0.073878	-0.22261	-0.02104	0.48351	-0.12464
KEGG_VAS	0.147514	0.092323	0.279686	-0.10841	-0.41901	0.139563	0.259084	-0.25131
KEGG_ALZ	-0.07333	-0.157	0.076322	-0.00803	-0.29385	0.370506	-0.35813	-0.08053
KEGG_PAR	-0.08076	-0.14249	0.051778	-0.11479	-0.19829	0.482821	-0.39575	-0.18177
KEGG_AMY	-0.01045	0.075523	0.158719	0.010685	-0.26608	0.24471	0.107634	-0.23383
KEGG_HUN	-0.08429	-0.16321	-0.00891	-0.12228	-0.22124	0.367399	-0.34009	-0.06923
KEGG_PRI(	-0.07775	0.166963	0.368455	-0.20584	0.009101	-0.00206	0.516393	-0.37174
KEGG_VIBI	-0.12435	0.138888	0.127265	-0.01087	-0.23101	0.095306	0.003989	-0.2475
KEGG_EPIT	-0.09245	0.125549	0.287815	0.128205	0.019893	-0.08359	0.160514	-0.03415
KEGG_LEIS	0.021584	0.429659	0.669492	-0.06717	0.149184	-0.40594	0.554266	-0.32674
KEGG_PAT	0.029458	0.07001	0.103148	-0.07165	0.020355	-0.31206	0.288236	0.103572
KEGG_COL	0.197878	-0.15238	-0.12842	-0.21749	-0.04736	-0.3522	0.327245	0.144428
KEGG_REN	-0.20451	0.07171	0.133265	-0.11786	-0.25716	-0.27172	0.33493	0.077004
KEGG_PAN	0.108815	0.099273	0.095334	-0.01302	0.05306	-0.28172	0.370123	0.180727
KEGG_END	0.08635	-0.15617	0.060969	-0.12534	-0.02967	-0.40849	0.395817	-0.03619
KEGG_GLI(	-0.18193	-0.21621	0.067061	-0.15294	-0.10641	-0.20668	0.391177	-0.07399
KEGG_PRC	-0.04362	0.066077	0.234884	-0.18647	0.00604	-0.27365	0.424328	-0.08997
KEGG_THY	0.029019	-0.01838	0.126154	0.025572	-0.0842	-0.06881	0.256226	0.177293
KEGG_BAS	0.126506	-0.34322	-0.28837	-0.36137	0.333926	-0.50627	-0.00457	0.159456
KEGG_MEI	-0.17375	-0.13308	-0.12195	-0.23101	-0.09859	-0.24563	0.336469	-0.05231
KEGG_BLA	-0.05096	0.112558	0.033771	-0.05809	-0.02202	-0.12421	0.474246	-0.14315
KEGG_CHR	-0.01032	-0.15229	0.142911	-0.09965	-0.13001	-0.20926	0.378704	-0.06675
KEGG_ACL	0.068552	-0.04643	0.176608	-0.16269	-0.0388	-0.39556	0.406043	0.088863
KEGG_SM/	-0.07404	0.153712	0.328011	0.152536	0.169989	-0.28592	0.39783	0.149418
KEGG_NON	-0.10093	-0.00274	0.25802	-0.05503	-0.04409	-0.16852	0.464215	-0.19142
KEGG_AST	-0.07873	0.313613	0.534714	-0.47852	-0.11216	-0.41155	0.483749	-0.29285
KEGG_AUT	0.243806	0.415807	0.450514	-0.32789	-0.07208	-0.37443	0.535862	-0.21205
KEGG_ALL(	0.368816	0.533079	0.583125	-0.4173	-0.06776	-0.38713	0.57763	-0.2746
KEGG_GR/	0.246297	0.54083	0.507607	-0.35439	-0.03216	-0.32876	0.619337	-0.08441
KEGG_VIR/	0.055288	0.326336	0.387569	-0.26986	-0.09825	-0.43101	0.37266	0.012156

TCGA-2J-A	TCGA-2L-A	TCGA-2L-A	TCGA-2L-A	TCGA-2L-A	TCGA-3A-A	TCGA-3A-A	TCGA-3A-A	TCGA-3A-A
0.121186	-0.07066	0.271684	-0.33711	-0.42047	-0.17777	-0.02885	0.089504	0.137931
0.222521	-0.3587	0.426917	-0.47343	-0.14799	0.219082	0.047525	-0.39326	-0.61187
-0.33837	-0.15386	0.318787	0.270139	-0.11196	0.12555	0.10872	0.214235	0.0707
-0.177	0.114373	-0.13669	-0.08961	-0.11729	0.101107	0.345773	-0.15267	-0.02543
0.012855	-0.19192	-0.15242	0.310355	0.06816	-0.22264	0.502683	0.153447	-0.11082
0.033741	-0.21193	0.142187	0.264521	0.075772	0.328278	-0.10556	0.137273	-0.33818
0.539202	0.176775	0.286032	-0.27142	-0.03278	-0.35069	0.003418	0.140193	0.133137
0.078224	0.049336	0.021263	0.093049	0.083633	0.333978	-0.10737	0.062727	-0.0419
0.058567	0.162378	0.009	0.227566	0.023806	0.090927	-0.11915	0.019111	0.006206
0.312594	-0.17237	0.028679	0.094313	0.354968	0.223605	-0.16664	-0.05256	-0.2175
0.262638	0.001288	-0.01741	0.384537	0.028083	0.298594	-0.05085	-0.1041	-0.04309
0.252642	0.029726	0.002132	0.257249	-0.12274	0.136002	-0.11839	0.092174	0.147363
0.007887	-0.23477	0.364948	0.356085	0.03198	0.453595	0.10383	-0.18262	-0.40509
0.077039	-0.08587	0.006984	0.487984	0.344165	-0.00838	0.191732	0.428815	0.381084
-0.20959	0.208886	-0.00203	0.43523	0.099589	0.251382	0.457311	0.219174	0.015388
-0.14167	-0.08779	-0.39685	-0.13802	0.103412	0.123118	0.35047	-0.25771	-0.42309
0.153464	-0.23287	0.120692	-0.16432	-0.01912	0.021651	0.09694	-0.03046	0.099095
0.209853	0.077943	0.104006	0.082832	0.021797	-0.08005	-0.08983	0.197281	-0.0873
0.340396	-0.28643	0.16287	-0.24688	-0.02846	0.36603	0.155363	0.095404	-0.17816
0.408796	0.193349	0.151547	-0.11663	-0.21191	-0.42113	-0.44446	0.120633	0.157028
-0.24095	0.314195	-0.14142	0.233051	-0.21502	-0.1176	-0.00685	-0.24695	0.175658
-0.59362	-0.0975	0.143186	-0.51988	-0.29599	-0.23262	-0.6158	0.197573	-0.42373
0.151564	0.21347	-0.09071	-0.01533	0.185065	0.107378	0.18307	-0.12012	-0.21671
-0.16288	-0.10033	0.106656	-0.1031	-0.04852	0.165142	-0.09498	-0.05085	-0.18132
0.11656	-0.01013	0.167145	0.224833	0.358437	0.321414	0.022838	0.44534	0.034153
0.074782	-0.03635	0.00947	-0.25949	-0.06845	0.546357	-0.031	-0.06247	-0.07704
-0.54065	-0.24873	-0.04197	-0.20088	-0.19867	-0.37497	0.234449	-1.16E-05	0.344949
-0.36373	-0.00464	-0.02088	-0.06688	-0.16474	0.181817	-0.18815	-0.21001	-0.0066
0.445604	-0.19843	0.485241	0.006022	0.285633	0.275057	-0.42939	0.287884	-0.50041
-0.4908	-0.05352	0.003228	0.261549	-0.0344	0.397128	0.078748	0.249596	0.002907
0.014274	-0.51856	-0.24967	-0.33872	0.022304	-0.1228	0.182293	-0.53632	-0.44961
-0.20768	0.326508	-0.26283	0.097917	-0.17456	0.397171	-0.22967	-0.44464	-0.40763
-0.37259	-0.19318	-0.04444	0.194129	-0.03728	0.279754	-0.00742	0.11618	0.097578
-0.42544	-0.22935	-0.03513	0.153366	-0.12622	0.356628	0.073593	0.203999	0.139294
-0.35938	-0.13065	-0.03484	0.05225	-0.05296	0.229151	0.056325	0.099216	-0.03664
-0.05144	0.014031	0.411844	-0.14405	-0.17589	0.370183	-0.01266	0.320602	-0.00144
0.130918	-0.29262	0.432747	-0.42311	-0.16004	0.58263	-0.57008	0.54605	-0.50757
0.246981	0.226915	0.370637	-0.15458	0.015735	0.160505	0.108672	0.249487	0.387215
0.131599	0.524307	0.456624	0.060106	-0.11342	0.563954	-0.40359	-0.40623	-0.32062
0.012035	-0.04879	0.507561	-0.14065	-0.20871	0.057764	-0.08708	0.288248	-0.07639
-0.02624	0.132228	0.183868	-0.34955	-0.27615	0.170207	-0.13449	0.317559	-0.0899
-0.05667	0.207125	0.428732	0.367	0.072306	0.513483	-0.08335	-0.24728	-0.46191
0.421132	-0.34509	0.453129	-0.18875	0.107285	0.57571	-0.28153	0.18033	-0.41374
0.371113	-0.39833	0.721694	0.435906	0.424623	-0.51109	0.173286	0.474452	0.155357
0.036205	-0.23839	0.335304	-0.1077	0.186521	-0.049	-0.07986	-0.3889	-0.41601
0.088296	0.074166	0.218178	0.373305	0.177152	0.081914	-0.28688	-0.07961	-0.24846
0.296351	0.049037	0.141352	0.282234	0.434018	0.404343	-0.10637	0.499853	-0.12167
0.208634	-0.05111	0.208516	-0.13541	0.007424	-0.13093	0.03212	0.036067	-0.32594
0.285981	0.05613	0.005593	-0.02857	0.067698	-0.19301	0.022109	0.17857	-0.158

0.46651	0.062428	0.223605	-0.02302	-0.02063	0.060723	-0.13873	0.07898	-0.22451
0.558825	0.037088	0.251937	-0.24694	-0.03005	0.181563	-0.22773	-0.22883	-0.48166
0.066703	-0.13887	0.327349	0.056745	-0.22578	0.21211	-0.07816	-0.01622	-0.10409
0.55622	-0.27201	0.334403	-0.41682	-0.08652	0.143144	-0.38505	0.270386	-0.18849
0.147228	-0.42496	-0.11861	-0.21481	-0.30904	0.028035	-0.09862	-0.15272	-0.09361
0.378743	-0.22658	0.338554	0.126439	0.148751	0.26386	0.043193	0.286098	-0.31872
0.323275	-0.08196	-0.03694	0.095561	0.181808	0.152312	-0.14514	0.209287	0.009177
0.375804	-0.06691	0.034809	-0.04376	0.190944	0.00024	-0.14303	0.154808	-0.03709
0.373806	-0.07755	0.131297	0.128877	0.109632	-0.12202	-0.4427	0.34526	-0.03386
0.29237	-0.39993	0.135888	-0.05178	0.176417	0.151016	-0.33168	-0.05894	-0.443
0.172721	-0.35981	0.409534	-0.14288	-0.13614	0.156364	-0.2578	0.011183	-0.41881
0.480007	-0.32399	0.465666	0.124453	0.320057	-0.39678	-0.19309	0.004183	-0.48651
0.359654	0.066844	0.243081	-0.20106	-0.27855	-0.10098	-0.38726	-0.0685	-0.2263
0.304336	-0.26514	0.278319	0.127182	0.280809	0.057814	0.011703	0.158352	-0.23409
0.14335	0.352556	0.264465	-0.03276	-0.26308	0.377886	-0.17397	0.128376	-0.08096
0.113847	-0.06043	0.121131	-0.23878	-0.08992	-0.33865	0.343969	-0.02062	0.006729
-0.19645	0.044918	-0.47322	-0.28403	-0.15205	0.197454	0.598245	-0.17729	-0.0539
0.016047	0.212834	-0.17729	0.162998	-0.12068	0.067322	0.519134	-0.19444	-0.03198
-0.377	0.130214	-0.03553	0.040557	-0.25263	0.350237	-0.23291	-0.20823	-0.31234
0.312598	-0.25258	0.251329	-0.12834	0.079556	0.267585	-0.12939	0.294133	-0.41266
0.53711	-0.00979	0.082225	0.096422	-0.02474	0.125829	-0.27061	0.473696	-0.7333
0.421536	-0.59069	0.513833	-0.21136	0.106848	0.127076	-0.35414	-0.0181	-0.4598
0.390949	-0.33112	0.435846	-0.15135	0.043564	0.15698	-0.17269	0.291775	-0.34047
0.481462	0.364946	0.265866	-0.2744	-0.34313	-0.47215	0.01795	0.361178	-0.64001
0.450572	0.23243	-0.0318	0.131444	0.485481	-0.05263	0.163746	0.529278	-0.21404
0.111622	0.143006	0.287195	0.297483	0.224792	0.402616	-0.33675	-0.1339	-0.43657
0.291732	0.415879	0.40144	0.193834	0.284789	0.285825	-0.19465	0.096972	-0.38632
0.075685	-0.38081	0.625365	0.164241	0.611671	-0.07878	0.256007	0.60539	-0.17792
0.38787	-0.197	-0.08246	0.380844	-0.05818	0.124712	-0.04657	-0.33452	-0.01456
-0.04317	-0.38218	0.502416	0.132602	0.208477	0.569464	-0.1286	-0.15708	-0.17591
0.330146	0.038803	0.223658	0.277958	0.249862	0.427771	-0.22269	-0.1735	-0.28084
0.33039	0.039884	0.254007	0.251834	0.236546	0.374509	-0.29026	-0.19302	-0.28373
0.207952	0.314886	0.112608	0.331253	0.197904	0.415101	-0.17788	0.057486	-0.26227
0.067703	-0.14632	0.478566	0.39178	-0.02219	0.305289	-0.31859	0.183173	0.061972
-0.17968	-0.03753	0.116166	0.056071	-0.08396	0.385394	-0.17564	-0.09124	-0.33872
0.713305	-0.01481	-0.41223	-0.31757	0.40694	-0.176	-0.49445	-0.64106	-0.52152
0.06981	-0.01796	-0.17524	-0.24808	-0.10934	-0.30805	-0.38541	0.061962	0.191001
0.56085	-0.14768	-0.27701	-0.2869	0.116861	-0.34073	-0.157	-0.20172	-0.09927
0.426716	0.125487	0.312641	-0.21274	0.10497	-0.38907	0.105872	0.268452	-0.01139
-0.06359	-0.06355	-0.01023	-0.13712	0.026953	-0.40897	-0.29151	-0.35017	0.156611
0.326382	0.048795	0.191929	0.479081	0.29066	0.000752	-0.15274	0.267693	-0.12692
0.571301	-0.49273	0.278121	-0.15067	-0.12327	-0.45157	-0.35258	0.016879	-0.23339
0.156029	0.057931	0.209058	0.041508	0.164392	0.41558	-0.23265	0.163357	-0.39865
0.26457	0.318285	0.018768	-0.18237	0.249643	-0.25491	-0.12152	0.155734	0.108392
0.14364	0.180098	-0.25845	-0.31486	-0.16049	-0.44776	-0.36558	0.094342	0.250004
0.201964	0.007276	0.03619	-0.37303	-0.27097	-0.53529	-0.19767	0.285812	0.162846
-0.04305	0.397729	-0.03521	-0.2108	-0.40812	-0.44286	0.27998	0.130287	0.108211
-0.28429	-0.11532	-0.05842	0.161872	-0.08914	0.16469	0.037756	0.02706	-0.03299
-0.35449	-0.0794	0.084561	0.082166	-0.42283	0.148458	-0.23288	-0.09196	-0.04734
-0.22119	-0.10471	-0.07689	0.076757	0.079185	0.307	0.035853	0.107071	-0.23606

-0.1813	-0.16254	-0.16598	0.183164	-0.04031	0.137313	0.085661	-0.28283	-0.21744
-0.34941	-0.1835	-0.23143	0.091863	-0.16658	0.169215	-0.1239	-0.2389	-0.24049
-0.42777	0.180182	-0.14655	0.183325	-0.1885	0.338966	-0.22667	0.010661	-0.33089
0.042559	-0.15869	0.005291	0.039711	0.205416	0.084662	0.091526	-0.01068	-0.2694
-0.28394	-0.00263	0.102089	-0.02223	-0.24852	-0.1792	0.006651	0.192915	-0.14373
-0.28618	0.062897	0.180133	-0.04073	-0.28017	-0.1405	0.002942	0.194278	-0.21665
-0.18823	-0.00236	-0.00975	0.201933	-0.1655	0.049909	0.056442	0.145725	0.002837
-0.30013	0.085666	-0.05982	-0.10838	-0.24921	-0.25968	-0.16673	0.112647	0.229449
0.07886	-0.24341	0.075234	-0.13165	0.040575	-0.21668	-0.0022	0.063114	-0.02669
-0.31232	0.021289	0.012664	0.111025	-0.16127	0.173986	0.00781	-0.04078	-0.0603
0.291519	0.386578	0.504267	-0.03382	0.174197	0.408003	-0.24149	0.235381	-0.01709
-0.14075	-0.04968	-0.26164	0.104101	-0.19732	-0.08506	-0.21191	-0.14956	-0.05084
-0.35177	0.045464	-0.04691	0.150775	-0.22404	0.021927	-0.24519	0.08037	-0.1149
-0.25671	0.002667	-0.10491	0.076052	-0.04406	0.226085	-0.00694	-0.01842	-0.22914
-0.27831	0.080374	-0.05985	0.115587	-0.10402	0.209092	0.037796	0.075596	-0.03417
-0.48649	-0.32271	0.388273	0.180813	-0.44828	0.062178	-0.24767	-0.02578	0.176483
-0.34832	-0.03635	-0.09666	0.148127	-0.08631	0.033368	0.124109	-0.12098	0.274229
-0.4298	0.292251	0.012323	-0.09346	0.020042	0.215295	0.079378	0.29852	-0.25167
-0.43317	-0.19838	-0.01961	-0.37369	-0.36505	0.201084	-0.04699	-0.18293	0.257856
-0.42415	0.102315	0.136711	0.229084	-0.09994	0.063375	0.093684	0.271525	0.140334
-0.25772	0.015253	-0.12238	0.183799	-0.13329	0.197853	-0.00503	0.00212	-0.03942
-0.46685	-0.08853	-0.07901	-0.00151	-0.33443	0.359207	0.105649	-0.02279	0.042953
-0.48898	-0.19076	0.009068	-0.17808	-0.31612	0.410002	0.369124	-0.0426	0.07798
-0.32625	-0.42149	-0.02878	0.081269	0.00786	0.390678	0.129098	-0.10548	-0.33917
-0.46691	-0.28597	0.007808	0.238982	-0.30431	0.26129	0.067082	0.057353	0.201671
-0.34383	0.029401	0.186814	0.287601	-0.03977	0.136022	0.041044	0.18794	0.271787
-0.40871	-0.17334	0.096727	-0.02928	-0.19511	0.208789	0.075231	0.157128	-0.14817
0.122965	-0.233	0.272408	-0.13352	0.143732	-0.30762	-0.14665	-0.24205	-0.06798
-0.36945	-0.3951	0.022447	-0.10859	-0.05735	0.208892	0.350723	-0.30244	-0.37525
-0.34152	-0.00751	0.004035	0.021199	-0.29831	-0.01055	-0.06667	-0.10354	-0.05924
-0.32251	-0.28921	-0.16296	0.06787	-0.35682	-0.23483	-0.07568	-0.20746	-0.12257
-0.22701	0.295708	0.024496	-0.05573	-0.14724	0.184088	0.054313	0.227038	0.077273
0.167354	0.190004	-0.04678	-0.2292	0.187997	0.122628	-0.01836	-0.10711	-0.02236
-0.28805	-0.00076	-0.20973	0.067973	-0.2959	0.064885	-0.07458	-0.36825	-0.18293
-0.15727	-0.29513	-0.21533	-0.00768	-0.20308	0.303728	-0.05549	-0.39471	-0.43714
-0.3657	-0.18486	-0.10764	0.24239	-0.26654	0.280348	-0.10647	-0.12547	-0.13041
-0.42967	0.007583	-0.05432	0.194841	-0.40404	0.229195	-0.18351	-0.1859	-0.21631
-0.39998	0.053212	-0.0164	0.144546	-0.2813	0.24451	-0.22895	-0.04173	-0.16276
-0.30287	-0.07798	-0.09471	0.112323	-0.3083	0.15448	-0.2754	-0.04798	-0.3019
-0.46078	-0.01687	-0.06865	0.162078	-0.17978	0.185959	-0.12977	-0.07692	-0.20726
-0.45272	-0.1469	-0.05692	0.386895	-0.1476	0.242596	0.034037	-0.01542	-0.023
-0.19739	-0.36377	-0.35006	0.112362	0.125531	0.451267	0.069423	-0.44397	-0.50022
-0.38429	0.049099	0.003578	0.176306	-0.16529	0.131619	-0.19301	-0.00766	-0.2242
-0.43125	0.094125	-0.13593	0.138331	-0.34858	0.253183	-0.11996	-0.09449	-0.22454
-0.22466	-0.12447	0.019035	0.033297	-0.19573	-0.01781	0.007261	0.109431	0.02114
-0.09873	0.031246	0.228266	0.026328	-0.06467	0.239994	0.155759	0.22949	0.086579
-0.41626	-0.06513	-0.0621	0.105786	-0.1555	0.33079	0.037314	-0.02806	-0.02271
-0.20581	0.080608	-0.09908	0.028477	-0.22782	0.225674	-0.20379	0.013489	-0.12266
-0.30275	-0.03432	-0.05036	0.101003	-0.24169	0.174412	-0.1317	0.040793	-0.08578
-0.19075	0.028346	0.04204	-0.11213	-0.23345	-0.25281	-0.03814	0.087724	-0.22472

-0.37245	0.112399	-0.07981	-0.16045	-0.08765	0.292546	0.207574	0.015018	-0.09576
-0.21928	-0.02944	-0.16939	-0.01911	-0.09684	0.27318	-0.20615	0.064552	-0.13847
-0.2999	0.033141	0.028953	-0.07358	-0.04025	-0.01681	-0.27594	0.092634	-0.35164
-0.27633	-0.51387	-0.08972	-0.2519	0.015198	0.180235	0.22044	-0.31416	-0.55937
0.507511	-0.53382	0.618788	-0.20385	0.554104	-0.12282	-0.40909	0.275954	-0.31243
-0.26409	-0.07059	-0.05865	0.221295	-0.09364	-0.10074	-0.07845	-0.01813	-0.21548
-0.33868	-0.19751	0.36337	-0.03013	-0.18778	-0.11923	-0.36237	0.190401	-0.03886
0.150855	-0.1062	0.196934	0.199456	0.276651	0.317367	-0.01176	0.439575	-0.07385
0.316637	0.040992	0.184177	0.076441	0.404107	0.46007	-0.01546	0.494032	-0.25611
-0.02365	-0.01916	0.119604	0.118968	-0.07541	0.183367	0.015504	0.056588	-0.12963
0.256904	0.070494	0.101706	0.148211	0.246456	0.315019	-0.0467	0.415315	-0.17149
-0.25711	-0.3257	-0.03453	-0.02769	-0.1278	-0.13733	0.142404	-0.33059	-0.44223
-0.26103	-0.13729	0.060224	0.389663	-0.15297	0.081419	-0.11281	0.222514	-0.07562
-0.38726	0.033963	0.028661	0.364603	-0.33417	0.01862	-0.13441	0.133302	0.066871
-0.36845	-0.42102	-0.16134	-0.05738	-0.16781	0.045999	-0.00839	-0.31761	-0.32658
-0.38288	0.03965	-0.07864	0.005168	-0.20628	0.17514	0.044632	0.030354	0.00722
-0.34168	0.087696	-0.15453	-0.02011	-0.35106	0.058141	-0.18188	0.102665	-0.21384
-0.50619	-0.13226	0.111166	-0.07443	-0.42029	0.196112	-0.2904	-0.17578	0.022523
-0.37434	0.142155	-0.00291	0.100521	-0.32227	0.081109	-0.12543	0.166318	-0.08748
-0.32863	0.0741	-0.00503	0.083963	-0.3759	0.169242	-0.34638	-0.06188	-0.11429
-0.39339	-0.10256	-0.03498	0.096279	-0.37267	0.307486	-0.17741	-0.14288	-0.10288
-0.34154	-0.10323	-0.08947	-0.09819	-0.31935	0.177645	-0.13705	-0.05754	-0.06497
-0.408	-0.07796	0.149044	-0.03135	-0.20584	0.258982	-0.33849	0.224753	0.115138
-0.3797	0.356489	-0.22867	-0.18338	0.104548	0.406978	0.363111	0.11661	-0.0886
-0.25526	-0.07817	-0.0345	-0.03305	-0.13146	0.114549	-0.00899	-0.11208	0.082618
-0.10674	-0.11815	0.005702	0.134408	-0.22992	0.06377	-0.20817	-0.17524	0.08125
-0.4407	0.012874	-0.17634	0.075697	-0.3675	0.226764	-0.21221	-0.11066	-0.09145
-0.34825	-0.08132	-0.13128	-0.09344	-0.32771	0.260933	-0.15548	-0.1809	-0.03207
-0.43045	0.008923	-0.06909	-0.07002	-0.39828	-0.02329	-0.14078	0.011569	0.030536
-0.36102	0.040745	0.105176	0.034298	-0.26052	0.206366	-0.30744	-0.05272	-0.02224
-0.11677	-0.43983	-0.3494	-0.53646	0.038752	0.386086	0.35962	-0.50687	-0.5427
-0.27244	-0.44707	-0.20128	-0.29807	-0.05209	0.231804	0.379848	-0.37873	-0.4681
-0.31704	-0.56357	-0.18829	-0.29655	-0.0469	0.283339	0.378015	-0.45502	-0.55082
-0.30533	-0.60123	-0.21418	-0.17002	-0.01398	0.188914	0.227283	-0.49382	-0.52962
-0.42452	-0.4051	-0.1104	-0.29814	-0.14974	0.27941	0.356078	-0.11549	-0.26041

TCGA-3A-A	TCGA-F2-6	TCGA-F2-6	TCGA-F2-A	TCGA-F2-A	TCGA-F2-A	TCGA-FB-A	TCGA-FB-A	TCGA-FB-A
0.249327	-0.05051	0.412507	-0.20366	0.214643	-0.10473	-0.15831	-0.38726	-0.35466
0.006171	-0.36159	0.473332	0.138127	-0.59835	0.121194	-0.21771	-0.23186	-0.20263
0.220262	0.328769	-0.11379	0.124808	-0.34512	0.137518	-0.28622	0.127796	0.19462
-0.13542	-0.2183	-0.13269	-0.13864	-0.4484	0.142978	-0.03526	-0.26163	0.222186
-0.15946	-0.04103	-0.15989	-0.03251	-0.14765	0.446228	0.305753	-0.25974	0.123798
0.226987	0.041196	-0.0023	0.160229	0.019218	0.179097	-0.11932	0.353449	-0.03603
0.489095	-0.31292	0.468604	-0.17913	-0.51538	-0.25389	0.024786	-0.02801	-0.06435
0.156635	0.032615	-0.07934	0.218421	-0.10605	0.18174	-0.04872	0.232101	-0.07978
0.212775	0.315254	0.042033	0.10635	0.185634	0.252333	-0.32161	0.047658	-0.23373
-0.04162	0.054808	-0.01649	0.040837	-0.13757	0.274436	-0.14919	0.164201	-0.23146
0.229592	0.357628	-0.06446	0.293224	-0.06727	0.452718	-0.32164	0.337622	-0.23316
0.370888	0.13429	0.200951	0.248113	0.373617	0.243	-0.32245	0.255434	-0.15637
0.113912	-0.01304	-0.02387	0.094484	-0.42426	0.176441	-0.36484	-0.22112	0.159797
0.208545	0.012054	-0.12333	0.173426	-0.05456	0.034584	0.251473	-0.00321	-0.22297
-0.14884	0.06627	-0.3711	-0.10353	0.276587	0.438268	0.219124	-0.49673	0.187063
-0.13459	0.094792	-0.12026	-0.04149	-0.33908	-0.06546	0.226184	-0.50586	0.23122
-0.25673	-0.10555	0.177918	0.157488	0.006578	0.071327	-0.1965	-0.04615	0.050204
0.220881	0.047336	0.04343	-0.28109	0.08826	0.307927	-0.23991	-0.18651	0.132398
0.143341	-0.35782	0.343194	0.173126	0.420796	0.323944	0.015485	-0.19373	-0.49453
-0.30235	0.011605	0.213061	0.30954	0.442789	0.492273	0.101874	0.374518	-0.55465
-0.0719	0.161332	-0.36006	0.232116	0.285791	0.007061	0.02608	0.017023	-0.18315
-0.20737	0.223222	-0.17357	-0.2772	0.407885	0.069809	-0.04677	-0.30278	-0.16334
-0.17903	-0.10438	0.017639	-0.15803	-0.28454	-0.10647	0.06557	0.122074	0.131419
-0.30118	-0.19362	-0.04654	-0.1431	-0.18942	0.116185	-0.08728	-0.16447	-0.06751
-0.17424	-0.03036	-0.11378	0.228038	-0.11674	-0.11997	0.114582	-0.00912	0.11543
-0.23789	0.085904	0.078617	-0.07907	-0.02957	-0.32969	0.081635	-0.18016	0.326456
0.060161	-0.20003	-0.18918	0.463175	-0.29648	-0.0654	-0.37131	0.250578	-0.26734
-0.09328	0.096214	-0.18583	0.135308	-0.2315	-0.17277	-0.30309	0.255075	-0.11604
-0.02349	0.014935	0.456973	0.156247	0.208726	0.233541	-0.20334	0.370661	0.369043
-0.1414	0.043162	-0.48219	0.054549	-0.03566	0.027954	-0.09762	-0.14603	0.295531
-0.43762	-0.31814	-0.56727	-0.09594	0.481253	-0.1165	0.192536	-0.05473	0.166971
-0.41475	-0.32695	-0.54544	-0.22237	0.51397	-0.26234	0.39883	-0.17212	-0.14596
0.250921	0.128548	-0.33029	0.083403	-0.33863	-0.32055	-0.27106	-0.18918	0.398801
0.068516	-0.0243	-0.39053	0.070901	-0.26744	-0.29467	-0.28237	-0.25211	0.457774
0.099466	0.051236	-0.27053	0.049101	-0.3194	-0.31661	-0.25839	-0.20142	0.440421
0.285226	0.0115	-0.18968	0.291127	0.090693	0.273693	-0.1348	-0.00728	0.15231
-0.13603	-0.01381	0.489559	0.046642	0.59221	0.469933	-0.02974	-0.51801	0.095225
0.093743	0.306513	-0.19034	0.266095	0.191338	0.490939	0.207619	0.35149	-0.09251
0.173047	0.460713	-0.14345	0.285116	-0.06731	0.373282	-0.16765	0.108149	-0.41026
0.258974	0.137197	-0.0516	0.393869	0.01482	0.106194	-0.13343	0.218461	0.056016
0.099649	0.01487	-0.24186	0.204128	0.040443	0.319138	0.256641	0.305221	-0.01607
0.184393	0.413332	-0.03015	0.069655	0.0337	0.102993	-0.40155	-0.08141	-0.30917
0.003351	-0.02308	0.443817	-0.2121	0.362186	0.348965	-0.18933	0.126482	-0.11735
0.426831	-0.20154	0.190232	0.316514	-0.26863	0.252354	-0.10055	0.123935	-0.62522
-0.40569	-0.04635	0.293998	-0.39312	-0.27871	0.57684	-0.38084	-0.2608	-0.31638
-0.0021	0.254534	-0.24296	0.246404	-0.22723	0.400793	-0.14526	0.104534	-0.36576
-0.28247	-0.06179	0.152948	0.072472	0.077385	0.107454	0.373513	-0.08867	-0.0343
0.121889	-0.02536	-0.01328	-0.02391	0.165333	-0.11712	0.140761	-0.13757	0.044555
0.197009	0.051578	-0.16877	0.036787	0.365892	0.004107	0.359032	-0.04016	0.000556

-0.09047	0.053644	0.371393	0.209369	0.339929	0.330317	-0.03657	0.184083	0.061739
0.068813	-0.2336	0.63612	-0.09866	0.324989	0.124026	0.061436	0.120462	0.236184
-0.01925	-0.26691	0.09444	0.225061	0.41603	0.359985	0.033726	0.208091	0.121634
-0.11856	0.228762	0.693653	-0.22923	0.331861	0.467655	-0.20304	0.007717	-0.41292
-0.25655	0.074606	0.471396	-0.25759	0.296598	-0.07655	-0.08666	0.159049	-0.03778
0.082236	0.163503	0.093287	0.11807	-0.08316	0.406082	-0.08269	0.141409	0.182864
0.003789	0.040544	0.335675	-0.23853	-0.21763	0.018197	0.078841	0.174402	0.045136
-0.04501	-0.31785	0.205965	-0.09387	-0.00042	0.195207	0.274433	0.124759	0.194423
-0.09433	-0.10046	0.152799	0.152113	-0.11698	0.16085	0.31009	0.224552	0.50557
-0.18192	-0.14482	0.226297	0.009344	0.228511	0.042182	0.18537	0.21887	0.149682
-0.05958	0.191599	0.356587	-0.22746	0.275312	0.206168	-0.24563	0.019269	-0.11153
-0.27901	-0.24705	0.39199	-0.21044	-0.5228	0.014896	-0.36888	0.292492	-0.35879
-0.11119	-0.0359	0.452626	0.097618	0.133944	0.45075	-0.23764	0.241069	-0.3594
0.290577	-0.12368	-0.04654	-0.02262	-0.05137	0.201141	0.009074	-0.15726	-0.33411
0.334119	0.303222	0.022182	0.274328	0.119364	0.39243	-0.09719	-0.17043	-0.17794
0.075588	-0.30016	-0.08305	0.077003	-0.06665	0.441738	0.031843	-0.09612	-0.23186
0.203634	-0.26938	-0.35842	0.274018	-0.43031	-0.14159	0.108357	-0.5145	0.3831
0.223145	0.064591	0.064591	0.160508	-0.27002	0.33638	-0.24301	-0.3749	0.097635
-0.18772	0.067916	0.028498	-0.04626	-0.24167	-0.25023	-0.37867	0.118827	0.266265
0.088064	-0.06176	0.275525	-0.11328	0.230302	0.366186	-0.09083	-0.14882	0.060116
0.349273	-0.15425	0.422788	0.32127	0.609916	0.054763	0.38719	-0.23985	0.196491
-0.26942	0.06172	0.579719	-0.218	0.196718	0.184768	-0.27831	-0.06942	-0.26413
0.042377	0.089197	0.430437	0.006708	0.113879	0.39052	-0.22345	0.020434	-0.22938
0.41739	-0.26416	0.220959	0.440655	0.460838	-0.24212	0.336908	-0.07614	-0.13621
0.431948	0.488263	-0.10729	0.121015	0.286413	0.198857	0.359635	-0.16383	-0.43733
0.036677	0.373253	-0.15561	0.08792	-0.12541	0.396233	-0.13404	0.060225	-0.25643
-0.07839	0.329938	-0.07853	0.256341	0.293996	0.22125	-0.03397	-0.27133	0.143543
0.095996	0.158637	-0.02627	0.325399	0.080324	0.571508	-0.19986	-0.2064	-0.49307
0.369542	0.253107	0.297093	0.472788	-0.25945	0.220443	-0.24145	0.313666	0.125111
-0.04467	0.299132	-0.13996	-0.27885	-0.17026	-0.26151	-0.25	0.105659	-0.34215
-0.08942	0.143182	-0.11225	-0.05877	-0.17741	0.389865	-0.14409	0.085896	-0.20914
-0.1906	0.126675	0.072224	-0.09392	-0.18592	0.359383	-0.11854	0.131575	-0.2031
0.071343	0.339208	-0.28778	0.296265	0.03715	0.355905	0.169005	0.245309	-0.04652
0.477044	0.244813	-0.05691	-0.06679	0.441964	0.068574	-0.02515	-0.0264	0.013538
0.000252	-0.09891	-0.041	-0.02994	-0.15024	0.163983	-0.24744	0.060173	0.157444
-0.36649	-0.28045	0.579097	0.200878	0.070421	-0.18626	0.62299	0.342844	-0.65089
0.054279	0.030675	-0.06503	0.103023	0.430956	-0.1633	0.215607	0.018477	-0.426
-0.2589	-0.09394	0.260432	-0.08743	0.203667	-0.44968	0.637458	0.051898	-0.34241
0.401634	0.051559	-0.23562	-0.13836	0.62938	-0.05857	0.63639	-0.33594	-0.08837
-0.12869	-0.15565	-0.3017	0.007046	0.350874	-0.3316	0.211138	0.155058	-0.36277
-0.40447	-0.04244	-0.42467	-0.31639	0.624676	-0.42423	0.676656	-0.39959	0.358996
0.026359	0.014945	0.77884	-0.37217	0.25667	-0.17762	-0.2119	-0.57526	-0.32269
0.292232	-0.00515	0.095095	0.065154	0.222543	0.264614	-0.0161	0.034797	-0.04717
0.125935	0.121791	-0.43415	0.055015	0.336868	0.092477	0.480968	0.277684	-0.46339
-0.0522	0.078357	-0.04789	-0.11049	0.424189	-0.00858	0.323025	-0.21922	-0.16901
0.314746	0.035084	-0.15928	0.086412	0.52931	-0.09735	0.486735	-0.32242	-0.28265
0.479053	0.125375	-0.48972	0.187433	0.433848	0.002213	0.326022	0.067635	-0.25797
0.036486	-0.0693	-0.15308	0.111926	0.02994	-0.15222	-0.22034	-0.13566	0.226614
-0.02924	-0.0564	-0.02572	-0.02689	0.157232	-0.15317	-0.18876	-0.09792	0.319413
-0.10343	0.051104	-0.09723	-0.08899	-0.03429	-0.03354	-0.11401	-0.1477	0.313467

-0.03995	-0.21592	-0.3796	0.10064	0.202624	-0.2283	0.213848	-0.13339	0.251408
-0.25127	-0.12602	-0.36704	-0.03804	0.237431	-0.05525	-0.07041	-0.22839	0.140424
-0.20664	0.067008	-0.17898	-0.10804	-0.26955	-0.19681	-0.29978	0.152904	0.288784
-0.16134	-0.18215	0.101019	-0.15312	0.07379	-0.1229	0.000508	-0.07492	0.230076
0.307659	0.237441	-0.4257	0.236622	0.503513	-0.0867	0.248329	-0.20949	0.051215
0.214423	0.283392	-0.26904	0.15853	0.302868	-0.13311	-0.05749	-0.1409	0.154916
0.241379	0.203301	-0.35577	0.365155	0.345069	0.009273	0.115071	-0.09998	-0.05109
0.082505	0.097621	-0.05265	0.089477	0.380869	-0.11291	-0.15089	-0.18109	-0.02816
-0.19095	-0.17926	0.332595	-0.14571	0.091124	-0.01192	0.221709	0.265122	0.383873
-0.10888	0.122255	-0.15451	0.051708	-0.00348	0.024651	-0.15243	-0.04519	0.101585
-0.1475	0.037629	0.311195	-0.16656	0.150341	0.432632	-0.12923	0.161282	-0.31339
-0.08721	-0.12686	0.164243	-5.79E-05	0.125437	-0.11969	-0.10336	-0.17273	0.103673
-0.05056	-0.03307	-0.37492	0.217751	0.226569	-0.18417	-0.1885	-0.0852	0.020319
-0.19622	0.058064	-0.01923	-0.12013	-0.05852	-0.1306	-0.32285	-0.10008	0.376554
-0.07343	0.142593	-0.15314	-0.08056	0.012931	0.150595	-0.08631	-0.31509	0.245221
0.072321	-0.21536	-0.20024	-0.05016	0.132742	-0.20247	-0.44046	-0.14714	0.414719
-0.28755	-0.10875	0.042557	-0.10047	0.04586	-0.09937	-0.26284	0.03479	-0.01632
-0.20948	0.230128	-0.24294	-0.08746	0.024626	0.181083	-0.09002	-0.22364	0.220221
-0.0943	0.16254	-0.19232	-0.02716	-0.0536	-0.22166	-0.2889	-0.34631	0.228929
-0.06854	0.045735	-0.29846	-0.03041	0.116302	0.253573	-0.21441	-0.31165	0.276095
0.159493	0.040085	-0.1593	0.159321	0.052085	0.152301	-0.21581	-0.10687	-0.0316
-0.07595	0.068216	-0.28344	-0.04501	0.117071	-0.08701	-0.28816	-0.39165	0.389513
0.202638	0.062046	-0.48433	0.055063	-0.11796	-0.09482	-0.29599	-0.42927	0.515705
-0.39984	-0.26054	-0.43129	-0.32751	0.087157	-0.21568	-0.01952	-0.38869	0.344919
0.088019	0.070548	-0.28946	0.028733	0.029316	-0.02922	-0.32748	-0.24828	0.251796
0.116648	0.094636	-0.16694	0.057633	-0.07982	0.181074	-0.2696	-0.15946	0.177255
-0.12596	-0.00539	0.00197	-0.10233	-0.12231	-0.15401	-0.23458	-0.1153	0.397816
0.063108	-0.30124	-0.30537	-0.16185	0.186171	0.079382	-0.04181	-0.06263	0.321701
-0.14661	-0.17465	-0.54255	-0.21074	0.465875	0.273325	-0.13588	-0.25804	0.284678
-0.0873	-0.10472	-0.36446	0.132698	0.31508	-0.22588	-0.06065	-0.05491	0.077467
-0.00744	-0.16969	-0.42707	0.166587	0.43406	-0.24448	-0.06185	-0.16239	0.062675
-0.04055	-0.21395	-0.30126	-0.04817	0.285369	-0.21197	0.140727	0.066606	0.245315
-0.27678	-0.20117	-0.27793	-0.05789	0.414105	-0.36697	0.316908	0.121619	0.209566
-0.11072	-0.00828	-0.28363	0.062355	0.206995	-0.23979	-0.04375	0.066018	0.188277
-0.17357	-0.23586	-0.48389	0.058782	0.219503	-0.28649	0.214139	-0.06635	0.048549
-0.08793	-0.1467	-0.39511	0.095421	0.347394	-0.11059	0.016266	-0.10154	0.161087
-0.09139	-0.07996	-0.34664	-0.00959	0.298995	-0.14611	-0.21893	-0.22765	0.031532
-0.21413	-0.05902	-0.32623	0.071083	0.281478	-0.17202	-0.17933	-0.17771	-0.01592
-0.04188	0.068141	-0.18141	0.021129	0.131491	0.020729	-0.24653	-0.01866	-0.03674
-0.18224	0.090552	-0.31764	-0.09913	0.06151	0.099953	-0.13373	0.00976	0.030979
-0.11743	-0.11157	-0.43757	-0.18563	0.095597	0.130955	-0.17628	-0.33045	0.215668
-0.46411	-0.39586	-0.57299	-0.20113	0.490665	0.064483	0.119584	-0.23742	0.086136
-0.03918	0.334667	-0.11694	0.082194	0.127912	0.025595	-0.29687	-0.07903	0.082959
-0.21873	0.017838	-0.26102	-0.0647	0.185021	-0.06451	-0.24886	-0.29526	0.154954
0.182536	0.082105	0.018616	-0.04804	0.163564	-0.04691	-0.30675	-0.18918	0.150398
0.087297	0.258312	0.12566	0.269294	0.06938	0.062319	0.076702	0.233058	0.157853
-0.10181	0.102061	-0.30114	-0.06415	0.182501	-0.15975	-0.14354	-0.21991	0.333895
-0.04568	-0.0074	0.004333	0.02698	0.12042	0.030638	-0.09779	0.006023	0.201624
-0.02412	0.120849	-0.09941	0.105221	-0.06656	-0.05981	-0.30552	0.112304	0.17732
0.225635	0.086886	-0.15711	0.132415	0.276467	-0.07876	0.015631	-0.243	0.260845

-0.11535	0.152457	-0.0799	-0.10918	-0.10447	0.027874	-0.14345	-0.25757	0.318384
-0.05779	-0.08156	0.043461	0.046529	0.190505	0.006759	-0.1706	-0.06961	0.331502
0.041364	0.073263	-0.04469	-0.06077	0.24998	0.047329	-0.22286	0.068111	-0.02312
-0.41804	-0.35844	-0.52475	-0.1736	0.525595	0.030994	0.250083	-0.16155	-0.07303
-0.20849	-0.24697	0.64129	-0.46543	0.03594	-0.33557	-0.29584	0.57251	-0.43168
0.027588	0.146595	-0.04829	0.0077	0.135677	0.144744	-0.19101	-0.0724	0.198177
-0.33722	-0.14405	-0.10565	-0.43145	-0.13621	-0.32998	-0.34805	-0.08307	0.243356
-0.20811	0.142881	0.021838	0.095979	0.110794	0.022048	0.110195	-0.00944	-0.00595
-0.20043	-0.10924	0.180847	0.124823	0.168485	0.111327	0.219999	-0.09448	0.074796
-0.13282	0.232846	-0.31275	0.329528	0.297838	-0.02208	0.072437	0.180902	-0.09087
-0.205	0.07275	0.103937	0.108043	0.27374	0.028358	0.143571	0.014466	-0.04358
-0.05237	-0.16357	-0.27208	-0.12728	0.288224	-0.32345	0.145721	-0.11454	0.37131
0.004525	0.070168	-0.00855	-0.15289	-0.24461	0.083606	-0.05387	-0.21753	-0.14525
-0.04015	-0.04421	-0.37765	-0.05474	0.107435	-0.06762	-0.07458	-0.19806	-0.07458
-0.36936	-0.32316	-0.57933	0.041502	0.403917	-0.0215	-0.08853	-0.03686	-0.0614
0.049739	0.031614	-0.2954	0.048691	0.15894	-0.16391	-0.16394	-0.23735	0.159691
-0.05841	0.130306	-0.12502	-0.00871	0.206227	-0.03103	-0.19795	-0.27073	0.193825
0.009995	0.111683	-0.07475	0.012614	0.199884	-0.12835	-0.41423	-0.29287	0.130305
0.000341	0.115598	-0.21873	-0.01359	0.296743	-0.15589	-0.19899	-0.19621	0.276795
-0.281	0.078067	0.040626	-0.01847	0.218021	-0.06804	-0.18938	-0.14956	0.137905
-0.09085	0.091077	-0.05802	-0.06727	0.233135	-0.06892	-0.1207	-0.22506	0.249076
-0.03378	0.13735	-0.02797	-0.04574	0.248131	-0.18891	-0.18614	-0.26033	0.196531
-0.11019	0.038781	-0.18827	-0.04334	0.159412	-0.27125	-0.02269	-0.13706	-0.06209
-0.19024	0.240367	-0.2871	-0.11654	-0.05113	0.173782	0.087173	-0.34602	0.265809
0.173575	0.132174	0.058043	0.073438	0.205263	-0.20721	-0.17652	-0.20095	0.305497
0.220243	-0.04237	-0.03246	0.07024	0.279764	-0.15997	-0.01491	-0.00897	0.091443
-0.02553	-0.11352	-0.28989	0.079545	0.297301	-0.21755	-0.2456	-0.09065	0.27104
-0.23358	-0.31206	-0.14871	0.024834	0.224036	-0.35209	-0.09394	-0.07119	0.299294
0.095382	-0.05673	-0.43575	0.038301	0.289809	-0.27507	-0.23503	-0.2616	0.147879
-0.19148	-0.11221	0.029702	-0.15993	0.058128	-0.20634	-0.16736	-0.01533	-0.03078
-0.5532	-0.34802	-0.5923	-0.3576	0.408991	0.024743	0.114331	-0.28227	-0.13609
-0.48646	-0.32129	-0.53356	-0.19996	0.539189	0.18434	0.14772	-0.15277	0.254261
-0.50383	-0.436	-0.66868	-0.20032	0.590178	0.069075	0.210921	-0.24729	0.093071
-0.52284	-0.33528	-0.55452	-0.13459	0.609531	0.117683	0.245462	-0.32244	0.17325
-0.28874	-0.02225	-0.48541	0.132782	0.065609	0.041335	-0.15098	-0.22862	0.162258

TCGA-FB-A	TCGA-FB-A	TCGA-FB-A	TCGA-FB-A	TCGA-FB-A	TCGA-FB-A	TCGA-FB-A	TCGA-FB-A	TCGA-HV-/
-0.09274	0.022882	-0.45179	-0.09575	-0.41322	0.335143	-0.28765	-0.12756	0.042535
-0.05376	-0.01406	0.44826	-0.49804	-0.53508	-0.19445	-0.1713	-0.09518	-0.327
0.397626	0.194822	-0.24746	-0.04349	-0.09175	-0.17299	-0.22307	-0.11965	0.263344
-0.08212	-0.4616	0.272727	-0.4073	-0.50803	-0.14954	-0.12245	-0.23566	-0.27582
0.094232	0.157933	-0.2248	0.051774	-0.30609	-0.14732	-0.41393	0.330214	0.252853
0.213069	0.254118	0.25981	0.057741	0.138417	-0.08683	-0.12128	0.113493	-0.07788
0.268554	0.153036	-0.30904	-0.25483	-0.28756	0.46311	0.059524	0.197383	-0.04714
0.072425	-0.00913	0.375143	0.080918	0.044897	0.009116	-0.08576	0.055962	0.226263
-0.17736	-0.13228	0.411277	0.008405	-0.04031	-0.0455	0.212491	0.176194	0.133388
0.148919	-0.12309	0.541524	-0.07997	-0.1274	-0.22225	0.27139	0.171879	0.194944
0.196245	0.31908	0.552552	-0.17432	0.294936	-0.26198	0.342168	0.439696	0.358011
-0.07494	0.102736	0.567049	-0.08002	-0.01586	-0.21866	0.278425	0.256579	0.244939
0.123428	-0.02536	-0.05717	-0.36455	-0.39937	-0.28489	-0.29107	0.131394	-0.11155
0.048531	0.342395	-0.04213	-0.13416	0.074888	-0.03305	0.00597	0.179747	0.091008
-0.27705	-0.23961	-0.13066	-0.08532	-0.51957	-0.29827	-0.02192	-0.18464	0.299636
-0.37398	-0.47829	-0.37373	-0.39912	-0.63343	-0.06007	0.023705	-0.13333	-0.31035
0.246508	0.000103	-0.00076	-0.38171	-0.10602	-0.00455	-0.12414	0.138739	-0.46404
-0.09555	-0.26358	0.011384	-0.21205	-0.14565	-0.00778	0.124137	-0.02486	0.035753
-0.0264	-0.10878	0.208877	-0.40636	-0.09965	0.229663	0.200742	0.177252	-0.04253
-0.06627	0.575422	-0.30354	-0.27275	0.259644	0.612615	0.099166	0.613016	-0.09824
0.061976	0.352891	-0.31448	0.104692	0.250491	0.103108	0.059974	0.253748	0.0155
-0.10988	0.349387	-0.21982	-0.20708	-0.01546	0.606686	-0.21333	0.232322	-0.02547
0.243822	0.102842	0.171488	-0.09705	0.236575	-0.24809	0.243385	-0.00179	-0.11703
-0.05619	-0.36788	0.336818	-0.11741	-0.47745	-0.22418	-0.1995	-0.27649	-0.12833
0.120468	0.114334	0.212832	-0.17032	0.052004	0.195968	0.382857	0.207206	-0.16973
-0.0149	-0.34065	0.09849	0.033804	-0.30251	-0.11018	-0.35628	-0.3031	0.09974
0.595823	0.207611	-0.22406	0.349175	0.394602	0.197489	-0.62657	-0.3846	0.202425
-0.27196	-0.0689	0.38045	0.246217	0.141759	0.123375	-0.20006	-0.20546	0.224774
0.29847	0.046281	0.206671	0.276093	-0.14774	-0.19568	0.312281	0.012373	-0.31012
-0.1173	-0.07615	-0.21375	0.261578	-0.16788	-0.28958	-0.28416	-0.44821	-0.07504
-0.29164	-0.60284	0.467309	0.074226	-0.58881	-0.62393	-0.10691	-0.4821	-0.40372
-0.4451	-0.26971	0.461487	-0.00471	-0.27759	-0.26361	-0.20563	-0.20069	-0.17898
0.045401	-0.15813	-0.22424	0.135132	-0.05461	-0.09132	-0.26309	-0.28804	0.109248
-0.0569	-0.19636	-0.31521	0.157751	-0.12039	-0.01843	-0.34958	-0.30044	0.155444
-0.12853	-0.11089	-0.21679	0.076425	-0.12081	-0.13855	-0.29342	-0.3679	0.015289
0.276909	0.122801	0.356083	-0.04229	0.085702	0.201349	-0.07025	0.108941	0.127992
0.314695	0.176555	-0.18584	-0.32539	-0.33484	0.367361	-0.08191	0.222567	-0.59009
-0.04949	-0.06343	0.343138	0.001475	0.190106	0.479	-0.28163	-0.05558	0.367899
0.36367	0.327368	0.359656	0.32604	0.341638	-0.18725	0.397807	-0.30089	0.177063
0.487473	0.350561	0.096943	-0.22285	0.344911	0.217941	-0.1616	-0.01092	0.09701
0.222141	-0.04361	0.243066	0.096049	-0.15265	0.329683	-0.29963	-0.29591	0.145635
0.367591	0.268027	0.398034	0.284926	0.352963	-0.08121	0.373669	-0.05103	0.063945
-0.1845	-0.0013	0.331928	-0.14071	-0.2944	-0.18626	0.213914	-0.20976	-0.46374
0.584957	0.617359	0.46719	-0.28115	0.152693	0.226779	0.394881	0.227288	-0.49778
0.188365	0.053706	0.114049	-0.20666	-0.55446	-0.41993	0.629146	-0.07066	-0.52584
0.3758	0.223939	0.333817	0.270506	0.229608	-0.26026	0.495489	0.093709	0.186607
0.355425	0.269641	0.309133	-0.19994	-0.01587	0.356414	0.601273	0.44096	-0.38363
-0.02154	0.022666	-0.07486	-0.16618	-0.04469	0.176502	0.170071	0.206328	-0.29461
-0.19474	0.125216	-0.16061	-0.14716	-0.11763	0.409732	0.220101	0.323194	-0.25231

0.093584	0.185704	0.046442	-0.02378	-0.065	0.088832	0.173095	0.554144	-0.09186
-0.17183	-0.19653	-0.00592	-0.04013	-0.23426	-0.32403	0.009741	-0.01032	-0.36662
0.062577	-0.00127	0.042975	-0.25893	-0.24895	0.240705	0.094218	0.368057	-0.31393
-0.12414	-0.21177	0.251872	-0.40476	-0.24942	0.28748	0.270054	-0.01894	-0.55436
-0.07428	0.215603	-0.0628	-0.24168	-0.04978	-0.03802	-0.4097	-0.00793	-0.43612
-0.07285	0.148464	0.093875	-0.18401	0.11067	-0.07774	0.238825	0.288389	-0.24868
-0.02046	0.312354	0.381671	-0.27039	0.115705	0.028983	0.327834	0.136857	-0.27023
-0.02431	-0.11689	0.33092	-0.15143	-0.18817	0.199831	0.270288	0.10971	-0.18184
0.254991	-0.23332	0.102797	-0.04328	-0.08136	0.30272	-0.13807	0.211661	-0.14113
0.067294	-0.16901	0.313849	-0.18347	-0.34209	-0.30114	0.164531	-0.27273	-0.5493
-0.15206	-0.10194	-0.09065	-0.03631	0.056106	-0.05412	0.059234	-0.01064	-0.51972
0.215942	-0.04581	0.326037	0.272906	-0.20599	-0.62337	0.329425	0.26245	-0.05827
0.034482	0.13422	0.326981	-0.36873	0.028666	-0.00743	0.252648	0.228501	-0.32668
0.010221	0.021604	0.473582	-0.23743	-0.01146	0.020998	0.293969	0.170099	-0.30079
0.294222	0.073391	0.160958	0.377033	-0.00261	0.220432	0.045149	-0.3237	0.276835
0.336501	0.012885	0.140351	0.05998	-0.01657	0.161394	0.091085	0.089425	0.097964
-0.33381	-0.53235	-0.14489	-0.33005	-0.4517	-0.17036	-0.3114	-0.41336	-0.30978
-0.06893	-0.16252	-0.08666	-0.23498	-0.28719	-0.18022	-0.21	-0.26063	0.177948
-0.08674	0.029018	0.001419	-0.02777	-0.06775	-0.32565	-0.50086	-0.16163	0.097085
0.272225	0.21691	0.303733	-0.21997	0.006013	0.066024	0.263935	0.048034	-0.4957
0.439595	0.209718	-0.20372	0.32457	-0.37699	0.199215	-0.14181	0.452801	-0.64341
0.257058	0.14908	0.094846	-0.46601	-0.13504	-0.06771	0.171202	0.22944	-0.64547
0.158465	0.102436	0.274612	-0.26855	0.027772	0.08241	0.365834	0.176644	-0.54437
-0.05694	0.543303	-0.09667	0.264169	0.241027	0.420923	0.234673	0.548986	-0.26039
0.287305	0.191517	-0.33732	0.19737	-0.04348	0.542344	0.486616	0.039603	-0.47811
0.282366	0.259251	0.358611	0.188147	0.179159	-0.24047	0.371826	0.169577	0.313064
0.140219	0.118864	0.307918	0.063005	0.083706	0.137915	0.398435	0.09668	0.010396
0.686975	0.247172	0.426621	-0.6175	0.417193	0.207176	0.343831	0.270696	-0.5958
0.288108	0.218676	0.054705	-0.35847	0.307902	-0.04134	0.44162	0.289521	0.008301
-0.28205	-0.05637	0.559366	-0.38861	-0.16452	-0.20704	0.266085	0.111305	0.416523
0.200871	0.079042	0.532345	0.010897	0.28183	-0.15711	0.416839	0.151228	0.038038
0.152627	0.108308	0.469157	-0.05885	0.282929	-0.16414	0.396027	0.144486	0.04862
0.21405	0.264751	0.28785	0.314384	0.262858	0.259428	0.351061	0.168031	0.309529
-0.01759	0.093522	-0.08927	0.041137	-0.2028	0.262259	0.246936	-0.20828	-0.42369
0.131154	0.052994	-0.1478	-0.21098	-0.17242	-0.20438	-0.19529	-0.08978	0.058965
-0.2812	0.245443	0.433873	0.475709	0.462292	0.525243	0.583397	0.664786	-0.34198
0.155473	0.247327	-0.25005	0.156909	0.440858	0.415322	0.14242	0.367267	-0.20444
-0.45456	-0.09559	-0.13772	-0.41943	-0.1433	0.37472	0.1427	0.57859	-0.24523
-0.38865	0.313736	-0.3859	0.12967	-0.05638	0.617113	0.069171	-0.02786	-0.46113
-0.03112	0.046552	-0.06899	0.328635	0.192705	0.3336	-0.00757	0.25262	-0.18299
-0.4244	0.098742	0.398534	0.369465	-0.03233	0.30843	0.44714	-0.08656	-0.354
0.004909	-0.05942	-0.51293	-0.2767	-0.38728	0.138779	0.224693	0.124827	-0.56316
0.166191	0.258586	0.151648	0.13575	-0.1353	-0.12766	0.103741	-0.15656	-0.12888
-0.37111	0.264366	0.102431	-0.1434	0.427086	0.406357	0.196061	0.383183	0.248064
-0.18756	-0.10089	-0.26616	0.048034	0.056777	0.579968	0.171671	0.118367	-0.29028
-0.48741	0.301662	-0.50701	-0.15805	-0.07167	0.546699	0.029833	-0.21288	-0.48403
-0.26091	0.501978	-0.33457	-0.21385	0.212253	0.430932	0.021317	-0.01539	-0.25409
0.052072	-0.09522	-0.08325	0.146475	-0.14318	-0.18473	-0.2375	-0.2445	0.001561
-0.08293	-0.02348	-0.25759	0.235942	-0.31351	-0.05868	-0.43569	-0.33924	0.02838
-0.22193	-0.08452	-0.01093	0.084107	-0.25692	-0.20368	-0.21595	-0.2115	-0.11329

-0.09653	-0.2812	0.362101	0.283615	-0.28593	-0.42882	0.028945	-0.30044	-0.19225
-0.2904	-0.28268	0.212053	0.194386	-0.42456	-0.37421	-0.30816	-0.45983	-0.28875
-0.20368	0.007937	0.089346	0.024328	-0.14917	-0.19315	-0.50802	-0.26737	0.020393
-0.13549	-0.19785	0.101286	0.012946	-0.27621	-0.13796	-0.13463	-0.21548	-0.19329
-0.10355	0.223693	-0.41189	0.14037	0.044707	0.485529	-0.06983	-0.01636	-0.20094
-0.15508	0.157368	-0.21901	0.134742	0.025994	0.335588	-0.11964	-0.20847	-0.23463
-0.00599	0.253331	-0.11217	0.236825	-0.07108	0.04743	0.003134	0.147967	0.184448
0.036803	0.04927	-0.28781	0.00943	0.000123	0.15358	-0.17209	0.065376	-0.07668
0.108058	-0.14856	-0.21282	0.099839	0.195453	-0.15466	0.031419	-0.29521	0.090087
0.122487	-0.01489	-0.03704	0.223216	-0.14499	-0.2916	-0.35819	-0.22774	0.050918
-0.1921	0.026685	0.302537	-0.40797	-0.07842	0.140911	0.409096	0.145866	0.039243
-0.04021	-0.38142	-0.23385	0.125826	-0.2248	0.015053	-0.38715	-0.24244	-0.05026
-0.09109	0.061225	0.072161	0.248391	-0.16382	-0.24974	-0.29148	-0.21973	-0.03387
-0.28554	-0.14561	0.048548	0.017307	-0.265	-0.31275	-0.26855	-0.13917	-0.25553
-0.17617	-0.0471	-0.21546	0.154776	-0.10559	0.054434	-0.25431	-0.14118	-0.06364
0.052214	0.063799	-0.16615	0.188734	-0.00866	-0.29689	-0.50003	-0.20876	-0.00263
-0.18305	-0.31051	-0.0421	0.081969	-0.10955	-0.27319	-0.37684	0.035084	0.333135
-0.24834	-0.00718	-0.11366	0.133417	-0.16133	-0.02783	-0.35106	-0.11771	0.042247
-0.21855	-0.30618	-0.35355	0.174749	-0.23181	-0.29458	-0.32507	-0.37709	-0.2973
-0.05426	-0.14755	-0.25672	0.317175	-0.25834	0.049424	-0.40434	-0.30854	0.283287
-0.14583	-0.10904	0.102645	0.187215	-0.15622	-0.04472	-0.28612	-0.10318	0.243339
-0.31561	-0.36067	-0.35296	0.260088	-0.3638	-0.15853	-0.46328	-0.41681	0.018654
-0.25998	-0.52279	-0.30163	0.235612	-0.32069	-0.19554	-0.51472	-0.41426	0.137495
-0.20202	-0.41843	0.337712	0.10824	-0.50234	-0.4303	-0.30028	-0.44768	-0.30795
-0.01225	-0.10404	-0.32567	0.267706	-0.18812	-0.00513	-0.47711	-0.37978	-0.09297
0.016519	0.070235	-0.03871	0.331574	0.072863	-0.17928	-0.30565	-0.26749	0.156378
-0.09215	-0.01535	-0.20969	0.130312	-0.2631	-0.31839	-0.43157	-0.34376	-0.29383
-0.03222	-0.45411	0.31814	0.208921	-0.38668	-0.47966	-0.04798	-0.25742	-0.13787
0.135381	-0.30784	0.291735	0.122323	-0.30652	-0.46075	-0.1569	-0.08172	-0.47185
0.086461	-0.1042	0.207724	0.234249	-0.12729	-0.4023	-0.25937	-0.37152	-0.03832
0.17019	-0.14091	0.034565	0.299932	-0.26847	-0.32847	-0.2071	-0.4309	-0.1587
0.130348	-0.01665	0.185743	0.271651	0.252603	-0.22446	0.082814	-0.22762	0.16077
-0.19905	0.012433	0.331493	-0.22345	0.189525	-0.25156	0.010906	0.235454	-0.05433
-0.12282	-0.22489	0.175717	0.365513	-0.07956	-0.30556	-0.28295	-0.24368	-0.08926
-0.21151	-0.43835	0.443742	0.272581	-0.42355	-0.36736	-0.18936	-0.48038	-0.10217
0.072095	-0.16208	0.163353	0.372892	-0.17901	-0.27948	-0.22322	-0.31248	-0.12878
-0.06389	-0.06812	0.011996	0.379065	-0.29608	-0.19842	-0.3781	-0.38186	-0.08108
-0.18871	-0.06348	0.080453	0.284117	-0.32693	-0.25847	-0.38026	-0.36609	-0.14855
-0.23099	-0.03839	0.129195	0.132415	-0.34085	-0.22062	-0.35973	-0.26849	-0.08281
-0.28111	-0.05628	0.110414	0.128081	-0.33913	-0.34252	-0.36565	-0.39447	-0.04621
-0.21946	-0.21176	0.171286	0.291111	-0.38927	-0.27452	-0.34344	-0.44658	-0.05376
-0.13746	-0.44002	0.594463	0.178182	-0.47413	-0.50781	-0.12928	-0.31893	-0.48026
-0.06893	0.095789	-0.09504	0.114793	-0.12564	-0.10092	-0.20249	-0.07017	-0.09908
-0.23221	-0.13926	-0.16312	0.041873	-0.33933	-0.26872	-0.45593	-0.35527	-0.11398
-0.08398	0.02318	-0.04233	0.099024	-0.23274	-0.13687	-0.16598	-0.11626	-0.1188
-0.11366	0.150077	0.312248	-0.00935	0.191894	-0.12723	0.018531	-0.13936	0.138643
-0.25717	-0.24983	-0.2175	0.24539	-0.32802	-0.14034	-0.38766	-0.38777	-0.0377
-0.07227	-0.00455	-0.09139	0.312705	-0.12381	0.012375	-0.39411	-0.29938	-0.06399
-0.17478	0.125418	0.056203	0.116632	-0.11947	-0.17632	-0.29322	-0.17887	0.068677
-0.13109	-0.06841	-0.2823	0.233742	-0.17865	0.225054	-0.27829	-0.29596	-0.20896

-0.28819	-0.09281	-0.01832	0.016992	-0.16108	-0.1494	-0.216	-0.26425	-0.09053
0.175042	0.029143	-0.11153	0.360691	-0.0192	0.005658	-0.29308	-0.06003	0.10569
0.050294	-0.07175	0.003991	0.230016	-0.29836	0.079826	-0.41659	-0.27945	0.005404
0.227476	-0.34213	0.63433	0.213827	-0.48552	-0.62748	0.056715	-0.30534	-0.44606
-0.00709	0.303106	0.436229	-0.01638	-0.03898	-0.16584	-0.00813	0.398064	-0.24869
-0.01297	-0.29168	0.029329	0.357862	-0.2967	-0.20772	-0.33458	-0.25297	-0.07187
0.098992	-0.19229	-0.19924	0.140268	-0.01165	-0.25293	-0.31746	-0.25981	-0.20228
0.203118	0.167503	0.117044	-0.15936	-0.04131	0.180927	0.295236	0.354346	-0.26961
0.261475	0.13954	0.28758	-0.12007	0.009644	0.164567	0.575239	0.380553	-0.37349
0.162982	0.256233	0.100023	0.055653	-0.01522	-0.09921	-0.03762	-0.03689	-0.01871
0.206551	0.236915	0.10296	-0.09437	0.078938	0.117526	0.358959	0.371453	-0.32799
-0.0703	-0.10086	0.13538	0.163212	-0.38171	-0.47072	-0.39553	-0.32103	-0.34642
0.13332	0.203361	-0.03648	-0.13394	-0.32942	0.018422	-0.20848	-0.11092	-0.28403
0.216079	0.045944	0.040217	-0.011	-0.27028	-0.01094	-0.22227	-0.3589	-0.10807
-0.18472	-0.4353	0.484362	0.212943	-0.52554	-0.56711	-0.22148	-0.55871	-0.29089
-0.17819	-0.13752	-0.17235	0.231126	-0.21353	-0.11979	-0.34482	-0.29191	0.038349
-0.3498	-0.17292	-0.35601	0.101016	-0.32669	0.049227	-0.46949	-0.41061	-0.18699
-0.0695	-0.0581	-0.26349	0.264422	-0.3336	-0.21223	-0.42764	-0.3711	-0.01774
-0.14461	-0.10009	-0.27748	0.247635	-0.29115	0.102843	-0.44364	-0.36801	-0.0385
-0.2971	-0.11391	-0.3337	0.208821	-0.34443	0.119895	-0.62579	-0.35679	-0.12047
-0.28946	-0.16343	-0.32632	0.232245	-0.44231	-0.03952	-0.52484	-0.28314	-0.13532
-0.10685	-0.14943	-0.36997	0.199449	-0.28581	-0.0852	-0.47574	-0.29485	-0.11182
-0.17741	0.260813	-0.3227	0.236906	-0.18831	0.299084	-0.47196	-0.25679	0.096575
-0.44879	-0.29441	-0.02456	-0.07695	-0.02465	-0.09874	-0.30599	-0.1721	0.179001
-0.15369	-0.20286	-0.32808	0.218156	-0.36347	-0.14191	-0.3971	-0.3073	-0.14915
0.019719	0.13594	-0.22752	0.302873	-0.17716	-0.0073	-0.51515	-0.19668	0.095309
-0.13378	-0.00216	-0.32335	0.306511	-0.25012	-0.18444	-0.56883	-0.24084	-0.12597
-0.2748	-0.09758	-0.04407	0.282534	-0.25582	-0.21645	-0.56881	-0.35792	-0.15796
-0.12945	-0.18888	-0.28356	0.387563	-0.22952	0.083811	-0.41612	-0.20987	0.174551
-0.25329	-0.03148	-0.15613	0.323395	-0.39869	0.101769	-0.55491	-0.33179	-0.08286
-0.28694	-0.64382	0.669327	-0.21084	-0.59165	-0.575	-0.0943	-0.54353	-0.54985
0.041978	-0.5087	0.627685	0.269527	-0.31493	-0.62343	0.023993	-0.3359	-0.44357
0.059057	-0.45502	0.665117	0.173929	-0.59098	-0.73118	0.077949	-0.38717	-0.49914
0.212763	-0.53913	0.65117	0.335479	-0.56166	-0.73366	0.099586	-0.47153	-0.58119
0.020896	-0.49957	0.296013	0.2	-0.45415	-0.50599	-0.27296	-0.49775	-0.22708

TCGA-HV-/	TCGA-HV-/	TCGA-HV-/	TCGA-HV-/	TCGA-HV-/	TCGA-HZ-7	TCGA-HZ-7	TCGA-HZ-7	TCGA-HZ-7
-0.24186	0.179534	-0.34036	-0.27484	0.111004	0.384835	0.413916	-0.07084	0.237942
0.004416	0.214576	-0.31787	0.236501	-0.24079	0.235501	0.654397	-0.47187	0.38311
-0.05366	-0.19063	-0.35841	-0.41357	0.263716	0.218799	0.320866	0.316319	0.187306
0.294935	0.104844	-0.00959	0.008937	-0.16152	0.415471	0.094863	-0.23864	-0.05786
-0.33249	0.429438	0.027009	-0.09541	0.292985	0.256383	0.043166	-0.1591	-0.30335
0.139384	0.13095	0.15158	-0.09984	0.379628	0.066381	-0.10525	-0.08812	0.347176
0.154477	0.273933	0.259322	0.367781	0.407786	0.387232	0.11741	-0.01164	0.179799
0.084734	0.085684	0.178547	0.077752	0.006147	-0.03309	-0.1407	-0.07147	0.191202
0.154554	-0.0908	0.199446	0.045162	0.03071	0.006047	0.108658	0.351377	0.330202
0.35302	0.093711	0.288832	0.007075	0.087387	-0.24732	-0.10485	-0.06894	0.222763
0.286452	0.02481	0.340635	0.130009	0.336235	0.014741	-0.14695	0.20375	0.416535
0.34509	-0.28837	0.480542	0.098064	0.095163	0.050191	0.035929	0.06807	0.064067
0.188953	0.328932	-0.2078	0.002823	0.075207	0.147535	0.165435	0.094864	0.46556
0.248383	0.189446	0.31186	0.045064	0.311343	0.358617	-0.01363	0.047805	-0.23164
-0.14034	0.171726	-0.02861	0.113441	0.128002	0.060028	0.273402	0.085519	-0.41988
-0.24345	0.383946	-0.02576	-0.11688	-0.05495	0.319318	0.298229	-0.3349	-0.22314
0.325916	0.287845	0.118485	-0.04368	-0.24565	-0.17497	0.318051	0.067589	0.007417
0.141684	0.318519	0.113428	-0.01978	0.046203	-0.03519	-0.05227	-0.0732	0.153219
0.206194	0.121644	0.29778	0.000831	0.174205	0.276071	0.11727	0.179899	0.353116
0.630752	-0.38974	0.056573	0.241487	0.471984	0.356051	-0.05107	0.167058	0.541811
-0.27726	-0.51179	-0.2281	-0.18954	-0.11749	0.191832	-0.09279	-0.00316	0.021616
0.432407	-0.43696	-0.42616	0.079576	0.402833	-0.265	0.137307	0.401178	0.518364
-0.28293	0.253471	0.175187	-0.02109	-0.23902	-0.07963	-0.07687	-0.19518	-0.33458
-0.06849	0.414125	-0.01585	-0.28241	-0.14187	-0.20316	0.415439	-0.25244	-0.28065
-0.08745	-0.0053	0.26201	0.226093	0.131668	-0.00802	-0.28321	-0.06826	0.091354
-0.13481	-0.11978	0.129804	-0.19065	0.096827	0.260276	-0.28725	0.175224	0.343403
0.248926	-0.34774	-0.44502	-0.56581	-0.19427	-0.41442	-0.1672	0.116596	-0.4169
-0.18668	0.137237	-0.23256	-0.35951	-0.18366	-0.24004	-0.25123	-0.00062	-0.12062
-0.0635	0.591112	-0.19291	0.257936	0.421117	-0.23283	0.26621	0.047787	0.467181
0.090192	0.275437	-0.15195	-0.455	-0.13171	-0.20872	-0.11895	0.446395	-0.05247
-0.14311	0.324369	0.228226	-0.18628	-0.3015	-0.60306	0.674519	0.211466	-0.2444
-0.00646	0.426792	0.51863	-0.19065	-0.19495	-0.49004	0.499417	0.105036	0.053535
0.099883	-0.3186	-0.27429	-0.39976	-0.31218	-0.19132	-0.05954	0.288293	0.041608
0.164824	-0.33994	-0.27266	-0.30481	-0.28491	-0.15647	-0.09073	0.283048	0.040014
0.109023	-0.20161	-0.29876	-0.38867	-0.34936	-0.21649	0.039607	0.259384	0.008639
0.138206	0.391271	0.163188	-0.22056	0.302913	-0.00505	0.023588	0.227625	0.418463
-0.07431	0.43905	-0.19946	-0.20929	0.293887	-0.11372	0.408596	0.198423	0.714977
0.034401	0.158805	0.354461	0.102816	0.424821	0.151488	-0.02764	0.083248	0.296007
0.197958	-0.36758	0.24765	0.325048	0.289686	0.253715	0.075963	0.463766	0.439288
0.093744	0.385629	0.276463	-0.21817	0.165542	0.074459	-0.10436	0.262495	0.362655
0.095475	0.358426	0.357682	-0.35124	0.020048	0.00297	0.045747	0.238926	0.225553
0.465674	0.15513	-0.07463	0.085713	0.467473	0.326134	-0.03613	0.547387	0.575136
0.085454	0.398527	-0.08539	0.04406	0.049331	-0.25022	0.274157	-0.03301	0.657198
-0.41431	-0.19883	0.313249	0.031097	0.730541	0.10142	0.274422	0.152224	-0.43645
-0.4121	0.445001	-0.37895	-0.14631	0.198064	0.036942	0.412015	0.004157	0.573026
0.228234	0.107206	0.207304	0.073246	0.264815	0.297421	-0.17976	0.422249	0.295397
-0.31927	0.3093	0.46528	0.323981	0.302725	0.176283	-0.30128	-0.44939	0.051747
-0.07465	0.056581	0.12277	0.15714	-0.10873	0.051212	0.058375	-0.04085	0.119993
-0.08975	-0.05713	0.355975	0.297171	0.148847	0.204466	-0.07088	0.058948	0.028756

0.099642	0.35467	0.06126	0.391587	0.247233	0.384447	0.095985	0.025262	0.320323
-0.30717	0.318879	-0.05772	0.211821	-0.21632	0.306228	0.342405	-0.08334	0.156847
-0.20474	0.283831	-0.14668	0.005374	0.1199	0.138779	0.277013	0.004588	0.309248
0.055802	0.459933	-0.0306	-0.07907	0.353568	-0.12164	0.208603	-0.02975	0.600309
0.046206	0.203793	-0.3199	-0.16397	0.031346	-0.02514	0.439192	0.092243	0.408358
0.105245	0.399871	0.073807	0.197787	0.24747	0.180995	0.124321	0.019046	0.351496
0.053773	0.184397	0.273988	0.181086	0.200615	-0.01956	-0.06916	0.034101	0.305988
-0.11594	0.157816	0.267352	0.280259	0.038891	0.038178	-0.00968	-0.2213	0.197981
-0.34317	0.473229	0.295886	0.087049	0.088061	-0.28982	-0.01858	-0.13368	0.175508
0.083785	0.393174	0.098052	-0.12191	0.279209	-0.1608	0.459867	0.033088	0.384835
-0.31673	0.408449	-0.17707	-0.05946	-0.09662	-0.23262	0.446898	0.131909	0.549722
0.450442	0.732706	-0.12322	0.258262	0.078185	-0.61881	-0.03403	-0.2719	-0.15444
0.200298	0.272959	-0.17604	0.052574	0.207571	-0.03313	0.160278	0.016759	0.103409
0.049015	0.32349	0.009614	-0.20023	0.270358	-0.33798	0.270382	-0.0248	0.401111
0.18699	-0.02538	0.071969	-0.14578	0.241642	0.177705	0.037914	0.437251	0.389953
-0.20448	-0.03196	0.261867	0.018693	0.219976	0.149013	0.306296	0.176318	-0.03633
-0.07729	-0.13783	-0.23895	-0.25814	-0.35677	-0.28559	-0.25827	-0.40352	-0.60114
0.065519	0.151098	-0.00846	0.074451	0.118851	0.13525	-0.09016	-0.11823	-0.27242
0.182764	0.19763	-0.36923	-0.21514	-0.10133	0.009716	0.123807	0.084143	0.294572
-0.11473	0.366685	0.186137	0.072827	0.266957	-0.21057	0.015831	0.042287	0.234803
-0.43657	-0.09805	0.187063	0.025464	0.109239	0.42328	0.136594	-0.381	0.031523
-0.19464	0.467492	-0.26677	-0.04916	0.324719	-0.08874	0.472653	-0.00199	0.595785
0.115957	0.382821	0.313307	-0.02861	0.288393	-0.03843	0.197007	0.089886	0.486948
0.020546	-0.15583	0.155472	0.493479	0.224482	0.10232	0.137183	0.197641	0.339809
0.002791	0.243301	0.515945	0.310804	0.292432	0.538588	-0.34463	0.143237	0.162469
0.30476	0.012706	0.107051	0.151282	0.303693	0.317664	-0.18994	0.323221	0.537621
0.192199	0.179253	0.411309	0.231158	0.25513	0.250422	-0.16201	0.220693	0.309486
-0.36254	-0.33149	0.326258	0.295888	0.655791	0.274776	-0.27573	0.202246	-0.16107
0.048219	-0.02624	-0.18268	0.334683	0.299195	0.20456	-0.01889	0.096049	0.372927
0.295658	0.237663	-0.18305	-0.27222	-0.13623	-0.35924	0.158779	0.247878	0.444811
0.215057	0.14308	0.161008	0.122608	0.330753	-0.15183	-0.05463	0.292797	0.531639
0.202256	0.073496	0.084143	0.082129	0.299409	-0.14963	0.117814	0.309064	0.562397
0.203871	0.018494	0.266687	0.250691	0.340897	0.282058	-0.25314	0.348197	0.32455
-0.02979	-0.2848	0.166264	-0.20159	0.125249	0.239405	0.166679	0.23371	0.238477
0.158168	0.280652	-0.05557	-0.14231	-0.0394	0.054441	-0.06033	0.058311	0.252645
-0.61548	-0.67513	0.419345	0.679581	-0.68758	0.094611	-0.38256	-0.58536	-0.40712
-0.29877	-0.41217	0.123256	0.171275	-0.08998	0.322413	0.03081	0.099567	-0.15443
-0.40068	-0.23337	0.330244	0.584061	-0.43278	0.180195	-0.21441	-0.28945	-0.20613
-0.35069	-0.60758	0.490956	0.414018	0.218451	0.250871	0.034985	0.108547	0.286084
-0.2212	-0.39396	0.009784	-0.13944	-0.28107	-0.26621	-0.17885	0.011527	-0.06911
-0.56965	0.116162	0.400381	0.009773	0.186082	0.322899	0.32678	0.184686	-0.35862
-0.53715	-0.18161	-0.31256	-0.37511	-0.26017	0.392296	0.588816	-0.28651	0.043051
-0.04278	0.130678	-0.05634	-0.07575	0.058719	0.204765	0.100586	0.181145	0.433447
0.315648	-0.3507	0.552786	0.25353	0.267875	0.097623	-0.34243	0.02862	0.18199
-0.39079	-0.43187	0.134814	0.20446	-0.0471	0.033506	-0.13484	0.095747	0.017367
-0.31734	-0.60365	0.261946	0.076022	0.142661	0.31155	0.109917	0.351916	0.399226
-0.16771	-0.62798	0.355327	0.281993	0.427413	0.376831	-0.10774	-0.16105	0.157973
-0.1074	-0.11227	-0.1871	-0.25343	-0.23228	-0.19733	0.087515	0.186911	-0.0749
-0.1066	0.104564	-0.3456	-0.37648	-0.23517	0.005423	0.281813	0.271033	0.239837
-0.05825	0.110745	-0.15579	-0.1877	-0.09995	0.075635	0.106946	0.110578	0.257832

-0.07647	0.092559	0.21477	-0.3145	-0.29429	-0.2908	0.298355	0.007024	-0.12602
-0.22343	0.289565	-0.04677	-0.36831	-0.31949	-0.28136	0.334593	0.061566	0.187501
0.125782	0.152562	-0.20165	-0.21414	-0.10931	0.01319	0.135326	0.091672	0.299822
-0.05899	0.150974	-0.03088	-0.06614	-0.14453	-0.18839	0.108982	0.019619	0.080224
-0.18671	-0.37396	-0.03772	-0.21039	0.167461	0.150242	0.008439	0.353321	0.222554
-0.22915	0.076677	-0.12927	-0.32671	-0.07265	-0.02521	0.156209	0.347175	0.243848
0.226262	-0.35405	0.055211	-0.17942	-0.00325	-0.03226	-0.00982	0.26081	-0.18726
-0.19161	-0.21802	-0.25892	-0.25885	-0.07925	0.058437	0.250061	0.196689	-0.00747
0.055966	-0.05073	-0.27301	-0.20811	-0.33808	0.083133	0.029072	0.068476	0.354041
0.027737	0.089306	-0.16769	-0.25957	-0.0328	-0.22876	0.036351	0.178981	-0.01126
0.069443	0.046197	0.121712	0.082248	0.30301	0.226806	0.058032	-0.00919	0.403505
-0.17182	0.102893	-0.2556	-0.26768	-0.11469	0.03601	0.127875	0.118617	0.197015
0.006663	0.104569	-0.13216	-0.30669	-0.16639	-0.20736	0.279533	0.322395	0.055051
0.035282	0.221339	-0.19807	-0.36647	-0.35828	-0.22405	0.13841	0.132151	0.300729
-0.0897	-0.06236	-0.26297	-0.23338	-0.02879	-0.08141	-0.027	0.175781	0.051551
-0.03421	0.357456	-0.41727	-0.49459	-0.25503	-0.25686	0.346928	0.19131	0.152832
0.460858	0.315112	-0.24403	-0.33679	0.027323	-0.33445	-0.13902	-0.12184	-0.22946
0.182865	0.068082	-0.26387	-0.39567	0.087954	-0.22423	-0.10322	0.240189	-0.15434
0.048489	-0.06612	-0.47411	-0.54734	-0.33352	-0.37797	0.240425	0.333221	-0.16526
-0.07257	0.130433	-0.2215	-0.39703	-0.04384	-0.21993	-0.03109	0.272629	-0.01164
-0.06441	0.14523	0.016271	-0.18084	-0.08477	0.060026	0.030338	0.201321	0.075003
-0.00471	0.096104	-0.41655	-0.51758	-0.38279	-0.31498	0.126731	0.269408	0.09545
0.28301	0.042732	-0.43305	-0.53708	-0.32075	-0.37063	-0.0042	0.102432	-0.10494
-0.16109	0.373782	0.057733	-0.41202	-0.24212	-0.4538	0.428943	0.184231	-0.02377
-0.15717	-0.0727	-0.40906	-0.38888	-0.23669	-0.1644	0.077726	0.397751	0.233908
0.124703	0.150874	-0.19915	-0.31644	-0.06422	-0.18001	-0.13652	0.295462	0.114501
-0.07468	0.203584	-0.36119	-0.43575	-0.1892	-0.00357	0.193815	0.308663	0.246929
-0.16356	0.425331	-0.19445	-0.20123	-0.40478	-0.37073	0.308367	0.162827	0.04789
0.096028	0.25444	0.123579	-0.37043	0.272166	-0.38252	0.55153	0.207081	0.036037
0.025394	0.139899	-0.07756	-0.36911	-0.34852	-0.14234	0.335393	0.209786	-0.09915
-0.28745	-0.05214	-0.14752	-0.37498	-0.31739	-0.26708	0.282306	-0.11444	0.052673
0.025519	0.047972	0.043553	-0.12994	-0.04021	0.047636	0.050419	0.03885	-0.20397
0.140349	-0.00511	0.252821	0.181537	0.003759	-0.05374	0.070801	-0.20127	-0.11658
0.020148	0.048146	-0.09881	-0.32514	-0.25658	-0.22358	0.270449	0.149556	-0.04511
-0.27914	0.05942	0.217416	-0.39336	-0.41692	-0.35993	0.317026	0.174958	0.061967
-0.0784	0.08635	0.103021	-0.31683	-0.23053	-0.17323	0.424843	0.250439	0.07521
-0.3444	0.200804	-0.14981	-0.37357	-0.34395	-0.25383	0.400427	0.324029	0.097496
-0.25814	0.279126	0.065372	-0.38122	-0.24669	-0.23797	0.305303	0.245117	0.102683
-0.1706	0.022023	-0.0568	-0.25315	-0.24747	-0.00579	0.335183	0.335657	0.285127
0.073721	0.252892	-0.04566	-0.34572	-0.14554	-0.31062	0.16461	0.269925	0.130596
-0.11806	0.309266	-0.14367	-0.33739	-0.29905	-0.37313	0.125783	0.259208	0.08191
-0.16708	0.37389	0.425518	-0.14595	-0.20792	-0.53127	0.602601	0.255175	0.196272
-0.10897	0.186695	-0.26629	-0.30748	-0.24965	0.18058	0.242537	0.370537	0.405455
-0.16248	0.140975	-0.32275	-0.46549	-0.28605	-0.00244	0.311315	0.29619	0.212533
-0.09481	0.168333	-0.09234	-0.36696	-0.12082	0.02349	0.130442	0.247197	0.243143
0.161727	0.113893	0.036352	0.478059	0.220529	0.078115	0.208746	0.116146	0.193887
-0.0488	0.052457	-0.2489	-0.34761	-0.22242	-0.28963	0.162966	0.241017	0.036242
-0.15244	0.046656	-0.2345	-0.23454	-0.21241	-0.02646	0.162107	0.081186	0.248445
0.068301	0.059172	-0.13937	-0.29007	-0.12692	-0.02032	0.110061	0.225697	0.347667
-0.29509	-0.01473	-0.23001	-0.27029	-0.30565	0.115918	0.12984	0.302251	0.182869

0.120495	0.100769	-0.21817	-0.33387	-0.05498	-0.01079	0.010208	0.14593	0.185545
-0.09848	0.112664	-0.28342	-0.24276	-0.15306	0.069316	-0.02188	0.110779	0.175373
-7.67E-05	0.132233	-0.33358	-0.1954	-0.14401	0.152061	0.320108	0.354548	0.231771
0.021574	0.396696	0.406982	-0.25821	0.157068	-0.42383	0.600816	0.243965	-0.16611
0.386753	0.721774	0.043762	0.134605	0.227337	0.102045	0.264156	0.096461	0.386214
-0.27622	0.270427	-0.26306	-0.14445	-0.01123	0.256104	0.401196	0.254618	0.260907
-0.15844	0.288214	-0.269	-0.44571	-0.30979	-0.29768	0.11861	0.274602	0.204285
-0.13165	0.134333	0.28822	0.139515	0.295367	0.219676	-0.13423	-0.12675	0.278563
-0.19605	0.317332	0.402092	0.22117	0.385547	0.188336	-0.30991	-0.40562	0.332004
0.091159	0.22309	0.044152	0.02352	-0.14187	0.180981	0.13933	0.267957	0.160082
-0.13873	0.162182	0.308554	0.236951	0.248276	0.212317	-0.22597	-0.18184	0.288437
-0.22266	0.28577	-0.26372	-0.27802	-0.41355	-0.4027	0.446461	-0.1043	-0.04876
-0.3192	0.440661	-0.08424	-0.25796	0.070264	0.147677	0.299852	-0.0665	-0.07829
-0.30204	0.18561	-0.2012	-0.34167	-0.19274	-0.15494	0.147745	0.134148	-0.1063
-0.04823	0.277906	0.017242	-0.42294	-0.36742	-0.48785	0.541597	0.151497	-0.29093
0.048613	0.000864	-0.25054	-0.34008	-0.15351	-0.19048	0.05729	0.230605	0.022244
-0.0595	-0.00566	-0.26036	-0.25818	-0.39879	0.045962	0.205705	0.279289	0.184516
-0.21648	0.300165	-0.41995	-0.39212	-0.30715	-0.05399	0.316201	0.357521	0.2974
-0.17764	0.072235	-0.34784	-0.3863	-0.22693	-0.11068	0.239354	0.303741	0.150347
-0.12251	0.157532	-0.35424	-0.30225	-0.29399	0.096586	0.272363	0.201864	0.259995
-0.194	0.120213	-0.36523	-0.43229	-0.21804	0.033302	0.33303	0.245445	0.255664
-0.11472	0.035626	-0.38876	-0.45156	-0.20036	-0.07122	0.28973	0.228189	0.186219
-0.01743	-0.0708	-0.273	-0.36213	-0.09943	0.055789	0.062604	0.271713	0.342224
0.319017	0.083324	-0.15909	-0.25939	0.069892	-0.08879	-0.26075	-0.03766	-0.27141
-0.18296	-0.02808	-0.31528	-0.25035	-0.21325	-0.08214	0.237974	0.129879	-0.06751
-0.03359	0.087718	-0.3588	-0.35994	-0.1289	0.128055	0.123316	-0.04213	0.083177
-0.01675	0.169691	-0.38218	-0.38699	-0.33162	-0.14604	0.221991	0.247151	0.130231
-0.09277	0.149102	-0.20029	-0.40026	-0.36605	-0.21328	0.109176	0.090053	0.078843
0.032662	0.016785	-0.35315	-0.41377	-0.27206	-0.16941	0.144141	0.221999	0.027927
-0.25363	0.339632	-0.26245	-0.34221	-0.20152	-0.03905	0.284675	0.075029	0.260631
0.067513	0.153455	0.249259	-0.17937	-0.11419	-0.61135	0.693855	0.372505	0.006004
-0.03989	0.317782	0.392262	-0.06821	0.14835	-0.57518	0.602341	0.36739	-0.09919
0.012199	0.387	0.462949	-0.21836	0.147021	-0.57071	0.659911	0.272491	-0.20987
0.009359	0.169791	0.402902	-0.36225	0.048381	-0.51013	0.702018	0.330361	-0.08372
0.219362	0.192666	0.124515	-0.4257	0.056334	-0.42041	0.383425	0.257754	-0.05295

TCGA-HZ-7	TCGA-HZ-8	TCGA-HZ-8	TCGA-HZ-8	TCGA-HZ-A	TCGA-HZ-A	TCGA-HZ-A	TCGA-HZ-A	TCGA-HZ-A
0.214636	-0.19428	0.08714	0.316972	-0.18689	0.223892	-0.03627	-0.13567	-0.22769
-0.19657	-0.11467	0.536291	0.279011	0.659584	-0.18461	0.236319	-0.38602	0.080328
0.452042	0.219438	-0.41791	0.252523	-0.09789	0.168625	0.075727	0.137554	0.286529
0.048987	-0.24027	0.112322	-0.18283	0.234962	-0.02477	0.21541	-0.03679	-0.19386
-0.04993	-0.49765	-0.37718	-0.29833	-0.02667	0.290845	-0.04562	0.250409	0.301281
0.080895	-0.22536	-0.02462	0.181272	0.239514	0.171908	-0.03074	0.189225	0.196056
0.312985	-0.4231	-0.31938	0.363132	-0.17877	0.074787	0.171405	-0.39984	-0.00077
-0.04969	-0.28314	-0.00552	0.159589	0.302078	0.122562	0.087455	0.0699	0.181971
0.048153	0.163152	0.116101	0.300035	0.389832	0.248229	0.2375	0.326621	0.019541
-0.04806	-0.06435	0.033041	0.253176	0.438851	0.324721	0.303643	0.155197	0.330264
0.164631	0.106446	0.012779	0.527062	0.378995	0.350146	0.488834	0.389535	0.092775
-0.23986	-0.0812	0.060469	0.321156	0.44593	0.341472	0.270482	0.380117	-0.2314
0.264453	0.034426	0.092426	0.240632	0.177507	-0.19929	-0.10109	-0.13962	0.187791
-0.0657	-0.24095	-0.35478	-0.17203	0.168036	0.220051	0.089898	0.025371	0.110427
0.143767	0.192949	-0.2817	-0.31279	0.413412	0.039621	-0.16892	-0.14293	0.385592
-0.0568	-0.38128	0.012298	-0.19536	0.031394	0.212544	-0.41143	0.033543	0.074798
-0.11731	0.131053	0.405509	-0.23214	-0.1228	-0.23689	-0.48915	0.198098	-0.19713
-0.00687	0.136521	-0.1272	0.317504	-0.04058	0.462498	0.086891	-0.21673	-0.02998
-0.12131	-0.05176	0.296957	0.42727	-0.19444	-0.14835	-0.05045	-0.37577	-0.20523
-0.18126	-0.58474	0.137633	0.251133	-0.49591	-0.18832	-0.03946	-0.38856	-0.46205
0.070661	0.199154	-0.13722	-0.23324	-0.43531	-0.12401	-0.13093	0.092517	-0.35266
0.152909	0.227665	0.555103	0.446054	-0.06285	-0.32388	0.055717	0.133783	-0.25686
-0.30871	-0.26511	0.112281	-0.02405	-0.09862	0.249826	0.061707	0.211968	-0.20055
-0.2471	0.251727	0.336554	-0.30783	0.48833	-0.23886	0.294024	-0.08308	0.311672
-0.22935	-0.18481	-0.24679	0.125288	-0.08762	0.028808	-0.15985	-0.12359	0.06822
0.274077	-0.3108	-0.09746	-0.05321	0.468934	0.545919	-0.1271	-0.2777	0.49617
0.282579	0.473868	-0.50258	-0.51394	0.434844	-0.24353	0.219494	0.51008	-0.06976
0.080107	0.320974	0.162706	0.236364	0.280326	0.333646	0.245962	0.130518	0.320917
0.441248	-0.00274	-0.08282	0.450957	0.427072	0.456962	-0.21729	-0.31799	0.254508
0.39972	0.284628	0.144685	-0.242	0.013272	-0.06509	-0.00724	0.565159	-0.0045
-0.20886	0.403391	0.670023	-0.1489	0.461202	0.535609	0.179475	0.188081	0.212007
-0.02076	0.189102	0.699822	-0.06484	0.307258	0.444211	0.143393	0.53049	0.450889
0.27203	0.165858	-0.25557	-0.04333	-0.03621	-0.04301	0.234072	0.331548	-0.02052
0.249101	0.152939	-0.30332	-0.26144	-0.05116	-0.17695	0.359664	0.349483	-0.07815
0.188708	0.133537	-0.26233	-0.07438	0.073422	-0.01326	0.273755	0.368377	-0.02538
0.155995	-0.01225	-0.06049	0.415741	0.332877	-0.07462	0.077782	0.035313	0.107285
-0.08796	-0.43159	0.487239	0.521926	0.121655	-0.033	-0.39603	-0.20727	-0.269
-0.19191	-0.49741	-0.30222	0.190224	0.334358	0.059369	0.372889	-0.31639	0.110792
0.321886	0.270926	0.304282	0.437182	-0.01653	0.262056	-0.18307	-0.35433	-0.12981
0.041932	-0.38284	-0.01721	0.238883	0.262707	-0.06402	0.065656	-0.24448	-0.0117
-0.0112	0.015883	-0.02461	0.134121	0.488861	-0.02148	0.113728	-0.17759	0.205159
0.614555	0.469271	0.159967	0.613444	0.14108	0.298236	-0.13929	-0.30618	-0.04369
0.384366	0.196878	-0.03129	0.642023	0.561281	0.486945	-0.22724	-0.21116	0.112847
-0.22794	-0.38982	-0.14384	0.313122	0.255589	-0.04668	0.193463	0.498436	-0.44652
0.39989	0.12352	0.058062	0.464307	0.524462	0.447523	-0.15598	0.112469	0.460224
0.398671	0.313445	0.082116	0.361025	0.122261	0.154114	0.005183	0.068347	0.223681
-0.44412	-0.28423	0.179908	0.316532	-0.30203	0.024757	-0.33954	-0.52121	-0.06757
-0.08415	-0.27851	-0.07929	0.022131	-0.16643	0.142408	-0.1545	-0.27462	-0.18815
-0.07493	-0.41113	-0.02984	0.100893	-0.4385	0.107204	-0.20024	-0.31293	-0.16419

0.35989	-0.2419	-0.1201	0.186972	-0.05758	0.192132	-0.1502	0.005698	-5.44E-05
0.134956	-0.26799	-0.10368	0.181029	0.4285	0.490706	0.193407	-0.20101	-0.34682
0.353838	-0.0443	-0.12486	0.266325	0.063084	-0.17765	-0.20577	-0.07049	0.035718
-0.07252	-0.18303	0.043644	0.621226	0.483497	0.443939	-0.32661	-0.43972	-0.20186
-0.07774	-0.17504	0.155688	0.403839	0.253956	-0.32747	-0.37336	-0.13638	0.101354
0.102794	-0.14232	-0.20979	0.366282	0.057233	0.298422	-0.04386	-0.22654	-0.11404
0.25661	-0.04285	-0.22916	0.190671	0.088053	0.291635	0.218449	-0.2476	-0.11359
0.111352	-0.00804	-0.36771	0.159335	0.201223	0.323918	0.214356	-0.33724	-0.01424
0.025482	-0.21337	-0.32973	0.172339	0.32895	0.217871	0.273864	-0.32393	0.12844
-0.04375	-0.15358	0.149804	0.20076	0.339442	0.453348	0.003384	0.141707	-0.02766
0.320838	0.218282	0.016952	0.600323	0.387876	0.25742	-0.03014	-0.33127	0.099271
-0.02647	0.231772	-0.43791	-0.13288	0.637704	0.211976	0.481877	0.169724	0.593228
-0.08952	-0.23586	-0.13332	0.114585	0.237023	0.314305	-0.09027	-0.43182	-0.24721
0.191501	-0.25969	0.159885	0.4262	0.219194	0.389084	-0.01096	-0.19461	0.156144
0.238952	0.289391	-0.0071	0.34442	0.316995	0.058627	0.036005	-0.07619	-0.05176
-0.17857	-0.01315	0.094287	0.075572	-0.06865	0.234599	-0.15893	-4.65E-05	-0.2234
-0.42536	-0.07419	-0.47512	-0.63927	0.051192	0.17118	0.369852	0.08583	0.09816
-0.20202	-0.144	-0.32695	-0.32477	-0.09883	-0.05556	-0.10946	-0.26981	0.01972
0.16148	0.07204	0.019283	0.167154	0.333548	-0.35455	0.106203	0.234121	0.243734
-0.19399	-0.30624	0.015418	0.372878	0.215509	0.303233	-0.21007	-0.17276	-0.20005
-0.14892	-0.5448	0.326481	-0.04379	-0.2528	0.21324	-0.24192	-0.38768	-0.56309
0.024302	-0.10722	-0.23423	0.611257	0.410566	0.447043	-0.36379	-0.15777	-0.08785
-0.15999	-0.13155	-0.17555	0.527703	0.251026	0.423359	-0.30258	-0.32679	-0.07195
-0.11932	-0.3912	0.10312	0.119938	-0.25144	0.161626	0.020109	-0.24466	-0.29167
-0.1986	-0.40556	-0.09105	0.131656	-0.48024	0.300866	-0.51244	0.209241	-0.44617
0.54741	0.284318	-0.08944	0.567632	0.147822	0.087833	0.217872	-0.00885	0.218797
0.218606	-0.19403	0.179257	0.341068	-0.30399	0.33366	-0.01974	-0.38291	-0.08744
-0.53157	-0.47982	-0.02162	0.583827	0.015585	0.241349	-0.08327	0.630728	-0.71544
0.394771	0.008387	-0.17932	0.256326	0.054744	0.414996	0.44258	-0.00208	-0.14821
-0.28928	0.289762	0.320667	0.365662	0.330578	0.483327	0.32989	0.295199	0.211502
0.487004	0.276791	-0.03601	0.495857	0.166175	0.337064	0.182507	-0.20417	0.20225
0.425539	0.300634	-0.00896	0.483917	0.257452	0.358702	0.183035	-0.22304	0.164773
0.39661	-0.12153	0.062206	0.337558	-0.01224	0.01425	0.099452	-0.1711	0.164267
0.127327	-0.10468	-0.31321	0.111004	-0.19863	-0.32457	-0.32559	0.141153	-0.03684
0.087339	-0.02283	0.11556	0.207398	0.253319	0.26593	-0.1821	-0.03478	0.220155
-0.56361	-0.43477	-0.2573	-0.554	-0.60728	0.434571	-0.70169	-0.6458	-0.6068
-0.04881	-0.15294	0.228551	-0.08833	-0.32209	0.064012	-0.44372	-0.32655	-0.50344
-0.28876	-0.47815	-0.01915	-0.40021	-0.57158	0.08522	-0.27446	-0.58533	-0.50253
-0.42124	-0.60672	0.492615	0.404249	-0.55518	-0.08577	-0.56098	0.029373	-0.38978
-0.23116	-0.04045	0.309866	-0.11343	-0.32551	0.007415	-0.35435	-0.15819	-0.21777
-0.24376	-0.32632	0.532627	0.091755	-0.64067	-0.26316	-0.46576	0.373261	0.133084
0.107535	-0.00397	0.139582	0.461624	-0.51344	0.529377	-0.57466	-0.20121	-0.59267
0.225048	-0.09774	0.140173	0.259364	0.366727	0.22619	-0.23917	0.202629	0.076086
-0.42228	-0.33986	0.219566	0.203082	-0.25491	-0.14484	-0.19642	-0.00423	-0.02397
-0.19019	-0.26768	0.111121	0.159601	-0.45826	0.006695	-0.45413	-0.02427	-0.39522
-0.10428	-0.50887	0.536226	0.565709	-0.48202	-0.00842	-0.60084	0.019326	-0.53347
-0.19552	-0.5377	0.463934	0.330362	-0.503	-0.44639	-0.27748	-0.15076	-0.44751
0.199249	0.151415	0.041609	-0.10761	0.152114	0.152295	0.156031	0.32077	-0.06924
0.328971	0.227766	-0.0259	0.1526	0.276941	-0.00658	-0.07987	0.278189	0.079872
0.077025	0.097672	0.061883	0.085667	0.155224	0.15126	0.009642	0.29893	0.059417

0.250368	0.296251	0.364323	0.049812	0.389134	0.40403	0.283588	0.336599	0.108793
0.253247	0.262506	0.407853	0.055752	0.399108	0.253186	0.150533	0.36262	0.227034
0.137572	0.050335	0.095279	0.178275	0.216028	-0.28151	0.04819	0.330254	0.170387
0.009649	-0.03738	0.064265	-0.05032	0.277901	0.347073	0.235467	0.273362	0.161209
0.091364	-0.17466	0.221693	0.157472	-0.36677	-0.3002	-0.36868	0.299829	-0.32865
0.153463	0.038113	0.110881	0.295432	-0.05756	-0.24718	-0.37483	0.272597	-0.15331
0.002456	-0.02927	-0.01772	-0.10942	-0.1589	-0.04274	0.225431	0.254874	0.00052
0.162355	0.119136	0.16681	0.002968	-0.31331	-0.30078	-0.34798	-0.0462	-0.22559
0.109805	0.069836	0.300304	0.44102	-0.19318	0.153397	0.159572	0.08166	0.252968
0.194614	0.117787	-0.05806	-0.04172	0.073497	-0.23199	0.022882	0.216154	0.2069
-0.04362	-0.15261	0.054746	0.369064	0.03542	0.292751	0.086458	-0.28787	0.132269
0.052272	0.053748	0.042262	0.020771	0.122527	0.058813	-0.23048	0.025155	0.099215
0.336406	0.126728	0.270875	0.259628	0.229391	0.146085	0.249377	0.339134	0.061337
-0.01402	0.17732	0.044623	0.237696	0.30045	0.156422	-0.03298	0.332832	-0.01295
0.260948	0.255829	-0.18681	-0.21674	-0.15204	-0.18786	-0.0022	0.303819	-0.16228
0.395315	0.279167	0.102004	0.019922	0.417946	-0.28614	-0.00929	0.164023	0.104498
-0.21372	0.165652	-0.14352	-0.35827	0.389611	-0.44209	0.340039	0.38845	0.440174
0.053861	0.310256	-0.21853	-0.34423	0.058458	-0.28082	0.26366	0.3308	0.312316
0.2138	0.43072	-0.25622	-0.21326	0.273492	-0.04255	0.042625	0.345414	-0.38563
0.323227	0.311015	-0.02413	-0.21624	0.153179	-0.10354	0.182522	0.423569	0.017909
0.191912	0.107604	0.038415	0.06743	0.329346	0.057187	0.165777	0.35577	-0.06177
0.315122	0.295958	-0.07618	-0.09439	0.283687	-0.14107	0.033591	0.398035	-0.03631
0.250972	0.126906	-0.09053	-0.19615	0.291145	-0.15605	0.27267	0.432877	-0.02392
0.244865	0.271853	0.488212	-0.04473	0.393841	0.276194	0.257128	0.467868	0.260491
0.495971	0.406524	-0.18034	-0.17115	0.027953	-0.41801	0.065809	0.373759	-0.21769
0.358985	0.239256	-0.26958	-0.01613	0.056331	-0.21563	0.065377	0.342507	0.047591
0.255881	0.080958	-0.10878	0.05289	0.333475	-0.01836	-0.01148	0.321502	0.193238
0.35424	0.21235	0.102572	0.052875	0.443638	0.38603	0.397714	0.213626	0.411358
0.021985	0.248598	0.650395	0.299047	0.177043	0.043583	0.169945	0.319231	0.324277
0.199555	0.161507	0.48043	0.147468	0.418287	0.226042	0.058092	0.267462	0.011334
0.290035	0.308537	0.500695	0.361534	0.370142	0.196922	0.115814	0.42412	-0.02393
-0.04412	0.010698	0.38092	0.225321	0.09833	0.018745	0.168051	0.444645	0.093548
-0.01038	-0.11657	0.454513	0.203062	0.009393	0.273215	0.095977	0.17786	-0.21362
0.260084	0.292809	0.386564	-0.03116	0.409388	0.233812	0.229027	0.42728	0.206101
0.243557	0.227752	0.475083	-0.15824	0.416798	0.453115	0.373794	0.352857	0.302299
0.288994	0.277965	0.434732	0.140634	0.203233	0.056664	0.183867	0.378389	0.184738
0.330278	0.251034	0.394304	0.03995	0.2795	0.063026	-0.04669	0.495793	-0.08927
0.411013	0.2064	0.398382	-0.08164	0.369398	-0.02133	0.038616	0.413257	0.140089
0.251816	0.139344	0.290894	0.158226	0.42857	0.063247	-0.01996	0.324523	-0.01159
0.131929	0.224003	0.234457	-0.0849	0.378	-0.10868	-0.01821	0.358167	0.20277
0.352081	0.358951	0.252457	-0.11012	0.352909	-0.11948	0.253788	0.497062	0.196382
0.308242	0.305269	0.615827	0.240305	0.399314	0.525913	0.365746	0.31979	0.304502
0.259789	0.261659	0.158898	0.231297	0.048753	-0.12348	-0.11117	0.354346	0.088393
0.345947	0.27251	0.312075	0.066925	0.237821	-0.0501	-0.13651	0.334787	0.157505
0.195792	0.028269	0.041847	0.214804	0.368783	0.18745	-0.0404	0.366468	-0.0889
0.192542	0.224862	0.189692	0.1631	0.284234	0.064232	0.039011	0.028178	0.097947
0.296476	0.239143	0.069963	-0.15551	0.235319	-0.19218	0.045954	0.355209	-0.02067
0.146472	-0.01605	0.021872	0.03732	0.18963	-0.073	-0.22564	0.161781	0.165032
0.197553	0.212595	0.118816	0.29645	0.32691	-0.0099	0.051258	0.309136	0.137719
0.060443	-0.15201	0.020889	0.214901	-0.0193	-0.18005	-0.24935	0.33269	-0.04104

0.1379	0.276203	-0.0939	-0.04319	0.257016	-0.16277	0.086366	0.264429	0.28429
0.140232	-0.07306	0.115517	0.131286	0.32768	0.083183	-0.03405	0.261325	0.217137
0.318817	0.006081	0.184905	-0.05037	0.25262	-0.06354	-0.06386	0.392448	0.157492
0.039717	0.243158	0.699048	0.222175	0.435846	0.427795	0.264208	0.407365	0.280905
0.233191	-0.02862	-0.03399	0.203557	0.614509	0.250772	0.158655	0.302833	0.324587
0.456568	0.284895	-0.11542	0.101494	0.247431	0.094942	0.037352	0.250937	0.489705
0.183962	0.308098	-0.10466	0.184113	0.193129	-0.01199	-0.06977	-0.0063	0.282772
-0.10972	-0.19259	0.091479	0.357496	-0.24379	0.034059	-0.29936	-0.28951	-0.07553
-0.35077	-0.30664	0.159412	0.465849	-0.31564	0.233987	-0.36797	-0.50929	0.010769
0.219287	-0.12737	0.28935	0.064018	-0.05899	0.162199	0.090453	0.249278	-0.15588
-0.22196	-0.23851	-0.02818	0.317379	-0.24844	-0.09441	-0.32603	-0.37631	0.021666
0.097752	-0.09208	0.331498	-0.06489	0.352192	0.232591	-0.07424	0.168535	0.20929
-0.03301	0.033923	0.072839	-0.11273	0.134006	-0.38727	0.029652	0.014772	0.111605
0.19244	0.250416	0.292665	0.034264	0.15157	-0.13854	0.127013	0.245916	-0.07664
-0.01592	0.424328	0.684662	-0.15882	0.551585	0.359915	0.33669	0.392167	0.078843
0.254142	0.251947	0.062204	-0.12025	0.195898	-0.14204	0.143412	0.366055	0.061081
0.392914	0.065796	-0.05578	-0.05308	0.076131	0.008085	0.03495	0.395543	-0.22933
0.390552	0.215805	0.179707	0.137665	0.356354	-0.13719	-0.26178	0.260962	0.112756
0.425184	0.204175	0.026218	-0.00315	0.186765	-0.22304	0.086431	0.373096	-0.09108
0.354702	0.104314	0.051707	-0.12304	0.287169	-0.1348	-0.01018	0.378238	0.202209
0.254147	0.261963	0.152699	-0.05348	0.327429	-0.14854	-0.14583	0.403712	-0.04288
0.405599	0.207263	0.030035	-0.05002	0.305498	-0.15679	-0.11112	0.343762	0.069806
0.361786	0.195938	0.229278	-0.23256	0.203658	-0.31009	-0.15901	0.418981	-0.04429
-0.09921	0.372351	-0.30984	-0.40257	0.152166	-0.25555	0.38225	0.285639	0.324718
0.301356	0.237963	-0.03419	-0.24579	0.285157	0.105739	0.116854	0.353618	-0.11775
0.256413	0.022902	0.100704	-0.18903	0.161683	0.021675	-0.00031	0.350186	-0.10107
0.417431	0.213307	0.087881	-0.17408	0.189395	-0.08183	-0.01658	0.462671	0.054611
0.221281	0.101126	0.134574	-0.36308	0.490737	-0.09751	0.140213	0.377812	0.278261
0.281644	0.111899	0.208451	-0.04833	0.316353	-0.23346	0.194396	0.47228	0.026964
0.361879	0.042512	0.23057	0.028421	0.418087	-0.16579	0.001152	0.346018	0.163413
-0.09778	0.440398	0.77236	0.127734	0.499636	0.611377	0.239016	0.342043	0.257983
0.022096	0.335365	0.764273	0.377789	0.40012	0.514331	0.378661	0.439515	0.381903
0.043189	0.340154	0.813331	0.327978	0.479808	0.520538	0.421867	0.469548	0.410091
0.060415	0.33499	0.730796	0.423074	0.421307	0.48277	0.30615	0.388481	0.322441
0.118597	0.31254	0.443462	-0.00466	0.197974	0.145677	0.255835	0.375063	0.185071

TCGA-HZ-A	TCGA-HZ-A	TCGA-IB-7(	TCGA-IB-7(	TCGA-IB-7(	TCGA-IB-7(	TCGA-IB-78	TCGA-IB-78	TCGA-IB-78
0.268139	-0.18583	0.20818	0.081208	0.187348	0.244039	0.388159	-0.11062	0.317584
0.055832	-0.04729	-0.06121	-0.15635	0.413269	0.539272	0.193526	0.202108	-0.2491
-0.05247	0.0363	0.337168	0.137692	0.296497	-0.18198	0.332377	0.351201	0.296126
-0.17006	-0.06641	-0.22103	-0.0392	0.136259	0.184496	0.386619	-0.27687	0.245997
-0.00943	-0.31596	-0.3935	-0.18836	0.044238	0.062511	0.479154	-0.20706	0.028165
0.1229	-0.19655	-0.05495	-0.29711	-0.02963	-0.07266	0.169111	-0.12533	-0.20139
0.451089	0.177594	0.026894	0.166183	-0.18338	0.136676	0.229414	0.106248	0.544686
0.045157	0.173026	-0.16319	-0.3088	-0.20882	-0.27382	0.037636	-0.17285	-0.04842
-0.1339	0.05952	0.207719	-0.25797	0.021292	-0.14502	0.160977	0.057948	0.381895
-0.06899	-0.09304	0.004311	-0.35127	0.143882	-0.18925	-0.02532	-0.2379	0.238833
0.132173	0.122323	0.265799	-0.33393	-0.06136	-0.29912	0.027258	-0.02829	0.278233
0.24251	0.047049	0.102225	-0.37043	-0.21964	-0.14816	0.059452	-0.26124	0.166134
0.042102	-0.20052	0.270045	-0.04084	0.171185	0.252594	0.300744	0.185393	0.112012
-0.08172	-0.12656	0.090861	0.097977	-0.12639	-0.19514	0.421714	-0.15077	-0.06724
-0.42135	-0.5462	-0.37725	0.034308	0.14141	-0.0075	0.542876	0.226208	-0.25402
-0.00535	-0.50026	-0.12682	0.023699	0.247626	0.296835	0.527452	-0.04746	0.110807
0.323584	0.180541	0.093526	0.185887	0.251518	0.394808	0.263978	0.112384	-0.05955
-0.10306	-0.14729	-0.18903	0.02801	0.286943	-0.06615	0.174322	0.026769	-0.00082
0.209344	-0.18735	0.234436	-0.12117	0.080423	0.244687	0.055879	0.023693	-0.31932
0.663854	0.48661	0.465168	0.092421	-0.43414	0.326305	0.395736	0.307391	0.308825
0.022109	0.186738	0.176018	0.250057	-0.04348	0.023903	-0.07536	0.258947	0.090915
0.109861	0.015423	0.176542	0.03584	0.163983	0.396232	-0.30147	0.407802	0.316678
-0.16732	0.399722	-0.23061	0.162394	-0.11574	0.091697	-0.03409	-0.00458	-0.21563
-0.16693	-0.17747	-0.256	-0.11406	0.335614	0.359083	0.359645	-0.27166	-0.14431
0.101988	0.063538	-0.02967	-0.04651	0.082844	-0.18275	-0.09627	-0.20611	-0.22255
-0.41196	-0.31789	-0.0535	0.020898	0.418674	0.017279	0.202019	0.018236	-0.33571
-0.36013	0.247346	-0.11953	0.050041	-0.4664	-0.22313	-0.18669	-0.05575	-0.39972
-0.18904	0.241153	-0.15467	-0.32211	0.029398	-0.29291	0.066549	-0.09504	-0.17626
0.22651	-0.12749	0.218086	-0.27087	0.568784	-0.02573	-0.27329	0.038609	-0.62114
-0.19265	-0.0263	-0.01312	0.129585	0.071375	0.215584	0.408449	-0.01002	-0.22958
0.12502	-0.45052	-0.2129	0.408516	0.476898	0.540648	0.372449	0.381647	0.269954
-0.01432	-0.33257	-0.30089	0.000954	0.279182	0.353461	0.33679	0.113786	0.087171
-0.23223	0.054839	-0.13066	0.295564	0.03177	0.105914	0.355198	0.16464	-0.08345
-0.14181	0.093786	0.158841	0.284375	0.170872	0.174444	0.401488	0.176483	-0.03773
-0.15734	-0.0517	-0.14921	0.156657	0.096068	0.113549	0.333639	0.071706	-0.16354
0.162383	-0.19987	0.112419	0.042585	0.251751	0.189275	0.372445	0.026155	-0.03318
0.351668	-0.09758	0.014779	0.243008	0.11353	0.055142	-0.05667	0.441739	-0.31617
0.181575	0.040285	0.277133	-0.35161	-0.01262	0.223179	0.302961	-0.28836	0.15009
0.318588	0.394619	0.488236	-0.13435	0.177911	-0.09462	0.064012	0.05904	-0.04335
0.128064	0.168165	0.258814	-0.07365	0.214136	0.43745	0.322194	-0.18937	0.094053
0.189528	-0.0726	0.09402	-0.16231	0.212318	0.158721	0.231371	-0.27791	-0.19896
0.315899	0.305973	0.482855	-0.02417	0.292838	-0.39477	-0.09545	-0.06945	0.053814
0.347692	0.075386	0.170549	-0.38089	0.489702	-0.01298	-0.00097	-0.07197	-0.22207
0.075462	0.564835	-0.43752	-0.25114	-0.34458	-0.06135	0.640168	-0.32715	-0.226
-0.09941	-0.24657	0.070184	-0.10058	0.69985	0.073067	-0.21002	-0.28777	0.142798
-0.03805	0.231111	0.27177	-0.01829	0.315912	-0.13096	0.010595	-0.01936	0.291301
0.136672	-0.03647	-0.2207	-0.17384	0.184963	-0.19559	-0.14919	-0.42407	-0.21804
0.235365	-0.09507	0.017866	0.01358	0.024973	0.124448	-0.00615	0.105726	-0.09708
0.387593	0.062978	0.037796	0.166376	-0.26058	0.030077	0.096711	0.078539	-0.14559

0.129044	0.260565	0.069358	-0.0353	-0.0045	0.033487	-0.1242	0.06754	0.100554
0.322751	0.349007	-0.34808	-0.05764	0.323633	0.23606	-0.28957	-0.27385	-0.21235
0.257429	0.230465	0.140485	0.012916	0.347115	0.28521	-0.19213	0.237812	0.003826
0.415316	0.249525	0.241409	-0.382	0.437281	-0.01335	-0.05115	-0.08825	-0.07517
0.357056	0.015622	0.20602	-0.00594	0.217225	0.085795	-0.06543	0.080623	-0.15463
0.14681	-0.16859	-0.00988	0.148481	0.241942	-0.05196	-0.11381	0.145212	-0.16521
0.294878	-0.04094	0.061588	-0.20692	0.178247	-0.3255	0.206152	0.088576	0.265863
0.205281	0.043386	-0.15361	-0.34016	0.000268	-0.24449	0.060246	-0.35988	0.133763
0.054562	0.121427	-0.02199	-0.10647	0.022972	-0.25585	-0.08184	-0.3314	-0.17334
0.294678	-0.14076	-0.10251	-0.19313	0.416109	-0.01539	0.202188	-0.07035	-0.09099
0.256357	0.201817	0.085135	-0.06536	0.497716	0.121282	0.314193	0.121631	0.207183
-0.34186	0.184301	-0.39411	-0.58452	0.273021	-0.20585	-0.32521	-0.46187	-0.28399
0.293239	0.322324	-0.04859	-0.38546	0.260829	-0.065	-0.20949	-0.10582	0.364801
0.120664	-0.11305	-0.10807	-0.15109	0.422873	-0.00769	0.292261	-0.27576	0.225462
0.179454	0.071258	0.354656	0.02776	0.26805	0.078875	0.17723	0.14992	0.202665
0.112345	0.116023	-0.01017	0.287134	0.143438	0.295931	0.323512	0.06366	0.193182
0.263945	-0.42952	-0.46991	-0.15952	-0.04757	0.034026	0.583874	-0.27963	0.46427
0.17835	-0.26023	-0.19133	-0.24877	-0.02258	-0.28455	0.433852	0.055035	0.315787
-0.33731	-0.19635	0.057734	-0.24888	0.089266	0.053253	-0.12594	-0.12654	-0.36057
0.419463	0.044789	-0.0942	-0.16778	0.112845	-0.04355	0.181193	0.26295	-0.20372
0.184888	0.371977	-0.50674	-0.32886	-0.58342	-0.05457	-0.34881	-0.20621	-0.25936
0.434261	0.127264	0.165382	-0.26042	0.577646	0.068927	-0.05589	0.077419	-0.11629
0.236554	0.135428	0.21734	-0.17732	0.263342	-0.12934	0.163086	0.023105	-0.17789
0.348415	0.302989	-0.248	-0.03122	-0.25229	0.070831	-0.33139	0.203101	-0.28024
0.230084	-0.04661	0.154926	0.027736	-0.08662	-0.30246	0.315431	0.166603	0.097463
-0.02711	0.116701	0.362831	-0.1609	0.225775	-0.23014	0.109344	0.054496	0.203233
0.350501	0.124868	0.321459	-0.02138	0.020162	0.118447	0.175762	-0.25756	-0.00565
0.078485	0.667745	-0.30813	-0.36295	-0.49269	0.224363	0.570776	0.012518	0.261816
-0.08029	0.123822	0.303671	-0.30069	0.351472	0.057158	-0.20584	0.233971	-0.25402
-0.03372	-0.30341	-0.07612	-0.16042	0.271507	0.142796	0.031499	-0.04834	0.104843
0.017642	0.164214	0.304715	-0.26531	0.352525	-0.25006	0.120282	-0.2133	0.211457
0.02901	0.060434	0.342572	-0.25434	0.418983	-0.2445	0.133251	-0.16218	0.037728
0.300635	0.24276	0.216908	-0.08801	-0.06572	-0.32182	0.142299	-0.09866	-0.22308
0.391612	-0.05859	0.222837	0.085819	0.076441	0.222546	0.35611	0.128095	-0.19483
-0.07677	-0.1522	0.16241	-0.09063	0.013413	0.142139	0.239117	-0.19814	-0.09722
0.12396	0.700308	0.492648	-0.62401	0.231008	-0.51951	-0.58107	0.637761	0.458497
0.176503	0.183559	0.017725	0.159818	-0.10986	0.223613	-0.31384	0.155223	0.02891
0.543631	0.225693	-0.00669	-0.08691	-0.13854	0.202318	-0.02372	0.107684	0.194694
0.630853	0.121748	0.059821	0.435311	-0.16877	0.026911	0.421523	0.342369	-0.11826
0.074793	0.375596	-0.0642	0.189782	-0.22369	-0.24226	-0.33135	0.296966	0.073186
0.698968	-0.07081	-0.48951	0.510574	-0.16313	0.40754	0.300251	-0.2139	0.140285
0.48867	-0.34322	-0.46521	0.482604	0.472758	0.409931	0.429458	-0.06596	0.377105
-0.09681	-0.01752	0.108547	-0.29513	0.291312	0.035259	0.105183	-0.00496	-0.05389
0.329084	-0.22556	0.246218	-0.13213	-0.42815	0.001341	-0.17441	-0.01968	-0.20954
0.284849	0.06228	-0.02153	0.208946	-0.03989	0.164589	-0.09943	0.362745	0.192634
0.488215	0.113281	0.076804	0.452542	0.074499	0.286621	0.407845	0.516198	0.091269
0.451656	0.143561	-0.09377	0.271457	-0.43121	0.072498	0.394095	0.467354	0.111132
-0.14979	-0.06177	-0.09431	0.034826	0.039006	0.034566	0.158824	0.100493	-0.01998
-0.12087	-0.31104	0.010312	0.145118	0.199299	0.066898	0.12145	0.127443	-0.19791
-0.1149	-0.21191	0.00451	-0.21086	0.130006	-0.06797	0.05318	0.131882	-0.18246

-0.19049	-0.31893	-0.18575	0.100214	0.289682	0.038186	0.451996	0.123155	0.060736
-0.27729	-0.36816	-0.09003	-0.05772	0.337429	0.01505	0.386852	0.130541	-0.18765
-0.27246	-0.21688	-0.00666	-0.30595	-0.00522	0.125492	-0.14053	-0.1091	-0.35017
-0.16143	-0.20008	-0.14398	-0.12501	0.245059	-0.06148	-0.03714	-0.15816	-0.1852
0.353422	-0.04501	0.253116	0.417439	-0.23429	0.101005	0.236257	0.16178	-0.17712
0.058486	-0.11969	0.109808	0.091648	0.048273	0.237374	0.253651	0.236498	-0.23328
0.084109	0.027992	0.055967	0.274757	-0.22845	0.071688	0.095428	-0.02542	-0.24913
-0.04139	-0.07139	0.065588	0.123382	0.156375	0.062048	-0.03688	0.121251	-0.03952
-0.15185	-0.09582	-0.14796	0.101964	0.037182	0.038515	-0.1002	-0.24042	0.029161
-0.15506	-0.00208	-0.03375	-0.0329	0.118476	0.165704	0.207047	0.054958	-0.19168
0.224951	0.05646	0.238857	-0.33944	0.077491	-0.06153	-0.03825	-0.14705	0.312026
-0.21152	-0.27994	-0.20135	-0.18132	0.002943	0.068269	0.184757	-0.17088	0.151413
-0.12371	-0.02069	0.094418	0.111631	0.151922	0.134075	0.311187	0.037223	-0.09507
-0.24086	-0.26567	-0.08833	-0.25838	0.193562	-0.16158	-0.05184	0.099709	-0.2482
-0.10558	-0.16289	0.165744	-0.04197	0.13402	-0.02473	0.229552	0.143983	-0.10397
-0.23951	-0.22998	0.101264	0.083343	0.428107	0.300513	0.247401	0.424834	-0.19003
-0.21581	-0.16605	-0.10477	-0.31928	-0.06881	0.032752	0.107612	-0.15999	-0.24019
-0.10554	-0.26415	0.010481	-0.15941	0.101238	0.037205	0.146784	0.039418	-0.01933
-0.30175	-0.34232	-0.09427	0.239717	0.320726	0.222436	0.30948	0.268936	0.012716
-0.21502	-0.18641	0.00733	0.045024	0.111408	-0.13508	0.360052	0.118795	-0.01129
-0.03984	-0.08187	0.052588	-0.15935	0.034938	0.043714	0.249956	0.020097	0.072742
-0.16042	-0.32434	-0.06614	0.236266	0.2767	0.298997	0.36005	0.238232	-0.11848
-0.08217	-0.28305	0.014208	0.361133	0.164795	0.312517	0.561255	0.048299	0.043553
-0.24195	-0.35448	-0.25469	0.314447	0.425255	0.288895	0.501141	-0.01277	0.017771
-0.28547	-0.03707	0.141506	0.252596	0.308262	0.237662	0.461526	0.375294	-0.09994
-0.06171	0.208819	0.117946	0.002758	0.162571	-0.0181	0.225232	0.332744	-0.13524
0.012371	-0.07923	0.015056	-0.13388	0.190768	0.051414	0.138804	0.093912	-0.07549
0.03911	-0.2725	-0.38735	0.038298	0.448314	0.245686	0.010536	0.142637	-0.1358
0.036767	-0.23685	-0.28069	0.532194	0.280883	0.594278	0.430306	0.405784	0.350224
-0.17829	-0.3635	-0.0511	0.131236	0.205492	0.320834	0.326395	0.20528	0.036734
-0.31082	-0.17671	-0.0934	0.349918	0.159678	0.163655	0.404852	0.34556	0.158508
-0.2686	-0.05986	-0.04454	0.008781	-0.17378	0.076118	-0.03551	0.080661	-0.1564
0.05516	-0.19808	-0.01069	-0.09036	-0.07934	0.109491	-0.03031	-0.01749	-0.24693
-0.30191	-0.30748	-0.05455	0.102278	0.208645	0.203485	0.242447	0.219202	-0.12966
-0.18293	-0.37391	-0.12467	0.272672	0.426061	0.33128	0.506748	0.162025	0.027539
-0.24013	-0.28551	-0.13723	0.2402	0.313424	0.392779	0.422974	0.195738	-0.08452
-0.34934	-0.24549	0.059682	0.063218	0.276115	0.137233	0.354894	0.238806	-0.13237
-0.33438	-0.34499	-0.01254	0.138085	0.296678	0.2206	0.383236	0.226191	-0.24197
-0.31198	-0.33565	-0.03423	-0.08036	0.221358	0.186647	0.218906	0.216332	0.063998
-0.33443	-0.30693	-0.11191	-0.07859	0.153028	0.187055	0.282406	0.087129	-0.12641
-0.28223	-0.19429	-0.10733	0.101204	0.374883	0.099216	0.43415	0.169875	-0.22621
-0.16605	-0.40522	-0.25443	0.397001	0.371067	0.522622	0.467592	0.355513	0.35994
-0.30039	-0.09475	0.173899	-0.06586	0.203576	-0.02555	0.096255	0.329594	-0.19697
-0.30351	-0.26719	0.083734	-0.10677	0.227304	0.231492	0.243201	0.179225	-0.16946
-0.12106	-0.09826	-0.0165	-0.1197	0.160967	-0.23226	0.134604	0.192249	-0.07312
0.052972	-0.15765	0.131628	0.057995	0.199636	0.16338	0.125037	0.219066	0.183162
-0.15063	-0.22144	-0.00944	0.100965	0.184566	0.185229	0.321881	0.171415	-0.16709
-0.19267	-0.1414	-0.12568	-0.084	0.112305	0.17739	0.090724	0.022439	-0.19881
-0.25381	-0.17217	0.018545	-0.26433	0.021681	0.010101	0.019766	0.152529	0.038029
0.105518	-0.1842	0.011823	0.174025	0.073625	0.240294	0.190272	0.089866	-0.08648

-0.20815	-0.23366	0.097655	-0.14502	0.154052	0.041742	0.135792	0.113996	-0.07291
-0.31114	-0.11654	3.83E-05	-0.07194	0.100513	0.028386	-0.07797	0.117244	-0.12025
-0.31587	-0.37624	0.002549	-0.1094	0.353893	0.233005	-0.05153	0.05114	-0.23529
-0.16324	-0.26642	-0.33008	0.427549	0.295966	0.669704	0.411847	0.356669	0.292782
0.196241	0.16792	-0.03642	-0.50248	0.333828	-0.3586	-0.54931	-0.27637	-0.53598
-0.15667	-0.0332	0.107432	0.106235	0.429391	0.182086	0.10456	0.234081	0.000817
-0.05851	0.127648	0.08514	-0.15086	0.314009	0.059849	-0.0509	0.085318	-0.01068
0.108679	-0.04099	0.09129	-0.04927	0.151642	-0.04387	-0.01182	-0.15221	-0.21555
0.125889	-0.00821	-0.00461	-0.15065	0.226918	-0.162	-0.21443	-0.34156	-0.21307
0.084697	0.200704	0.243069	0.127968	0.165476	-0.00643	0.191098	-0.00183	0.071728
0.143515	0.056932	0.047682	-0.04536	0.047139	-0.17542	-0.17736	-0.13405	-0.18936
-0.0422	-0.24157	-0.26581	0.155004	0.400138	0.286052	0.124129	0.198475	-0.23572
-0.14356	-0.14815	-0.21846	0.015333	0.083815	0.156814	0.413016	-0.07323	-0.09377
-0.36488	-0.31565	-0.01655	0.079892	0.223566	-0.00704	0.468096	0.06058	0.053307
-0.2299	-0.41083	-0.20275	0.371053	0.359547	0.469911	0.425211	0.328421	0.186855
-0.11237	-0.15901	0.053548	0.140662	0.158511	0.190286	0.291306	0.113268	-0.04557
0.004014	-0.25856	0.162843	0.118624	0.250436	0.257507	0.340245	0.094339	0.003398
-0.28729	-0.32712	0.15505	0.06452	0.403921	0.157881	0.283726	0.170524	0.054477
0.038127	-0.16909	0.021399	0.205526	0.21743	0.219485	0.31506	0.204999	-0.05067
-0.04941	-0.26734	0.246378	0.076226	0.280394	0.265086	0.328595	0.151128	-0.02637
-0.08267	-0.1336	-0.02025	0.154561	0.191955	0.247791	0.159202	0.259626	-0.15866
-0.18053	-0.15274	0.220079	0.250389	0.229613	0.227989	0.345646	0.261925	-0.00285
0.006835	0.169151	-0.0202	0.072273	0.075377	-0.06609	0.394132	0.03874	0.081288
-0.05865	-0.36598	0.054173	-0.21778	0.006874	0.089753	0.270388	-0.1454	0.088781
0.036925	-0.07379	0.01018	0.241394	0.155255	0.160772	0.266027	0.239857	0.0225
0.208918	-0.10364	-0.01712	0.108355	-0.01717	-0.17304	0.239974	0.12663	-0.05205
-0.1032	-0.11724	0.10861	0.132134	0.158963	0.193668	0.159409	0.181191	-0.21943
-0.14583	-0.37392	0.105216	-0.05144	0.224035	0.11032	0.361016	0.139815	-0.10375
0.006685	-0.10117	-0.00519	0.251461	0.108997	0.258703	0.251791	0.132486	-0.1138
-0.04525	-0.04935	-0.08855	-0.01936	0.245509	0.272306	0.114803	0.099358	-0.17295
-0.08986	-0.57079	-0.2757	0.395319	0.473606	0.555343	0.533871	0.389457	0.426553
-0.10393	-0.41606	-0.3151	0.412247	0.319768	0.65575	0.503114	0.351675	0.329389
-0.13149	-0.4503	-0.42079	0.456367	0.330833	0.696789	0.567329	0.395188	0.333221
-0.22247	-0.47559	-0.33023	0.562281	0.456442	0.785723	0.579886	0.450179	0.331039
-0.19502	-0.27861	-0.02875	0.386006	0.309987	0.494012	0.487368	0.36975	0.253074

TCGA-IB-78	TCGA-IB-78	TCGA-IB-81	TCGA-IB-81	TCGA-IB-A5	TCGA-IB-A5	TCGA-IB-A5	TCGA-IB-A6	TCGA-IB-A7
0.045312	0.128194	0.163366	-0.31312	-0.00195	0.086977	0.103815	0.130302	-0.14466
0.018209	0.312362	-0.13045	0.004875	-0.31647	0.280194	0.035516	-0.15381	-0.16787
-0.11856	0.264967	-0.09906	0.224307	0.224324	-0.01343	0.185061	-0.11074	-0.23417
-0.11503	0.028311	-0.36198	-0.27916	-0.44371	0.442263	0.580454	-0.15003	-0.41061
-0.01412	-0.10321	-0.19984	-0.3417	0.114417	0.598332	0.4164	0.108525	-0.25944
-0.1008	-0.03215	0.079292	-0.01321	0.451169	-0.10564	-0.21027	-0.30505	-0.2819
0.230282	-0.22333	-0.17274	-0.44795	0.030328	-0.11187	-0.23203	-0.24254	-0.06186
-0.0676	-0.23471	-0.13639	-0.15774	0.30528	-0.24473	-0.2744	0.017687	-0.01321
0.090438	0.017488	0.105763	-0.08146	0.38117	-0.28578	-0.28665	-0.06156	-0.1433
0.04524	-0.01624	0.08752	-0.20718	0.305351	-0.09159	-0.344	-0.26239	-0.2598
0.043254	-0.27287	0.097391	-0.13127	0.376572	-0.36878	-0.46067	-0.08491	-0.15761
-0.15181	-0.12455	0.111284	-0.24901	0.255353	-0.37148	-0.25559	-0.14231	-0.15989
-0.11164	0.1414	-0.28658	0.068414	0.358414	0.244443	0.1317	-0.32192	-0.25838
0.339678	0.031833	-0.19874	-0.29339	0.068841	0.316192	-0.08728	-0.04915	0.208609
0.217904	0.29814	-0.21468	-0.07533	0.063736	0.522837	0.400501	-0.19859	0.060648
0.106231	0.296047	-0.19441	-0.37831	-0.32301	0.522024	0.492097	-0.36415	-0.34294
-0.0238	0.271262	0.071558	0.17423	0.162265	0.357363	-0.02508	-0.15043	-0.33097
0.197096	0.063922	0.239862	-0.0951	0.166011	-0.12992	0.102995	-0.22494	-0.48721
-0.15234	0.058943	0.24738	-0.19643	0.312917	-0.16822	-0.17639	-0.22472	-0.1083
0.356269	-0.10993	-0.54617	-0.20802	0.180874	-0.44463	-0.12972	0.002052	-0.23401
0.001831	-0.08952	-0.14557	0.26882	-0.21209	-0.14689	-0.15492	0.401644	0.192387
-0.05859	0.254105	-0.17198	-0.04508	0.008098	-0.31653	-0.22276	-0.16136	0.058765
0.17913	-0.08079	-0.02847	-0.02245	0.129304	-0.30888	-0.03457	0.272323	-0.10451
0.108253	0.259573	-0.25514	0.048945	-0.01706	0.436272	0.298012	-0.3041	-0.35357
0.147055	-0.29487	-0.12069	-0.27111	0.224153	0.281277	-0.22047	0.030406	0.025236
-0.16743	0.307974	0.149441	-0.33341	-0.2914	0.336294	0.27763	0.208542	-0.02159
-0.57928	-0.3084	-0.08107	0.141175	-0.48667	-0.22932	-0.15262	0.322821	0.670969
-0.16235	0.077067	0.147487	0.201118	-0.11645	-0.14667	-0.24887	-0.09691	0.267114
0.274673	0.406833	0.646989	-0.09458	0.415879	-0.15322	-0.23717	0.103184	-0.08066
-0.10017	0.278985	-0.30785	0.350166	-0.07719	0.146195	0.333993	0.000901	-0.21719
0.435279	0.59393	0.376151	0.199645	-0.40402	0.238855	0.090918	-0.37516	-0.45689
0.417182	0.362893	0.275709	0.233764	-0.10389	-0.00693	-0.20861	-0.37791	-0.32675
-0.15802	0.213114	0.065749	0.210052	-0.31884	0.328086	0.267208	-0.19515	0.183899
-0.1761	0.244011	-0.04515	0.29092	-0.35512	0.335754	0.407652	-0.28505	0.209784
-0.16025	0.220956	0.075954	0.21885	-0.3226	0.373277	0.223307	-0.27952	0.153187
0.024009	0.184941	-0.21659	-0.0814	0.303529	-0.14177	0.195218	-0.02885	-0.11311
0.416331	0.367453	0.354705	-0.2609	0.629331	0.080668	-0.18155	-0.1718	-0.47289
-0.08299	-0.09652	-0.3553	-0.31448	0.558145	-0.3608	0.306728	0.029168	-0.00642
-0.13203	0.087534	-0.00833	0.275637	0.497017	-0.47311	-0.00463	0.011364	0.017616
-0.24402	-0.17514	-0.29135	-0.31392	0.511569	-0.13148	0.094216	0.07135	-0.12671
-0.01712	0.157069	-0.41392	-0.02606	0.299811	0.265558	0.410823	0.1656	0.005179
-0.13556	0.139008	0.125375	0.346148	0.387019	-0.50435	-0.23962	-0.08011	-0.19988
0.26488	0.366201	0.456088	-0.36933	0.316779	-0.19825	-0.38622	-0.37696	-0.4765
0.453112	0.30119	-0.25122	-0.12724	0.572434	-0.27392	-0.31305	-0.2331	-0.60276
0.219665	0.57772	0.518414	-0.41832	0.133484	-0.07308	-0.46611	-0.35965	-0.55316
0.187045	-0.00245	-0.12324	0.145706	0.203594	-0.3596	-0.12415	-0.05271	0.09565
0.277509	-0.41906	-0.30101	-0.41701	0.50011	0.121689	-0.38999	0.055996	-0.46826
0.210072	0.049625	-0.09942	-0.16164	0.075655	0.126503	0.007664	0.003895	-0.14081
0.189697	-0.08433	-0.35228	-0.26099	0.251975	0.071165	0.105698	0.211688	-0.16245

0.010067	0.118046	0.101889	-0.17509	0.268942	-0.30199	-0.03935	-0.113	-0.20039
0.423259	0.37519	0.645291	-0.19954	0.126307	-0.14505	-0.09125	-0.2684	-0.3576
0.238856	0.410507	0.255396	-0.00441	-0.04596	-0.13503	0.11019	-0.28285	-0.21506
0.334009	0.351138	0.51195	-0.43091	0.468894	-0.36808	-0.54053	-0.36275	-0.46377
-0.07725	0.272729	0.086522	-0.14895	0.004405	-0.33617	-0.36129	-0.17966	-0.12337
0.315174	0.209512	0.073647	-0.08486	0.260229	-0.29234	0.092655	-0.26192	-0.38882
0.458558	-0.00683	0.029567	-0.2531	0.370568	-0.43884	-0.12893	0.010349	-0.39977
0.230329	-0.1374	0.049833	-0.38779	0.073243	-0.16111	0.096949	0.054138	-0.20834
0.091643	-0.12192	0.046007	-0.3605	0.246173	-0.24463	0.293749	0.181142	-0.03937
0.404987	0.377529	0.220086	-0.26029	0.090249	0.046409	-0.26521	-0.36196	-0.49118
0.211748	0.52867	0.382424	-0.36061	-0.01607	-0.19166	-0.35165	-0.30489	-0.44528
0.063061	0.177927	0.460728	-0.16793	-0.16687	-0.00655	-0.51983	-0.10346	-0.21595
0.397691	0.304915	0.375555	-0.15389	0.18534	-0.32761	-0.31557	-0.06581	-0.46458
0.175118	0.109168	0.084747	-0.31396	0.317993	-0.11188	-0.32432	-0.30184	-0.44212
-0.14749	0.313617	0.061032	0.290259	0.343439	-0.17948	0.228427	-0.08155	0.030624
0.150509	0.184935	-0.27314	-0.15115	0.261148	-0.11104	0.222998	0.10201	-0.27986
-0.40082	-0.07472	-0.36729	-0.10829	-0.46287	0.568506	0.571607	-0.20771	-0.13513
-0.23865	0.196822	-0.2417	-0.17302	-0.28906	0.465951	0.395748	-0.10258	-0.20528
-0.28697	0.077827	0.121351	0.227513	0.087217	-0.16879	-0.05235	-0.3933	-0.06326
0.263062	0.238742	0.153877	-0.29996	0.419752	-0.02327	-0.32935	-0.09375	-0.43754
0.091749	0.130081	-0.26943	-0.40304	0.282586	0.50506	-0.02593	-0.03511	0.12987
0.396083	0.458773	0.555698	-0.39474	0.30366	-0.37832	-0.51335	-0.37585	-0.54727
0.284194	0.205584	0.169155	-0.24029	0.45289	-0.32933	-0.4626	-0.36971	-0.47204
0.244117	0.316489	-0.31401	-0.31881	0.078587	-0.23018	-0.10228	-0.39877	0.149187
0.198062	-0.22698	-0.43865	0.015472	0.430855	0.365597	-0.07606	0.263295	-0.37138
0.021047	-0.23468	0.175576	0.347914	0.422831	-0.34424	-0.16581	-0.23135	-0.03074
0.248738	-0.11059	-0.33713	0.148573	0.437548	-0.17938	0.24039	0.241644	-0.10735
0.247789	0.153341	-0.45409	-0.33905	0.451439	-0.27707	-0.43772	-0.17673	-0.62389
-0.12256	-0.06654	0.376077	-0.05193	0.228524	-0.46084	-0.25149	0.303557	0.197871
0.013534	0.172379	0.116057	0.454699	0.047146	-0.25615	0.113591	0.003736	-0.41674
0.218162	-0.09147	0.158174	0.060437	0.43237	-0.43622	-0.26727	-0.16412	-0.14126
0.280944	-0.03539	0.307072	0.054894	0.415109	-0.41925	-0.28964	-0.09365	-0.21741
0.149389	-0.29482	-0.27348	0.142186	0.419257	-0.2822	0.099592	0.012555	0.220732
-0.00584	0.288346	-0.32505	-0.08706	0.489451	0.118744	0.304567	-0.33932	0.194872
-0.27838	0.14962	0.099973	-0.07932	0.001587	-0.05113	-0.38317	-0.2315	-0.30392
0.719198	-0.24239	0.322203	-0.55129	-0.18853	0.089829	-0.52621	0.183237	0.458262
0.046124	-0.12545	-0.03957	0.006686	-0.20861	-0.26739	-0.21564	0.330173	0.083238
0.431834	-0.02423	-0.32877	-0.56618	0.022765	0.160397	-0.06769	0.252058	-0.28395
0.417563	-0.1767	-0.44912	-0.38888	0.39583	-0.01933	0.209873	0.344399	0.249096
0.13003	-0.15503	-0.18478	-0.02481	-0.35374	-0.28217	-0.40789	0.255588	0.226301
0.492423	0.333962	-0.5103	0.083975	0.20888	0.322029	0.053789	-0.15586	-0.25582
0.623344	0.442665	0.635368	-0.34001	-0.583	0.069338	-0.30356	0.377819	-0.47303
-0.16428	0.219875	0.281995	0.012272	0.40921	0.059169	-0.0632	-0.35057	-0.46053
0.121797	-0.44402	-0.54594	-0.07845	0.483517	-0.45277	-0.31016	0.352649	0.084241
0.22317	-0.10774	-0.19513	-0.21318	0.090335	-0.33903	-0.04259	0.333194	0.215466
0.439108	0.134537	-0.24379	-0.34643	0.168031	-0.07867	0.015341	0.349636	0.086513
0.128555	-0.2414	-0.50205	-0.26615	0.222751	-0.30566	0.289454	0.1089	0.167896
-0.01498	0.174053	0.214681	0.106924	-0.25971	0.143465	0.05865	-0.13702	0.01802
-0.08222	0.348841	0.353369	0.151209	-0.11559	0.220188	0.090098	-0.20805	0.086799
0.044376	0.227228	0.162101	0.010178	0.082653	0.256116	0.023526	-0.27249	-0.18552

0.314415	0.381842	0.363099	0.054344	-0.36157	0.257582	0.043843	-0.37026	-0.25651
0.256245	0.396508	0.314042	0.038461	-0.1306	0.227163	0.147801	-0.38775	-0.29267
-0.26979	0.052883	-0.03734	0.26955	0.155997	-0.06014	-0.16894	-0.3965	-0.11505
0.088951	0.200046	0.306009	-0.11209	-0.19274	0.316193	-0.00041	-0.28803	-0.18291
-0.13411	-0.02385	-0.16086	0.003197	-0.01884	0.205612	0.206701	0.125509	0.218645
0.028471	0.15206	-0.13071	0.088389	-0.01514	0.252034	0.055917	-0.10832	-0.10707
-0.20944	-0.13954	-0.10691	0.299425	-0.20965	0.13394	0.240177	-0.0366	0.134191
-0.10927	0.197032	-0.018	0.009718	-0.17305	0.047434	-0.01846	0.141401	-0.00278
0.06577	0.104168	0.395117	-0.30074	0.187497	-0.1175	-0.18447	0.119218	-0.15287
-0.03014	0.147186	-0.06222	0.116189	0.03969	0.123137	0.21059	-0.11175	0.011405
0.200981	0.05707	0.196019	-0.23374	0.525842	-0.33448	-0.32544	-0.26777	-0.20802
-0.16523	0.118991	0.244213	-0.21042	-0.09949	0.1061	-0.18209	-0.12943	0.066945
0.076425	0.307435	-0.01411	0.176305	0.069765	0.070434	0.119488	-0.22603	-0.12262
-0.1687	0.237233	0.229296	-0.05058	-0.05018	0.216553	-0.06001	-0.34851	-0.23596
0.041391	0.188537	-0.01784	0.161149	-0.19512	0.197666	0.24403	-0.14205	0.127858
-0.20239	0.47564	0.225128	0.231357	-0.26426	0.254126	0.192862	-0.47323	0.15702
-0.27835	-0.13169	0.12252	0.312695	-0.32822	0.280348	0.321553	0.127715	0.357056
-0.05526	0.23759	-0.25881	0.235074	-0.30042	0.179427	0.358379	-0.2791	0.077633
-0.09688	0.419463	0.296362	0.277092	-0.4493	0.388943	0.254544	-0.25046	-0.12176
-0.01885	0.084857	-0.04854	0.158772	-0.07383	0.198364	0.318569	0.085974	0.198416
-0.06284	0.107828	0.101694	0.060539	0.010534	-0.01866	-0.01835	-0.18008	0.005616
-0.16202	0.372239	0.125268	0.209848	-0.34889	0.359701	0.375366	-0.14841	0.057715
-0.41318	0.34551	0.026502	0.170217	-0.49806	0.459928	0.500739	-0.26158	0.068625
0.18827	0.470555	0.283104	0.100674	-0.3652	0.398499	0.052281	-0.50634	-0.34023
-0.00727	0.340174	0.042136	0.158277	-0.32183	0.17526	0.266811	-0.27199	0.075999
-0.01154	0.284307	-0.12933	0.057249	0.085533	0.11003	0.111196	-0.16199	0.139521
-0.17264	0.312429	0.101181	-0.0274	-0.16489	0.327532	0.165878	-0.20765	-0.20518
0.253718	0.384403	0.375589	0.237254	-0.32535	-0.0096	0.023818	0.192812	-0.25888
0.108621	0.372242	0.113263	0.261303	-0.13881	-0.00968	-0.00355	-0.25576	-0.17088
0.175849	0.450815	0.205458	0.209653	-0.08744	0.172681	0.178861	-0.3066	-0.11538
0.084945	0.395082	0.277044	0.210023	-0.29511	0.039814	0.103346	-0.16796	-0.20658
-0.17468	0.262837	-0.08306	0.294695	-0.02995	-0.03051	-0.25164	-0.1239	-0.0226
0.16954	0.202421	-0.05984	-0.11485	0.101409	-0.06139	-0.083	-0.19853	-0.25756
-0.02504	0.348789	0.36492	0.10455	-0.28699	0.181249	-0.14248	-0.31571	-0.15051
0.261381	0.429315	0.319756	0.251595	-0.45225	0.287949	0.199932	-0.35448	-0.34977
0.22605	0.325024	0.25363	0.201165	-0.06631	0.053862	0.189266	-0.2696	-0.0984
0.055913	0.398861	0.306029	0.335816	-0.1504	0.18639	0.12713	-0.30883	-0.11552
0.226194	0.433056	0.205235	0.150605	-0.03373	0.105881	0.216256	-0.33463	-0.17976
0.126947	0.358943	0.299776	0.09151	0.007292	0.036228	-0.10597	-0.32385	-0.19886
0.071466	0.330635	-0.04844	0.127822	0.054008	0.014555	0.149141	-0.24739	-0.2015
0.158716	0.382152	0.149931	0.156381	-0.14595	0.296763	0.147047	-0.34512	-0.08096
0.406329	0.457443	0.382797	0.124948	-0.16834	0.150045	0.0272	-0.44026	-0.41186
-0.05771	0.234474	0.080198	0.105325	0.258761	0.205256	-0.03363	-0.22751	-0.11393
-0.05624	0.325896	0.091164	0.148618	-0.15918	0.241127	0.177894	-0.22757	-0.12521
-0.19476	0.223271	0.225665	-0.10588	0.025148	0.206058	-0.11095	-0.20136	-0.26039
0.07041	0.183575	0.230812	0.135045	0.143755	0.112775	0.185093	0.229528	0.172846
-0.00711	0.320455	0.044817	0.252979	-0.19092	0.234748	0.313948	-0.21462	-0.03939
-0.16402	0.14828	0.115828	-0.03102	0.082732	0.148798	-0.13868	-0.12455	-0.03876
-0.22653	0.108614	0.1548	0.008024	0.015093	-0.057	-0.20229	-0.34234	0.001468
-0.09314	0.157198	0.039635	0.020068	-0.10127	0.302733	0.051854	-0.05531	-0.01963

-0.20778	0.154925	0.049695	0.016083	-0.18345	0.291794	0.201766	-0.30646	0.014662
-0.23229	0.144107	0.19097	0.104979	0.094819	0.124296	-0.08865	-0.04333	-0.0679
-0.24828	0.3387	0.137762	-0.09359	0.174215	0.224848	-0.08316	-0.19366	-0.13458
0.40113	0.495126	0.38467	0.325827	-0.15864	0.192127	-0.14104	-0.59419	-0.34894
-0.1587	0.39234	0.591879	-0.37459	0.430769	0.221621	-0.59681	-0.10111	-0.40518
0.199631	0.333447	0.316572	0.126954	-0.07576	0.199932	-0.07414	-0.11088	0.051354
0.032522	0.34431	0.235661	0.345045	0.171224	0.195576	0.02092	-0.33438	-0.09889
0.189793	-0.14149	-0.07988	-0.22946	0.367689	0.029239	-0.16373	-0.06551	-0.40827
0.263587	-0.37112	-0.1164	-0.38489	0.480399	-0.06115	-0.3528	-0.02352	-0.46062
0.136569	0.255611	-0.07525	0.135779	0.248779	0.036949	-0.16873	-0.34888	-0.15709
0.204238	-0.22258	-0.0946	-0.17962	0.405585	-0.03694	-0.18839	-0.01603	-0.3195
0.04445	0.466614	0.317777	0.160877	-0.37509	0.330161	0.304079	-0.20858	-0.37959
0.105949	0.227831	-0.21756	-0.10055	0.257177	0.396513	0.050447	-0.04775	-0.33917
0.248604	0.293381	-0.07767	0.15404	0.162057	0.256828	0.041578	-0.07424	-0.20394
0.303635	0.555787	0.217688	0.384889	-0.33638	0.308239	0.230981	-0.46614	-0.35697
-0.09282	0.279906	0.052303	0.179292	-0.25707	0.19024	0.249891	-0.14669	0.067733
0.142674	0.326953	0.206489	0.001844	-0.13655	0.272854	0.326748	-0.14009	-0.10938
-0.15395	0.417062	0.282752	0.197673	-0.16464	0.166892	0.000801	0.059491	-0.02443
0.117474	0.302745	0.144164	0.07647	-0.09826	0.178955	0.373766	-0.05822	0.096908
-0.03544	0.334027	0.261737	0.130897	-0.13701	0.185298	0.287321	-0.24022	-0.1581
0.015825	0.414104	0.266871	0.100244	-0.26556	0.364773	0.234327	-0.27542	0.151434
-0.11556	0.353933	0.299758	0.122646	-0.24464	0.240694	0.233285	-0.1674	0.089465
-0.18023	0.359051	0.127318	-0.01092	-0.31213	0.211097	0.24675	-0.30085	0.161257
-0.11252	0.095693	-0.31649	0.234116	-0.3355	0.199895	0.479876	-0.23286	0.243556
0.082471	0.329345	0.340268	0.105819	-0.35489	0.347602	0.283769	-0.25159	-0.02537
-0.00864	0.140028	0.359122	0.018347	-0.30317	0.270616	0.173501	-0.20528	0.241443
-0.08308	0.324864	0.174221	0.132491	-0.19386	0.309897	0.23847	-0.22963	0.005823
-0.09024	0.299066	0.24735	0.015402	-0.20953	0.24955	0.24375	-0.34952	-0.15719
-0.25243	0.273624	-0.06485	0.312879	-0.27511	0.254722	0.190241	-0.24145	0.13427
-0.04991	0.376534	0.316978	-0.05772	-0.08587	0.180639	0.096339	-0.18743	0.04529
0.46722	0.609756	0.426647	0.015239	-0.42331	0.403057	-0.14962	-0.53888	-0.52219
0.260319	0.463464	0.360987	0.256017	-0.16026	0.040643	0.093528	-0.59369	-0.40996
0.369178	0.560711	0.358079	0.287744	-0.25835	0.171964	-0.01921	-0.64558	-0.40575
0.352301	0.468044	0.499193	0.274368	-0.29051	-0.00038	0.035971	-0.63505	-0.39795
0.167123	0.356955	0.154219	0.255302	-0.33297	0.168146	0.338847	-0.49825	-0.29904

TCGA-IB-A	TCGA-IB-A	TCGA-IB-A	TCGA-IB-A	TCGA-IB-A	TCGA-L1-A	TCGA-LB-A	TCGA-LB-A	TCGA-M8-A
-0.08052	0.146275	-0.252	-0.29079	0.001979	-0.07125	0.10129	0.465549	-0.05271
-0.39708	-0.3058	-0.56912	0.211441	0.262694	-0.20509	0.597198	0.574689	-0.10222
-0.17498	0.080424	-0.1634	0.361766	0.433749	-0.03817	-0.18114	0.042672	0.009857
-0.42158	-0.26187	-0.32927	0.03756	-0.38528	-0.1276	0.070649	0.216028	0.319296
-0.04519	0.031621	-0.13473	0.054122	-0.18718	-0.0663	0.046178	0.304195	0.453543
-0.14618	0.003419	-0.05768	0.222182	0.129119	-0.3571	0.023794	-0.05444	0.259987
0.155687	0.042076	0.033554	0.003331	-0.2854	-0.09797	0.519091	0.178877	0.279928
0.03879	-0.10207	0.094236	0.149528	0.068084	-0.18633	0.076079	-0.20894	0.240176
-0.00272	-0.2552	-0.07947	0.090231	0.312049	-0.04639	0.092589	-0.16654	-0.02385
-0.00624	-0.34979	0.059499	0.148232	0.383568	-0.17806	-0.17027	0.013929	-0.31723
0.026304	-0.21913	0.197001	0.309334	0.378878	-0.22659	-0.0282	-0.13955	-0.14774
0.049678	-0.35868	0.01977	0.228065	0.343821	0.177195	0.261308	-0.18561	-0.00011
-0.2314	0.036998	-0.26192	0.178692	0.300563	-0.49247	0.154993	-0.03021	0.240543
-0.06481	0.228844	0.093471	-0.1033	-0.1702	-0.18952	0.005338	0.105718	0.308847
-0.19295	0.198815	-0.58234	0.059385	-0.17496	-0.20775	-0.11083	0.473577	-0.04053
-0.26015	0.032829	-0.15468	-0.09969	-0.07972	-0.19271	0.187503	0.251875	-0.08551
-0.44437	-0.15511	-0.34112	0.144897	-0.52628	-0.01897	0.264836	0.253177	0.228811
-0.04013	-0.19232	0.02862	0.109562	0.147111	0.190194	-0.05288	0.318258	0.059329
-0.06652	-0.61209	0.184486	0.016232	0.118094	0.004681	0.280226	0.363667	-0.37281
0.503402	0.13105	-0.28723	-0.34492	-0.0839	-0.10121	0.391962	0.344099	-0.39248
0.352235	-0.04858	-0.0247	0.181738	-0.3945	0.20801	-0.10372	-0.33675	0.11402
0.035225	0.504023	-0.3076	0.269513	-0.22677	-0.18511	-0.3442	-0.57163	-0.16059
0.000811	-0.26891	0.062305	0.101998	-0.26839	-0.34918	0.063053	0.137968	0.112231
-0.3597	-0.37117	-0.40089	0.033456	-0.09927	-0.2876	-0.03178	0.361742	0.272562
0.114754	-0.05836	0.125179	-0.00517	-0.2446	-0.11115	-0.0878	-0.06475	-0.20961
-0.17424	-0.27258	-0.18485	-0.10641	0.480654	0.101425	-0.11934	0.2781	-0.27129
-0.33864	-0.07716	-0.07784	0.173058	-0.01194	0.141822	-0.28719	-0.44832	0.153959
-0.00474	-0.1406	0.253555	-0.12111	0.252546	0.205408	-0.28962	-0.2514	0.08521
0.370238	0.033601	0.124394	0.307295	0.534008	-0.29722	0.075322	0.350694	-0.47175
0.049646	0.087859	-0.13064	0.420591	-0.14825	0.015269	-0.27912	-0.14395	0.373959
-0.10754	-0.11011	0.027678	-0.03062	0.338818	-0.35616	-0.21689	0.431989	-0.00387
0.483653	-0.02347	0.078803	-0.20226	-0.02151	-0.32795	-0.15909	-0.28621	0.214281
-0.35094	-0.06329	-0.03899	-0.12846	-0.04751	-0.11647	-0.37152	-0.15134	0.255523
-0.23214	0.034616	-0.07291	-0.0971	-0.12707	0.058813	-0.40572	-0.13242	0.300812
-0.3878	-0.04408	0.022378	-0.1922	0.110545	-0.17933	-0.35174	-0.10602	0.174517
0.215312	-0.04287	-0.05911	0.387094	0.009978	0.059281	-0.25634	0.062065	-0.0568
0.103859	-0.38694	-0.57014	0.033801	0.211545	-0.07439	0.326368	0.436051	-0.64231
0.488804	-0.04321	-0.18422	0.463331	-0.11546	0.35897	0.007201	0.157618	0.116417
0.19625	-0.33502	-0.0644	0.471281	0.044679	-0.28118	-0.30855	0.133298	-0.27926
0.07213	-0.11743	-0.236	0.322586	-0.05107	0.005602	-0.08817	0.124422	0.221445
0.111762	0.046974	-0.1142	0.232769	0.028752	0.13527	-0.15668	-0.04239	0.297067
-0.27904	-0.19096	0.13562	0.37551	0.411364	-0.48806	-0.14902	0.113621	-0.47667
-0.04591	-0.21933	-0.50851	0.138609	0.611322	-0.30119	0.205833	0.479237	-0.50724
0.014256	-0.39428	-0.16629	0.079936	0.414334	-0.27799	0.500686	0.346668	0.479972
-0.34016	-0.25073	-0.52427	-0.20401	0.58756	-0.46574	0.272775	0.223323	-0.62152
0.032378	0.011588	0.193168	0.3759	0.210914	-0.32712	0.136435	-0.12921	-0.14713
0.340049	-0.21468	0.160108	-0.05508	-0.25699	-0.10979	0.09781	0.044477	-0.356
0.058918	0.066918	0.001227	-0.10157	-0.05049	0.047932	0.14047	0.105685	-0.34794
0.35292	0.145198	0.173506	-0.03132	-0.21022	0.168584	0.275811	0.022531	-0.31997

0.495818	0.074105	-0.07533	0.100006	0.147572	-0.2275	0.110673	0.349682	-0.31261
-0.16324	0.140042	-0.12246	-0.31766	0.395563	-0.10652	0.521821	0.596096	-0.45538
0.138121	0.10905	-0.10966	0.065358	0.032632	0.086974	0.147611	0.271451	-0.15819
0.002568	-0.40673	-0.52907	-0.17157	0.561048	-0.23239	0.162314	0.576437	-0.5024
-0.02133	-0.07791	-0.40519	-0.42195	0.276466	-0.14516	0.155323	0.247439	-0.30374
0.070185	0.135002	-0.30831	0.187117	0.237884	-0.25665	0.269871	0.103803	-0.24958
-0.20139	0.174803	0.001866	0.041599	0.238574	-0.28515	0.154795	0.317152	-0.28717
0.115976	0.202476	0.125042	-0.0025	0.230768	0.102736	0.027545	0.191368	-0.3575
-0.00966	0.235877	0.173908	0.016987	0.373864	0.027311	0.121908	0.373764	-0.26391
-0.31773	-0.09949	-0.25154	-0.02651	0.389605	-0.47338	0.104025	0.340307	-0.22882
-0.38477	-0.35223	-0.37978	-0.20239	0.519618	-0.2471	-2.28E-05	0.497381	-0.44102
-0.40152	-0.29962	-0.22274	0.354642	0.490233	-0.32854	0.498729	0.246657	-0.54684
0.135411	-0.31212	-0.27191	0.16729	0.330429	0.047041	0.100066	0.422928	-0.42191
-0.17748	-0.19918	-0.19401	0.285946	0.363365	0.009432	0.074864	0.201913	-0.30149
-0.09464	-0.03053	0.008864	0.362255	0.030415	0.1272	-0.25094	0.254307	-0.20633
-0.13961	-0.3493	-0.31885	0.232121	-0.08268	-0.04119	0.132788	0.348094	0.066203
-0.35637	-0.01228	0.111965	-0.17571	-0.46184	0.158041	0.161263	-0.05017	0.388713
-0.15918	-0.23855	-0.01601	0.019668	-0.21307	-0.0876	0.204364	-0.10787	0.178761
-0.08641	-0.17425	-0.18237	0.323017	0.247215	-0.28505	-0.26567	0.097009	-0.03793
0.194755	-0.24496	-0.28474	0.09	0.261091	0.0171	0.204814	0.366324	-0.35871
0.232744	-0.1743	0.11833	-0.25504	-0.15174	0.048631	0.299415	0.152911	-0.31078
-0.07363	-0.42901	-0.52107	0.037405	0.509079	-0.40576	0.209922	0.61335	-0.47963
0.082787	-0.53649	-0.44645	-0.08413	0.503645	-0.35675	0.176046	0.315444	-0.38658
0.200887	0.403514	0.135639	0.050343	-0.28603	0.441564	0.169451	0.303038	-0.6019
0.112246	0.487503	0.259039	-0.22287	-0.13288	0.035007	0.148685	0.176433	-0.37668
0.03864	-0.13764	0.03961	0.438799	0.319358	-0.40686	-0.2187	-0.16353	-0.30287
0.318067	-0.31958	0.150426	0.37258	-0.09447	-0.2284	0.139244	0.294204	-0.17519
-0.30584	-0.42699	-0.54852	0.273239	-0.01162	0.186662	0.593022	0.172327	0.021217
0.304407	0.392374	-0.2665	0.182465	0.2383	-0.36312	0.14956	0.120298	-0.12271
-0.39263	-0.43547	0.203782	0.654191	0.363599	0.188015	0.014747	0.052878	0.016068
-0.01193	-0.24353	0.03684	0.479032	0.302078	-0.41552	-0.10858	0.053696	-0.32089
-0.1988	-0.10804	-0.03008	0.440317	0.342769	-0.43408	0.019973	0.106572	-0.3392
0.123205	-0.01526	0.354576	0.397583	-0.03188	-0.2951	0.031449	-0.08843	-0.11333
0.308209	-0.01194	-0.12173	0.233026	0.085626	0.374131	0.328939	-0.13029	0.050114
-0.1154	-0.1389	-0.21354	0.313978	0.307797	-0.21026	-0.12952	0.081517	0.078793
0.707308	-0.61807	0.213397	-0.64268	0.151758	-0.45707	0.380656	0.395066	-0.7992
0.379074	0.399252	0.172504	-0.09939	-0.39486	0.26157	-0.15772	-0.40136	-0.39974
0.501187	-0.15017	-0.34432	-0.30902	-0.2137	-0.17272	0.410381	0.051115	-0.52769
0.425537	0.530761	0.407151	-0.42355	-0.58994	0.453906	0.291539	-0.34606	-0.32749
0.448898	0.062236	0.325395	-0.06518	-0.18324	0.234645	0.062441	-0.328	-0.3998
0.404738	0.361206	0.482576	0.264558	-0.62388	-0.03773	0.082264	-0.06944	-0.16253
-0.14236	0.094747	-0.27912	-0.43289	0.242695	-0.14316	0.383363	0.706293	-0.51084
-0.04607	-0.26355	-0.38029	0.314297	0.377754	-0.16147	0.086329	0.146901	-0.22682
0.278457	0.135456	0.55755	0.05599	-0.51949	-0.13226	0.080479	-0.26077	-0.1746
0.353751	0.345342	0.113171	-0.17069	-0.37574	0.269913	0.192589	-0.2797	-0.29679
0.213084	0.359974	0.20022	-0.458	-0.39243	0.577022	0.151988	-0.27458	-0.39001
0.463037	0.045783	0.30993	-0.19684	-0.65934	0.503053	0.239982	-0.57443	0.229605
-0.1579	-0.06317	-0.03535	0.081407	0.127436	-0.1284	-0.2041	-0.00743	0.086283
0.062254	0.107624	-0.17455	0.046211	0.127503	0.072902	-0.40176	0.092025	0.063244
-0.25002	0.025651	-0.20721	0.09814	0.177024	-0.11338	-0.28664	-0.05239	-0.13155

-0.12622	-0.18856	0.104038	0.13131	0.249462	-0.31399	-0.21469	0.145739	0.163336
-0.16817	-0.26395	-0.15631	0.083518	0.223186	-0.26126	-0.39923	0.159627	0.211362
-0.18632	-0.18268	-0.12984	0.282224	0.158808	-0.30127	-0.2936	-0.22239	0.000862
-0.26426	-0.05521	-0.08941	0.03296	0.319763	-0.19131	0.072645	0.227041	-0.10487
0.339499	0.473833	0.187301	-0.06492	-0.3982	0.411643	-0.25636	-0.34068	-0.08403
-0.08637	0.312721	-0.05285	0.199619	-0.10551	0.239504	-0.37397	-0.26581	-0.0404
0.303231	0.215622	0.402891	0.000284	-0.2663	0.248193	-0.14329	-0.34338	-0.01444
-0.09611	0.296035	-0.10188	-0.09649	-0.15274	0.115135	-0.28537	-0.13802	-0.08045
-0.33203	0.080995	0.249817	0.058894	0.226278	-0.27554	0.145993	0.121396	-0.15224
0.004969	0.141677	-0.20101	0.15404	0.0407	-0.1942	-0.28089	-0.08197	0.189825
0.246825	-0.13533	-0.37863	0.400781	0.216783	-0.13797	0.189169	0.327623	-0.31693
-0.2318	-0.08064	-0.16166	-0.14309	0.183058	0.159233	-0.38393	0.098755	0.082542
-0.1933	-0.08018	0.048735	0.255628	0.235993	-0.14692	-0.35783	-0.09998	0.35819
-0.35854	-0.17374	-0.21833	0.005125	0.333938	-0.21131	-0.22692	-0.06747	-0.1469
-0.03139	0.169078	-0.11021	-0.0136	-0.02691	-0.12639	-0.19892	-0.17817	0.01916
-0.32301	0.163706	-0.2322	0.006591	0.145804	-0.12763	-0.45945	0.026786	0.142797
0.018841	-0.07566	-0.01206	0.082112	-0.11847	0.046974	-0.00058	0.069484	0.24799
-0.24621	0.080096	0.263423	-0.05666	0.013422	-0.36073	-0.27239	-0.17683	0.121819
-0.28984	-0.01294	-0.17074	-0.18904	0.2162	-0.02828	-0.38142	-0.06832	-0.09249
-0.21659	0.278124	-0.09364	0.062763	0.070386	0.04812	-0.32007	0.05346	0.180343
-0.03597	-0.03416	-0.23318	0.104902	0.081701	0.027982	-0.34344	-0.0808	0.19326
-0.28924	0.179625	-0.32929	-0.0875	0.038203	-0.09214	-0.41834	0.109481	0.257029
-0.35736	0.042811	-0.24898	-0.32379	-0.09058	0.038021	-0.36059	0.218449	0.269879
-0.17319	-0.09709	-0.16799	-0.04718	0.274698	-0.32036	-0.24145	0.178714	0.083532
-0.17503	0.290765	-0.32365	0.066262	0.107801	-0.03163	-0.40138	-0.00096	0.197727
-0.1341	-0.01184	-0.09828	0.297118	0.064946	0.00736	-0.11722	-0.1984	0.261834
-0.32876	0.064052	-0.21956	0.09329	0.200233	-0.20119	-0.24447	0.081697	0.011858
-0.40179	-0.11089	-0.05555	0.20845	0.466001	-0.45417	0.224752	0.458838	-0.13738
0.182654	0.394056	0.238567	0.172246	0.018989	-0.47955	-0.36973	-0.1238	0.367376
-0.24493	-0.00764	0.01466	0.270059	0.183644	-0.22555	-0.28056	0.218794	0.363662
-0.27897	-0.25054	0.093472	0.13268	0.119234	-0.0278	-0.34975	0.240358	0.346215
-0.25022	-0.09117	0.265542	0.383936	0.034843	-0.16381	-0.09247	-0.23575	0.318387
-0.12107	-0.2735	0.28101	0.116116	0.215859	-0.44409	0.165945	0.12393	0.14853
-0.0996	-0.1897	0.096466	0.214789	0.26049	-0.26987	-0.2876	-0.0929	0.190168
-0.14608	-0.2391	-0.06078	0.22101	0.252881	-0.42529	-0.40313	0.156897	0.147888
0.041268	0.038991	0.175714	0.174688	0.041959	-0.2961	-0.35004	-0.03114	0.351667
0.045931	-0.09325	-0.13739	0.185936	0.018786	-0.13123	-0.46028	-0.08468	0.154542
-0.1977	-0.10644	-0.09798	0.271178	0.1707	-0.2606	-0.44574	0.059548	0.302303
-0.10596	-0.18089	-0.20374	0.185097	0.199179	-0.2093	-0.37524	-0.03802	0.100001
-0.14489	-0.16323	-0.13247	0.127089	0.004045	-0.24525	-0.37863	0.044308	0.171397
-0.09312	-0.19835	-0.30638	0.31648	0.119036	-0.09347	-0.36341	0.035605	0.316509
0.046837	0.09222	0.034036	0.014343	0.368037	-0.40445	-0.38464	0.145542	0.029702
-0.1996	-0.07349	-0.20949	0.174976	-0.00594	-0.18213	-0.41836	-0.19302	-0.10906
-0.09321	-0.06542	-0.29882	0.08327	0.05074	0.006249	-0.4404	-0.16061	0.047365
-0.28364	-0.0808	-0.19573	-0.03313	0.256021	-0.18436	-0.3146	0.029028	-0.09345
0.022892	0.273885	0.212932	0.288502	0.253428	0.173049	0.160456	0.144484	0.237262
-0.20063	0.044679	-0.17265	0.09383	0.017399	-0.05144	-0.35825	0.043863	0.174615
-0.16539	-0.05977	-0.11101	0.02857	0.103321	0.123001	-0.28138	-0.03087	0.138684
-0.24871	-0.12869	-0.07686	0.063932	0.232383	-0.17364	-0.30765	-0.15612	0.022351
0.017555	0.20146	-0.06325	0.029887	-0.10197	0.175232	-0.34722	-0.04792	0.017812

-0.12045	0.108569	0.067204	-0.04998	0.056973	-0.25017	-0.35646	-0.13304	-0.05162
-0.16392	-0.1279	-0.31465	0.154564	0.165225	-0.11713	-0.15108	0.117517	0.062242
-0.1522	0.121643	-0.25039	0.257402	0.162432	-0.01136	-0.41965	0.047808	-0.0175
0.210796	0.152914	0.265257	0.293051	0.01051	-0.52788	-0.35835	0.006197	0.193446
-0.07587	0.000748	0.022634	0.44923	0.650906	-0.52114	0.52856	0.702977	-0.48893
0.073554	0.207393	-0.32494	0.071831	0.359559	-0.00446	-0.40795	0.110377	0.128606
-0.11145	-0.28181	-0.0254	0.141418	0.396633	-0.34425	-0.34637	-0.10721	-0.06074
0.142974	-0.04841	-0.00333	0.16915	-0.21775	-0.17089	0.10604	0.075192	-0.27511
0.407919	-0.23889	0.096629	-0.05326	-0.21815	-0.20058	0.133208	0.038043	-0.43163
0.005841	-0.03875	-0.13937	0.301554	0.080711	-0.03364	-0.14698	-0.22692	0.029978
0.395152	-0.071	-0.02788	0.061925	-0.20403	-0.14702	0.153877	-0.01551	-0.35864
-0.33265	-0.14153	-0.08373	-0.33154	0.317374	-0.36088	0.031876	0.39094	-0.29576
-0.10828	-0.18512	-0.05729	-0.05119	0.102029	-0.0404	-0.22108	0.103315	0.201789
-0.10235	-0.09038	-0.19382	0.173439	0.005472	-0.12278	-0.36001	0.209255	0.285096
-0.16243	0.000375	-0.13961	0.284726	0.154313	-0.37191	-0.39737	0.374718	0.437065
-0.10067	0.081025	-0.05399	0.012219	-0.0196	-0.08302	-0.31541	-0.04564	0.164789
0.051465	0.180771	-0.19554	-0.09019	0.065005	-0.08309	-0.37108	-0.02359	0.019692
-0.11573	-0.13051	-0.34559	0.15816	0.121776	0.022249	-0.49485	0.183741	0.138658
-0.12644	0.203754	-0.21062	0.111654	0.066929	-0.05248	-0.42452	0.063843	0.214088
0.091454	0.174677	-0.24729	0.031253	0.12221	-0.03418	-0.41922	0.153608	0.237234
-0.00617	0.072034	-0.1858	-0.00345	0.167021	0.165461	-0.36876	-0.10131	0.104902
-0.10426	0.087946	-0.16917	-0.0029	0.168668	-0.04539	-0.35871	-0.01215	0.180436
0.053803	0.363254	-0.17596	-0.02128	-0.01829	-0.0029	-0.32813	-0.03312	0.025942
-0.00914	0.152936	0.199951	-0.13726	-0.23964	-0.29655	-0.14667	-0.1505	0.197095
-0.02722	0.20518	-0.03278	-0.16615	0.248117	-0.00766	-0.26466	0.195938	0.113961
0.291493	0.048926	-0.09625	-0.02386	0.054723	0.098778	-0.2434	0.08788	0.094183
-0.01766	-0.06548	-0.12296	-0.08279	0.178719	-0.1451	-0.43816	-0.04308	0.245442
-0.08149	-0.1151	-0.12033	0.150167	0.01724	-0.36321	-0.52192	0.219326	0.314864
-0.0365	0.048537	-0.07609	-0.16583	-0.17504	0.059295	-0.36603	-0.09693	0.317578
0.227629	0.054806	-0.28427	-0.01315	0.205962	-0.06998	-0.35244	0.024694	0.094258
0.100075	0.064569	0.239001	-0.19796	0.285475	-0.602	-0.46832	0.133365	0.092739
0.182792	0.30025	0.350394	-0.00709	0.134117	-0.51759	-0.35745	-0.14177	0.450635
0.21867	0.202377	0.350177	0.026531	-0.03988	-0.56799	-0.41448	-0.13839	0.415458
0.085643	0.188737	0.366442	0.042417	-0.03478	-0.56948	-0.4488	0.092613	0.378572
0.013761	0.159398	-0.1129	-0.0401	-0.04305	-0.30439	-0.40645	-0.02673	0.3464

TCGA-OE-A	TCGA-PZ-A	TCGA-RB-A	TCGA-RL-A	TCGA-US-A	TCGA-US-A	TCGA-YH-A	TCGA-YY-A	TCGA-2J-A
-0.42875	-0.32692	-0.13466	-0.26397	0.349319	0.210988	-0.08127	-0.2783	0.055238
-0.48961	-0.04012	-0.60091	0.10779	0.610676	-0.3166	-0.01976	-0.06832	-0.15885
-0.18666	0.041389	-0.08216	-0.34257	0.343617	0.052164	-0.09072	-0.01565	0.056376
-0.00324	0.315927	-0.5079	0.283333	0.238692	0.30985	0.002678	-0.38182	0.080963
0.046614	0.053678	-0.24557	0.056339	0.499001	-0.06557	0.447518	-0.01464	0.098023
-0.12596	0.031579	-0.16103	-0.12072	0.194585	0.108743	-0.11482	-0.10319	-0.10466
0.011901	-0.03689	0.044256	-0.02678	0.402783	0.231074	-0.37794	0.279939	-0.194
0.058602	0.069298	-0.15172	-0.28696	-0.04587	0.177356	-0.02043	0.168242	-0.21412
0.05357	0.085033	-0.19994	0.062731	0.026177	0.316266	0.002299	0.202475	-0.18276
0.147447	0.184376	-0.1259	0.331054	0.309843	0.171377	0.181137	0.16586	-0.30414
0.206882	0.15524	-0.4247	-0.2795	0.240164	0.551366	0.214519	0.327047	-0.33249
0.13559	0.12727	-0.46857	-0.08851	0.267483	0.291625	0.224722	0.235262	-0.23416
-0.20042	0.136355	0.146122	-0.26358	0.23567	0.13023	-0.07073	0.109144	-0.43576
0.038282	0.218112	0.0821	0.106587	0.254291	-0.01374	0.211063	-0.11429	-0.04339
-0.02504	0.308254	-0.32186	0.152779	0.568827	-0.57061	0.230079	-0.16789	-0.41786
-0.16785	-0.33111	-0.35833	0.136093	0.634011	-0.5691	0.014251	-0.45801	-0.24216
0.02656	0.318136	-0.06621	-0.16851	0.159619	-0.30424	0.07221	0.053244	0.384488
0.139248	-0.00706	-0.26904	-0.13415	0.134861	-0.15948	-0.37689	-0.1632	-0.09857
0.330109	0.245538	-0.14689	0.183354	0.253073	-0.15684	-0.31564	-0.03583	0.058079
0.087987	-0.17048	-0.04926	-0.1422	0.243732	0.273048	-0.17418	0.467118	-0.02646
-0.35359	-0.28737	0.042622	-0.15716	-0.34824	-0.13165	-0.09289	0.149297	0.192229
-0.25382	-0.42839	0.161609	-0.35367	-0.18203	0.397765	-0.15287	0.010112	0.32676
0.334823	0.220423	-0.17008	-0.02808	-0.29208	0.021635	-0.00418	0.047997	0.198338
-0.226	0.26582	-0.41591	0.123177	0.422653	-0.26442	-0.12462	-0.28305	-0.19995
0.224857	0.209147	0.027044	0.184008	-0.27954	-0.17118	0.208686	0.135544	0.089586
-0.15003	0.16151	-0.47294	0.352656	0.516936	-0.32493	0.192627	-0.05353	-0.08584
-0.47849	0.051974	-0.05954	-0.55097	-0.43002	0.022264	0.468196	0.282376	0.315462
-0.13592	-0.19785	0.005502	-0.13255	0.246854	0.158572	0.108209	0.110634	-0.1817
-0.13878	0.200966	-0.00274	0.247543	0.406152	0.039892	-0.37665	0.129703	-0.43403
-0.24387	0.355818	0.425655	-0.09993	0.032254	-0.10369	0.011575	-0.17899	0.051562
0.078861	0.221987	-0.36723	0.511551	0.538164	-0.51202	-0.24885	-0.59153	-0.26369
0.236342	0.242755	-0.35416	0.486324	0.387267	-0.25691	-0.13233	-0.01091	-0.26623
-0.24359	-0.04335	0.016763	-0.19645	-0.10789	-0.19615	0.182648	-0.1419	0.230595
-0.24107	-0.10803	0.14826	-0.23277	-0.05614	-0.13551	0.233075	-0.23606	0.305413
-0.27052	-0.05748	0.117151	-0.03985	0.035729	-0.22378	0.185296	-0.16921	0.09094
-0.03548	0.348172	-0.17144	0.084134	0.26232	-0.13543	0.010475	0.072861	0.072872
-0.31914	0.029445	-0.09349	0.28502	0.425902	-0.31831	-0.38759	-0.3013	-0.17754
0.080836	0.271617	-0.40127	0.221918	0.18507	0.155155	-0.00531	0.257327	0.213824
0.175428	0.590431	-0.36171	-0.1943	0.585373	0.445375	-0.10528	0.334309	-0.28133
0.085731	0.368208	-0.37013	-0.39918	0.031852	0.325704	0.035302	0.458197	0.276426
-0.16373	0.397733	-0.28176	-0.10082	0.289433	0.113897	0.055216	-0.01558	0.355939
0.078037	0.392017	0.018273	0.12613	0.651919	0.571387	-0.06369	0.1968	-0.47901
-0.22079	-0.21861	0.109414	0.485507	0.636676	0.12173	-0.4761	-0.15844	-0.4856
-0.04397	-0.29912	0.445663	0.276012	0.37576	0.345799	-0.4301	-0.23602	0.35311
-0.16367	-0.2633	0.364671	0.450886	0.604992	-0.31497	-0.34217	-0.12529	-0.30795
0.186213	0.265887	0.02916	0.158055	0.410949	0.470701	-0.03571	0.234316	-0.25654
0.34171	0.314671	-0.2126	0.385565	-0.22621	-0.32084	0.065788	0.046684	-0.22556
0.095688	-0.05679	-0.12366	0.073673	0.065857	-0.16768	-0.01953	0.068506	0.052006
0.23229	-0.02823	-0.28734	-0.08779	0.070713	-0.06514	-0.12285	0.329515	0.200792

-0.02224	0.119837	-0.20737	-0.16491	0.368401	0.103607	-0.23315	-0.07197	-0.16642
0.107222	-0.16104	-0.17661	0.302886	0.466353	0.190943	-0.51426	-0.2166	-0.01937
-0.34934	-0.25725	-0.04289	0.091275	0.432139	-0.12022	-0.21943	-0.25926	-0.06956
-0.19448	-0.32739	0.002155	0.397245	0.529696	0.243265	-0.53942	-0.35036	-0.37576
-0.34216	-0.35704	0.136724	-0.19204	0.379389	0.061597	-0.21074	-0.36083	-0.00226
0.027939	0.232109	-0.24576	0.110169	0.419733	0.209899	0.048995	0.076991	-0.30615
0.110773	0.161111	-0.24908	0.33041	0.262789	0.284259	-0.34157	0.077907	-0.26117
0.276899	0.149059	-0.18095	0.398081	0.201137	0.135291	-0.24995	-0.15067	-0.10362
0.259875	0.181945	-0.27756	0.226788	0.326456	0.098366	-0.17527	-0.2086	-0.24517
-0.22941	-0.08316	-0.13249	0.446591	0.446069	0.039262	-0.2515	-0.2798	-0.15839
-0.33138	-0.36054	0.284228	0.194822	0.372078	0.142102	-0.38297	-0.29421	-0.44729
0.215689	-0.1424	0.455137	0.508098	0.302138	-0.02368	-0.33339	0.262955	-0.41824
-0.06166	-0.38199	-0.07667	0.217671	0.28701	0.164988	-0.22213	0.133897	-0.19363
0.057194	-0.04986	-0.22259	0.428257	0.498048	0.082332	0.168748	-0.15396	-0.04751
-0.07006	0.371306	-0.18538	-0.07634	0.390064	0.216569	-0.02685	0.092968	0.086628
-0.06863	0.335984	-0.47177	-0.04313	-0.0249	-0.03837	-0.17449	-0.08221	0.458997
-0.12446	-0.07878	-0.46677	-0.03461	0.459015	-0.55775	0.593407	-0.40586	0.336226
-0.03781	0.069072	-0.28915	0.136291	0.266987	-0.24605	0.400656	-0.30145	0.184555
-0.31113	-0.12532	-0.12211	-0.31418	0.144654	0.204617	-0.14971	-0.01322	-0.3035
-0.0474	0.133291	-0.12236	0.329147	0.419593	-0.13263	-0.38098	-0.18473	-0.03178
0.078069	-0.37629	-0.52468	0.138145	-0.0997	-0.23799	-0.26361	-0.24709	0.312502
-0.32793	-0.38865	0.191013	0.371922	0.520453	-0.00581	-0.43234	-0.38932	-0.53936
0.118689	0.002164	0.054226	0.343203	0.306788	0.24927	-0.35629	-0.33322	-0.30679
0.029393	0.047868	-0.49397	-0.26158	0.032319	0.257987	-0.14773	0.263934	0.246914
0.361701	0.195039	0.07904	0.205645	0.241702	0.219419	0.371267	-0.02561	0.252807
0.111304	0.415495	-0.00033	0.160384	0.435968	0.4481	-0.01444	0.227839	-0.38511
0.27803	0.53385	0.17133	0.162259	0.48882	0.402214	0.038843	0.312376	-0.12441
0.430196	-0.26745	0.422969	0.302355	0.469766	0.519419	-0.22336	-0.18974	0.402407
0.048311	0.312185	-0.33365	-0.08971	-0.07453	0.280566	-0.24481	0.259253	-0.21679
0.190682	0.32487	0.266678	0.099862	0.012829	0.111886	0.115066	0.525035	0.020427
0.258283	0.328936	-0.19935	0.367705	0.48926	0.459608	0.031091	0.2406	-0.27347
0.237457	0.295571	-0.23124	0.308733	0.491007	0.484948	-0.06326	0.14063	-0.32479
0.190385	0.420691	-0.07567	-0.13251	0.381363	0.426284	0.102484	0.332199	-0.03324
-0.22624	0.1014	0.33534	-0.12002	0.077951	-0.01816	-0.2832	-0.0224	0.150341
-0.14335	-0.00074	-0.1357	-0.06751	0.272923	0.098931	0.023208	-0.16125	-0.28021
0.590908	-0.43143	0.233672	0.610516	-0.34575	-0.57826	-0.48028	0.334924	-0.38729
-0.08545	-0.24233	-0.14769	-0.06474	-0.47849	-0.28408	0.05068	0.255103	0.311699
0.24036	-0.23809	-0.1534	0.23548	0.109543	-0.38442	0.050506	0.321844	0.069408
0.439489	-0.18663	-0.3425	0.187942	-0.01708	-0.04909	0.156384	0.05199	0.550102
-0.23015	-0.13698	0.367735	0.203958	-0.4533	-0.31665	-0.18274	0.168128	-0.04544
0.024157	0.273147	0.296101	0.160824	0.425601	-0.62997	-0.011	0.207952	0.257386
-0.48783	-0.38393	0.142628	0.198403	-0.08313	-0.65382	-0.26674	-0.55848	0.32316
-0.08316	0.215864	-0.18473	-0.00065	0.290825	0.063296	-0.37956	0.02883	-0.31562
0.474432	0.33463	-0.19946	-0.10215	-0.15426	0.267293	0.256244	0.43895	0.274461
0.145322	-0.07684	0.038142	-0.03118	-0.12542	-0.1615	-0.0649	0.048825	0.438753
0.186672	-0.18382	0.147625	-0.00529	-0.08343	-0.232	-0.0247	-0.20601	0.577552
0.461772	-0.12296	-0.4238	-0.38698	-0.27802	0.415676	0.196579	0.205141	0.498884
-0.23148	0.046563	0.023574	-0.05214	0.04251	-0.08406	0.05833	-0.12043	0.044838
-0.38636	-0.13411	0.004759	-0.36452	0.151442	-0.07554	-0.12818	-0.30978	0.101114
-0.13961	-0.01756	-0.05275	0.13386	0.060356	-0.11335	-0.06489	-0.05029	-0.25058

0.012154	0.17485	-0.02623	0.36061	0.364394	-0.33603	0.068893	-0.2711	-0.17882
-0.22341	0.10544	-0.00638	0.138767	0.336695	-0.14695	-0.21241	-0.34003	-0.04237
-0.28924	0.056357	-0.09203	-0.30783	0.017176	0.088198	-0.20361	0.008891	-0.29019
-0.01222	-0.03756	0.098309	0.319742	0.188043	-0.22226	-0.15001	-0.17407	-0.08641
-0.15875	-0.24592	0.096716	-0.23237	-0.2573	0.022326	0.122827	-0.19702	0.326674
-0.22544	-0.20823	0.256108	-0.14734	-0.06753	-0.01846	-0.05075	-0.22311	0.12311
0.111036	-0.05238	-0.09416	-0.35845	-0.31843	0.044641	0.180498	0.079716	0.070746
-0.35404	-0.27451	0.04694	-0.34346	-0.1469	-0.0265	-0.02319	-0.19819	0.121363
-0.0964	-0.09134	-0.10638	0.184572	-0.23257	-0.02603	-0.28368	-0.10501	0.044385
-0.16673	0.117054	0.141185	-0.15902	0.138982	0.063771	-0.02859	-0.10902	0.061618
0.111242	0.083529	-0.17894	0.182468	0.395241	0.40525	-0.3975	0.276196	-0.32854
-0.26428	-0.41321	-0.20941	-0.23751	0.066849	-0.11126	-0.33259	-0.33493	0.104894
-0.16128	0.019671	0.074566	-0.20056	0.091031	0.176587	-0.10822	0.02172	-0.09532
-0.20048	-0.1514	0.108591	0.03777	0.028366	-0.09037	-0.1077	-0.07819	-0.24712
-0.21426	-0.12187	0.142533	-0.13412	0.018827	-0.07766	0.038095	-0.12632	-0.04742
-0.31664	-0.33454	0.1917	-0.37845	0.202773	-0.20186	-0.21072	0.043894	-0.1925
-0.17865	-0.079	0.267542	-0.17765	0.249146	-0.09119	0.093093	-0.11691	0.117805
-0.0856	-0.1083	0.034436	-0.07446	0.270154	-0.23255	0.232853	-0.11809	-0.06607
-0.39819	-0.16293	0.239455	-0.20388	0.233062	-0.3839	-0.01149	-0.32418	-0.05288
-0.19051	0.065566	0.338461	-0.08255	0.158212	-0.0746	0.166511	-0.27435	0.154192
-0.07077	0.009066	-0.17558	-0.20411	0.146845	0.093051	0.053697	-0.06771	0.015175
-0.38297	-0.17984	0.19101	-0.20426	0.328794	-0.28504	0.129085	-0.43983	0.227888
-0.30576	-0.16969	0.002606	-0.16502	0.532935	-0.4319	0.418935	-0.52673	0.255704
-0.21883	-0.00049	0.11414	0.424188	0.384212	-0.506	-0.20308	-0.4356	-0.25611
-0.48504	-0.14647	0.370756	-0.33971	0.062528	0.003353	-0.03106	-0.34418	0.126517
-0.27102	0.058248	0.415134	-0.02593	0.047331	0.042122	0.138884	-0.03387	0.011523
-0.30678	-0.04591	0.301004	-0.06956	0.191953	-0.10172	-0.07537	-0.07757	-0.15753
0.165716	0.18095	-0.05334	0.398343	0.558677	-0.26254	-0.27424	-0.36299	-0.10211
0.027477	0.241481	-0.17995	0.335239	0.229913	-0.41936	-0.28014	-0.27601	-0.4073
-0.11049	0.133119	-0.08682	-0.05403	0.296748	-0.21259	0.056152	-0.1324	0.044751
-0.23927	0.035261	0.179204	-0.06839	0.070129	-0.16463	0.085103	-0.33272	0.139708
0.016662	-0.02059	0.144492	-0.15717	-0.01512	0.085485	0.156499	0.146402	0.068595
0.181892	-0.0089	-0.03533	0.174617	0.246163	0.092109	0.330396	0.310199	0.13127
-0.29734	-0.0402	-0.07495	0.035492	0.238094	-0.17917	-0.06655	-0.10119	-0.14186
-0.06159	0.280732	-0.28219	0.42758	0.438741	-0.37607	-0.0774	-0.35871	-0.17927
-0.12678	0.13327	-0.30757	0.038281	0.224136	-0.32006	-0.03188	-0.13903	-0.16703
-0.29834	-0.04138	0.074147	-0.21986	0.075582	-0.10494	-0.21232	-0.18362	-0.11283
-0.32398	0.19919	-0.07488	0.019725	0.224436	-0.02445	-0.23953	-0.21357	-0.1172
-0.25941	0.055685	-0.28469	-0.13953	0.268498	-0.10884	-0.24364	-0.23842	-0.14158
-0.21451	0.112506	-0.09969	-0.12403	0.220313	-0.00933	-0.07332	-0.30718	0.065938
-0.30035	0.046745	0.351558	0.014729	0.247585	-0.19858	-0.1458	-0.28062	-0.08645
0.1025	0.320689	-0.30376	0.550689	0.41456	-0.43913	-0.36855	-0.42303	-0.52889
-0.27143	-0.07198	0.075723	-0.30082	-0.13947	0.107279	-0.13758	0.05908	-0.10359
-0.37537	-0.10857	0.157302	-0.19848	0.066379	-0.0629	-0.23255	-0.34401	0.109898
-0.21145	-0.10502	-0.06616	-0.22534	0.020997	-0.11562	-0.07332	-0.01482	-0.08621
0.044725	0.176797	0.224745	0.052485	0.076212	0.102445	0.137992	0.13337	0.049948
-0.25444	0.048811	0.115243	-0.15807	0.245103	-0.21042	0.029417	-0.37171	0.143424
-0.2413	-0.11143	-0.16157	-0.30881	0.069554	-0.02254	-0.23914	-0.1628	0.087589
-0.19534	-0.03858	-0.00073	-0.22637	0.155317	0.115753	-0.04694	-0.0291	-0.2956
-0.20124	-0.19585	0.132144	-0.16515	0.07593	-0.21709	-0.2036	-0.32943	0.162856

-0.22674	-0.02559	0.155259	-0.0423	0.225454	-0.12522	0.098857	-0.13818	-0.10554
-0.18017	-0.06211	-0.00446	-0.29617	0.06727	0.189756	-0.20758	0.056513	0.053922
-0.30951	-0.02599	0.213802	-0.2673	0.116074	0.081162	-0.26775	-0.2525	0.180205
0.251235	0.358033	-0.22432	0.456307	0.306576	-0.48706	-0.29357	-0.36551	-0.36179
0.107168	-0.18538	0.653451	0.344571	0.484522	0.355211	-0.56328	0.269538	-0.38857
-0.19028	0.083265	0.078457	-0.07069	0.236875	0.143217	-0.36725	-0.24227	-0.20712
-0.40695	-0.14258	0.474901	-0.03273	-0.01798	-0.02591	-0.48561	0.089465	-0.43251
0.235838	0.211154	-0.01124	0.11406	-0.08143	-0.1551	0.1001	0.110166	-0.19312
0.303263	0.246078	-0.15329	0.3406	-0.21943	-0.31161	-0.05086	-0.0349	-0.34489
-0.16304	0.177159	0.030545	-0.09535	0.010477	-0.0829	-0.07544	0.130421	-0.02534
0.212786	0.203131	-0.05413	0.153863	-0.15589	-0.27708	-0.07722	0.163242	-0.16831
-0.2154	-0.04902	0.009969	0.261732	0.412136	-0.51707	-0.00938	-0.35477	-0.16951
-0.32586	0.061074	0.017029	0.014865	0.247791	-0.10575	-0.01021	-0.16427	-0.02831
-0.30009	0.100793	0.163923	-0.17816	0.1891	0.0806	0.07293	-0.12056	-0.07441
-0.21341	0.126496	-0.26899	0.298063	0.437339	-0.36159	-0.18872	-0.40895	-0.19502
-0.26201	-0.02098	0.204865	-0.11908	0.238506	-0.11926	0.108477	-0.20454	0.078922
-0.35522	-0.03296	-0.01369	-0.18171	0.116065	-0.1097	-0.22157	-0.30213	0.103761
-0.3961	-0.18855	0.207474	-0.32038	0.111447	-0.0616	-0.31701	-0.28773	0.088818
-0.21725	0.063141	0.233896	-0.13841	0.191006	0.047682	-0.09683	-0.21607	0.278043
-0.38575	-0.16944	0.050933	-0.23923	0.211154	0.005528	-0.33253	-0.35808	0.045599
-0.3349	-0.23377	0.181043	-0.23085	0.181355	-0.32907	-0.07652	-0.2717	0.176701
-0.33893	-0.17432	0.238395	-0.18072	0.17087	-0.06525	-0.05088	-0.24738	0.065718
-0.3724	-0.229	0.188863	-0.2691	0.082135	-0.20373	0.057283	-0.24272	0.158093
0.049609	0.006117	0.005352	0.087239	0.368387	-0.30589	0.365884	-0.21717	0.066916
-0.19601	-0.06756	0.154767	-0.04242	0.235941	-0.27991	0.078987	-0.34189	0.162052
-0.08973	-0.13556	0.176589	-0.17459	0.190745	-0.14182	-0.09436	-0.12251	0.161128
-0.42265	-0.20315	0.122894	-0.29371	0.073218	-0.07713	-0.10898	-0.19689	0.085152
-0.26368	0.022187	-0.13726	-0.1551	0.316475	-0.18577	-0.02011	-0.25604	-0.0024
-0.34079	-0.1763	0.293471	-0.28125	0.204049	-0.05941	0.161317	-0.2046	0.197975
-0.31227	-0.21399	0.11195	-0.15628	0.25202	-0.09155	-0.36484	-0.33536	0.150967
0.054767	0.37166	-0.37804	0.539017	0.564754	-0.56115	-0.4631	-0.48222	-0.49466
0.279374	0.404206	-0.43874	0.478097	0.397734	-0.54018	-0.27659	-0.35493	-0.44699
0.233682	0.379661	-0.42472	0.519723	0.449108	-0.58761	-0.35644	-0.45973	-0.44793
0.299969	0.253384	-0.47378	0.50371	0.502524	-0.62138	-0.32374	-0.39414	-0.41099
0.074503	0.293467	-0.30581	0.233788	0.324456	-0.40986	0.142503	-0.36514	-0.1215

TCGA-2J-A	TCGA-2J-A	TCGA-2J-A	TCGA-2J-A	TCGA-2J-A	TCGA-2J-A	TCGA-2J-A	TCGA-2L-A	TCGA-2L-A
-0.17819	0.008181	-0.00814	-0.23818	0.033504	0.413221	-0.52854	-0.21823	0.049349
-0.17593	-0.16862	-0.31344	0.165144	-0.46392	0.294496	-0.25747	-0.49155	-0.07179
0.134287	-0.19644	0.200556	0.142194	0.450175	-0.23097	-0.00916	0.26522	-0.41181
-0.13015	0.287396	0.239595	0.162127	-0.33386	0.367998	0.081592	-0.17456	-0.12868
0.156334	0.258464	0.137521	0.414726	0.104631	0.171055	-0.16261	0.058887	-0.45058
-0.16399	0.069903	-0.15875	0.239476	0.091283	-0.09634	0.306839	0.23365	-0.14794
0.234369	0.061874	-0.51572	-0.15468	0.279099	-0.31441	-0.60456	0.175764	0.123757
0.017182	-0.0813	-0.03114	0.253668	0.044132	-0.30204	0.117539	0.042396	-0.18907
0.315143	-0.03379	0.136787	0.334349	0.192077	-0.42839	-0.06793	0.193045	-0.12151
0.007469	-0.12774	0.349385	0.160661	0.094783	-0.37103	0.043333	0.021741	0.039009
0.264387	-0.38952	0.126089	0.438039	0.02932	-0.45073	0.114782	0.49004	0.088896
0.270826	-0.12044	0.000244	0.391503	0.197595	-0.44342	0.28794	0.325184	0.049903
-0.16818	0.145755	-0.0822	0.266893	-0.2598	-0.25641	-0.1216	-0.10449	-0.32497
0.336085	-0.20856	-0.07888	0.317057	0.031462	-0.27296	-0.02422	0.069333	-0.35193
0.244519	0.063735	0.550174	0.705198	0.073634	0.007814	0.07773	-0.0188	-0.54269
0.031939	-0.19028	0.047859	0.284872	-0.33053	0.487529	-0.04983	-0.36959	0.015886
0.398854	0.193716	-0.11666	-0.00154	0.085454	-0.11534	-0.24434	0.161839	-0.31083
-0.23779	0.013141	-0.25888	0.019175	-0.37181	-0.14278	-0.08736	-0.22433	-0.23314
-0.26058	-0.23663	-0.35048	-0.35701	-0.2829	0.078295	-0.43129	-0.10504	0.205879
0.299478	-0.11323	0.109132	-0.02569	-0.10645	0.151389	-0.21637	0.206413	-0.22836
0.108406	-0.05296	-0.10032	-0.4034	0.086598	0.174601	-0.0085	0.019402	-0.22569
0.381298	0.154893	0.179531	-0.16933	0.344419	-0.11303	0.167403	0.291479	-0.55712
-0.03906	-0.09424	-0.46424	-0.2657	0.19907	0.026083	0.041339	-0.24697	0.001188
-0.20469	-0.02121	-0.06485	0.308016	-0.29033	0.195901	0.164597	-0.38645	-0.2252
0.054624	-0.10138	-0.05549	-0.01669	0.324432	-0.10443	0.358315	0.074093	0.286275
-0.08045	-0.09115	0.471192	-0.00768	0.069364	0.090407	0.077272	-0.30833	0.233963
-0.33713	-0.21561	0.095117	0.009084	0.055212	-0.27853	0.237028	0.114314	0.241942
-0.02192	-0.18248	0.107093	0.244492	-0.22085	-0.09096	0.311003	0.183534	0.313661
-0.22528	0.273295	-0.5139	0.263452	-0.1229	-0.28892	0.252253	-0.16525	0.161271
0.072765	0.226579	-0.0923	0.077258	0.236967	0.030447	0.147181	0.107933	-0.46674
-0.1773	0.284918	-0.53915	-0.08908	-0.42171	0.407113	0.377879	-0.44949	-0.46006
-0.17359	-0.05764	-0.11997	-0.0735	-0.28265	0.337135	0.038304	-0.30955	-0.44247
0.272086	0.047537	0.188478	0.01099	0.25943	0.032076	0.338786	0.285411	-0.24988
0.133504	0.284809	0.037054	0.093365	0.351125	-0.07355	0.319449	0.279629	-0.3216
0.188385	0.194564	0.085725	-0.00224	0.280225	0.009295	0.375011	0.1808	-0.21932
-0.09001	-0.09838	0.06665	0.169743	0.104663	-0.20868	0.316691	0.212701	0.060644
-0.08558	0.371967	-0.52358	-0.18034	-0.38424	0.303772	0.579754	-0.08228	0.019023
-0.47751	0.005509	-0.11582	0.182349	-0.02059	0.049152	0.051024	0.009111	0.019603
-0.02221	-0.30319	-0.26392	0.068736	-0.20197	-0.29867	-0.37128	0.244464	-0.12798
-0.20532	-0.2354	-0.41923	0.061842	-0.23188	-0.11024	-0.13501	0.398498	0.070564
-0.48134	0.077666	-0.37663	0.321006	-0.27967	-0.01825	0.21679	-0.08611	0.065766
0.204525	-0.26095	0.064576	0.321484	-0.02294	-0.47116	-0.2499	0.558214	-0.30553
-0.20358	0.001911	0.251877	-0.00927	-0.36057	-0.36169	0.355598	-0.26489	-0.24359
-0.15497	0.51049	-0.51433	0.353174	0.316685	-0.52225	-0.53806	0.690608	-0.34796
0.123951	0.072923	0.21924	0.157268	-0.47106	-0.23885	-0.07846	-0.20275	0.374044
0.196663	-0.13378	0.200299	0.230009	0.130188	-0.41278	-0.27131	0.265061	-0.22763
0.106827	-0.29362	-0.18543	-0.02452	0.071149	-0.10568	0.402131	-0.25672	0.409242
0.047172	-0.0515	-0.0801	-0.15665	0.013139	0.119395	-0.13753	0.08913	-0.18586
-0.03133	-0.05964	-0.31771	-0.17732	0.031913	0.275082	-0.38201	0.2345	-0.15736

-0.03697	0.131311	-0.15723	0.195504	-0.10546	-0.13039	-0.03738	0.008576	-0.1265
-0.37564	0.589529	0.336044	-0.35684	-0.25739	-0.03179	-0.06564	-0.35622	0.048632
-0.15117	0.323273	-0.05731	-0.35339	-0.34544	0.033991	0.148393	0.012944	-0.27331
-0.05143	0.241175	-0.13573	-0.05093	-0.42797	-0.23383	0.344937	-0.07769	-0.09547
-0.17465	0.032614	-0.15055	0.019897	-0.31697	-0.12309	0.216721	0.006355	-0.17132
0.027385	0.147932	-0.17708	0.032488	0.00476	-0.22739	0.305823	0.293747	-0.22138
0.073993	-0.09726	0.355581	0.006826	-0.03585	-0.39881	0.093577	0.149674	-0.17847
-0.09236	0.097492	0.273083	-0.11573	0.155996	-0.21331	0.095687	-0.01751	-0.02218
-0.1536	0.234768	-0.06286	0.248229	-0.0419	-0.41283	0.265357	0.357714	-0.11238
-0.21911	0.353776	-0.0754	-0.06628	0.109563	-0.13112	0.313868	-0.14768	-0.20581
-0.04267	0.248997	0.238637	-0.01262	-0.10745	-0.29119	0.324932	0.016541	-0.14811
-0.27893	0.574094	0.45784	0.405116	0.168408	-0.40964	-0.56177	-0.08823	0.245814
-0.1339	0.305604	0.071045	-0.32021	-0.30891	-0.30897	-0.4341	-0.13112	-0.23536
-0.10137	0.161248	0.052509	-0.28124	0.128414	-0.0124	-0.14104	-0.19703	-0.20859
-0.00964	0.004096	-0.11442	0.148747	0.168372	-0.19303	0.052123	0.053606	-0.0734
-0.21974	-0.16696	-0.27535	0.124253	-0.36321	0.263131	-0.07182	-0.08957	-0.14289
0.383329	0.308965	0.154405	0.346083	0.250686	0.344939	0.070412	-0.27588	-0.36856
0.089164	0.021784	0.214854	0.232299	0.060457	0.344472	-0.39273	-0.18925	-0.12801
-0.04656	-0.00194	0.025664	0.244054	-0.17629	-0.26299	0.284171	0.05187	-0.01295
-0.33791	0.150129	-0.274	-0.21146	-0.42324	-0.00305	0.391366	0.183351	-0.00743
-0.1759	0.505096	-0.56064	0.103491	-0.23243	0.37138	0.315056	-0.20081	0.02574
-0.11189	0.273321	-0.10956	-0.18275	-0.19799	-0.33238	0.542161	0.087989	-0.26306
-0.05655	0.060171	-0.26223	0.157831	-0.13538	-0.26655	0.183136	0.225414	-0.02133
0.505308	0.26221	0.110835	-0.06231	-0.20032	0.135592	-0.42957	0.50909	-0.53569
0.380822	0.009203	0.194889	0.08279	-0.30216	0.212951	-0.52901	0.295179	-0.12158
0.022671	-0.38723	0.17058	0.127111	0.042001	-0.34907	-0.09121	0.247451	-0.04979
0.06879	-0.15353	-0.05875	0.322522	0.000977	-0.02996	-0.14309	0.213903	-0.07975
0.359533	0.011476	-0.49128	0.488706	0.097447	-0.11846	-0.00506	0.456955	-0.54261
0.081296	-0.32343	-0.11822	0.136184	-0.0943	-0.39798	0.231592	0.211825	-0.03344
0.470188	-0.0211	-0.14983	0.335028	-0.00632	-0.43627	-0.40818	-0.2827	-0.49709
-0.01206	-0.20261	0.327331	-0.01047	0.2267	-0.48498	-0.00301	0.158975	-0.12331
0.039647	-0.13867	0.358821	-0.04342	0.233124	-0.46633	0.077485	0.189926	-0.1389
-0.06252	-0.22121	0.098438	0.210781	0.166354	-0.21074	-0.22841	0.383901	-0.12177
0.037451	0.150492	-0.09993	-0.1749	0.381248	-0.42667	0.009173	-0.06707	-0.37606
-0.10451	-0.19461	0.059752	-0.21419	0.014844	-0.12303	0.081546	-0.26027	-0.02053
0.119788	-0.27783	0.29934	-0.50936	-0.42917	-0.00917	-0.29086	0.178585	-0.25158
0.170615	-0.32032	-0.285	-0.45989	-0.19085	0.230408	-0.29364	0.253402	-0.09558
-0.05882	-0.13493	-0.23012	-0.22545	0.296683	0.295922	-0.36193	0.071838	0.021183
-0.15178	0.134674	-0.43065	-0.54117	-0.28905	0.718715	-0.37862	0.253327	-0.11483
0.172169	-0.08124	-0.07879	-0.41414	0.177609	0.214073	-0.34558	-0.04946	-0.23929
0.35281	0.169774	-0.38282	-0.50184	0.508054	0.462979	-0.12402	-0.27261	-0.5762
0.106768	0.224711	-0.4323	-0.70383	-0.11014	0.17048	-0.60305	-0.35045	0.209351
-0.10576	-0.02374	0.045245	0.056661	-0.31151	-0.23703	0.259231	0.028766	-0.05767
-0.10558	-0.24096	0.003142	-0.08386	-0.06102	0.36235	-0.35147	0.383648	-0.01545
0.230595	-0.11369	-0.12074	-0.22601	-0.05144	0.477634	-0.35432	0.021654	0.246483
-0.29199	-0.19037	-0.26405	-0.49568	-0.34196	0.622707	-0.51742	0.102924	-0.13057
0.344059	-0.00714	-0.11552	-0.36613	-0.10621	0.605047	-0.44285	0.229253	-0.19551
0.025701	0.194788	0.03017	-0.02649	-0.02706	-0.06483	0.266243	0.064504	-0.19956
-0.2758	0.220971	-0.04838	-0.31877	-0.1059	-0.1307	0.145704	-0.15245	-0.32835
-0.2024	0.20991	0.053121	0.112625	-0.01837	-0.03511	0.437266	-0.01679	0.042876

-0.0582	0.196544	-0.04676	0.071318	-0.2148	0.125922	0.132757	-0.17977	-0.35688
-0.2701	0.138571	-0.29297	-0.08382	-0.24912	0.071035	0.348205	-0.33654	-0.4056
-0.04534	-0.12888	0.100024	0.242343	-0.06519	-0.21879	0.461398	0.054383	-0.1575
-0.20042	0.244271	-0.02542	0.095761	0.066823	0.054613	0.253156	-0.13504	0.128046
0.128691	-0.20144	-0.18799	-0.39482	-0.18977	0.334556	-0.37094	0.213499	-0.33207
0.013681	0.176706	-0.14839	-0.34881	-0.09055	0.126188	0.032908	-0.02644	-0.29456
0.039498	-0.14862	0.228104	0.166299	0.015433	-0.02653	-0.36708	0.293689	-0.33104
-0.20114	-0.04872	-0.06033	-0.27468	-0.07875	0.034755	-0.101	-0.16819	-0.17355
-0.27464	0.058838	-0.09503	0.023562	-0.33089	0.146045	0.082648	-0.0627	0.234823
-0.07163	0.200661	0.048322	0.162876	-0.10097	-0.15671	0.102722	0.000196	-0.35482
-0.23451	-0.00352	0.155172	0.006809	0.088623	-0.22228	-0.05711	-0.19025	-0.10743
-0.31406	0.083418	-0.1413	-0.10835	-0.3534	0.039431	0.322704	-0.33989	-0.17907
-0.32881	0.035773	0.087863	0.035505	-0.13763	-0.08638	0.03321	-0.10452	-0.44066
0.036239	0.197875	0.055448	0.132812	0.014225	0.018673	0.444021	0.024789	-0.09171
-0.06107	0.207509	0.132883	0.045766	0.147861	-0.08961	0.151551	-0.07148	-0.23189
-0.20728	0.319915	0.12513	-0.25044	-0.07785	-0.25652	0.355583	-0.0579	-0.12574
-0.13762	0.112846	-0.09493	0.280561	0.222212	-0.23591	-0.19869	-0.20382	-0.04132
0.015827	0.322203	0.417363	0.319195	0.402793	-0.18446	0.180884	0.064179	-0.23684
0.331374	0.439453	0.403797	-0.13365	0.102565	-0.28607	-0.0111	-0.19687	-0.33842
-0.29177	0.273359	0.010409	0.13532	0.148467	-0.18309	0.110475	-0.09648	-0.35174
0.001136	-0.00712	0.003545	0.099659	-0.04925	-0.06982	0.10172	0.069479	-0.37078
-0.02649	0.34616	0.046443	0.011215	0.243855	0.029459	0.235082	-0.17952	-0.40422
0.300652	0.407151	0.125663	0.350776	0.345077	-0.04317	0.327814	-0.10633	-0.38991
-0.10159	0.262387	-0.26858	-0.11817	0.007178	0.004385	0.227097	-0.46856	-0.37653
0.006919	0.277327	-0.00411	-0.01123	0.1302	-0.19691	0.057132	-0.01264	-0.47759
0.066034	0.282273	0.077389	0.018104	0.173152	-0.25239	0.11665	0.106034	-0.41675
-0.26168	0.38709	0.001319	-0.13436	0.100267	0.136021	0.362503	-0.07174	-0.20921
-0.33835	0.413626	-0.11037	0.056128	-0.23532	-0.07402	-0.04989	-0.36314	-0.00671
0.152675	-0.21222	-0.26882	-0.03505	-0.39842	0.370135	0.243539	-0.25835	-0.4168
-0.23024	-0.02535	-0.18621	0.134376	-0.42765	0.18432	0.172164	-0.1709	-0.4219
-0.0652	-0.09871	-0.30182	0.039409	-0.42123	0.192593	0.064573	-0.26592	-0.44976
0.056369	-0.14619	-0.21031	0.121176	-0.12453	-0.07365	0.023765	-0.06029	-0.28368
0.068758	-0.1968	-0.14464	0.201827	0.009144	0.152661	-0.11367	0.132141	-0.32162
-0.19012	-0.03715	-0.01531	0.067847	-0.25943	-0.00011	0.116393	-0.16368	-0.29755
-0.15387	0.18181	-0.14882	0.073256	-0.30313	0.225903	0.235143	-0.25919	-0.31753
-0.24354	-0.03827	-0.09181	-0.08896	-0.23955	0.270526	-0.01845	-0.22905	-0.37973
-0.25339	0.021716	-0.06427	-0.22589	-0.14543	-0.03576	0.118939	-0.11636	-0.45861
-0.31069	0.023119	-0.22577	-0.12383	-0.23805	0.002796	0.019635	-0.26918	-0.47823
-0.01763	-0.19763	-0.08654	0.076844	-0.19675	-0.03396	0.208902	-0.10267	-0.25003
-0.03969	0.062597	-0.10727	0.193277	-0.17201	0.127677	0.034097	-0.00462	-0.43631
-0.24459	0.23962	-0.06801	0.166903	-0.09519	-0.05327	0.255547	-0.17754	-0.48951
-0.34015	-0.07823	-0.39337	-0.28776	-0.56679	0.275439	0.274932	-0.39686	-0.4851
-0.14172	0.233695	-0.04928	-0.04435	-0.12672	-0.0458	0.386889	0.027712	-0.19041
-0.27037	0.210774	-0.02053	-0.24489	-0.21034	0.058967	0.303567	-0.3061	-0.36574
0.04143	0.120565	-0.17154	-0.03799	-0.08824	-0.12628	0.310206	0.040287	-0.08555
0.036799	0.053919	0.136026	-0.00099	0.06028	0.017652	0.262425	0.155876	0.090875
0.086564	0.28991	-0.02456	0.018463	0.117682	0.031857	0.237402	-0.10517	-0.39407
-0.31872	0.067861	-0.08781	-0.17492	-0.15786	-0.05448	0.382396	-0.26668	-0.10888
-0.01788	0.065449	0.059443	0.079967	-0.05711	-0.12671	0.294795	0.017748	-0.13974
-0.14582	0.047471	-0.15759	-0.37201	-0.21956	0.269361	0.086488	-0.17716	-0.27692

-0.0238	0.230465	0.214625	0.227556	0.090157	0.012861	0.394817	-0.11227	-0.19086
-0.27338	0.01814	-0.18588	0.111206	-0.13002	-0.20478	0.418908	-0.07793	-0.19379
-0.31495	-0.22461	-0.29758	-0.06358	-0.25569	-0.09597	0.108293	-0.16766	-0.12855
-0.01652	-0.1841	-0.54289	-0.17228	-0.49522	0.258352	0.225994	-0.47261	-0.4112
-0.46064	0.343596	-0.46698	-0.31726	-0.03832	-0.55828	-0.58015	-0.11932	-0.10011
-0.30365	0.053401	0.082739	-0.01667	-0.23107	-0.27841	0.360109	-0.25249	-0.19891
-0.04458	0.193154	-0.09418	0.025084	0.058784	-0.39641	0.343834	-0.09545	-0.09101
0.158207	-0.15683	-0.27797	0.051597	0.105174	-0.21284	0.227797	-0.02098	0.196288
0.011328	-0.29849	-0.1132	-0.09936	0.084841	-0.11819	0.477794	-0.26324	0.435283
-0.04719	0.207659	0.057736	-0.01319	-0.21421	-0.01985	-0.0216	0.034105	-0.26487
0.115712	-0.08255	-0.17533	0.015695	0.056425	-0.1088	0.258351	-0.11369	0.327408
-0.14367	0.358382	-0.12151	-0.00411	-0.32746	0.333872	0.247603	-0.26474	-0.16698
0.010049	0.016451	-0.02774	-0.06	-0.16107	0.237883	0.052083	-0.06492	-0.055
-0.11587	0.021403	0.01078	-0.13047	-0.29437	0.04652	0.110943	-0.11125	-0.34669
-0.28096	0.293145	-0.36653	0.234914	-0.52739	0.331788	0.403796	-0.39454	-0.54347
-0.03516	0.191869	0.123803	0.025439	0.082119	0.021569	0.112344	-0.00737	-0.3753
-0.31488	0.127058	0.060307	-0.15173	-0.1412	0.225124	0.027765	-0.10054	-0.342
-0.38101	0.1729	-0.16165	-0.21215	-0.21747	-0.02483	0.283907	-0.31829	-0.44644
-0.07698	0.144564	-0.13365	-0.26163	0.012107	0.213612	-0.01625	-0.06054	-0.41748
-0.38615	0.268141	0.042911	-0.20059	-0.02752	-0.05562	0.24771	-0.28915	-0.42061
-0.04225	0.000209	-0.03322	-0.22205	0.023378	0.176706	0.179527	-0.24904	-0.4091
-0.10937	0.170906	0.056084	-0.17477	0.006899	0.004068	0.094272	-0.17748	-0.45297
-0.01864	0.363793	0.045881	0.021643	0.128612	-0.15159	-0.11797	-0.03594	-0.53339
0.202947	0.336987	0.481082	0.41979	0.364157	-0.03858	0.287662	0.0376	-0.27367
-0.02431	0.162813	0.177591	-0.1501	0.004024	0.053861	0.122879	-0.17088	-0.35015
-0.18393	0.153096	0.098449	-0.20224	-0.23129	0.181499	-0.17568	0.221521	-0.44522
-0.29784	0.120533	0.007464	-0.12386	-0.07428	0.049607	0.184723	-0.10997	-0.45742
-0.37483	0.291816	-0.11808	-0.19627	0.057865	0.138035	0.226963	-0.23911	-0.43991
-0.1422	0.034587	-0.11152	-0.08436	0.115341	0.074315	0.108447	-0.03054	-0.41297
-0.30349	0.05883	-0.14331	-0.32794	-0.23517	0.115358	0.171075	-0.2256	-0.35132
-0.13331	0.115585	-0.37597	-0.2379	-0.47362	0.392727	0.442763	-0.49215	-0.47931
-0.08383	-0.14234	-0.23136	0.044646	-0.52938	0.465254	0.272129	-0.50786	-0.47016
-0.05415	-0.13106	-0.4695	-0.24889	-0.61932	0.50599	0.369269	-0.55598	-0.56083
-0.06319	-0.23299	-0.58832	-0.26172	-0.59535	0.514201	0.227737	-0.57023	-0.47697
0.035529	-0.12555	-0.28405	0.102872	-0.3447	0.26565	0.272633	-0.28764	-0.44359

TCGA-3A-A	TCGA-3A-A	TCGA-3A-A	TCGA-3A-A	TCGA-3A-A	TCGA-3A-A	TCGA-3A-A	TCGA-3A-A	TCGA-3A-A
0.102425	-0.08782	0.027532	-0.34242	-0.24996	-0.12352	0.067982	-0.00776	0.148569
0.350791	0.010723	0.354446	-0.2567	0.067629	-0.39144	0.43626	-0.15487	0.010964
-0.5601	-0.3517	0.207921	-0.27199	-0.35712	-0.3521	-0.14528	-0.38564	-0.22028
0.050799	-0.15017	0.433068	-0.30913	0.492168	0.118635	0.003785	-0.29448	0.40375
-0.24423	0.016247	0.415383	0.060785	-0.34588	-0.33621	-0.35066	-0.32791	-0.41503
0.188055	0.171696	0.136025	0.057011	-0.2764	-0.1138	-0.04625	-0.1567	-0.24995
0.508569	-0.10994	-0.16912	0.427691	0.164144	0.043472	0.366985	0.23999	0.136521
0.177227	0.161703	-0.04873	-0.00676	-0.18609	-0.05967	-0.063	-0.15031	-0.03604
0.130103	0.063456	-0.04389	-0.30547	-0.35721	-0.25612	-0.2698	-0.21568	-0.19112
0.23534	0.181828	-0.15537	-0.1454	-0.19972	0.062857	-0.101	-0.19284	-0.21005
0.125951	0.272201	-0.1092	-0.23821	-0.26068	-0.13993	-0.02036	-0.04667	-0.15794
-0.02965	0.315254	-0.31046	-0.23036	-0.17837	0.007141	0.041482	-0.04151	0.064322
0.048677	-0.0852	0.22815	-0.06042	0.012756	-0.01045	0.259332	-0.15541	0.070165
0.116918	0.068846	0.220387	-0.02325	-0.42473	-0.24687	-0.24284	-0.31946	-0.19874
-0.32321	-0.2822	0.39211	-0.22296	-0.26819	-0.33931	-0.1473	-0.64904	-0.13265
0.059264	-0.36577	0.531781	0.259057	0.055115	0.0993	0.418233	0.023666	0.287229
0.078614	-0.08046	-0.4272	-0.09464	0.058668	-0.01453	0.457441	0.166437	-0.21057
0.0016	0.219933	-0.01752	-0.38792	-0.3558	0.125452	-0.09153	-0.31719	-0.19861
0.217963	0.185891	-0.35277	0.022088	0.054366	0.055796	0.362424	0.316642	0.045449
0.109773	-0.45208	-0.19277	0.153631	-0.30673	-0.56919	0.076224	-0.19371	0.008587
-0.14466	-0.44256	-0.03244	-0.02378	-0.17422	-0.35173	0.013567	0.021938	-0.11393
-0.24657	-0.44087	-0.0187	-0.52025	-0.45439	-0.53544	0.134336	-0.14143	0.070879
-0.05792	0.20099	-0.11552	-0.04739	-0.1177	0.109741	0.057016	-0.26785	-0.16847
0.102027	0.152527	0.298969	-0.20754	0.076361	-0.09365	0.260302	-0.21736	-0.00202
0.246911	0.192628	0.005218	0.383627	0.161922	0.228631	0.130723	0.1125	0.013519
0.351384	0.091759	0.284932	0.015082	0.200672	0.371883	0.007088	-0.20597	0.018465
-0.559	-0.41199	0.142458	-0.06154	-0.22846	-0.25306	-0.15708	-0.00688	0.059001
-0.2436	0.293492	-0.09629	0.214918	0.291656	0.295605	0.260453	0.213554	0.389526
0.090995	0.163225	-0.1404	0.264943	0.022674	-0.04562	0.318447	-0.11388	-0.00937
-0.24529	-0.10596	0.318679	-0.4457	-0.59523	-0.45189	-0.61613	-0.43647	-0.57252
-0.27912	0.07609	0.087265	-0.52511	-0.44995	-0.37864	-0.61698	-0.50793	-0.59862
-0.23718	-0.068	-0.32107	-0.46223	-0.42046	-0.45595	-0.4961	-0.31597	-0.51039
-0.32487	-0.26342	0.306285	-0.16704	-0.09424	0.072923	-0.31313	-0.20991	-0.23041
-0.33611	-0.33333	0.32275	-0.14463	-0.12573	0.001329	-0.32556	-0.16678	-0.237
-0.33096	-0.19356	0.313767	-0.0977	-0.04683	0.143159	-0.22498	-0.15387	-0.20963
-0.09729	0.153528	0.167371	-0.28863	-0.35408	-0.43305	-0.25364	-0.22692	-0.44221
0.264842	-0.16735	-0.47811	0.2846	-0.30583	-0.4438	0.248429	0.015302	-0.25593
0.052181	0.222487	0.238572	-0.22973	-0.45247	-0.41975	-0.45699	-0.12361	-0.55925
0.197184	-0.2135	0.542065	-0.11573	0.148987	-0.21698	-0.24955	-0.19425	-0.2295
0.009338	0.224022	0.069191	-0.0854	-0.36101	-0.40373	-0.18408	0.069882	-0.37099
0.044313	-0.00284	0.377625	-0.14247	-0.2019	-0.36304	-0.14303	-0.17523	-0.34346
0.244089	-0.01356	0.414801	-0.39443	-0.02414	-0.34296	-0.22229	-0.29625	-0.4976
-0.01197	0.116117	-0.36466	-0.27174	-0.30188	-0.23037	-0.02625	0.085156	-0.2584
0.430491	0.383593	-0.43692	-0.25341	-0.53737	-0.54463	-0.32082	-0.1749	-0.55514
0.11318	-0.00729	0.094824	0.100208	-9.98E-05	0.000557	0.125647	0.019045	0.133718
0.35787	-0.04377	0.232433	-0.28291	-0.26638	-0.25854	-0.26771	-0.20446	-0.33008
0.573157	0.233308	0.00821	0.534682	0.116018	0.018399	0.257312	0.287318	0.087031
0.131811	-0.18794	-0.0118	-0.02996	-0.25193	-0.14215	0.063772	-0.07883	-0.09519
0.185748	-0.17559	-0.01472	-0.00355	-0.25089	-0.27797	-0.14157	-0.13791	-0.1439

0.223244	-0.00851	0.178376	-0.00348	-0.41288	-0.30868	0.039604	-0.09255	-0.1156
0.180706	0.331895	0.190558	0.020841	0.115732	0.019866	0.107665	0.044014	-0.01771
-0.11596	-0.07095	0.38748	-0.40528	-0.27608	-0.35709	0.048103	-0.35927	0.004817
0.119824	0.303537	-0.46484	0.01794	-0.05957	-0.4061	0.258314	0.222851	-0.19144
0.00182	-0.22451	-0.36552	-0.20941	-0.09077	-0.22032	0.068382	-0.09829	0.090245
0.242598	0.072442	0.084019	-0.24229	-0.29339	-0.26777	-0.05792	-0.21355	-0.28751
0.255532	0.268502	-0.2878	0.047675	0.230402	-0.22062	-0.05065	0.031181	-0.23933
0.283289	0.268114	0.294621	0.174857	0.205465	-0.02691	0.137679	0.242346	-0.00204
0.312222	0.361156	0.495221	-0.02802	-0.01534	-0.22813	-0.12645	-0.13202	-0.13788
0.217625	0.040797	-0.06211	-0.1394	-0.13319	-0.23571	0.228743	-0.08654	-0.25335
-0.0265	0.068973	-0.11385	0.095372	-0.33217	-0.23813	-0.06393	-0.07175	-0.23817
0.552152	0.41138	-0.35418	0.13065	0.00863	0.12314	-0.14761	-0.34954	-0.31237
0.016195	0.24298	-0.23989	0.021187	-0.01308	-0.34561	0.057363	-0.02377	0.050896
0.18502	0.272446	-0.20637	-0.08388	-0.35197	-0.39854	-0.15972	-0.51288	-0.46622
-0.04976	0.043046	0.393771	-0.33882	-0.14226	-0.29583	-0.18493	-0.20602	-0.4498
0.196497	-0.23653	0.056246	-0.32959	-0.4667	-0.41159	-0.13418	-0.23131	-0.20595
-0.12768	-0.12485	0.653689	-0.35822	-0.27128	0.10468	-0.35032	-0.20646	-0.2712
0.099459	0.064846	0.365163	0.084015	0.218404	0.123666	0.131084	0.043323	0.135077
-0.32878	-0.02138	-0.00767	-0.18904	-0.12593	-0.06537	0.029326	-0.06696	-0.09523
0.263944	0.241518	-0.32809	0.20597	-0.1918	-0.19867	0.239914	0.029258	-0.25872
0.382536	-0.15915	0.197021	0.327536	-0.14127	-0.11395	0.402301	-0.03481	-0.13875
0.157963	0.256956	-0.46382	-0.12393	-0.28538	-0.41247	0.128392	-0.12655	-0.33061
0.069528	0.231273	-0.27713	0.035541	-0.16782	-0.05722	0.09107	0.012672	-0.14037
0.271105	-0.33178	0.147529	-0.35434	-0.3326	-0.63945	-0.29021	-0.30528	-0.51804
0.561083	0.266726	-0.29646	0.382006	-0.01194	0.128057	0.352053	0.224469	0.19894
0.02295	-0.17396	0.211417	-0.21157	0.048515	-0.00964	-0.021	-0.05253	0.003075
0.31054	0.036722	0.505788	-0.02145	-0.02149	-0.29854	-0.34527	-0.30229	-0.40787
0.346278	-0.21048	-0.6692	0.225839	-0.45983	-0.42708	0.063168	0.100882	-0.30864
-0.13605	-0.06139	-0.42499	-0.08387	-0.14791	-0.18698	-0.00221	0.177211	-0.30703
0.182795	0.229735	0.241601	-0.07911	-0.29897	-0.24823	0.188485	0.147074	0.220031
0.101926	0.245546	0.191465	-0.19408	-0.24438	-0.25737	-0.32272	-0.21252	-0.40556
0.061101	0.263603	0.222115	-0.20818	-0.23372	-0.23529	-0.2651	-0.21404	-0.41109
0.211064	0.157385	0.322123	-0.25405	-0.13475	-0.18621	-0.27965	-0.11327	-0.3395
0.244891	-0.20236	-0.17944	-0.12981	-0.47531	-0.31863	-0.05172	0.011706	-0.10526
0.102482	0.184917	0.254897	-0.00156	0.092119	0.185619	0.144938	0.160823	0.039204
0.664227	0.105647	0.163333	0.488582	0.197597	0.252383	-0.28174	-0.23402	-0.50975
0.181176	-0.32586	0.001504	-0.0041	-0.13007	-0.18611	-0.01026	-0.13471	0.01853
0.451776	-0.15624	-0.14636	0.483671	0.116329	0.054802	0.251979	0.187775	0.19178
0.262552	-0.43819	-0.16619	-0.09455	-0.47221	-0.58738	-0.44192	-0.38586	-0.16051
0.141425	-0.35414	-0.32447	0.033679	-0.06689	-0.29236	-0.17463	0.037773	-0.16289
0.446668	-0.53423	0.439659	0.059856	-0.48427	-0.4354	-0.15423	-0.15431	-0.37595
0.40224	-0.03212	0.019265	0.022297	0.018209	0.100306	0.509673	0.447574	0.038372
-0.00713	0.131723	0.014045	-0.04118	-0.22499	0.099403	-0.06948	-0.03702	-0.08306
-0.05045	0.063763	-0.08292	-0.21251	-0.34586	-0.5361	-0.41311	-0.38218	-0.37924
0.032749	-0.33797	-0.01779	0.097433	-0.22775	-0.35968	0.034051	0.071129	0.108431
-0.08922	-0.58444	-0.10166	0.12381	-0.44318	-0.63397	-0.28695	-0.25929	0.209689
0.110977	-0.37149	-0.18979	-0.2649	-0.47104	-0.53576	-0.22064	-0.3588	0.067084
-0.25313	-0.12207	0.087543	-0.18438	-0.17175	-0.07222	-0.22571	-0.13837	-0.17697
-0.34485	-0.30569	0.227142	-0.26204	-0.24344	-0.23869	-0.21455	-0.23976	-0.23874
-0.31671	-0.18583	0.209968	0.033578	-0.03803	0.203446	0.032109	-0.07243	0.026974

-0.25864	-0.0209	0.157608	-0.44713	-0.31809	-0.21099	-0.43147	-0.43185	-0.45172
-0.34907	0.050769	-0.02878	-0.33802	-0.28095	-0.21641	-0.31157	-0.26237	-0.33744
-0.34965	-0.08596	-0.14837	-0.02005	-0.17054	0.022275	-0.09667	-0.05075	-0.13701
-0.02086	0.058657	0.140115	0.166106	0.137579	0.277025	0.032767	0.055625	-0.02296
-0.11605	-0.42052	0.186149	-0.33601	-0.46995	-0.4927	-0.38564	-0.35918	-0.08474
-0.25527	-0.33069	0.198707	-0.0946	-0.35176	-0.34851	-0.13891	-0.10485	-0.03502
-0.00828	-0.1655	0.164785	-0.32092	-0.44645	-0.35899	-0.37691	-0.42919	-0.31024
-0.30168	-0.20768	0.041799	-0.08675	-0.15452	-0.27567	0.052564	0.028089	0.075877
0.080606	0.106527	0.241401	0.159337	0.147997	0.167237	0.051183	0.184802	0.008329
-0.30968	-0.1057	-0.00897	-0.29662	-0.18835	-0.21506	-0.28483	-0.21046	-0.22756
0.199373	0.28926	-0.21097	-0.01718	-0.19886	-0.19435	0.049396	0.170245	-0.10712
-0.23992	0.034555	0.057697	-0.18818	-0.05088	0.016261	-0.22733	0.005741	-0.0451
-0.32851	-0.15224	-0.04939	-0.43967	-0.4763	-0.40809	-0.40712	-0.47891	-0.45758
-0.29348	-0.11369	0.129102	-0.18269	0.004629	0.131135	-0.10697	-0.05916	-0.14824
-0.21981	-0.22159	0.135133	-0.04701	-0.28712	-0.10334	-0.19047	-0.12403	-0.08434
-0.44226	-0.39098	0.13548	-0.34376	-0.32245	-0.2207	-0.31599	-0.32213	-0.34963
-0.28355	-0.07991	0.188286	-0.31793	-0.08657	-0.17294	-0.3031	-0.34203	-0.34326
-0.27181	-0.18967	0.252289	-0.20028	0.026513	0.117097	-0.26487	-0.23242	-0.05121
-0.23649	-0.21287	0.278478	-0.40281	-0.33907	-0.10435	-0.30824	-0.32431	-0.30755
-0.27995	-0.12923	0.345188	-0.21978	-0.35489	-0.2933	-0.35918	-0.15323	-0.28658
-0.27874	-0.09089	0.068666	-0.30339	-0.31738	-0.32339	-0.29356	-0.27565	-0.33508
-0.3836	-0.28218	0.343822	-0.46988	-0.31494	-0.25651	-0.39692	-0.38994	-0.42533
-0.34613	-0.28079	0.432158	-0.54211	-0.29066	-0.28057	-0.42472	-0.44977	-0.49494
-0.28972	-0.23436	0.299754	-0.34821	-0.33837	-0.25522	-0.35304	-0.39085	-0.47392
-0.42579	-0.3278	0.073606	-0.42141	-0.32882	-0.38038	-0.38015	-0.32953	-0.33559
-0.19258	-0.22083	0.019247	-0.36114	-0.37435	-0.3354	-0.27031	-0.38039	-0.34947
-0.31959	-0.13768	0.209151	-0.24123	-0.23066	-0.06422	-0.21032	-0.23131	-0.1597
0.338699	0.236523	0.38245	-0.32575	-0.15829	0.017437	-0.39086	-0.44893	-0.40077
-0.40916	-0.17354	-0.18108	-0.46393	-0.41971	-0.30339	-0.26443	-0.43106	-0.41709
-0.31565	-0.03036	-0.18985	-0.39843	-0.38499	-0.27046	-0.37261	-0.331	-0.44236
-0.2936	-0.27676	-0.05286	-0.39656	-0.38488	-0.39759	-0.24192	-0.43164	-0.38691
-0.14765	0.010098	-0.28935	-0.31974	-0.26072	-0.22487	-0.24221	-0.26967	-0.31053
0.098501	0.011801	-0.34266	-0.27446	-0.30137	-0.19207	-0.30075	-0.29806	-0.28677
-0.36361	-0.14802	0.044802	-0.35463	-0.16968	-0.14598	-0.3043	-0.25	-0.26252
-0.36413	-0.04539	0.038765	-0.47858	-0.24369	-0.2033	-0.42725	-0.48411	-0.48324
-0.41147	-0.14536	0.020934	-0.43592	-0.45126	-0.3401	-0.34386	-0.44427	-0.41425
-0.43606	-0.31091	-0.08713	-0.38997	-0.43404	-0.41101	-0.33029	-0.24115	-0.36067
-0.35581	-0.1918	-0.11624	-0.46534	-0.48272	-0.45824	-0.39855	-0.39796	-0.41451
-0.34517	-0.09939	-0.1702	-0.24782	-0.30235	-0.21283	-0.11348	-0.13802	-0.21622
-0.25535	-0.01309	-0.02408	-0.41707	-0.39417	-0.36042	-0.41097	-0.3826	-0.45144
-0.2857	-0.29304	0.24632	-0.51349	-0.40967	-0.37577	-0.37146	-0.51332	-0.46759
-0.50447	-0.11128	0.05472	-0.59432	-0.43484	-0.33997	-0.52803	-0.40963	-0.52042
-0.42291	-0.29942	0.112836	-0.20979	-0.2175	-0.15844	-0.02606	-0.09844	-0.09757
-0.44639	-0.19719	0.155366	-0.40265	-0.38098	-0.36635	-0.31501	-0.2069	-0.34655
-0.24866	-0.07957	-0.02503	-0.08031	0.043749	0.119538	0.108992	0.01547	0.166442
-0.06891	0.212185	0.032772	0.128167	0.145134	0.22473	0.129558	0.23639	-0.04535
-0.35222	-0.21005	0.278863	-0.37626	-0.28622	-0.26054	-0.37531	-0.33954	-0.3789
-0.22485	-0.1351	0.065396	-0.1929	-0.19255	-0.06453	-0.17457	-0.0627	-0.08972
-0.36388	-0.09756	-0.08159	-0.14934	-0.25517	-0.12346	-0.00713	-0.07595	-0.10405
-0.28455	-0.33997	0.29158	-0.14009	-0.16378	-0.16279	-0.06036	-0.20444	0.023475

-0.35515	-0.14955	0.242735	-0.12151	-0.16086	0.047247	-0.13684	-0.12441	-0.11688
-0.24341	0.032095	-0.06315	-0.13894	-0.23802	0.13088	-0.06254	-0.2274	-0.02406
-0.173	-0.18957	0.09364	0.086653	0.051382	0.101827	0.039737	0.102004	0.233948
-0.44899	-0.25783	-0.1507	-0.46581	-0.42431	-0.14745	-0.46612	-0.46497	-0.40913
0.594392	0.545653	-0.36323	0.347553	0.301276	0.385847	0.0469	0.227386	0.004189
-0.24435	-0.04581	0.08676	-0.22405	-0.20782	-0.06697	-0.14707	-0.35065	-0.07278
-0.3412	-0.2105	0.152319	0.097395	-0.00315	0.021433	0.125616	0.01272	0.038513
0.341476	0.073889	0.015471	0.294479	-0.16811	-0.15771	-0.05722	-0.0661	-0.17582
0.527588	0.181331	-0.11276	0.473676	0.009374	0.023904	0.133446	0.152312	-0.07527
-0.03559	-0.15044	0.085483	-0.14732	-0.48207	-0.40216	-0.32025	-0.14835	-0.34179
0.370981	0.123821	0.023299	0.333709	-0.05487	-0.03911	0.044998	0.055008	-0.00899
0.107481	-0.17877	0.057178	-0.18506	-0.1053	0.130015	-0.30088	-0.16288	-0.26783
0.317904	0.049988	0.06435	-0.05841	-0.09217	-0.04156	0.104179	-0.20496	-0.01628
-0.16247	-0.1039	-0.10039	-0.08383	-0.24035	-0.3712	-0.05233	-0.317	-0.16663
-0.43079	-0.01643	0.142388	-0.62155	-0.51433	-0.47745	-0.57607	-0.54985	-0.60029
-0.34767	-0.28433	0.182646	-0.40838	-0.3223	-0.27489	-0.38766	-0.41417	-0.36333
-0.4223	-0.31107	0.09863	-0.28707	-0.36363	-0.24548	-0.42587	-0.32668	-0.30517
-0.36542	-0.33578	0.24958	-0.37211	-0.27226	-0.28454	-0.34613	-0.24429	-0.34891
-0.40916	-0.26596	0.169817	-0.43854	-0.44089	-0.34237	-0.45951	-0.45633	-0.43692
-0.49667	-0.24231	0.311666	-0.30665	-0.36043	-0.27159	-0.46851	-0.2758	-0.44491
-0.35568	-0.27652	0.294666	-0.40335	-0.27538	-0.3037	-0.36961	-0.3085	-0.37812
-0.41663	-0.19556	0.092963	-0.39948	-0.37864	-0.34805	-0.41817	-0.38654	-0.4155
-0.37857	-0.38756	-0.13468	-0.39703	-0.42675	-0.33181	-0.43314	-0.47276	-0.40503
-0.30315	-0.08529	0.311772	-0.29825	-0.08842	0.02515	-0.4261	-0.37932	-0.32672
-0.30654	-0.13907	0.340797	-0.35159	-0.14128	-0.22592	-0.39202	-0.23142	-0.33665
-0.25524	-0.2004	0.230965	-0.49479	-0.33464	-0.23043	-0.40523	-0.36262	-0.3669
-0.40078	-0.39328	0.209251	-0.47162	-0.37786	-0.34398	-0.42621	-0.40055	-0.41009
-0.49309	-0.18052	0.063094	-0.47253	-0.29723	-0.30323	-0.45279	-0.47863	-0.46248
-0.33333	-0.29156	0.139359	-0.44424	-0.3697	-0.29119	-0.45266	-0.49813	-0.44035
-0.35067	-0.18617	0.155125	-0.38243	-0.27139	-0.25502	-0.34909	-0.40621	-0.34538
-0.5532	-0.03214	-0.08516	-0.50635	-0.09979	0.008617	-0.43209	-0.47702	-0.44111
-0.59025	-0.11314	-0.12781	-0.56529	-0.3194	-0.14605	-0.4395	-0.47651	-0.49415
-0.65846	-0.32094	-0.1143	-0.64294	-0.41469	-0.35017	-0.52267	-0.52969	-0.55448
-0.61032	-0.25171	-0.14122	-0.6528	-0.49556	-0.23449	-0.56845	-0.59558	-0.62265
-0.47941	-0.28126	-0.00164	-0.5447	-0.4581	-0.30344	-0.55865	-0.52337	-0.51975

TCGA-3A-A	TCGA-3A-A	TCGA-3A-A	TCGA-3E-A	TCGA-3E-A	TCGA-F2-7	TCGA-F2-7	TCGA-F2-A	TCGA-FB-A
0.177036	0.00464	0.081732	-0.42529	-0.30172	-0.06422	-0.03032	0.130082	-0.07966
0.005801	0.374241	0.540625	-0.13973	-0.61214	0.235017	0.247337	0.554963	0.101743
-0.13266	-0.05329	0.011573	-0.48507	0.169546	0.251121	0.034877	-0.27943	-0.34705
0.068405	0.094484	0.010635	0.00829	-0.18155	0.256104	0.181028	0.496058	0.361487
-0.46945	0.141897	-0.18716	-0.17928	0.077617	-0.01453	-0.02325	0.340815	0.369341
-0.10229	0.01294	0.143676	0.019234	0.188322	0.025984	0.204801	0.171948	0.153281
0.44778	-0.51446	0.205917	-0.46164	-0.16598	-0.44584	-0.37356	-0.13131	-0.4295
-0.1873	-0.07927	0.195212	-0.00044	0.280576	-0.21466	-0.10403	0.114456	0.106349
-0.1016	0.194763	0.138171	-0.02939	0.167183	-0.13129	0.009297	0.355855	-0.11475
-0.08407	0.120612	0.114374	0.228193	-0.13371	-0.02528	-0.21534	0.270829	0.317943
-0.16286	-0.02552	0.352034	-0.05586	-0.02638	-0.27893	-0.33006	0.202194	-0.10087
0.031537	-0.0643	0.46995	-0.13906	-0.17412	-0.25583	-0.17321	0.505224	0.018964
0.001348	-0.0389	0.079327	-0.06257	0.055902	0.198416	0.160959	-0.04632	-0.11839
-0.30341	-0.25361	-0.17614	-0.20673	-0.07076	-0.05751	-0.31049	0.070336	0.084295
-0.50557	0.134607	-0.15779	0.144079	-0.00184	0.113186	0.033182	0.104485	0.412574
0.252142	0.205285	-0.03331	-0.36989	-0.40768	0.306527	0.262542	0.358269	0.42272
0.061554	-0.36292	0.157512	-0.14777	-0.10071	-0.13016	-0.08981	0.104722	-0.03403
-0.0424	-0.25589	0.164565	0.142664	-0.37798	0.097016	-0.06062	0.148331	0.085749
0.051187	-0.33649	-0.00886	0.471353	-0.54932	0.124368	-0.09846	0.298366	-0.21451
-0.21482	-0.44506	0.256279	-0.04925	-0.01614	-0.52274	-0.40167	0.186718	-0.49858
-0.06487	-0.02947	-0.11065	-0.28037	0.056288	-0.07381	0.106514	-0.15151	-0.34242
-0.13052	0.39071	0.312835	0.200354	0.19445	-0.25255	0.112865	-0.25888	-0.50393
0.048034	-0.24715	-0.20299	0.151561	-0.1274	-0.19886	-0.27743	-0.21463	-0.05871
-0.05292	0.144855	-0.02808	0.172758	-0.42137	0.331323	0.090493	0.450662	0.279891
0.029186	-0.19924	-0.00926	0.245	0.438088	-0.31261	-0.22773	0.015371	0.094151
0.228712	0.246482	-0.2586	0.169041	-0.018	0.068431	0.095311	0.090068	0.180181
0.319385	-0.19185	0.256529	-0.0349	-0.20464	0.283673	0.049484	-0.23239	-0.38334
0.219887	0.139793	-0.06992	0.219862	0.152558	0.111516	0.076824	-0.22038	0.129571
-0.04186	0.470538	-0.50272	0.40668	0.210275	0.166953	0.287538	0.424078	0.375159
-0.5224	0.254229	-0.08369	-0.01818	-0.15533	0.114467	0.339636	-0.20426	0.26489
-0.41628	0.396033	-0.32013	0.531684	-0.50848	0.540234	0.549214	0.476882	0.56016
-0.4988	0.43782	-0.29806	0.406166	-0.39393	0.023394	0.331487	-0.12529	0.726541
-0.14589	0.066376	0.062276	-0.04402	0.431114	0.165521	0.268006	-0.01872	0.126132
-0.18241	-0.01557	0.139617	-0.24204	0.427767	0.272404	0.322428	-0.03397	0.095811
-0.06229	0.183802	-0.02102	-0.05625	0.369224	0.305891	0.358974	0.081036	0.249176
-0.22785	-0.31659	0.306402	-0.01586	-0.0776	-0.2155	-0.13722	0.022886	0.18528
0.279159	-0.20201	0.126553	0.391231	0.383778	-0.04483	0.214524	0.573084	-0.04688
-0.35194	-0.38045	0.387647	-0.10565	-0.12958	-0.1366	-0.29667	0.240309	-0.22511
0.064172	-0.08923	0.38215	-0.31331	-0.20526	0.201643	0.067472	-0.26598	-0.20737
-0.14919	-0.52626	0.311754	-0.14977	-0.36088	-0.23502	-0.34082	-0.06349	0.107289
-0.19038	-0.5429	0.337854	-0.18497	-0.44706	0.168574	0.038634	0.23065	0.321902
0.034813	0.036891	0.166641	-0.07054	0.208671	-0.01118	0.174394	-0.24741	-0.16626
-0.08362	0.20359	0.045061	0.324615	-0.01844	0.108014	0.251812	0.194046	0.203935
-0.31418	-0.23873	0.307486	-0.00877	-0.07852	-0.23909	0.069385	-0.10946	0.22749
0.090175	0.46996	-0.40643	0.23738	-0.5708	0.375491	0.409676	0.196324	-0.07429
-0.18624	-0.13867	0.101998	-0.08241	-0.29902	-0.02981	-0.01358	-0.16925	-0.14045
0.137922	-0.40994	-0.09385	0.521618	0.395827	-0.55203	-0.47215	0.283918	-0.18847
-0.05494	-0.19753	-0.0312	-0.08822	0.211237	-0.03948	-0.02607	0.021131	0.070415
-0.26226	-0.40997	0.083451	-0.23239	0.145146	-0.3414	-0.32634	-0.02747	0.001918

0.150124	-0.19939	0.070796	0.141401	-0.05698	-0.07681	-0.15437	0.351001	0.087028
0.099006	0.461795	-0.16032	0.219407	-0.10135	0.457146	0.271527	0.47648	0.143068
-0.11243	-0.03946	0.061265	0.167489	0.10114	0.280529	0.161438	0.151071	-0.20699
0.177664	-0.03107	0.204845	0.17828	0.034736	0.014793	0.138323	0.360103	0.165251
0.063915	0.1162	-0.14181	-0.30867	0.256455	0.218648	0.330314	0.304086	-0.24463
-0.07215	-0.12885	-0.04356	0.144082	0.208015	-0.0664	0.019507	0.209371	0.100272
-0.10507	-0.04942	0.249538	0.089346	0.175549	-0.3056	-0.10745	-0.24814	0.065367
-0.14276	-0.11619	0.356252	0.160027	0.176112	-0.28002	-0.21787	-0.02421	0.196083
-0.22719	0.118477	-0.0079	0.322007	0.344548	-0.15094	-0.01309	0.277223	0.298795
-0.05769	0.159074	0.071048	0.24935	0.003673	0.240359	0.310863	0.358703	0.375085
-0.05326	0.240203	-0.19137	0.499634	0.089297	0.385152	0.317047	0.325773	0.128977
-0.1415	0.511966	0.227835	0.56601	-0.44915	0.418656	0.475131	0.349751	0.469884
-0.19982	0.130476	0.242564	0.174815	-0.08633	-0.04962	-0.01587	0.345689	-0.193
-0.33503	-0.01503	0.193018	0.390167	-0.26171	-0.03269	-0.08471	0.170522	0.14091
-0.00668	-0.09733	0.321193	-0.29587	-0.20584	0.159226	0.044269	-0.00674	-0.0669
-0.10303	-0.31327	0.173326	-0.09935	-0.4294	-0.01903	0.023158	0.220294	0.000808
-0.18	-0.22222	0.225049	-0.28691	-0.06761	0.182442	0.135895	0.553588	0.506093
0.117518	0.026849	-0.06299	-0.20364	-0.30284	0.089776	0.048491	0.330873	0.14703
0.06713	0.271694	-0.11642	-0.09602	0.200325	0.241422	0.231194	0.102642	0.174335
0.08452	-0.38262	0.129981	0.180117	-0.02625	-0.14737	-0.09154	0.242606	0.313749
0.368582	-0.24987	0.35282	0.074577	0.11453	-0.28647	-0.05128	0.547475	0.224861
-0.01035	0.04921	0.277034	0.175775	0.191488	0.223742	0.337207	0.339228	0.075144
0.165852	-0.17698	0.238139	0.025591	0.108462	-0.13385	0.061859	0.146235	-0.00039
-0.23795	-0.42566	0.261852	-0.17541	0.35395	-0.44523	-0.32144	0.237241	0.111922
0.156136	-0.56243	0.29535	-0.01151	-0.4203	-0.59127	-0.24553	-0.22057	0.206101
0.125564	-0.11807	0.161065	-0.15148	-0.30088	-0.13597	-0.08833	-0.18843	-0.06548
0.044668	-0.28038	0.223724	-0.03765	-0.00867	-0.27109	-0.12881	-0.1087	-0.0985
-0.30591	-0.52125	0.510766	0.272485	0.537779	-0.60396	-0.19729	-0.39772	0.157098
-0.09667	0.048867	-0.27824	0.374093	0.399941	-0.13307	-0.21519	-0.05782	0.146372
-0.21271	-0.02621	-0.3686	0.278397	-0.57501	-0.065	-0.1342	0.506262	0.090175
-0.23811	0.015113	0.208161	0.17921	0.083648	-0.05944	-0.22953	-0.20288	-0.15033
-0.23463	0.111178	0.0572	0.283207	0.11049	-0.00777	-0.18951	-0.17408	-0.06027
-0.09656	-0.3456	0.023354	-0.23975	0.176426	-0.33871	-0.36496	-0.24663	-0.26222
-0.15972	-0.36865	0.159893	-0.57818	-0.06805	-0.22097	-0.01317	-0.14128	0.190859
0.121374	0.008523	0.028572	0.249197	-0.28522	0.162574	-0.03549	0.12076	0.046255
-0.55669	0.330781	0.030307	0.091366	0.647892	-0.45738	-0.45763	-0.21002	-0.12593
-0.09786	-0.26811	-0.04274	-0.14348	0.160678	-0.19478	-0.18195	-0.35025	-0.14934
-0.10709	-0.15351	0.028577	-0.23546	0.441926	-0.24614	-0.06358	0.103089	-0.06225
-0.41807	-0.54297	0.511312	-0.10605	0.071813	-0.61534	-0.62485	-0.34862	0.104679
-0.3845	0.105253	0.152041	0.11096	0.191114	-0.38557	0.013594	-0.35385	0.015712
-0.46009	-0.4817	0.213712	0.421602	-0.26459	-0.54394	-0.16151	-0.46123	0.225355
0.501089	-0.09153	-0.34162	-0.14488	-0.20062	0.044664	0.292913	-0.07519	-0.29808
-0.05212	-0.01302	0.209023	0.054804	-0.23818	0.064762	0.168247	-0.12375	0.235184
-0.33932	-0.50952	0.36851	0.004564	0.227465	-0.66539	-0.64631	-0.2779	0.265317
0.129393	-0.35818	0.136192	-0.13426	0.105616	-0.46933	-0.45528	-0.2717	-0.21892
-0.18684	-0.47679	0.357102	0.153541	0.122626	-0.54952	-0.49144	-0.1902	-0.14932
-0.24077	-0.56783	0.490901	0.082001	0.169785	-0.54592	-0.54148	-0.40815	-0.22119
-0.11749	0.274148	-0.05515	-0.08282	-0.04627	0.307772	0.329357	0.099348	0.144684
-0.22596	0.228629	-0.20704	-0.29294	-0.04753	0.429263	0.310308	-0.03253	0.015004
0.118403	0.24849	-0.14291	0.064531	0.244482	0.308112	0.258231	-0.01436	0.299236

-0.43195	0.441892	-0.11233	0.338229	-0.29269	0.305627	0.362989	0.244677	0.388846
-0.30005	0.390512	-0.20585	0.231794	-0.27783	0.387692	0.452232	0.201213	0.406219
-0.0181	0.169946	0.029018	-0.04153	0.343238	0.144976	0.212416	-0.12069	0.266082
0.086097	0.285934	-0.22029	0.220114	0.027306	0.309156	0.281503	0.165138	0.312462
-0.1037	-0.25488	0.177474	-0.27552	0.149379	-0.25324	-0.17531	-0.32228	0.06383
-0.0677	-0.07646	0.060816	-0.21512	0.206665	0.049952	0.093505	-0.12311	0.259737
-0.28728	-0.20889	0.018215	-0.22856	0.02439	-0.13294	-0.12339	-0.17534	0.200459
-0.03955	-0.03002	-0.09252	-0.3513	-0.02234	-0.02462	0.117762	-0.20012	-0.18942
0.422966	-0.0853	0.108439	0.208957	0.269005	0.123561	0.188166	0.087089	-0.00908
-0.27504	0.141744	-0.03639	-0.09848	0.037841	0.156773	0.174375	0.040084	0.030456
-0.23731	-0.13731	0.129341	0.158855	-0.27912	-0.40843	-0.32582	-0.15277	-0.06889
-0.02351	-0.04338	0.095423	-0.29274	0.048561	0.281159	0.287142	0.092231	0.097971
-0.44712	0.219144	-0.26407	0.00628	-0.35198	0.194773	0.305974	-0.07591	0.177513
0.034581	0.287153	-0.05354	-0.01661	0.308786	0.319143	0.32802	0.216305	0.301764
-0.16795	0.140449	0.077658	-0.28357	0.206632	0.246232	0.182107	-0.03052	0.043708
-0.23019	0.250588	0.058106	-0.25953	-0.28801	0.617508	0.511225	0.187029	-0.08711
-0.26334	0.15624	0.090712	0.10382	-0.01805	0.174506	-0.11139	0.314139	-0.12137
-0.14584	0.243438	0.208175	-0.06952	0.243776	0.295533	0.026625	0.016825	0.032282
-0.17278	0.373634	-0.00542	-0.09876	0.054484	0.420075	0.411241	0.136728	0.18906
-0.22184	0.193958	-0.14831	-0.07779	-0.11869	0.301109	0.285368	-0.15015	0.012139
-0.3103	0.117996	0.162794	-0.17868	-0.11785	0.191527	0.297114	0.137813	0.184139
-0.32368	0.306307	0.003983	-0.2574	0.202035	0.429424	0.436885	0.228846	0.145334
-0.31662	0.29967	0.137733	-0.16816	0.313984	0.379256	0.381035	0.381876	0.258332
-0.43679	0.443077	-0.31694	0.308429	-0.3496	0.483972	0.478058	0.280213	0.467213
-0.34111	0.182723	0.18601	-0.4272	0.156084	0.343272	0.424424	-0.1106	-0.19569
-0.39334	-0.00795	0.164796	-0.22979	0.162324	0.180212	0.243716	0.039766	-0.14559
0.015481	0.277218	-0.17172	-0.12508	0.02573	0.373076	0.387753	0.00592	0.240953
-0.03236	0.32141	-0.30346	0.407484	-0.41448	0.536503	0.514884	0.307648	0.403685
-0.30113	0.20396	-0.05521	0.317635	-0.57005	0.326897	0.289981	-0.08074	0.478203
-0.43648	0.331856	0.058124	0.187812	-0.48306	0.361714	0.406568	0.300133	0.244445
-0.44933	0.416397	-0.01154	0.180581	-0.52277	0.235942	0.438953	0.214348	-0.08807
-0.24909	0.121222	0.220347	0.258806	-0.31304	-0.05632	0.072942	-0.25799	0.086118
-0.38777	0.175382	0.187649	0.381973	-0.011	-0.18023	0.101532	-0.04167	0.285857
-0.29454	0.455982	-0.10916	0.154978	-0.16239	0.342831	0.372514	0.077003	0.376869
-0.46147	0.424364	-0.24366	0.301458	-0.3152	0.359045	0.475302	0.293305	0.533025
-0.30948	0.25	0.133128	-0.08781	-0.37431	0.307415	0.348358	-0.09568	0.397773
-0.40997	0.334983	-0.06015	-0.1887	-0.31908	0.410499	0.481341	-0.12206	0.282567
-0.40333	0.438156	-0.10797	-0.13315	-0.32112	0.423005	0.518849	0.027397	0.469694
-0.20268	0.25087	0.167151	-0.2623	-0.22795	0.330242	0.303968	0.235749	0.187032
-0.31007	0.215649	0.001436	0.060046	-0.16118	0.193409	0.328272	0.174155	0.23448
-0.47766	0.286982	-0.2044	0.065075	-0.25816	0.346488	0.460329	0.133196	0.338949
-0.55701	0.530049	-0.48866	0.443726	-0.41147	0.504953	0.455866	0.13007	0.618647
0.032599	0.143604	-0.04894	-0.28526	0.074875	0.333128	0.240852	-0.17994	0.208716
-0.2371	0.256153	-0.10874	-0.23228	-0.12217	0.351559	0.392451	0.037966	0.229165
0.139282	0.199985	0.020836	-0.20986	-0.00392	0.307842	0.269772	0.088317	0.040504
0.201581	0.18764	-0.02165	0.247548	0.060483	0.248786	0.1318	-0.00101	0.159481
-0.29117	0.211996	0.046342	-0.11509	0.122473	0.321505	0.393467	0.135183	0.198267
-0.03085	0.012404	-0.05105	-0.29312	0.120396	0.261878	0.238341	0.002682	0.18417
-0.0403	0.119876	0.094351	-0.17449	0.039465	0.228503	0.218556	-0.00197	0.087599
0.033108	-0.08207	0.119541	-0.25023	-0.09511	0.200783	0.169919	0.093529	0.104514

-0.11009	0.215004	0.084969	-0.13683	0.269902	0.284837	0.186606	-0.0124	0.243874
-0.02461	-0.04166	-0.11817	-0.04565	0.049019	0.134695	0.168704	0.017565	0.042608
0.090846	0.068181	-0.10698	-0.35091	-0.11765	0.356826	0.357426	0.007043	0.166109
-0.4076	0.385982	-0.25884	0.591066	-0.68539	0.41884	0.350127	-0.1538	0.581093
-0.16067	0.308374	-0.40161	0.545965	-0.39886	0.428787	0.484859	0.372639	0.380526
-0.1549	0.37172	-0.19938	-0.07588	0.091636	0.429536	0.343601	-0.1358	0.155606
0.174237	0.362586	-0.31337	-0.11649	-0.089	0.378295	0.463117	-0.0511	-0.22486
-0.1493	-0.31128	-0.0621	0.25008	0.376624	-0.31961	-0.27564	0.165206	-0.13009
0.081937	-0.41172	-0.21412	0.558361	0.470629	-0.53268	-0.44783	0.207514	-0.06767
-0.23651	-0.00279	-0.19737	-0.19916	-0.26785	0.071373	0.114986	0.083074	0.122492
-0.03439	-0.27448	-0.10326	0.262135	0.389647	-0.38884	-0.34611	0.115403	-0.23879
-0.11929	0.385706	-0.39331	0.271798	-0.37497	0.567688	0.520324	0.429556	0.392663
0.148259	0.104821	-0.06406	0.156027	-0.27901	0.19754	0.24543	0.176541	0.010812
-0.34692	0.252208	-0.09927	0.187335	-0.33383	0.27791	0.343306	0.171785	-0.13806
-0.54633	0.440312	-0.36127	0.399604	-0.65556	0.517787	0.488108	0.438263	0.499211
-0.32873	0.254502	0.044505	-0.10207	0.075193	0.226219	0.226719	0.03995	0.136235
-0.1558	0.151747	0.006172	-0.35069	-0.04106	0.272284	0.348899	0.066102	0.175001
-0.1785	0.367407	-0.14162	-0.29174	-0.22416	0.456308	0.45427	-0.00923	0.124128
-0.26837	0.24266	-0.0367	-0.24685	-0.26703	0.313455	0.30183	-0.02489	0.011418
-0.29801	0.290645	0.112631	-0.42839	-0.08581	0.450819	0.424396	0.134936	0.194897
-0.22122	0.312806	0.166063	-0.35497	-0.08905	0.374946	0.380004	0.04041	0.231298
-0.37873	0.323648	0.088449	-0.33381	-0.05115	0.43746	0.412715	0.112654	0.111807
-0.31208	0.191256	0.028358	-0.50804	0.106325	0.282491	0.359631	-0.10174	0.124137
-0.32796	0.176905	0.239183	-0.06419	0.34237	0.129961	-0.20106	0.249237	0.152214
-0.1856	0.321948	0.016917	-0.26933	0.045192	0.371177	0.36038	0.290276	0.179023
-0.24756	0.365044	0.109712	-0.2702	-0.2992	0.276068	0.349889	0.253578	0.131796
-0.28534	0.364369	-0.08053	-0.23972	0.102703	0.383904	0.419549	0.093307	0.119263
-0.33896	0.296449	-0.0032	-0.10761	-0.06668	0.418903	0.406278	0.12314	0.319344
-0.40611	0.192359	-0.09488	-0.23363	0.01455	0.207573	0.276012	0.090348	0.070712
-0.23311	0.321782	0.108205	-0.29378	-0.2662	0.374397	0.315925	-0.0134	0.292599
-0.4528	0.57575	-0.51719	0.524402	-0.6403	0.46146	0.534125	0.484425	0.562212
-0.44259	0.394255	-0.02753	0.515707	-0.63131	0.428246	0.362387	-0.11396	0.674189
-0.57611	0.395872	-0.35557	0.588554	-0.72268	0.432814	0.392337	-0.0994	0.668696
-0.51971	0.269471	-0.23874	0.490234	-0.80065	0.466536	0.359003	-0.1167	0.660132
-0.4508	0.20699	-0.03373	0.211563	-0.26362	0.36011	0.376431	0.098615	0.558613

TCGA-FB-A	TCGA-FB-A	TCGA-FB-A	TCGA-FB-A	TCGA-FB-A	TCGA-H6-8	TCGA-H6-A	TCGA-H8-A	TCGA-HV-A
-0.11484	-0.23487	0.315133	-0.30246	-0.04696	0.261352	-0.2807	-0.12963	-0.06674
0.296271	-0.39825	0.251583	0.188098	0.05042	0.411428	0.247296	0.441527	-0.58054
0.062279	0.254439	0.17022	-0.12057	0.093214	0.054734	0.190842	-0.07742	0.031532
0.138341	-0.2957	-0.15094	0.423323	-0.06987	0.22998	0.191356	-0.07939	-0.04463
-0.16559	0.169707	-0.39051	0.155321	-0.3116	0.343227	0.461163	-0.2521	-0.20142
0.170637	-0.00776	0.12499	0.02117	-0.08615	-0.26767	0.028197	0.01406	-0.22507
0.067749	-0.27555	0.619557	-0.44564	0.009386	0.188078	-0.3316	-0.34877	-0.20011
0.27449	0.195556	-0.13631	-0.09329	0.104319	-0.09508	0.206875	0.282461	0.152362
0.174876	0.069276	-0.07621	-0.23194	0.009646	0.082199	0.423153	0.315387	-0.10382
0.295442	-0.31352	-0.27591	-0.18636	0.059378	-0.27545	0.367372	0.360448	-0.2956
0.325447	-0.08901	0.118599	-0.30793	0.021257	0.084902	0.066005	0.453392	-0.13985
0.031716	-0.16754	-0.20036	-0.11222	-0.31018	0.047406	0.135364	0.479193	-0.08971
0.318384	-0.11865	0.175001	-0.36899	-0.03909	-0.10451	0.20735	0.003654	-0.43509
-0.06022	0.283038	-0.15163	-0.10094	-0.02162	0.231815	-0.00695	-0.13086	0.027423
-0.15036	-0.10901	-0.50782	0.020187	-0.6547	0.335926	0.107414	-0.07916	-0.02203
-0.05009	-0.3303	0.039369	0.432845	0.305094	0.18888	0.175791	-0.18682	-0.07716
-0.26282	-0.09889	-0.17745	0.096565	-0.48093	0.045985	0.283061	-0.06373	0.215618
0.097095	-0.09948	-0.07862	-0.09302	-0.11361	-0.28848	0.0461	-0.20238	-0.0861
-0.13889	-0.07032	0.132071	-0.21885	0.091249	0.136101	0.024267	0.149432	-0.27251
-0.27943	-0.37661	0.575243	-0.58378	-0.08757	-0.11356	-0.40858	0.156506	-0.47439
-0.47673	-0.14332	-0.02082	-0.11641	0.145966	0.245973	-0.47925	-0.0469	-0.11782
-0.19946	0.012038	0.327938	-0.60792	-0.25939	0.383298	0.057866	0.57123	0.413605
-0.26269	-0.23336	-0.09782	0.199874	0.279345	-0.32833	-0.06393	-0.2899	-0.08805
0.117165	-0.42316	-0.33367	0.269629	0.025808	-0.15049	0.440327	-0.1217	-0.3154
-0.30142	-0.23565	-0.05001	0.134605	0.016163	-0.31109	-0.09573	0.203111	-0.01362
0.477298	-0.25003	-0.2103	0.102155	0.02666	-0.24693	0.134822	0.089464	-0.49588
0.18097	0.446329	-0.49113	0.202556	-0.22406	0.415788	0.387975	-0.13905	0.039493
0.391812	0.263093	0.001804	0.000494	0.327764	0.050328	0.376193	-0.03436	0.20607
0.156491	-0.30514	-0.18058	-0.16484	0.206047	-0.50015	0.398994	0.263926	-0.44615
-0.00043	0.141979	-0.29921	-0.03125	-0.17346	-0.02927	0.425417	-0.16702	0.314766
0.240592	-0.62598	-0.65229	0.407385	0.177045	-0.49281	0.370201	0.133748	-0.3353
0.153381	-0.50256	-0.49243	0.466712	-0.44824	-0.51999	0.383446	0.21696	-0.18424
-0.03903	0.142297	-0.37863	0.108861	-0.25839	0.24538	0.214942	-0.27231	-0.19781
-0.17222	0.083085	-0.40206	0.103328	-0.1868	0.339243	0.196298	-0.3141	-0.16311
0.099931	0.0221	-0.43029	0.218266	-0.26787	0.184383	0.287627	-0.27479	-0.26626
-0.06543	0.019443	-0.01979	-0.28468	0.296541	0.117893	0.034886	-0.05604	-0.24202
-0.42492	-0.55598	0.475596	-0.50182	-0.2998	-0.18929	0.142319	0.015107	-0.25386
-0.28048	-0.08614	-0.16413	-0.50176	0.364434	0.050205	-0.22367	0.018186	0.047443
-0.03442	-0.19704	0.14523	-0.35998	-0.29169	0.128741	-0.04768	0.263891	-0.21484
-0.25594	-0.10209	-0.07347	-0.28404	0.127546	0.277913	-0.00358	0.016448	-0.19262
0.062669	-0.31691	-0.33381	-0.04455	0.030454	0.190349	0.283879	-0.39113	-0.27377
0.282308	-0.11399	0.069145	-0.34384	-0.19668	0.149657	0.046014	0.227835	-0.40302
0.458512	-0.50691	0.103774	-0.34504	-0.22793	-0.31926	0.112021	-0.00635	-0.45042
0.192573	-0.05638	0.116136	-0.47366	0.475042	-0.42332	0.163339	-0.1665	-0.29562
0.431182	-0.48205	-0.23906	-0.28601	0.121932	-0.18439	0.303052	0.028696	-0.49878
0.27006	0.029371	0.079562	-0.4314	0.01357	0.117153	0.106163	0.311108	-0.27426
-0.52407	-0.53822	0.333526	-0.03339	0.420926	-0.39313	-0.30459	0.327381	0.034829
-0.16777	-0.11149	0.088288	-0.1384	0.079188	-0.07414	-0.25192	-0.14056	-0.13563
-0.37334	0.109041	0.402662	-0.3089	0.237553	0.017924	-0.38857	-0.03583	0.036556

0.012079	-0.28637	0.331668	-0.39775	-0.15577	-0.42121	-0.20213	0.071067	-0.18595
0.206874	-0.023	-0.01455	-0.18869	0.080916	-0.05189	-0.11456	-0.23652	-0.2704
0.032141	0.115837	-0.17493	-0.19542	0.040052	-0.0816	-0.3013	-0.11198	-0.05246
0.283982	-0.52576	0.227922	-0.36768	0.032232	-0.04108	0.018446	-0.36263	-0.39229
0.242008	-0.31971	0.225195	-0.16968	-0.2897	-0.03076	0.00148	-0.23032	-0.3706
-0.0164	-0.3539	0.23819	-0.34686	-0.19889	-0.18649	-0.231	-0.11849	-0.17633
-0.10956	-0.32892	0.161971	-0.44638	-0.13419	-0.25222	-0.40168	-0.18289	-0.2126
0.147602	-0.06348	0.151695	-0.25199	0.283682	-0.33747	-0.2201	0.019712	-0.24294
0.222354	-0.02954	-0.33754	-0.27722	0.314844	-0.4754	0.023618	-0.12709	-0.27205
0.07071	-0.44035	-0.08611	-0.08354	-0.25907	-0.40118	0.287945	-0.13032	-0.292
0.193167	-0.21558	-0.20309	-0.27213	-0.08214	0.051713	-0.22028	-0.40122	-0.31918
0.544731	-0.24639	-0.57473	-0.03738	-0.00918	-0.34335	0.660513	0.097747	-0.02357
0.005369	-0.33017	0.140966	-0.43331	-0.07285	-0.16022	-0.14313	0.379273	-0.28886
0.127113	-0.42242	0.019758	-0.2994	0.158116	-0.11442	0.260249	0.32574	-0.19648
0.085605	0.16132	-0.11127	-0.2129	-0.05941	0.341319	0.14807	-0.03074	-0.18046
-0.33127	-0.35862	0.218604	-0.06315	0.09062	0.137408	-0.10597	0.008023	0.08506
-0.10529	0.217491	-0.64543	0.738858	0.054318	0.413481	-0.01842	-0.0672	0.082636
-0.03121	-0.0273	0.080765	0.500161	-0.16596	0.419891	-0.07056	0.07709	0.168408
0.430723	-0.10694	-0.04276	-0.02177	-0.38915	-0.17786	0.271766	0.144007	-0.26895
-0.10026	-0.29214	-0.03809	-0.0985	-0.08445	-0.15228	-0.22336	-0.06391	-0.24607
-0.41564	-0.21613	0.422537	-0.34292	-0.1949	-0.25192	-0.58711	-0.09549	-0.18449
0.154539	-0.41764	-0.06159	-0.36024	-0.03899	-0.2638	-0.21039	-0.38988	-0.5116
0.026738	-0.45374	0.199125	-0.20489	-0.30082	-0.20822	-0.01952	-0.17149	-0.08615
-0.35966	0.008762	0.145571	-0.38161	0.203678	0.495913	-0.52482	0.289363	-0.24324
-0.51125	0.049422	0.288162	-0.03234	0.242056	-0.33566	-0.17365	0.017972	0.293817
0.246081	-0.08951	0.179265	-0.29629	-0.1058	-0.05559	0.269379	0.319044	-0.32405
-0.11579	-0.21008	0.064232	-0.18492	0.047161	-0.09956	-0.1375	-0.18124	-0.41738
-0.59617	-0.45504	0.177516	-0.23443	-0.29403	-0.32236	-0.34654	0.486433	-0.42046
0.071148	0.076336	0.277462	-0.15535	0.034162	-0.13167	-0.30715	0.369796	0.104152
0.021295	-0.07864	-0.0284	-0.14965	0.210639	-0.06558	0.283048	0.213016	0.153369
0.329938	-0.22019	0.089184	-0.35177	0.072301	-0.17337	0.126708	0.430411	-0.41534
0.375562	-0.25856	0.033271	-0.33597	-0.08219	-0.16928	0.179816	0.39853	-0.44726
0.041879	0.231723	0.179744	-0.35316	0.191983	0.143517	0.064361	0.14747	-0.15827
-0.09391	-0.01923	0.004328	-0.33712	-0.36832	0.010899	-0.44046	-0.23705	-0.31603
0.189282	-0.11192	0.024157	-0.17609	-0.31307	-0.35705	0.23464	0.202144	-0.31237
-0.69439	-0.50987	-0.24513	0.037479	0.208981	0.041449	-0.52381	0.573503	-0.57128
-0.47061	-0.19219	0.239458	-0.18058	0.114584	0.231287	-0.52366	0.031006	0.020034
-0.44177	-0.05486	0.434415	-0.32319	0.145757	-0.21185	-0.57652	-0.08865	-0.2629
-0.66487	0.423434	0.523331	-0.42733	0.389427	0.431342	-0.50382	-0.29866	0.291991
-0.37472	-0.0376	0.266323	-0.19673	-0.03879	0.160938	-0.43684	0.171079	0.124843
-0.67322	0.138525	0.245146	-0.20191	0.295511	-0.35654	-0.49885	0.182648	0.459362
-0.52968	-0.52987	0.323544	-0.20376	0.277905	0.340497	-0.55246	-0.23001	-0.00288
0.124078	-0.25188	-0.00175	-0.06732	-0.17181	-0.04822	0.080637	0.092662	-0.24608
-0.39407	0.252925	0.444702	-0.37176	0.033006	0.214401	-0.27744	0.129836	0.37267
-0.53155	0.171	0.254484	-0.17941	0.18974	0.363291	-0.33607	-0.28452	-0.03564
-0.61885	0.248571	0.518525	-0.49384	0.491278	0.557164	-0.43324	-0.41885	-0.07345
-0.61325	0.392908	0.605858	-0.35584	0.535367	0.351459	-0.61671	-0.02193	0.082566
0.122955	0.091508	-0.33679	0.129739	-0.17839	-0.10701	0.186077	-0.15736	-0.16455
0.292226	0.152663	-0.2752	-0.14599	-0.19741	0.213083	0.195624	-0.24751	-0.21153
0.225125	-0.11731	-0.05271	0.196417	-0.28964	-0.12281	0.22099	-0.16505	-0.29362

0.225937	-0.28389	-0.44298	0.30837	-0.1379	-0.26352	0.272962	0.154469	-0.23909
0.276788	-0.33783	-0.37459	0.222828	-0.25468	-0.34667	0.342999	0.080926	-0.25178
0.355593	-0.06124	-0.03828	0.012556	-0.4587	-0.16799	0.345683	0.104065	-0.24315
0.299807	-0.1822	-0.30164	0.236937	-0.04	-0.18402	0.301869	-0.09979	-0.19367
-0.46554	0.285299	0.261253	-0.36393	0.339168	0.329468	-0.3255	-0.10516	0.092094
-0.1297	0.106186	0.06119	-0.23687	-0.00854	0.059979	-0.05712	-0.23422	-0.11467
-0.29174	0.224065	0.074797	-0.02708	0.133674	0.284057	0.046483	0.046882	0.179923
-0.27615	-0.08696	0.011234	-0.13459	-0.02466	0.220802	-0.28567	-0.10298	0.034818
0.177219	0.165117	0.183184	-0.01304	-0.2062	0.04271	0.082679	-0.15148	-0.00811
0.189316	0.042669	-0.29341	-0.02429	-0.20999	-0.10643	0.318556	-0.03922	-0.03577
0.001528	-0.16217	0.161468	-0.51848	-0.20746	-0.20146	-0.17965	0.103281	-0.09854
0.327476	0.285637	-0.13991	-0.10574	0.027482	-0.11136	0.11413	-0.21931	-0.09575
0.159288	-0.02174	-0.22564	-0.07577	-0.18478	-0.10321	0.289254	0.077431	-0.06719
0.341085	-0.0363	-0.32254	0.271577	-0.21621	-0.20857	0.364734	-0.00341	-0.25492
0.012137	-0.04129	-0.16209	0.06926	-0.188	0.205665	0.112263	-0.27004	-0.00992
0.364362	0.449729	-0.255	-0.08202	0.049542	-0.11466	0.223021	-0.00124	-0.35241
0.325386	0.249853	-0.17901	0.168361	-0.05017	0.15693	0.493086	0.154741	-0.08192
0.056246	0.117009	-0.37918	0.249652	-0.2089	0.199815	0.387863	-0.12494	-0.01008
0.213728	0.052029	-0.42198	0.28432	-0.23601	0.226682	0.19547	-0.20504	-0.24051
0.236962	0.252236	-0.39569	-0.00804	-0.02433	0.144605	0.385924	-0.28166	-0.07848
0.214526	0.086094	-0.35436	0.003565	-0.22776	-0.03926	0.330408	0.105649	-0.03452
0.265039	0.217793	-0.49456	0.191688	-0.22807	0.263061	0.330273	-0.1742	-0.203
0.271587	0.280872	-0.5657	0.350696	-0.26989	0.397796	0.400282	-0.03083	-0.20859
0.328031	-0.43514	-0.5959	0.36739	0.035038	-0.31501	0.469629	-0.09059	-0.44198
0.289116	0.236295	-0.32725	-0.08961	-0.08768	0.232049	0.186128	-0.2671	-0.08666
0.161109	0.17511	-0.3375	-0.11407	-0.16386	0.149691	0.258	-0.15712	0.019744
0.383118	0.175086	-0.24668	0.129383	-0.2286	-0.03217	0.336427	-0.23384	-0.21776
0.463654	-0.2497	-0.53887	0.119022	0.384487	-0.13261	0.435214	0.114474	-0.36269
0.156245	-0.45791	-0.3728	0.114357	-0.08814	-0.47278	0.222598	0.287184	-0.39617
0.246983	-0.28196	-0.45332	0.166153	-0.17401	-0.28463	0.289277	-0.00693	-0.00101
-0.12009	-0.39906	-0.37992	-0.04477	-0.19183	-0.27788	0.085441	0.252908	0.133467
0.07803	-0.05222	-0.25597	0.137288	-0.22921	-0.16963	0.165408	0.195455	0.182546
-0.11035	-0.21426	-0.01704	-0.04109	-0.11893	-0.32886	-0.07556	0.283427	-0.16563
0.337019	-0.18632	-0.34771	0.175672	-0.28292	-0.18173	0.220068	-0.0544	-0.19096
0.316126	-0.3726	-0.47844	0.318945	-0.09531	-0.24348	0.382558	0.249937	-0.31627
0.208733	-0.19858	-0.33515	0.151995	-0.22904	-0.22427	0.240158	-0.1175	-0.16618
0.297813	-0.18087	-0.35318	0.065983	-0.2838	-0.12024	0.22715	-0.19041	-0.17847
0.293933	-0.14956	-0.40525	0.109227	-0.23947	-0.25305	0.309797	-0.1886	-0.19074
0.228804	-0.14704	-0.19009	-0.02786	-0.31838	-0.15479	0.282007	0.065756	-0.17279
0.240467	-0.03637	-0.22467	0.040694	-0.19763	-0.24802	0.445476	-0.01197	-0.06286
0.257358	-0.1243	-0.45843	0.151661	-0.05534	-0.11106	0.46578	-0.01138	-0.11239
0.363798	-0.55614	-0.5507	0.429081	-0.03296	-0.54024	0.263795	0.254756	-0.5912
0.308124	0.08235	0.126915	0.012782	-0.33931	-0.02074	0.142851	-0.18613	-0.08074
0.303234	0.037479	-0.15286	0.045873	-0.36713	0.060161	0.244359	-0.17661	-0.10382
0.302653	0.093072	-0.25806	-0.08477	-0.21034	0.11178	0.336184	-0.06347	-0.25179
0.083286	0.085537	0.541421	0.266933	-0.04755	-0.02215	0.217566	0.194681	0.186849
0.160994	0.054475	-0.41141	0.15826	-0.18704	0.033466	0.303055	-0.20793	-0.04148
0.263108	0.197103	-0.16128	-0.01078	-0.11289	-0.02242	0.0879	-0.2201	-0.04383
0.309497	0.026035	-0.14452	0.036766	-0.23362	0.000422	0.312834	-0.03882	-0.17648
-0.07372	0.094441	-0.10147	-0.0444	-0.0011	0.06275	0.118246	-0.25393	-0.0121

0.247477	0.017816	-0.27695	0.305079	-0.23478	0.053394	0.311843	-0.16266	0.034434
0.348509	0.035455	0.080668	-0.13089	-0.23362	-0.14026	0.231802	-0.02676	-0.08042
0.252053	-0.12644	-0.2563	-0.10189	-0.26876	-0.12445	0.404618	-0.09738	-0.26629
0.087365	-0.5822	-0.5309	0.246319	-0.01139	-0.63509	0.490046	0.186355	-0.37183
0.514892	-0.3362	0.247479	-0.50806	0.47853	-0.48511	0.650263	0.294675	-0.41041
0.408128	-0.23949	-0.07399	-0.20485	-0.23882	-0.09147	0.263888	-0.07173	-0.35017
0.333946	-0.19482	-0.15818	-0.14201	-0.20622	0.008411	0.288294	-0.19011	-0.07399
-0.32557	-0.40101	0.287925	-0.07145	0.082099	-0.26945	-0.23047	0.257718	-0.00236
-0.49216	-0.517	0.437244	0.008209	0.325013	-0.40417	-0.28347	0.353051	0.089874
-0.07142	0.083504	-0.09218	-0.17549	-0.2731	-0.25339	-0.03685	0.012357	0.134013
-0.41044	-0.38375	0.357331	-0.09892	0.183169	-0.2122	-0.35076	0.257231	0.047453
0.253408	-0.35189	-0.5733	0.280393	-0.05978	-0.32143	0.37119	-0.10939	-0.34099
-0.11451	-0.29941	0.004159	0.010576	0.056542	-0.3145	0.305221	-0.03513	0.096843
-0.13351	-0.24011	-0.2676	-0.14805	-0.19883	-0.34215	0.250094	-0.01664	-0.0204
0.202551	-0.57364	-0.60728	0.301548	-0.04363	-0.48143	0.471424	0.176271	-0.27806
0.117249	0.170412	-0.3441	0.086081	-0.22781	0.154313	0.294115	-0.0696	-0.0368
-0.03001	0.011683	-0.19905	0.13065	-0.25247	0.193649	0.188973	-0.34892	-0.18793
0.325216	0.087095	-0.32838	-0.10776	-0.24193	0.160251	0.289341	-0.1122	-0.2986
0.143036	0.133382	-0.27396	-0.02151	-0.02963	0.164948	0.201028	-0.30765	-0.1647
0.272291	0.082368	-0.14387	-0.03136	-0.26034	0.19125	0.270263	-0.24648	-0.20427
0.306529	0.157183	-0.1586	0.04548	-0.25668	0.188581	0.233514	-0.2416	-0.12841
0.255745	0.122988	-0.21013	-0.06066	-0.26756	0.096325	0.143952	-0.10616	-0.12005
0.061962	0.226388	-0.34729	-0.08354	-0.2683	0.072506	0.317857	-0.1556	-0.19407
-0.04463	0.150183	-0.43853	0.429487	-0.19038	0.212576	0.411296	-0.18287	0.181542
0.186314	0.260529	-0.33215	0.182164	-0.15775	0.115861	0.229006	-0.25237	-0.12112
0.078915	0.385274	-0.24179	-0.07095	-0.00721	0.284231	0.327129	-0.18572	-0.1311
0.22196	0.286989	-0.27429	-0.06839	-0.31323	0.189456	0.233529	-0.21457	-0.15507
0.27506	0.08827	-0.41378	0.259482	-0.19349	-0.12753	0.304672	-0.03092	-0.28357
0.195648	0.306697	-0.29102	-0.10054	-0.14424	0.212604	0.306271	0.093331	0.014567
0.402318	0.285928	-0.18544	-0.10411	-0.12559	0.099443	0.260479	-0.18542	-0.09081
0.275402	-0.65277	-0.61854	0.5161	-0.17472	-0.51191	0.401226	0.283752	-0.62278
0.216098	-0.41008	-0.53764	0.539645	-0.21341	-0.48918	0.316546	0.090074	-0.49191
0.263194	-0.64151	-0.61494	0.547947	-0.10875	-0.66003	0.400065	0.105169	-0.54101
0.128922	-0.65198	-0.50641	0.465071	-0.0444	-0.68529	0.471212	0.124038	-0.46071
0.077431	-0.3484	-0.49645	0.407242	-0.32484	-0.31082	0.369559	0.061053	-0.25077

TCGA-HV-/	TCGA-HZ-7	TCGA-HZ-7	TCGA-HZ-7	TCGA-HZ-7	TCGA-HZ-8	TCGA-HZ-8	TCGA-HZ-8	TCGA-HZ-8
-0.06743	0.082171	0.213127	-0.24312	-0.14291	-0.0124	0.085421	0.13787	0.223935
0.45649	0.146438	-0.08848	0.213316	-0.48357	0.233314	0.364045	0.385348	0.210237
0.297842	0.015347	0.289388	0.032813	0.306598	0.001116	0.084119	0.00555	-0.13067
0.457214	0.111971	-0.2323	0.07441	0.227903	0.304178	-0.07743	-0.03745	-0.1181
0.617709	-0.20578	0.266255	-0.22294	0.383947	0.356415	-0.29877	-0.23288	-0.21447
-0.10896	0.014804	-0.04078	-0.03477	-0.14979	-0.072	0.04188	-0.18158	-0.06487
-0.17342	0.216069	0.165676	-0.40577	-0.04196	-0.24647	-0.35901	-0.42277	-0.37248
-0.06913	-0.18894	-0.15521	-0.02758	-0.14395	-0.05447	-0.08152	-0.26244	-0.12936
-0.10577	-0.09357	0.05124	0.183644	0.034798	0.08895	0.085735	-0.04693	-0.3605
0.214474	-0.28634	-0.08279	0.36138	-0.00366	0.188841	-0.04782	-0.14539	-0.33094
0.006991	-0.37393	-0.02322	0.239454	-0.05164	0.01419	-0.18615	-0.00519	-0.41058
-0.10216	-0.25263	0.062175	0.084295	0.100166	0.215865	-0.16739	0.231339	-0.32313
0.193937	0.042669	0.15123	-0.02402	-0.08158	-0.17586	0.07402	0.032176	-0.17545
0.075426	-0.06202	-0.10288	-0.09048	0.032644	-0.18808	-0.25131	-0.18706	0.067026
0.33906	-0.06142	0.453353	0.178269	0.358597	0.141029	-0.0898	0.008514	-0.08059
0.213044	0.28634	0.322124	-0.02986	0.22472	0.220293	0.169503	0.03541	0.274648
0.007359	0.224801	0.22597	-0.12236	-0.08273	0.045966	-0.3614	0.301597	-0.37665
-0.02755	0.185289	0.032862	0.182437	0.008615	0.28877	0.016142	0.240522	-0.24361
0.007793	-0.25697	0.215033	-0.03734	0.133576	0.193589	-0.10573	0.33943	-0.1971
-0.60713	-0.48938	-0.30318	-0.68059	-0.29248	-0.12274	-0.24758	-0.55941	0.739829
-0.31045	0.216621	0.076972	-0.28312	0.183279	0.128226	0.095606	-0.02674	0.419433
-0.64333	0.294805	0.329158	-0.02429	-0.1851	-0.21986	0.282342	0.210577	0.422089
-0.04246	-0.17876	-0.15026	-0.07248	-0.15447	0.181388	-0.12074	-0.02417	-0.01065
0.43291	0.098015	-0.11879	0.231836	0.046847	0.281467	0.040354	0.233274	0.140252
-0.00605	-0.08813	-0.33998	-0.1958	-0.02653	0.341259	-0.24083	-0.38562	-0.40379
0.434243	0.181287	-0.10201	0.439259	-0.0049	0.394227	0.173364	0.037383	0.195366
-0.14224	0.124563	-0.02618	0.139796	0.312858	-0.37658	-0.10146	-0.1229	0.326254
-0.09698	0.31481	-0.03569	0.261241	-0.15286	-0.19589	0.152533	0.037217	-0.10307
0.067154	0.098505	-0.22201	0.097938	-0.42481	-0.04259	0.223662	0.305091	-0.45234
0.199168	0.133616	0.311745	0.02071	0.124071	-0.08158	0.086794	-0.01723	0.04349
0.072027	0.342463	0.083328	0.601469	0.302615	0.568856	0.495469	0.481488	0.392861
-0.32757	0.210035	-0.18513	0.425283	-0.10306	0.431663	-0.05836	0.419275	0.102801
0.010953	0.003933	0.274546	0.19386	0.340429	0.07984	0.082796	-0.0006	0.143764
0.050851	0.161717	0.353281	0.110364	0.341127	0.033925	0.120044	0.022503	0.336766
0.128647	0.153053	0.244232	0.288911	0.359026	0.162411	0.220418	0.129332	0.066083
0.151266	-0.15717	0.096523	0.100762	0.087478	0.304649	-0.00217	-0.10765	-0.00815
-0.56644	0.272682	-0.09735	-0.42289	-0.01986	0.431263	0.169505	0.319538	-0.35762
-0.04957	-0.35446	-0.07654	-0.0846	-0.15305	0.215569	-0.3285	-0.18582	-0.0098
-0.40208	-0.28057	0.108742	-0.08843	-0.12044	0.012762	0.256865	-0.17853	-0.1773
0.133197	-0.10033	0.19858	-0.09898	-0.18366	-0.01092	-0.32109	-0.09316	0.007688
0.333338	-0.02304	0.044588	0.160654	0.096308	0.344118	-0.03945	0.00912	0.387082
-0.27781	-0.19093	0.035937	0.038808	-0.11217	-0.09618	0.364466	-0.18598	-0.33762
-0.19369	0.092003	-0.14682	0.201302	-0.21774	0.389101	0.281893	0.291227	-0.53224
0.125371	-0.40185	0.15439	-0.37392	-0.33626	0.122688	-0.40154	0.060296	-0.59673
-0.12496	0.184123	-0.03852	0.571546	0.024088	0.266653	0.445409	0.168179	-0.5386
-0.05313	-0.2562	0.142768	0.131717	-0.12805	-0.01863	0.088102	-0.20698	-0.38117
-0.21193	-0.15692	-0.57995	-0.52548	-0.36299	0.372162	-0.38637	-0.52247	-0.48312
-0.17194	0.071312	-0.02837	-0.18017	0.192494	0.19944	-0.02809	-0.08999	0.055968
-0.37707	-0.12994	-0.06237	-0.4567	0.001699	0.198703	-0.21791	-0.19051	0.1973

-0.32443	-0.09483	-0.10158	-0.07675	-0.12605	0.006025	-0.1007	0.211035	0.018042
0.039169	-0.18609	-0.23707	-0.15258	-0.29347	0.167703	0.245546	0.471379	-0.26333
-0.10316	0.097596	0.076906	-0.10579	0.150674	-0.18065	0.118446	0.304711	0.143459
-0.37288	-0.05469	-0.12735	-0.19477	-0.44039	0.216436	0.135188	0.421103	-0.34252
0.001227	0.156547	-0.00411	-0.0556	-0.11982	-0.093	0.322225	0.343087	0.235036
-0.0022	-0.19493	-0.22052	-0.2234	0.012484	0.02452	-0.03465	-0.01021	-0.1328
-0.35679	-0.15094	-0.26449	-0.214	-0.24982	0.116432	-0.1649	-0.28634	-0.41738
-0.18503	-0.1563	-0.33381	0.005201	-0.32659	0.328069	-0.13052	-0.2494	-0.19957
0.067258	-0.18835	-0.36914	-0.08126	-0.19263	0.204161	-0.08693	-0.28405	-0.27182
-0.08276	0.276373	-0.25661	0.158666	-0.10474	0.387989	0.142169	0.338505	-0.26921
-0.30418	0.119339	-0.06329	0.109353	0.038298	0.208795	0.504549	0.308659	-0.31178
0.311882	-0.11142	-0.20128	0.512522	-0.22125	0.201242	0.41472	0.11965	-0.59276
-0.27283	-0.02301	-0.20233	-0.15161	-0.09341	0.04855	0.052798	0.205712	-0.14911
0.017206	-0.19814	-0.2338	-0.03924	-0.1332	0.144535	0.060404	-0.10396	-0.33793
-0.04329	-0.09712	0.316941	0.176995	0.130544	0.05086	0.159043	0.252807	0.146321
0.097346	-0.11327	0.290039	-0.12099	0.184574	0.380537	-0.01056	0.199056	-0.03132
0.696978	-0.05777	0.129537	-0.15965	0.399127	0.113986	-0.0235	-0.10012	0.387454
0.588204	0.096389	0.230311	-0.12693	0.27016	0.086101	0.047004	-0.06986	0.146062
0.001345	0.040705	0.104857	0.199767	-0.10305	-0.39335	0.127288	0.141485	-0.20516
-0.21156	0.013591	-0.24013	-0.2008	-0.30517	0.262729	0.020992	-0.06035	-0.30871
-0.55899	0.149372	-0.36961	-0.52374	-0.28104	0.453581	-0.33068	-0.02829	0.084767
-0.20693	0.056809	-0.04462	-0.00671	-0.21044	0.275263	0.394359	0.378255	-0.36201
-0.26401	0.107068	-0.19397	-0.13774	-0.32578	0.407408	0.155564	0.119789	-0.43062
-0.37459	-0.26884	0.100317	-0.34976	-0.02048	0.226682	0.171173	0.019081	0.16987
0.01338	0.051195	-0.27145	-0.38993	-0.07819	0.530508	-0.29711	-0.378	-0.22643
-0.18841	-0.16288	0.066895	0.225988	-0.06185	0.085958	0.214755	-0.18553	-0.36514
-0.13701	-0.28456	-0.20156	-0.34593	-0.08073	0.275483	-0.09023	-0.34464	-0.24159
-0.05891	-0.21311	0.270462	-0.55888	0.151566	0.147616	-0.58394	0.032788	-0.5042
-0.36259	-0.15988	0.025007	-0.11315	-0.40103	-0.37122	-0.04828	0.272636	-0.29634
-0.18012	-0.072	0.056067	0.013866	0.165065	0.388413	0.019652	0.013954	-0.34359
-0.19742	-0.28751	-0.12007	0.145248	-0.13717	0.275416	0.13951	-0.15565	-0.47667
-0.11401	-0.2981	-0.1105	0.17535	-0.1702	0.178967	0.198959	-0.05309	-0.46723
-0.28832	-0.30606	-0.10934	-0.20793	-0.17636	0.039562	-0.15592	-0.29538	0.017062
-0.11298	-0.04444	0.145715	-0.39256	-0.27654	0.133994	-0.00313	-0.30575	-0.1213
0.109979	0.143832	0.003247	0.241647	0.159337	-0.08354	-0.00883	0.118429	-0.07814
-0.55965	0.292292	-0.45592	-0.01645	-0.3343	0.659694	0.133153	-0.30959	-0.35044
-0.4661	0.185027	-0.04609	-0.21875	-0.10896	0.125701	0.040555	-0.14169	0.32911
-0.3967	0.215861	-0.05517	-0.3271	0.278494	0.484107	-0.10609	-0.10604	0.375574
-0.67383	-0.49587	0.011573	-0.61142	-0.40161	0.241009	-0.38744	-0.28238	0.352126
-0.47559	0.30639	-0.07121	-0.12276	-0.29009	0.183717	0.17168	-0.11078	-0.06335
-0.61908	0.320196	-0.03773	-0.601	-0.02737	0.558752	-0.20618	0.127515	0.201455
-0.46879	0.635428	0.215051	0.02148	-0.10668	0.595157	0.287994	0.509317	-0.22214
-0.0223	0.192307	0.021335	0.297663	-0.30164	0.24999	0.233548	0.269417	-0.34384
-0.42091	-0.48606	-0.22418	-0.56016	-0.13466	0.018565	-0.40506	-0.47578	-0.01141
-0.48069	0.051311	-0.06629	-0.3641	-0.19266	0.285525	-0.135	-0.1599	0.306684
-0.701	-0.17063	0.063523	-0.44758	-0.19941	0.225582	0.016821	-0.17789	0.312956
-0.51363	-0.5057	-0.06856	-0.53654	-0.22368	-0.06095	-0.2492	-0.37467	0.353961
0.015999	0.148497	0.126632	0.292231	0.179095	-0.00824	0.146571	0.22811	-0.00711
-0.14517	0.253868	0.290879	0.184623	0.146175	-0.37689	0.213313	0.395032	0.125456
0.057658	0.215442	0.147141	0.305818	0.162418	0.162732	0.201227	0.172487	-0.20144

0.016934	0.129001	0.07285	0.462047	0.279902	0.294455	0.291543	0.26787	-0.14989
-0.04343	0.266668	0.139454	0.409834	0.152899	0.118361	0.303055	0.433555	-0.22939
-0.15641	0.092767	0.025666	0.238557	0.011353	-0.34206	0.11143	0.051505	-0.14236
0.031005	0.246265	0.088645	0.320094	0.202305	0.265275	0.248101	0.254442	-0.25228
-0.48908	-0.06871	0.279961	-0.32975	0.096712	-0.23201	-0.00471	0.029805	0.36616
-0.18267	0.170964	0.221451	-0.09379	0.102762	-0.01524	0.257851	0.157907	0.004559
-0.18847	-0.09663	-0.01358	-0.16033	0.030364	-0.12941	-0.04218	-0.15378	0.165976
-0.28811	0.196642	0.255368	-0.14161	0.253361	-0.13708	0.179829	0.212961	0.278502
-0.29072	0.197367	0.106577	0.294938	-0.33311	0.126242	0.099681	0.363381	-0.05645
0.082782	-0.07117	0.219693	0.104136	0.119221	-0.11967	0.036245	0.062949	0.032793
-0.29716	-0.32358	-0.19346	-0.30191	-0.36368	0.174709	-0.1092	-0.01595	-0.41204
-0.07028	0.186707	0.182795	0.177652	0.083835	-0.21004	0.161825	0.359444	0.142731
-0.14274	0.047827	-0.05264	0.179978	0.077369	-0.12558	0.133173	0.138828	-0.1559
0.141106	0.202753	0.201184	0.367682	0.206276	0.083294	0.276063	0.295009	-0.31541
0.027303	0.214147	0.219434	0.198219	0.169537	-0.10229	0.12997	0.040889	0.153107
-0.0908	0.251707	0.451978	0.369787	0.376292	-0.4547	0.367338	0.359294	0.043828
0.280505	-0.36195	0.03067	-0.06514	0.004685	-0.38956	-0.08996	-0.01709	-0.09565
0.358361	-0.03857	0.335631	0.293449	0.218232	-0.21489	0.156301	-0.07882	0.150745
0.234233	0.334109	0.451909	0.399844	0.377818	0.006359	0.454707	0.280702	0.073575
0.188319	0.058799	0.199841	0.12878	0.203178	-0.12742	0.172806	0.004717	0.098033
0.028133	0.07179	0.215026	0.248171	0.204178	-0.09157	0.065762	0.183628	-0.02656
0.290531	0.180891	0.400934	0.255864	0.382592	-0.02136	0.306224	0.325388	0.327534
0.468874	0.043317	0.444989	0.346584	0.439289	0.189721	0.294525	0.279821	0.380939
0.127733	0.393401	0.170984	0.489327	0.209452	0.334151	0.344366	0.353856	0.016853
0.14445	0.290707	0.489359	0.234272	0.269326	-0.25509	0.370905	0.265024	0.292663
0.111777	0.004173	0.32548	-0.06632	0.057005	-0.13386	0.075978	-0.02837	-0.06148
0.286371	0.277669	0.270461	0.183536	0.130448	-0.21508	0.280361	0.263788	-0.17812
0.402646	0.376637	-0.00081	0.500862	0.002018	0.298012	0.391088	0.24657	-0.16531
-0.17607	0.311162	-0.13967	0.425732	0.260037	0.351702	0.258992	0.391934	0.517933
-0.11902	-0.00913	0.204816	0.402418	0.17965	-0.02041	0.17882	0.428123	0.204134
-0.22105	0.088513	0.083721	0.439215	0.318956	0.032067	0.291297	0.272165	0.126702
-0.05166	-0.17908	0.099662	0.139302	0.072297	-0.15661	-0.05012	0.2288	0.026733
-0.28731	-0.09549	-0.17995	0.115449	0.14941	0.373503	0.008868	0.168871	-0.08366
-0.13401	0.287474	0.202286	0.525121	0.164138	0.033958	0.335163	0.360694	0.026905
0.100719	0.361569	0.150158	0.561835	0.189023	0.457098	0.291532	0.307558	0.028465
-0.11775	0.258791	0.136246	0.365287	0.081176	0.168228	0.208838	0.370529	0.296004
-0.18597	0.258323	0.348423	0.352586	0.156815	-0.13567	0.282293	0.435625	-0.03176
-0.13664	0.268488	0.266402	0.345877	0.146949	-0.06794	0.256562	0.436773	-0.1116
-0.25241	0.187843	0.308015	0.424744	0.087037	-0.02254	0.221404	0.386809	-0.16624
-0.01395	-0.0278	0.206623	0.252361	0.201139	0.011185	0.160774	0.257134	-0.07444
0.076256	0.225019	0.304788	0.352663	0.185448	-0.04309	0.269021	0.195141	-0.01397
-0.19729	0.312084	0.053497	0.596182	0.080235	0.468026	0.255891	0.401839	0.212061
-0.15541	0.275272	0.253766	0.245098	0.106145	-0.15339	0.241176	0.316897	-0.24651
-0.01055	0.206877	0.242323	0.320366	0.114487	-0.3228	0.255932	0.327828	0.033791
-0.02019	0.314082	0.26836	0.250612	0.21767	0.084975	0.175828	0.30512	-0.238
0.179734	0.238948	0.073834	0.29323	0.210883	0.086523	0.21042	0.278532	-0.08571
0.134937	0.130893	0.314879	0.202333	0.272879	-0.05485	0.217475	0.261469	0.16509
-0.01289	0.118089	0.161397	0.249554	0.064995	-0.20359	0.16025	0.279463	-0.05008
0.04353	0.109554	0.193933	0.323981	0.098531	-0.22855	0.197807	0.225655	-0.11362
-0.23532	0.195169	0.336275	0.091282	0.1936	-0.09081	0.228581	0.293251	0.216556

0.313444	0.210958	0.254677	0.31639	0.220628	-0.08178	0.214047	0.145269	-0.07048
-0.12158	0.082613	0.124574	0.246879	0.106187	-0.01995	0.159597	0.235035	-0.01171
-0.07017	0.412481	0.269427	0.421657	0.219635	-0.00646	0.306963	0.31706	-0.04564
-0.35827	0.303377	-0.20443	0.521731	0.264782	0.466002	0.143521	0.485823	0.442663
0.113495	0.332989	-0.43276	0.290818	-0.48521	0.137321	0.379597	0.374279	-0.59864
-0.23322	0.24355	0.363967	0.480196	0.191842	-0.08114	0.275157	0.18378	-0.23042
-0.03821	0.272697	0.100822	0.240353	0.126141	0.02166	0.355465	0.151767	-0.33827
-0.26756	-0.10836	-0.308	-0.3619	-0.15153	0.153506	-0.23981	-0.29155	-0.3701
-0.19489	-0.24911	-0.58268	-0.49276	-0.33764	0.337855	-0.34924	-0.44774	-0.50755
-0.24107	0.049761	-0.03458	0.023312	-0.02566	-0.0148	0.013865	-0.02177	-0.17797
-0.26544	-0.16943	-0.38562	-0.45588	-0.15091	0.141559	-0.2638	-0.36077	-0.31597
0.218911	0.296852	-0.07179	0.408495	0.329925	0.169265	0.406007	0.302069	-0.04024
0.201224	0.205796	0.14253	-0.16524	0.115097	0.10618	0.113839	0.112892	-0.13255
-0.13806	0.201746	0.118746	0.111978	0.172094	-0.03482	0.161627	0.137621	0.03666
-0.00058	0.117166	0.265172	0.528567	0.38382	0.259554	0.324137	0.523295	0.357797
0.159948	0.025766	0.275321	0.194205	0.256498	-0.13623	0.198063	0.164594	0.167709
-0.05974	0.233724	0.290137	0.179332	0.170909	-0.18706	0.212569	0.333495	0.304789
-0.15349	0.268071	0.302205	0.330909	0.190547	-0.2419	0.363065	0.484917	0.010506
-0.03123	0.049353	0.315315	0.082529	0.178961	-0.26113	0.208614	0.340251	0.305409
-0.04402	0.211279	0.30438	0.225594	0.118139	-0.3686	0.219961	0.432991	0.263858
-0.03167	0.34688	0.371664	0.270871	0.324181	-0.2459	0.3786	0.38704	0.125445
0.051485	0.150878	0.276022	0.214144	0.269183	-0.29894	0.323245	0.411788	0.201014
-0.01266	0.245229	0.27263	0.036883	0.113475	-0.14233	0.19911	0.400304	0.102834
0.5032	-0.28758	0.34479	0.227384	0.247333	-0.06424	0.093235	-0.14377	0.257586
0.059594	0.266755	0.277376	0.183433	0.292235	-0.20741	0.265572	0.320887	0.196609
-0.10471	0.024035	0.097759	-0.02107	0.044093	-0.24138	0.130858	0.260993	0.18216
-0.09163	0.190376	0.311099	0.15434	0.164359	-0.36645	0.199826	0.35265	0.176883
0.044163	0.058908	0.273113	0.290723	0.139231	-0.27739	0.125458	0.451066	0.202091
-0.02142	-0.05422	0.292025	0.124766	0.273865	-0.25541	0.149438	0.224199	0.283746
-0.1304	0.162237	0.215337	0.30656	0.00168	-0.28922	0.236829	0.412825	0.205408
-0.27879	0.311435	0.0464	0.728009	0.284898	0.673455	0.343737	0.469262	0.308822
-0.10582	0.326802	-0.19297	0.597851	0.29587	0.512979	0.258602	0.443146	0.45442
-0.25181	0.234136	-0.21541	0.58593	0.270386	0.494178	0.239718	0.466755	0.543658
-0.28301	0.319509	-0.21642	0.586105	0.339482	0.558448	0.25643	0.488129	0.574266
0.133868	0.13241	0.173983	0.416387	0.343155	0.256921	0.065689	0.343642	0.475623

TCGA-HZ-8	TCGA-HZ-8	TCGA-HZ-8	TCGA-HZ-A	TCGA-HZ-A	TCGA-HZ-A	TCGA-HZ-A	TCGA-IB-7	TCGA-IB-7
0.338897	-0.02138	-0.06767	-0.4353	-0.02627	-0.17766	-0.30394	-0.0714	0.325649
-0.16402	0.037623	0.039731	-0.53266	-0.1742	-0.13566	-0.21436	0.372703	0.394324
0.413505	-0.1791	0.46048	0.233312	0.156963	0.19151	0.184931	0.293923	0.265448
0.032865	-0.48918	-0.18556	-0.03915	0.602819	-0.40787	-0.3552	0.183513	0.300192
0.077387	-0.21355	-0.00621	0.181104	0.324647	-0.20621	-0.03586	-0.07998	-0.11756
0.263521	-0.11042	-0.31865	0.02474	-0.04956	0.196415	0.066538	0.066553	-0.10134
0.064594	-0.18422	-0.01093	-0.54963	-0.45608	0.227758	0.020506	-0.40855	-0.25523
0.074294	-0.13677	-0.39997	0.025607	-0.17877	0.167835	0.096572	-0.15937	-0.22069
0.356682	-0.38341	-0.07115	0.154403	0.096505	0.253381	-0.20456	0.108122	-0.02194
0.168229	-0.31309	-0.2334	0.337805	0.152051	0.121051	-0.34212	-0.02668	-0.13166
0.451265	-0.41661	-0.09347	0.294735	0.020496	0.252621	-0.03509	-0.00871	-0.14689
0.44471	-0.40058	-0.06079	0.29976	0.226048	0.099204	-0.14805	-0.01489	-0.21331
0.063344	0.163916	0.086433	-0.18186	-0.09089	0.023799	-0.03851	0.013917	0.38749
0.068526	-0.18768	0.035123	-0.17781	0.04467	0.009176	0.366766	-0.25943	-0.09252
0.087977	0.007435	0.244141	0.239354	0.650502	-0.27116	-0.47231	0.213946	0.050354
0.355353	0.227527	0.126273	0.207627	0.488743	-0.40043	-0.28734	-0.05922	0.116409
0.116026	0.125319	0.347387	-0.28857	-0.12816	0.118859	0.424392	0.073931	0.162305
0.443552	0.022704	-0.07824	0.140843	0.269697	0.030115	0.246553	0.210474	0.015928
0.144038	-0.29854	-0.33949	-0.3254	-0.172	-0.07008	-0.22726	-0.15723	0.074853
-0.32999	-0.28015	0.002044	-0.26769	-0.67458	0.652906	0.257629	-0.21701	0.067188
-0.10072	-0.13858	-0.02327	-0.03275	0.117368	0.041388	0.246252	0.124584	0.07826
-0.10402	0.332553	0.584605	-0.28819	-0.48681	0.337445	-0.20454	0.489289	0.536164
-0.23161	-0.33181	-0.00591	-0.20471	-0.20131	-0.00119	-0.08381	-0.10386	-0.11015
-0.3782	-0.01176	0.077658	-0.1942	0.303403	-0.36523	-0.28299	0.349818	0.405605
0.084418	0.195818	-0.32935	0.401979	0.025369	0.150289	0.221116	-0.06983	-0.3087
0.43162	0.152808	-0.21952	0.404223	0.493241	-0.21415	-0.32842	0.341269	-0.05481
-0.02647	-0.06169	-0.27528	-0.3851	0.267557	-0.42383	0.450899	0.115728	0.097037
0.087523	0.226157	0.070523	0.067126	-0.08026	-0.00184	0.387832	0.216101	-0.01855
0.64907	-0.00485	-0.23779	0.42307	-0.19911	0.194648	-0.19617	0.138481	0.058488
-0.09945	-0.14173	0.381872	0.036063	0.423551	-0.0989	-0.06056	0.261435	0.079584
0.068461	0.204718	0.310369	0.320549	0.482569	-0.57688	-0.48733	0.590939	0.512028
-0.45307	-0.01345	-0.09238	-0.36931	0.390974	-0.41793	-0.50679	0.269678	-0.24872
0.341385	0.028403	0.263978	0.356622	0.461939	-0.19905	0.143507	0.330393	0.039922
0.226744	-0.11043	0.314556	0.258179	0.383534	-0.30711	0.1072	0.274805	0.21518
0.347773	0.039529	0.216933	0.351395	0.472286	-0.25291	0.11013	0.325814	0.033204
0.218481	-0.23798	0.170542	0.337624	-0.02211	0.056408	-0.15948	-0.04682	-0.00372
0.497657	0.184264	-0.50919	0.563737	-0.28423	0.153087	0.098422	0.144057	0.117934
-0.18608	-0.43823	-0.12622	-0.06352	0.004725	-0.09753	0.149379	-0.19256	-0.04489
0.358231	-0.13922	0.081606	0.093122	-0.08795	0.345541	0.469044	0.122774	-0.03587
0.002238	-0.32178	-0.05654	-0.08077	-0.01097	0.152815	0.222913	-0.09913	0.164836
0.250384	-0.17387	-0.01229	-0.06429	0.299955	-0.24326	0.006002	0.139026	0.190629
0.467243	-0.14705	-0.06778	0.409449	-0.21613	0.442297	0.375252	0.023633	0.133741
0.479893	-0.04345	-0.3557	0.509919	-0.16829	0.235292	-0.0023	0.045243	-0.05971
0.064143	-0.41267	0.020539	-0.66421	-0.4476	0.492656	0.799173	0.010929	0.212602
0.447513	0.351872	-0.1623	-0.07276	-0.09579	-0.0803	-0.41101	0.454437	0.518029
0.199888	-0.2681	-0.05067	0.017961	0.023731	0.255973	0.228931	-0.01207	0.016505
-0.17725	0.255264	-0.48274	0.475876	-0.25969	0.262651	0.106717	-0.3025	-0.24277
0.116578	-0.02566	-0.11742	0.091546	-0.00255	0.175436	-0.14299	-0.0811	-0.17008
-0.01756	-0.24611	-0.14721	0.008892	-0.11969	0.298503	0.012451	-0.2409	-0.37926

0.343418	0.050966	-0.31865	0.118217	-0.28871	0.250589	-0.1594	-0.19113	-0.30424
0.562442	-0.30078	-0.42277	0.096108	-0.28574	-0.29801	-0.12249	-0.15168	0.130865
0.37911	-0.20127	-0.06395	-0.02954	-0.11512	0.239022	0.058917	0.075942	0.024621
0.540625	-0.16607	-0.46817	0.433642	-0.39259	0.083896	0.309696	-0.0601	0.10733
0.248885	0.046419	0.024895	0.147554	-0.39847	0.333615	0.023474	-0.0352	0.185608
0.490552	-0.0504	-0.09	0.290199	-0.13821	0.262004	-0.21932	-0.13006	-0.10049
0.361517	-0.28208	-0.25426	0.092909	-0.18282	0.41053	0.079231	-0.14004	0.076384
0.228304	-0.03977	-0.33173	0.270535	-0.16079	-0.05676	0.175829	-0.16618	-0.20075
0.242401	-0.11743	-0.52182	0.410756	-0.10794	-0.15143	-0.1473	-0.1004	-0.04045
0.285805	0.030459	-0.22806	0.298599	0.068738	0.168444	-0.10167	0.253303	0.12007
0.503635	-0.00246	-0.35861	0.412893	-0.29979	0.051537	-0.07898	0.336087	0.144475
-0.06147	-0.18016	-0.06674	0.089254	-0.10166	-0.12829	-0.38566	-0.16778	-0.20107
0.3063	-0.21475	-0.2598	-0.25909	-0.23925	0.122478	0.262759	-0.25066	-0.08299
0.309867	-0.2735	-0.33182	0.158713	-0.12916	-0.0832	-0.01861	-0.12274	0.111746
0.508603	-0.16888	0.153677	0.075469	0.218667	-0.02861	0.160653	0.065544	0.291187
0.089646	-0.27716	0.098932	-0.0981	0.252026	0.14577	-0.09789	0.177371	-0.07012
-0.16616	-0.19283	0.172202	0.098849	0.591964	-0.58037	-0.46821	0.021215	0.141518
-0.07314	0.034912	0.06408	-0.34724	0.168907	-0.3136	-0.16241	-0.11068	0.053508
-0.02118	0.115882	0.125325	0.161352	0.025792	0.074927	0.1353	0.101616	0.079844
0.319385	-0.09223	-0.39249	0.356374	-0.15041	0.283012	0.217138	-0.03347	-0.22316
0.279478	0.160937	-0.49578	0.182443	-0.32559	0.319226	0.088049	-0.25344	-0.42808
0.637999	-0.16114	-0.45283	0.480463	-0.34997	0.370743	0.361677	0.271783	0.105206
0.29231	-0.06854	-0.32036	0.336287	-0.26747	0.339309	0.09968	-0.08023	-0.25947
0.183848	-0.41569	-0.29984	-0.21487	-0.46067	0.430875	-0.07124	-0.24159	-0.00232
0.141767	0.337805	-0.43925	-0.44417	0.093934	0.295574	0.481897	-0.46883	-0.14975
0.429866	-0.16337	0.181963	0.223124	-0.00466	0.355007	0.090864	0.096416	-0.06594
0.27077	-0.16813	-0.08662	0.178623	-0.22335	0.11571	0.12824	-0.25145	-0.08744
0.232597	-0.17201	0.020265	0.08033	-0.40034	0.678201	0.684904	0.006562	-0.03359
0.502567	-0.04782	-0.04168	-0.12321	-0.40139	0.36367	-0.07247	0.069953	0.232778
0.08764	0.297591	-0.23838	-0.23596	-0.05842	0.25851	-0.00668	0.003049	-0.25979
0.342802	-0.25592	-0.15092	0.37394	-0.22584	0.196023	0.261897	-0.0548	0.0517
0.356418	-0.18468	-0.03016	0.379501	-0.24242	0.192989	0.230896	0.01273	0.041018
0.220279	-0.28606	-0.07077	-0.09343	-0.14856	0.097132	0.413766	-0.26421	-0.11087
0.314202	-0.41694	-0.30412	0.01029	0.07583	0.010578	0.516982	0.147531	-0.24146
0.249501	0.227595	-0.08379	0.072543	0.009932	-0.02833	-0.19002	0.33821	0.07676
-0.3011	0.40547	-0.5081	0.066701	-0.54599	0.442545	0.483518	-0.25629	-0.70897
-0.00263	0.05508	-0.05938	-0.37214	-0.30147	0.257814	-0.08868	-0.03247	-0.09305
-0.45863	0.209348	-0.22545	0.182803	-0.40388	0.191998	0.152246	0.057634	-0.37852
0.001596	-0.5441	-0.30106	-0.15596	-0.17508	0.309718	0.179163	-0.61496	-0.36274
-0.33796	0.287442	0.059593	-0.05798	-0.31913	0.306875	0.156958	-0.21449	-0.32309
-0.20748	-0.31763	-0.13298	-0.10198	0.155531	0.063334	-0.36081	-0.00551	-0.44119
0.61594	0.35546	0.205475	-0.45256	-0.28812	0.141772	-0.50278	-0.04889	-0.07002
0.323352	0.066027	-0.25448	0.221184	0.229528	-0.091	0.004085	0.32699	0.065161
-0.24042	-0.50388	-0.2868	-0.2252	-0.45205	0.328273	0.116265	-0.52007	-0.44588
-0.18214	-0.35337	-0.26193	0.030413	-0.26542	0.238847	0.231731	-0.40162	-0.09179
0.155815	-0.32002	-0.19896	-0.26781	-0.09507	0.229442	0.169493	-0.44989	-0.08654
-0.25169	-0.59111	-0.32788	-0.44749	-0.34866	0.552942	0.061084	-0.40912	-0.29188
0.23232	0.097741	0.201128	0.101105	0.378495	-0.23472	-0.06692	0.344998	0.083957
0.338143	0.129433	0.30073	0.116382	0.182858	-0.2647	-0.01985	0.301742	0.260029
0.287459	0.203086	0.00846	0.434026	0.306909	-0.11155	-0.0879	0.317887	0.184549

0.183477	-0.08244	0.297682	0.033841	0.48213	-0.35163	-0.39845	0.456335	0.165507
0.087613	0.077903	0.310272	0.194456	0.441668	-0.32662	-0.3038	0.468482	0.299918
-0.0328	0.201039	0.079999	0.35614	0.163929	0.0424	0.138951	0.187781	-0.02087
0.145031	0.282731	-0.00714	0.323141	0.32215	-0.22734	-0.00144	0.315657	0.327632
0.256968	-0.16849	0.017762	-0.30175	-0.25154	-0.03006	0.092044	-0.10596	-0.01674
0.353844	0.053987	0.037922	0.149879	-0.12279	-0.05016	-0.16685	0.124711	0.004698
0.02408	-0.37587	0.046496	-0.41265	0.126007	0.003513	0.007442	0.041781	-0.14101
0.042106	0.155948	0.133601	-0.10683	-0.16773	0.076803	-0.06945	0.132584	0.103619
0.20115	0.273788	-0.01503	0.390494	0.04938	-0.07424	-0.04701	-0.04792	0.187942
-0.02023	-0.02175	0.181507	-0.06933	0.28345	-0.16652	-0.01158	0.152896	-0.00039
0.192464	-0.36597	-0.28333	0.107298	-0.31633	0.150361	0.180887	-0.27461	0.070026
0.156934	0.214878	0.078078	0.352646	0.16281	-0.33822	-0.28786	0.235334	0.117046
0.227246	-0.13993	0.230429	-0.07718	0.380536	-0.13129	-0.03721	0.338167	0.147163
0.412825	0.262239	0.013384	0.48775	0.357333	-0.01135	-0.16343	0.273934	-0.00187
0.117859	0.065378	0.272856	0.05073	0.26329	-0.2067	-0.15519	0.273282	0.251925
0.30648	0.313919	0.419653	0.154312	0.302775	-0.08601	-0.18358	0.474818	0.430779
-0.27651	0.072832	0.414361	-0.17841	-0.02469	-0.01907	-0.05231	-0.14894	0.189449
-0.09067	-0.05667	0.303681	-0.01771	0.346627	-0.28615	-0.22707	0.273525	0.29916
0.291584	0.196228	0.394658	0.056844	0.429761	-0.38472	-0.2655	0.497212	0.356705
0.101348	0.027809	0.24392	0.019251	0.24005	-0.31348	0.080063	0.263548	0.239817
0.14902	-0.0894	0.186171	0.080707	0.329643	-0.19031	-0.11889	0.378737	0.033163
0.334986	0.051166	0.440063	0.213766	0.428526	-0.42493	-0.18726	0.441145	0.341886
0.459218	0.017963	0.52424	0.275936	0.548313	-0.55358	-0.14256	0.449156	0.266205
0.009195	0.189454	0.368569	0.055855	0.491088	-0.58182	-0.37734	0.500466	0.347015
0.208409	-0.01889	0.396641	-0.0731	0.255624	-0.35597	-0.02203	0.434956	0.341265
0.228776	-0.15041	0.234647	-0.02547	0.104941	-0.06751	0.160527	0.112483	0.116696
0.361846	0.13189	0.126694	0.392141	0.315465	-0.04006	-0.08092	0.300144	0.2044
0.499137	0.214539	0.054925	0.262354	0.423978	-0.41846	-0.42581	0.462458	0.254904
-0.41713	0.299541	0.198581	-0.09688	0.277054	-0.32243	-0.56829	0.51919	0.282541
-0.03338	0.087879	0.369184	-0.0409	0.422637	-0.27136	-0.32043	0.388386	0.247128
0.17975	0.108152	0.512366	-0.18014	0.289804	-0.16858	-0.29708	0.535515	0.368098
-0.1598	-0.02151	0.10726	-0.00219	0.165238	-0.02292	-0.17336	0.047187	-0.09463
-0.17514	0.23397	-0.15251	0.085759	0.167821	0.113899	-0.24016	0.227673	-0.34478
0.072351	0.221465	0.273459	0.048573	0.404921	-0.25777	-0.17087	0.449231	0.154251
0.12197	0.139789	0.364618	0.007429	0.599555	-0.47612	-0.40544	0.528892	0.22242
-0.1073	-0.01963	0.206214	-0.12441	0.459906	-0.2897	-0.37152	0.456659	0.194415
0.03023	0.096006	0.380719	-0.15617	0.393441	-0.2751	-0.2069	0.497792	0.292684
0.033325	0.005938	0.31147	-0.06782	0.471012	-0.27282	-0.27877	0.453271	0.309915
0.188104	0.026943	0.294477	0.043064	0.307145	-0.1856	-0.26066	0.409259	0.320627
-0.15134	-0.0779	0.29126	0.092042	0.367807	-0.16768	-0.135	0.329138	0.2558
0.053761	0.044913	0.39995	0.033374	0.413744	-0.38186	-0.23598	0.40895	0.311156
-0.07952	-0.01547	0.217906	-0.06786	0.463215	-0.54048	-0.53357	0.501083	0.343691
0.219882	0.191216	0.123793	0.312778	0.231877	-0.01908	-0.19902	0.391175	0.068421
0.200237	0.162387	0.298758	0.141612	0.335519	-0.30552	-0.16454	0.436762	0.243788
0.328682	0.257166	0.170822	0.31289	0.271579	-0.01784	-0.14719	0.279578	0.218451
0.275952	0.235901	0.21829	0.28281	0.197038	0.052868	0.24135	0.134535	0.067592
0.101392	0.109532	0.320772	0.202474	0.362344	-0.26867	-0.06451	0.351352	0.24864
0.292978	0.109704	0.061987	0.272512	0.173717	-0.24319	0.011573	0.264676	0.044253
0.333122	0.031947	0.120317	0.233164	0.25667	0.045696	-0.20773	0.247285	0.07211
0.304445	0.045704	0.044194	0.166266	0.030835	-0.27787	-0.23081	0.143165	0.099847

0.207631	0.153489	0.236223	0.332109	0.304985	-0.23714	-0.13066	0.11796	0.11253
0.227588	0.166876	-0.02642	0.377967	0.253002	-0.11271	-0.02679	0.287249	0.132176
0.196794	0.349736	0.247976	0.100686	0.074782	-0.3157	-0.19101	0.405441	0.370805
-0.39557	0.14929	0.300842	-0.36067	0.359127	-0.44594	-0.60353	0.553485	0.267657
0.350395	0.2682	-0.20154	0.151849	-0.54505	0.252831	-0.41392	-0.31444	0.285041
0.320734	0.189681	0.072862	0.279266	0.025321	-0.38292	0.08392	0.37789	0.359588
0.462816	0.352283	0.217511	0.390672	0.281645	-0.12024	0.157767	0.142322	0.192558
0.143462	0.192034	-0.25517	0.354431	-0.07367	0.3108	-0.00808	-0.06787	-0.18436
0.052524	0.192503	-0.50092	0.562413	-0.25761	0.349411	-0.02815	-0.30211	-0.32215
0.105634	-0.09114	-0.01912	-0.07117	0.188255	0.154412	0.236031	0.308543	0.099413
0.002354	0.065077	-0.28844	0.360884	-0.25167	0.335525	0.016787	-0.25609	-0.11579
0.211208	0.445163	0.0508	0.257989	0.475814	-0.30304	-0.12006	0.552839	0.293219
-0.02785	0.131057	0.045042	-0.06627	0.296194	-0.09032	0.073176	0.242765	0.34036
0.032351	0.225091	0.217899	-0.14132	0.369648	-0.04756	-0.01811	0.416487	0.351861
-0.18209	-0.02951	0.435662	-0.07482	0.552135	-0.55483	-0.4566	0.610063	0.534757
0.144148	-0.00012	0.290902	0.01797	0.308335	-0.29497	-0.11404	0.332305	0.191643
0.142074	-0.02149	0.298787	0.047687	0.354992	-0.35622	-0.18328	0.368588	0.332206
0.231653	0.22156	0.30378	0.06461	0.14066	-0.32842	-0.26839	0.499336	0.305727
0.165602	-0.13266	0.27672	-0.04826	0.296103	-0.29284	-0.00971	0.382672	0.342087
0.187262	0.098756	0.293697	0.056269	0.237526	-0.38251	-0.08771	0.370434	0.478919
0.338509	0.034682	0.339717	0.193228	0.287024	-0.2361	-0.08209	0.460369	0.202222
0.336387	-0.02587	0.25345	0.045551	0.275008	-0.25101	-0.20134	0.407938	0.366188
0.002395	0.036951	0.302623	-0.13446	-0.02451	-0.17898	0.295043	0.392189	0.189773
-0.23194	-0.18648	0.337827	0.027393	0.297335	-0.35729	-0.33115	-0.00647	0.273048
0.320455	0.009417	0.255617	0.145449	0.371634	-0.33865	-0.08535	0.376935	0.352803
0.301273	-0.33152	0.199265	-0.08397	0.101295	-0.23069	-0.11139	0.302901	0.00457
0.163428	0.03241	0.261465	0.082959	0.277396	-0.28077	0.125335	0.449678	0.267614
-0.07779	0.032872	0.137419	0.200198	0.361769	-0.36308	-0.19043	0.415727	0.237295
0.274614	-0.02569	0.293272	-0.08241	0.223551	-0.25681	0.115936	0.371667	0.150763
0.048679	0.025008	0.116964	-0.01441	0.110568	-0.29001	0.08512	0.334163	0.283589
-0.08399	0.097614	0.473876	0.096239	0.469383	-0.63718	-0.57183	0.700223	0.345084
-0.33952	0.208645	0.24724	-0.09413	0.555925	-0.50409	-0.59355	0.555878	0.243236
-0.44518	0.019194	0.324413	-0.32624	0.536987	-0.54975	-0.64377	0.627902	0.31792
-0.20629	0.072595	0.214754	-0.27561	0.569923	-0.51908	-0.67415	0.689814	0.242856
-0.14337	-0.07143	0.424571	-0.01508	0.464186	-0.42575	-0.34795	0.482007	0.274838

TCGA-IB-7(	TCGA-IB-7{	TCGA-IB-7{	TCGA-IB-7{	TCGA-IB-7{	TCGA-IB-A!	TCGA-IB-A!	TCGA-IB-A(	TCGA-IB-A/
0.210878	0.231624	0.272191	0.268903	-0.20851	-0.30061	0.184411	0.215973	0.169839
-0.48638	-0.16751	0.102069	0.191766	-0.15135	0.297096	0.003292	-0.16451	0.21906
0.404054	0.34562	0.384009	0.149747	-0.02451	0.481815	-0.10937	-0.46382	-0.13853
-0.2712	0.009784	-0.00702	0.136078	-0.32697	0.187882	-0.00779	-0.26213	-0.29899
-0.3121	0.019285	0.30948	0.165359	-0.3967	0.122147	0.2335	-0.32122	-0.37581
0.034486	0.075393	-0.25655	-0.25112	-0.05956	-0.03111	-0.09393	-0.15512	-0.08098
-0.16564	-0.39766	0.133054	-0.17944	-0.35933	-0.17278	-0.47195	-0.11576	-0.31627
-0.05156	-0.13754	-0.2549	-0.24129	-0.2168	-0.04395	-0.05798	0.105074	0.056042
0.085382	0.08993	-0.17614	-0.09962	-0.00944	-0.15218	-0.04764	0.153759	0.201394
-0.13524	0.015575	-0.33563	-0.19955	-0.24095	0.235359	-0.04101	-0.03882	0.296816
-0.02852	-0.05745	-0.3431	-0.27972	-0.15151	-0.11072	-0.08309	0.060035	0.341528
-0.09828	-0.04919	-0.31454	-0.20538	-0.03643	-0.06378	0.117216	0.28398	0.462958
0.324388	-0.07053	0.092256	-0.06589	-0.15669	-0.18992	-0.28353	-0.27905	-0.26431
0.14468	0.24061	0.035254	0.061911	-0.28062	-0.02376	-0.18158	0.004064	-0.02328
-0.18224	0.158247	0.386562	0.112911	-0.20898	0.231565	0.218274	-0.15024	-0.4807
0.18699	0.373859	0.412721	0.184467	-0.21071	-0.09061	0.280136	-0.31731	-0.21209
-0.04868	0.194363	0.178539	0.321988	-0.0756	-0.25599	-0.30737	0.00766	-0.10274
-0.07143	0.355819	-0.22287	-0.08845	0.057567	0.221577	0.095424	0.098274	0.118899
0.015418	-0.16869	-0.36819	-0.26288	-0.07184	-0.20559	0.244488	-0.00931	0.354094
0.197828	0.082319	-0.34043	-0.06186	-0.58094	-0.46513	-0.01339	-0.12458	0.013098
0.226651	0.117808	0.442496	0.391799	-0.02215	-0.07388	0.130425	0.025647	-0.29984
-0.0439	0.102664	0.476895	0.416113	0.221107	0.226143	-0.03849	0.024341	-0.36015
0.188782	-0.06466	0.104058	-0.13332	-0.15891	0.075462	0.034306	-0.10448	0.081217
0.074551	0.04778	0.223116	0.038323	-0.03001	0.296342	0.072608	-0.33882	-0.30267
-0.25081	0.076794	-0.283	-0.11384	-0.15457	-0.01316	-0.00947	-0.18799	-0.14513
-0.19004	0.530514	0.387625	-0.01	0.313699	0.130753	0.440685	-0.45154	-0.17083
-0.18315	-0.38979	0.38679	0.134534	0.123211	0.355924	-0.47785	0.132084	-0.09957
-0.15988	0.063878	-0.0595	-0.13737	0.342683	0.267831	0.001696	-0.10531	0.306923
-0.16408	-0.10406	-0.41608	-0.30935	-0.02731	-0.02827	-0.23806	0.277515	0.398625
-0.20329	0.153115	0.214524	0.27883	0.115499	0.139067	0.253205	-0.08779	-0.4185
-0.15356	0.616331	-0.01183	0.161959	0.480914	0.394573	0.519998	-0.23974	-0.18976
-0.23196	0.461288	-0.44154	-0.24517	0.232607	0.374786	0.608586	0.017684	-0.03216
0.086914	0.274938	0.328772	0.287007	0.215494	0.171771	0.090058	-0.25074	-0.27228
0.190224	0.20612	0.446581	0.343877	0.305825	0.291905	-0.00899	-0.23141	-0.40866
0.099241	0.292163	0.306938	0.252066	0.329642	0.274993	0.18446	-0.25427	-0.17991
-0.38329	0.017143	0.052761	0.065204	-0.21983	-0.02077	-0.22968	-0.23313	-0.21358
-0.25557	0.175357	-0.43975	0.00458	-0.16896	-0.33817	0.1714	0.114132	0.340235
-0.52989	0.044495	-0.36474	0.162062	-0.39596	-0.27708	-0.10299	0.101772	-0.13944
0.028537	0.240238	-0.2527	0.317509	-0.03972	-0.2396	-0.39663	-0.5018	-0.18149
-0.34394	0.274393	0.052972	-0.08588	-0.31638	0.027889	-0.39413	-0.1819	-0.25519
-0.32884	0.226483	0.275529	0.244876	-0.10014	0.389987	-0.2021	-0.26205	-0.37456
0.042337	0.208362	0.044351	0.143507	0.051801	-0.23254	-0.39475	-0.42159	-0.056
-0.05787	0.162559	-0.21703	-0.09952	0.004658	-0.20912	-0.20172	0.079575	0.497703
0.257192	0.101575	-0.47759	-0.18126	-0.41975	-0.26379	-0.0707	0.402674	0.27019
-0.09473	0.620718	-0.32331	0.106957	0.278641	-0.24028	0.191099	-0.3445	0.38333
0.073085	0.178761	-0.09802	0.115064	-0.17888	-0.21762	-0.20083	-0.2125	-0.07327
-0.49931	-0.2684	-0.50646	-0.40801	-0.46292	-0.34205	-0.12681	-0.22239	-0.08835
-0.12272	0.146203	-0.10418	0.116297	-0.11596	-0.18993	0.055681	-0.04242	-0.01551
-0.25461	-0.05024	-0.2247	0.17864	-0.33083	-0.35722	-0.05933	0.107865	-0.22456

-0.08198	0.122127	-0.37334	-0.23763	-0.16523	-0.20247	-0.22142	0.023433	0.226406
0.141163	0.033427	-0.42055	-0.02565	-0.19577	-0.199	-0.27997	0.310024	0.537751
0.128949	-0.00184	-0.03618	0.297385	-0.05575	-0.19699	-0.12976	0.104346	0.198093
-0.03612	0.060052	-0.36188	-0.24797	-0.13971	-0.24439	-0.31443	0.149309	0.523653
0.14317	-0.00444	0.087326	0.107383	0.145526	-0.33182	0.132861	0.112429	0.433989
-0.09375	0.131964	-0.29685	-0.23342	-0.33748	-0.10921	-0.23702	-0.10719	0.155709
-0.29659	-0.08816	-0.3742	-0.33953	-0.24331	-0.28926	-0.18064	-0.02988	0.270258
-0.39509	-0.15249	-0.33179	-0.18112	-0.18433	-0.16293	-0.29588	0.056351	0.147983
-0.52401	-0.16422	-0.42968	-0.38962	-0.28735	-0.14524	-0.24701	-0.05061	0.093567
0.017894	0.400864	-0.29853	-0.17005	0.138511	0.154209	-0.05259	-0.13368	0.263932
-0.07739	0.095936	-0.08504	-0.0088	0.257693	-0.23737	0.134728	-0.20557	0.328466
-0.53241	-0.07019	-0.39688	-0.54137	-0.09952	0.433575	-0.20809	0.33301	0.434048
0.032028	-0.11888	-0.40496	-0.08529	-0.18078	-0.35934	-0.29939	0.173248	0.496205
-0.30513	0.150084	-0.44952	-0.08877	-0.20299	-0.11571	-0.16265	-0.24464	-0.06422
0.064305	0.151519	0.159655	0.33875	0.129293	-0.03395	-0.30329	-0.18458	-0.1914
-0.16598	0.314877	0.132231	0.26628	-0.13115	-0.01278	0.174413	-0.51782	-0.21051
0.151362	-0.00172	0.494457	0.466159	-0.18151	0.557118	0.399546	-0.25559	-0.37383
0.091067	-0.12239	0.392192	0.338657	-0.1123	0.33781	-0.28203	-0.31806	-0.13666
-0.15217	0.1775	0.270972	-0.03516	0.150755	0.05114	0.137407	0.043882	0.226661
-0.2243	0.10511	-0.23661	-0.15032	-0.20761	-0.308	-0.14175	-0.03672	0.162296
-0.37664	-0.26943	-0.41129	-0.25699	-0.39929	-0.50679	0.102426	0.25691	0.079019
0.15309	0.243406	0.003935	-0.05533	0.001935	-0.37545	0.052232	0.06079	0.516626
-0.0957	0.396964	-0.38909	-0.20516	-0.04986	-0.12627	-0.25882	-0.22975	0.355242
-0.20583	-0.07286	-0.29147	0.217346	-0.35853	-0.13875	-0.12114	0.341923	-0.03041
-0.34561	-0.10303	-0.28147	-0.00655	-0.39915	-0.02369	0.256363	0.215228	-0.33477
0.084541	0.067669	-0.15518	0.005454	-0.04127	-0.09393	-0.24042	-0.37187	-0.17279
-0.13993	-0.12147	-0.16439	0.17725	-0.3846	-0.2648	-0.33401	-0.35904	-0.23748
-0.15887	0.11156	-0.59208	-0.18527	-0.32294	-0.26965	0.351763	-0.11554	0.17091
-0.18874	0.150737	-0.06878	-0.18478	0.012463	0.151605	-0.25442	0.171009	0.370931
-0.05056	0.200662	-0.0502	0.232215	-0.06667	0.156449	0.003155	-0.09404	0.018744
0.008009	0.132501	-0.3438	-0.06445	-0.03653	-0.12998	-0.3545	-0.17316	0.056165
0.025121	0.166707	-0.30913	-0.14459	0.02675	-0.09412	-0.31484	-0.19566	0.209132
-0.12603	-0.0753	-0.10656	-0.03228	-0.27879	-0.19931	-0.31819	-0.25566	-0.18318
-0.16355	0.093111	0.006077	0.301003	-0.11531	-0.36968	-0.34145	0.160824	-0.18122
0.235622	0.064861	-0.19803	-0.14479	0.202502	0.171989	0.108722	-0.10347	-0.10474
-0.54444	0.303933	-0.57721	-0.26673	-0.13858	-0.31824	0.520259	0.664321	0.618573
-0.15922	-0.09479	0.116298	0.15329	0.039482	-0.41169	-0.02077	-0.05102	-0.20024
0.194217	0.04731	-0.33125	0.01369	-0.28146	-0.5179	0.365626	0.213865	0.069255
-0.51185	-0.19787	-0.04962	0.408783	-0.40989	-0.42776	-0.06795	0.087967	-0.48253
-0.32384	-0.23335	-0.28527	-0.04756	0.100062	-0.22727	0.062819	0.118981	0.172948
-0.47623	0.18726	-0.42649	0.188382	-0.2966	-0.34875	0.218136	-0.2429	-0.467
0.272599	0.568412	0.320481	0.319035	0.239856	-0.60426	-0.19256	0.394264	0.634727
-0.08021	0.291884	-0.21561	-0.14245	0.230501	-0.15395	0.23238	-0.27859	-0.08198
-0.3743	-0.40557	-0.348	-0.12325	-0.57008	-0.10407	-0.0698	0.261838	-0.25596
-0.40678	-0.04129	0.160136	0.21016	-0.1811	-0.38955	-0.28322	0.163517	-0.27638
-0.50926	0.07478	0.085163	0.387854	-0.04075	-0.47414	-0.15413	0.051564	-0.43212
-0.40689	-0.25985	0.129305	0.319284	-0.42133	-0.33238	0.149824	-0.05735	-0.61697
0.172146	0.216834	0.154219	0.133074	0.250836	0.180258	0.147089	-0.05626	-0.11659
0.302455	0.265428	0.29399	0.238735	0.285391	0.221221	0.059894	0.136899	0.06806
0.02967	0.312907	0.02565	0.082684	0.312262	0.216855	0.221546	-0.24342	-0.058

-0.16646	0.384611	-0.14184	0.043564	0.277875	0.316007	0.312117	-0.10391	-0.12968
-0.05216	0.385665	-0.08776	0.046238	0.382693	0.225642	0.423165	-0.19035	-0.10914
-0.04915	0.21217	0.204135	-0.0915	0.179987	0.2383	0.249715	-0.08113	0.10264
0.089994	0.362677	0.105607	0.022306	0.301288	0.225006	0.186421	-0.17133	0.116372
-0.21521	-0.10917	0.188806	0.364507	0.078128	-0.16843	0.057635	0.013165	-0.47114
-0.11447	0.080301	0.139454	0.20924	0.322063	-0.06767	0.057565	-0.13	-0.30186
-0.3389	-0.07837	0.063709	0.136802	-0.1419	0.10336	-0.07401	0.013854	-0.3448
0.248023	-0.0178	0.197067	0.175757	0.186653	-0.26742	0.173865	0.136144	-0.11855
0.039522	-0.07827	0.086736	-0.10278	0.069694	0.180963	-0.25281	-0.00935	0.267395
0.260206	0.060953	0.253316	0.028436	0.058989	0.104826	0.049232	-0.12861	-0.1613
0.008781	-0.04544	-0.27888	-0.22669	-0.27644	-0.38411	-0.3063	-0.00072	0.185344
0.219302	0.153409	0.124427	0.057417	0.230349	0.097499	0.182614	0.103555	0.181208
-0.15093	0.238159	-0.14371	-0.05339	0.203043	0.013524	0.316496	-0.00435	-0.18527
0.079692	0.265179	-0.00896	0.017553	0.37176	0.215517	0.297065	-0.12729	0.190545
0.249936	0.089476	0.276302	0.204698	0.228572	0.128243	0.105256	0.033401	-0.19155
0.359472	0.320237	0.27928	0.296342	0.38327	0.265857	0.149949	0.120049	-0.23748
0.180111	-0.29224	0.171306	-0.16183	-0.19348	0.39512	0.019253	0.291334	0.213541
0.084118	0.206775	0.30799	0.073183	0.113704	0.361905	-0.00929	-0.08661	-0.23414
0.435019	0.392367	0.438089	0.450423	0.429615	0.315566	0.06256	-0.02536	-0.17235
0.196851	0.098661	0.410862	0.256332	0.228742	0.342367	0.005319	-0.03643	-0.32854
0.062115	0.199425	0.144133	0.038936	0.138255	0.184787	0.299466	0.082154	-0.0344
0.351035	0.260589	0.523346	0.428956	0.34888	0.306357	0.198091	-0.03182	-0.2266
0.288166	0.24787	0.616372	0.505614	0.297529	0.437392	0.320599	-0.27564	-0.3339
0.048356	0.455776	0.062808	0.048294	0.421667	0.476784	0.492529	-0.2934	-0.17141
0.38546	0.321262	0.475753	0.374227	0.387945	0.255713	0.000417	0.040693	-0.28936
0.125838	-0.00848	0.215645	0.212018	0.012891	0.0902	-0.14129	-0.03702	-0.16694
0.172055	0.254477	0.209999	0.161203	0.304527	0.139157	-0.02812	-0.02548	-0.05795
-0.24224	0.413483	-0.07805	0.009296	0.337115	0.403926	0.079495	-0.03693	-0.03424
0.046836	0.212286	-0.19357	-0.09581	0.264956	0.279246	0.465787	-0.2981	-0.27054
-0.05114	0.232372	-0.01589	0.056464	0.260623	0.194861	0.309429	-0.11628	-0.25562
-0.06509	0.25422	-0.15058	0.253187	0.2818	-0.1117	0.342929	-0.16413	-0.28416
-0.08599	-0.10522	-0.22246	-0.15421	0.029505	0.120282	0.322235	-0.0059	-0.17912
-0.17926	0.194229	-0.3963	-0.23659	-0.09012	0.005537	0.442515	0.223317	-0.076
0.065781	0.271362	0.090831	0.007501	0.405741	0.297896	0.322275	0.017044	-0.05478
-0.26815	0.447064	0.136183	0.130345	0.349355	0.403662	0.506335	-0.09371	-0.23323
-0.04222	0.341839	0.018617	0.070587	0.313712	0.305296	0.409506	-0.1574	-0.22286
0.042095	0.296398	0.144892	0.233935	0.387644	0.227407	0.361244	0.034362	-0.16215
-0.02894	0.400376	0.190542	0.146478	0.288463	0.421631	0.492816	0.055281	-0.29921
0.080054	0.304111	0.160298	0.063886	0.309593	0.133074	0.26957	0.027414	0.042265
-0.02231	0.361173	0.2523	0.220665	0.096679	0.262213	0.219859	-0.16705	-0.12551
-0.00874	0.340606	0.301998	0.216001	0.236988	0.347964	0.415194	-0.0785	-0.25097
-0.12265	0.540917	-0.22448	-0.08921	0.436321	0.475087	0.483876	-0.01911	-0.00238
0.320436	0.29958	0.119965	0.194971	0.384026	0.188898	0.173235	-0.1413	-0.18421
0.137785	0.272329	0.19276	0.104748	0.325097	0.139156	0.347249	0.038516	-0.14185
0.274813	0.25863	0.095866	0.192748	0.347712	0.04143	-0.00021	-0.03803	0.07001
0.176446	0.103078	0.059002	0.203602	0.204836	0.231034	0.275416	0.090322	0.243931
0.194208	0.257149	0.393733	0.319943	0.252503	0.314894	0.196156	-0.07461	-0.19632
0.081942	0.187013	0.039386	-0.01851	0.20937	-0.05829	0.084916	0.10353	-0.00746
0.198022	0.148464	0.034207	-0.12714	0.246022	0.214872	0.123896	-0.0449	0.057858
-0.08169	0.163082	0.180302	0.193093	0.337778	0.115614	0.048578	-0.12461	-0.19853

0.03946	0.117136	0.231082	0.203555	0.286258	0.298416	0.06264	-0.06798	-0.01854
0.103119	-0.02921	-0.10747	-0.07889	0.14539	0.108013	0.126602	0.043927	0.013898
0.153092	0.307435	0.200981	0.049382	0.453055	0.151757	-0.08383	-0.15826	0.011495
-0.13682	0.439128	-0.27158	-0.16137	0.45503	0.392406	0.46134	-0.34958	-0.25588
0.169985	-0.06551	-0.49585	-0.48457	0.286778	0.057653	-0.57613	0.286265	0.715531
0.1609	0.273208	0.236257	-0.04174	0.307138	0.242787	0.107161	0.188269	0.068651
0.147515	0.195289	0.201153	0.026818	0.423471	0.317651	-0.02857	-0.02874	-0.01248
-0.25065	0.003158	-0.30542	-0.22719	-0.22588	-0.18678	0.114915	-0.21436	-0.04699
-0.48575	-0.21653	-0.54243	-0.40223	-0.45337	-0.32846	0.03522	-0.24101	0.086881
0.102174	0.165668	-0.16799	-0.00915	0.098228	0.160929	0.218305	-0.31275	-0.27175
-0.24221	-0.15848	-0.39492	-0.29016	-0.34588	-0.32623	0.051262	-0.0469	0.057489
-0.04152	0.484609	-0.00116	0.141898	0.434098	0.211293	0.410848	-0.32677	0.017679
0.040096	0.161474	-0.02978	-0.03268	0.003781	0.123402	-0.091	-0.11581	-0.06962
0.15229	0.194131	-0.01255	0.040507	0.21317	0.037081	0.166614	-0.08098	-0.40753
-0.08601	0.458881	0.002336	0.118687	0.34876	0.413428	0.581979	-0.27916	-0.3617
0.141011	0.171111	0.293511	0.267771	0.201571	0.268751	0.160128	0.073733	-0.14758
0.170062	0.237855	0.344364	0.211933	0.293306	0.098327	0.161263	0.202512	-0.1582
0.201407	0.27505	0.362663	0.233361	0.417324	0.010647	0.195043	0.172974	0.002717
0.200947	0.122016	0.333146	0.272721	0.194186	0.136303	0.058636	0.245099	-0.18935
0.39087	0.236493	0.386309	0.130979	0.263962	0.129378	0.318887	0.160083	-0.00898
0.157702	0.178106	0.398551	0.339815	0.375283	0.114359	0.087401	0.124273	-0.10584
0.250802	0.139469	0.29251	0.281872	0.223303	0.111025	0.170677	0.231721	-0.10709
0.440901	0.204911	0.380746	0.277531	0.307466	0.192029	0.138809	0.278978	0.019285
0.080852	-0.00504	0.290942	0.186442	-0.13963	0.433292	0.134947	0.013159	-0.10584
0.247807	0.150604	0.377252	0.357526	0.253467	0.275639	0.012263	0.040214	-0.04857
0.064499	-0.0425	0.13036	0.151059	0.056012	-0.0389	0.032042	0.29214	-0.02502
0.21531	0.162289	0.272542	0.21101	0.271159	0.094066	0.238591	0.288009	-0.13876
0.281671	0.263953	0.216939	-0.00216	0.230781	0.251348	0.319838	0.10502	-0.05855
0.053341	0.041631	0.290591	0.281738	0.146557	0.013315	0.183761	0.161839	-0.19424
0.13511	0.195513	0.227589	0.110546	0.183647	0.053337	0.205909	0.271467	0.065779
-0.20808	0.537697	-0.01639	-0.06084	0.528917	0.35078	0.559236	-0.16573	-0.0878
-0.1339	0.427954	-0.23714	-0.08944	0.359249	0.408143	0.516273	-0.21227	-0.15812
-0.20485	0.432572	-0.33703	-0.21134	0.457304	0.436057	0.568401	-0.39896	-0.2458
-0.05558	0.506736	-0.35083	-0.0818	0.356879	0.399518	0.554308	-0.47731	-0.27108
0.098088	0.359795	0.298895	0.314866	0.334075	0.427692	0.449187	-0.39843	-0.33439

TCGA-IB-A	TCGA-IB-A	TCGA-IB-A	TCGA-IB-A	TCGA-IB-A	TCGA-IB-A	TCGA-LB-A	TCGA-Q3-A	TCGA-Q3-A
0.066943	-0.13124	-0.24758	0.129843	-0.18501	-0.13803	-0.02185	-0.29549	-0.45717
0.346661	0.085556	-0.36967	0.660151	0.608395	0.126211	-0.32879	-0.4308	-0.36424
0.003096	-0.11101	-0.31695	-0.15187	-0.34319	-0.0565	-0.30049	-0.35483	-0.04381
-0.19533	-0.08574	0.01848	0.561831	0.172945	0.453304	-0.31433	-0.18161	-0.18659
-0.16224	0.249644	0.059311	0.34871	-0.04941	0.146162	-0.20523	-0.20362	-0.13943
-0.23534	-0.17947	-0.08871	-0.12962	0.112023	-0.14448	-0.01241	0.115811	0.180622
-0.47569	-0.04962	-0.53376	-0.36782	-0.12009	-0.51197	-0.00147	-0.5925	-0.47534
-0.15537	-0.25212	-0.14642	-0.19112	0.01377	-0.18766	0.103842	-0.12083	0.250675
-0.11243	-0.27318	-0.18091	-0.24715	0.077778	-0.188	0.361918	-0.28473	0.31681
0.046093	-0.01681	-0.02142	0.012696	0.347251	-0.05747	0.208918	0.181737	0.094675
-0.17634	-0.23877	-0.0898	-0.23235	0.132422	-0.28664	0.271724	-0.05366	0.358317
0.02599	-0.41862	-0.10522	-0.25046	0.135906	-0.16901	0.383962	0.042438	0.357558
-0.32802	-0.18465	-0.43919	0.039508	0.227462	-0.13437	-0.14945	-0.43549	-0.2931
-0.3468	-0.19012	-0.31379	-0.1326	-0.07087	-0.3301	0.116625	-0.19804	0.151899
-0.03265	-0.34236	-0.06516	0.27068	0.103844	-0.06172	0.228919	-0.13641	-0.16799
0.170091	-0.21758	0.088529	0.465104	-0.05379	0.337606	-0.55792	-0.07139	-0.4512
-0.25793	0.059889	-0.24651	0.121653	-0.2594	-0.10545	-0.3148	0.051991	-0.04123
-0.09762	0.08668	-0.08645	0.110105	-0.2346	-0.00959	0.309745	0.0045	-0.36972
-0.22249	0.111764	-0.26765	-0.13701	0.082113	-0.13029	-0.04158	-0.21611	-0.34506
-0.39885	-0.11821	-0.42909	-0.52249	-0.57722	-0.59735	0.199589	-0.38717	-0.11354
-0.02918	-0.07187	-0.12761	-0.17522	-0.49973	0.184545	-0.09336	0.120213	-0.01048
0.464604	-0.08477	-0.06232	-0.59802	-0.64934	-0.56519	0.405534	0.202361	-0.27427
-0.14163	0.115922	-0.12684	0.142957	0.102292	-0.02814	-0.30803	-0.16346	-0.18838
0.071085	-0.00247	-0.25914	0.5453	0.431093	0.266153	-0.3545	-0.1674	-0.24717
-0.05797	0.122624	-0.09656	0.071567	-0.14253	-0.11864	0.016587	0.211502	0.343009
0.118261	0.26405	0.487174	0.566351	0.319485	0.418057	-0.19507	-0.06268	-0.04764
-0.33542	-0.00149	0.020959	-0.08133	0.301486	0.051655	0.040416	-0.3216	0.304806
0.205419	0.232688	0.300016	0.002073	0.233973	0.124132	0.206437	0.125375	0.283982
-0.49819	-0.19998	-0.43576	-0.31813	0.528978	-0.37065	-0.14278	-0.26364	-0.07185
0.258866	-0.07321	0.191605	0.147612	-0.06857	0.088506	-0.25256	0.018913	-0.23234
0.529042	-0.24821	0.55409	0.607228	0.572972	0.479692	-0.14365	0.506741	-0.32293
0.765256	-0.2287	0.711197	0.526449	0.485057	0.189999	0.023648	0.739036	-0.27474
0.135971	0.035336	0.108393	0.2155	-0.09308	0.188397	-0.3019	-0.16188	0.125167
0.227894	0.011052	0.010145	-0.00236	-0.07298	0.096268	-0.34149	-0.22258	-0.03789
0.253739	0.067691	0.227816	0.235185	0.017821	0.244005	-0.3473	-0.12598	0.039131
-0.23989	0.222216	-0.39019	-0.00885	0.124329	-0.32247	-0.02597	-0.28732	0.129126
-0.36094	-0.46317	-0.58542	-0.10209	0.064308	-0.47624	0.221807	-0.39866	-0.53535
-0.08736	0.345908	-0.23453	0.115877	0.130441	-0.42175	0.273739	0.0255	0.122102
-0.31369	0.277669	-0.43052	-0.14641	0.029325	-0.05045	-0.09515	-0.31753	0.212776
-0.48277	0.15381	-0.39448	-0.11324	0.081728	-0.45663	-0.2606	-0.29866	0.229349
-0.21894	0.39862	-0.23511	0.388626	0.255723	0.30029	-0.30827	-0.32087	-0.03468
-0.39415	0.191447	-0.39849	-0.29309	0.193881	-0.28635	0.05442	-0.39838	0.254019
-0.3273	-0.4348	-0.37413	-0.06112	0.313886	-0.20283	0.133231	-0.37719	-0.01622
-0.48149	-0.23953	-0.41427	-0.42199	0.207976	-0.60388	0.236336	-0.3091	-0.3785
-0.032	0.073876	-0.07132	0.398605	0.59369	0.192847	0.437008	0.063413	-0.33025
-0.24602	0.180126	-0.34144	-0.16028	0.140527	-0.25023	0.176712	-0.23342	0.101965
-0.34646	0.018221	-0.35051	0.079998	-0.20063	-0.35288	0.221799	0.21379	0.165157
-0.07484	0.096965	-0.12588	-0.02498	-0.20366	-0.146	-0.06647	-0.08072	-0.00911
-0.20073	0.067362	-0.30284	-0.20048	-0.46303	-0.26895	0.151855	-0.02762	0.018919

-0.36574	-0.02458	-0.3856	-0.2656	-0.04514	-0.32111	0.334145	-0.30567	0.051399
-0.41596	0.040884	-0.32611	-0.22607	0.477339	-0.26481	-0.14273	-0.17501	-0.14901
-0.18708	0.056094	-0.34597	-0.04337	0.246689	-0.23534	-0.14944	-0.1084	0.068537
-0.45173	-0.20976	-0.55008	-0.30247	0.477978	-0.39394	0.366029	-0.46105	-0.2198
0.046596	-0.2205	-0.12509	-0.29617	0.169204	-0.09538	0.18983	-0.27769	-0.32116
-0.43159	-0.00547	-0.44455	-0.20497	0.005624	-0.24114	0.120538	-0.40634	-0.05179
-0.43689	-0.21422	-0.34914	-0.3451	-0.27429	-0.33324	0.407876	-0.14249	0.289218
-0.25123	0.081304	-0.13193	-0.14499	0.109576	-0.11952	0.313745	0.113575	0.219714
-0.40892	0.042035	-0.17808	-0.04995	0.087692	-0.26193	0.25111	0.05709	0.170561
0.145456	-0.16022	0.048799	0.047467	0.258287	-0.05044	-0.08713	0.192641	-0.08605
-0.46349	-0.36174	-0.39863	-0.14623	0.409078	-0.14549	0.34351	-0.18039	0.064088
-0.36331	0.473297	-0.14564	-0.13524	0.517023	-0.01778	0.064349	-0.27332	0.006641
-0.37593	0.044858	-0.34287	-0.30647	0.136437	-0.42532	0.108886	-0.30845	0.074479
-0.32488	0.147017	-0.28452	-0.01641	0.331684	-0.36553	0.514651	-0.26196	0.016422
-0.10927	0.406441	-0.21535	-0.02929	0.026184	0.046751	-0.08484	-0.24982	0.061989
-0.38828	0.046589	-0.29616	0.321191	0.213027	0.247434	-0.20873	-0.34599	-0.17957
0.340769	0.63429	0.43581	0.555042	0.30969	0.692759	-0.43961	0.448102	0.043395
0.068657	0.08488	0.055772	0.439407	0.2302	0.391532	-0.18285	-0.02194	0.105624
0.124532	-0.25145	0.130418	-0.08712	0.146152	-0.10689	-0.06561	-0.07298	-0.00701
-0.45601	0.045584	-0.43651	-0.23396	0.081029	-0.1646	0.018401	-0.0511	-0.14153
-0.27964	0.369351	-0.34632	0.125473	-0.262	-0.17754	0.211912	-0.13134	-0.08567
-0.49731	-0.43738	-0.51624	-0.40996	0.34314	-0.16743	0.100674	-0.33675	-0.22496
-0.38582	-0.08228	-0.48835	-0.2622	0.426388	-0.26384	0.090376	-0.30666	-0.04392
-0.28172	-0.02503	-0.29731	-0.22942	-0.49927	-0.25508	0.093538	-0.20544	0.035083
-0.19779	0.441195	-0.38872	0.087801	-0.32134	-0.2783	0.548906	0.187022	-0.27159
-0.21242	0.198305	-0.29588	-0.20842	0.048109	-0.279	0.09997	-0.13573	0.288905
-0.3978	0.38166	-0.46094	0.060317	-0.13334	-0.28924	0.136873	-0.20119	0.068781
-0.57321	-0.12937	-0.34822	-0.40518	-0.20976	-0.601	0.289362	-0.33473	-0.35953
-0.41011	-0.38943	-0.37638	-0.32737	0.065208	-0.19159	0.230064	-0.32582	0.190992
-0.28277	-0.10783	-0.28884	-0.08015	-0.37207	-0.04234	-0.22484	-0.09741	0.193582
-0.19796	0.228599	-0.29682	-0.2162	0.294021	-0.23225	0.410494	-0.15266	0.297032
-0.2849	0.123118	-0.28609	-0.27135	0.293436	-0.21485	0.306098	-0.19377	0.290642
-0.43422	0.267109	-0.35334	0.043077	-0.21027	-0.30631	0.08617	-0.25627	0.246534
-0.58066	-0.08533	-0.50294	-0.04408	-0.27973	-0.50811	-0.46579	-0.25607	0.05376
0.243593	-0.08595	0.137679	0.090836	0.178686	0.084997	-0.20569	-0.1071	-0.02796
0.635018	-0.17221	0.660011	-0.21133	-0.20313	0.322964	-0.20231	0.784132	0.518303
0.186654	-0.21553	0.137413	-0.08583	-0.41007	-0.05492	-0.01039	0.323085	0.046622
-0.0987	-0.15777	0.130745	-0.33165	-0.45567	0.190115	0.112586	0.307083	-0.08031
0.46506	-0.0177	-0.26915	-0.23839	-0.59453	-0.62097	0.379845	0.329221	-0.03361
0.406901	-0.26678	0.392395	-0.20266	-0.37866	-0.16431	0.181364	0.437093	0.11606
0.057215	0.085746	-0.43357	0.173769	-0.51251	-0.48245	0.047983	0.050757	-0.13388
-0.23933	-0.11063	-0.43406	-0.05944	-0.26334	-0.08313	-0.20877	-0.18315	-0.12861
-0.31988	0.007712	-0.16861	0.057538	0.023143	-0.07054	-0.1056	0.084226	0.078749
0.280086	-0.33228	-0.17333	-0.33202	-0.54748	-0.63474	0.289629	0.065171	0.347711
-0.00388	0.161683	-0.32351	-0.33347	-0.51143	-0.30725	-0.03324	-0.01145	-0.0192
0.426658	0.045871	-0.39807	-0.39454	-0.53432	-0.5533	0.313143	0.111745	-0.01227
0.272136	0.131137	-0.20871	-0.22166	-0.65589	-0.4838	0.410829	0.211272	0.271738
0.197441	-0.16083	0.24783	0.18604	0.171891	0.239376	-0.06948	0.074903	-0.0981
0.138969	-0.22873	0.104143	-0.10792	0.112662	0.051138	-0.08469	-0.00899	-0.3656
0.229389	-0.18209	0.26359	0.138824	0.103658	0.216	-0.17628	0.118298	-0.071

0.282309	-0.18367	0.428768	0.408261	0.362051	0.189484	-0.12088	0.401523	-0.04101
0.303859	-0.25931	0.402981	0.400622	0.318365	0.298864	-0.1237	0.319389	-0.2417
0.275896	-0.2877	0.206907	-0.1221	0.065755	-0.03028	-0.08922	0.141141	-0.01176
0.183789	0.079248	0.294571	0.289503	0.250014	0.202451	-0.1476	0.236747	-0.04103
0.279257	-0.06545	-0.17148	-0.28913	-0.483	-0.36764	0.19907	0.089615	-0.22916
0.237908	-0.03808	-0.13791	-0.20248	-0.29722	-0.19188	0.011732	-0.18284	-0.22299
0.117599	0.110753	-0.04846	0.051276	-0.31592	-0.10259	-0.16595	0.206101	0.217963
0.239022	-0.05102	-0.20001	-0.29234	-0.35048	0.021683	0.096563	-0.01256	-0.21305
-0.20112	-0.11477	-0.28833	0.011645	0.010251	0.251037	-0.22345	-0.16893	-0.15255
0.027323	-0.21955	0.06225	0.031148	0.086161	-0.03838	-0.12603	-0.14994	-0.13628
-0.41476	-0.04045	-0.5363	-0.31138	-0.25583	-0.40778	0.308165	-0.34714	0.104082
0.121766	-0.04179	0.138045	0.188993	0.13468	0.246144	0.051804	0.162785	-0.3726
0.193887	-0.28584	0.286171	0.262651	0.037007	-0.02706	-0.11301	0.1044	-0.17605
0.309645	-0.1102	0.261938	0.274624	0.317	0.307745	-0.17352	-0.01286	0.042482
0.187738	0.036911	0.083153	-0.10931	-0.05854	0.128644	0.036325	-0.06454	-0.19928
0.251876	-0.00829	0.126645	0.046443	0.486619	0.116949	-0.34322	-0.23039	-0.25818
0.278428	0.062718	0.085258	-0.01037	0.260181	-0.1784	-0.06906	-0.23879	-0.04494
0.16587	0.308011	0.197083	0.015953	0.06845	0.126929	0.101741	-0.0416	0.005847
0.155688	-0.08382	0.210687	0.050211	0.237763	0.31183	-0.20034	-0.11178	-0.37805
0.155832	0.175508	-0.01121	-0.07092	0.055339	0.036452	-0.00073	-0.21104	-0.27805
0.281029	-0.32049	0.198672	0.169947	0.128953	0.126551	-0.02933	0.057463	-0.22962
0.319093	0.171009	0.157749	0.236484	0.124341	0.235222	-0.34815	-0.17721	-0.34579
0.365003	0.336562	0.183106	0.433172	0.213774	0.228128	-0.4367	-0.17992	-0.36289
0.509278	-0.10766	0.325937	0.414881	0.517024	0.265179	-0.23232	0.286037	-0.38514
0.067582	0.037849	-0.11136	-0.19592	0.106721	0.045631	-0.34265	-0.28566	-0.26924
-0.13883	-0.16407	-0.29858	-0.15695	0.140679	-0.16031	-0.10719	-0.41005	0.030167
0.165381	-0.00053	0.038666	0.143354	0.222178	0.230355	-0.19039	-0.20703	-0.20817
-0.08579	0.470371	0.193423	0.382145	0.506118	0.382713	-0.40428	-0.03256	-0.24842
0.492223	-0.31929	0.438889	0.551835	0.495558	0.126903	0.132462	0.372387	-0.30633
0.292895	-0.35216	0.314597	0.403773	0.257106	0.298649	-0.29918	0.341787	-0.29655
0.104304	-0.21922	0.32179	0.291638	0.064683	-0.00195	0.109179	0.249115	-0.06801
0.371302	0.066271	0.303273	0.116895	0.092976	-0.09318	-0.19423	0.389792	-0.18409
0.275478	0.015171	0.434555	0.287934	-0.1298	0.09527	0.026622	0.510026	-0.10819
0.354157	-0.18731	0.447268	0.312138	0.225691	0.184393	-0.20062	0.411159	-0.1522
0.503725	-0.23647	0.52401	0.524514	0.428939	0.36426	-0.29437	0.501131	-0.20543
0.467225	-0.27868	0.376494	0.453853	0.340412	0.174821	-0.2225	0.376411	-0.23441
0.519395	-0.35093	0.393128	0.226608	0.2446	0.208655	-0.22687	0.345984	-0.33655
0.578344	-0.3016	0.468696	0.431155	0.224436	0.314107	-0.2372	0.452593	-0.28846
0.377907	-0.3458	0.256287	0.173346	0.083679	0.078626	-0.06446	0.25621	-0.20223
0.364214	-0.24014	0.261825	0.264473	0.166701	0.09066	-0.13085	0.159269	-0.17631
0.380739	-0.09478	0.181601	0.274356	0.372959	0.102164	-0.25031	0.116334	-0.16921
0.631251	-0.34092	0.72505	0.616111	0.613424	0.393188	-0.12268	0.699267	-0.21601
0.417601	-0.2719	0.244701	-0.01497	0.084128	0.116742	-0.10701	0.095812	-0.23048
0.363301	-0.24569	0.230089	0.260247	0.181667	0.272525	-0.2484	0.117201	-0.28314
0.175707	-0.18906	-0.02574	-0.02143	0.154516	0.051482	-0.10633	-0.14739	-0.19508
0.227489	0.093395	0.281497	0.282392	0.185956	0.212153	0.154653	0.206004	0.261436
0.31007	-0.00914	0.152301	0.178191	0.119301	0.222647	-0.20386	-0.07141	-0.24273
0.069308	-0.0372	0.082631	0.081489	0.131175	0.034757	-0.04319	0.044029	-0.13194
0.235484	-0.29452	0.173309	0.016534	0.174361	0.067742	0.025888	-0.12465	-0.00654
0.248197	0.10745	-0.15817	0.135848	-0.03844	-0.04932	-0.09684	-0.15464	-0.34289

0.252251	0.134566	0.142816	0.175113	0.243428	0.250453	0.114957	-0.03252	-0.09435
-0.18861	-0.1327	0.060694	0.162996	0.20773	-0.0181	-0.02169	-0.17194	-0.06761
-0.0389	0.148795	-0.04862	0.209082	0.181834	0.006432	-0.23604	-0.11289	-0.28598
0.561932	-0.29733	0.541777	0.451015	0.459229	0.117603	0.064584	0.557792	-0.19692
-0.49045	0.418832	-0.50491	-0.56152	0.637044	-0.41514	-0.21853	-0.56355	-0.0705
-0.02632	0.071993	-0.05623	0.21008	0.327461	-0.00346	-0.06525	-0.06462	-0.31153
0.078729	0.042037	-0.10644	-0.0516	0.288009	0.111919	-0.20607	-0.3647	0.103246
-0.16122	-0.18443	-0.25436	-0.00489	-0.20421	-0.29791	0.19998	0.012713	0.039951
-0.28426	-0.04117	-0.27881	-0.03677	-0.15283	-0.29748	0.25084	0.187696	0.158147
-0.01324	-0.28546	-0.0127	0.06266	-0.17685	-0.0708	-0.14327	-0.0543	0.080318
-0.20974	-0.14937	-0.2738	-0.23858	-0.18056	-0.35486	0.191817	-0.03674	0.1272
0.238389	0.236323	0.377962	0.432109	0.410687	0.415671	-0.33669	0.310933	-0.2614
-0.16124	-0.00714	-0.282	0.308152	0.212061	-0.17003	0.012427	-0.28613	-0.26788
-0.06547	-0.35852	-0.0417	0.186519	0.022026	0.041588	0.039578	-0.10424	-0.2201
0.562544	-0.32615	0.525593	0.602696	0.485075	0.444983	-0.21901	0.476855	-0.20595
0.307182	-0.01168	0.123381	0.135412	0.111875	0.057194	-0.06942	-0.041	-0.27725
0.328098	-0.14294	0.12239	0.14272	0.044532	0.214982	-0.27608	0.080032	-0.40725
0.326789	-0.15463	0.027352	0.136683	0.254944	0.112221	-0.26768	-0.06083	-0.39829
0.190553	0.064429	0.023183	0.232639	0.081788	0.023113	-0.22396	-0.18045	-0.4582
0.329683	-0.18789	0.200758	0.090227	0.120892	0.170663	-0.24846	0.068737	-0.44809
0.36064	-0.06904	0.170135	0.057888	0.137692	0.182841	-0.15625	-0.03699	-0.41706
0.302496	-0.23157	0.160699	0.089699	0.15037	0.122078	-0.15717	0.026294	-0.35105
0.325135	-0.33346	0.025176	-0.12678	-0.26092	0.013605	-0.34503	-0.07656	-0.41349
0.287115	0.254431	0.171077	0.052171	0.159769	0.152318	0.251437	0.079205	-0.08585
0.245195	0.119325	0.104136	0.261177	0.204374	0.269228	-0.14336	-0.18909	-0.38004
0.206994	-0.06487	0.210894	0.070929	0.098747	0.109958	-0.30812	-0.00674	-0.3744
0.308248	-0.26017	0.270962	0.151712	0.117741	0.106934	-0.24278	0.001444	-0.26155
0.468997	-0.22131	0.380377	0.420181	0.311538	0.309656	-0.10671	0.372594	-0.35169
0.302302	0.0358	0.124385	0.186752	-0.02771	-0.04139	-0.29772	-0.07464	-0.35339
0.325511	-0.09343	0.236062	0.176355	0.298706	0.124996	-0.20865	0.119184	-0.43685
0.62325	-0.42859	0.77803	0.603375	0.529671	0.503959	0.269346	0.722367	-0.12297
0.672595	-0.34066	0.644618	0.632368	0.546145	0.329359	0.07169	0.658689	-0.14699
0.697996	-0.38584	0.720267	0.611792	0.596427	0.337389	0.00849	0.716606	-0.13657
0.658298	-0.38148	0.642943	0.726566	0.617445	0.366397	0.016626	0.662777	-0.31976
0.439687	-0.10952	0.39514	0.447331	0.229487	0.267821	-0.19959	0.358937	-0.33807

TCGA-RB-A	TCGA-S4-A	TCGA-S4-A	TCGA-S4-A	TCGA-US-A	TCGA-US-A	TCGA-US-A	TCGA-US-A	TCGA-XD-A
-0.39014	-0.27594	-0.14796	-0.35513	0.164463	-0.1836	0.033333	-0.09473	-0.18668
0.002749	-0.38327	-0.34125	-0.12151	-0.04837	-0.2784	0.197258	0.24175	0.023609
-0.03683	0.362686	0.218789	0.349651	0.12626	-0.1346	0.229078	-0.37285	-0.15298
0.293617	0.059862	-0.31467	-0.35139	0.147985	-0.40812	0.383578	0.272079	0.446483
-0.02883	-0.34045	-0.25312	-0.05319	-0.19907	-0.05682	0.077501	0.207213	0.225772
0.031502	0.07666	0.120888	-0.07965	0.078379	0.202394	0.066485	0.209559	-0.14655
-0.16121	0.042734	0.070628	-0.20414	0.389327	0.321273	0.165642	-0.18842	-0.27188
-0.01295	0.227378	-0.1409	-0.03399	-0.09924	0.147187	0.220699	-0.04603	-0.0667
-0.01457	0.194106	-0.32128	0.242962	0.018993	0.192558	0.044804	-0.21057	-0.08084
0.21046	0.16175	-0.00024	0.213609	-0.10866	0.325673	0.151961	0.301032	0.132998
0.319563	0.479358	-0.02948	0.336296	0.398501	0.487263	0.404419	-0.14866	-0.25882
0.157138	0.301862	0.030988	0.303007	0.009714	0.338121	0.188038	-0.17936	-0.14149
-0.24807	0.115055	-0.40285	-0.19934	-0.40159	0.037159	0.382757	-0.10568	0.013958
0.130192	-0.10267	-0.06772	-0.07103	-0.019	0.176906	0.239542	-0.11841	-0.19848
-0.43094	-0.12222	-0.02447	-0.12398	-0.42065	-0.08502	0.127323	0.041373	-0.12524
0.173746	-0.54585	-0.30164	-0.19659	-0.1498	-0.48391	0.30719	0.396108	0.204091
0.17947	0.040924	0.167712	-0.00353	-0.21896	-0.28037	0.294123	-0.3906	-0.2499
-0.23158	0.047034	-0.21582	-0.33441	-0.18652	-0.22427	-0.14589	-0.04555	-0.0474
-0.03762	-0.09689	-0.49537	-0.39247	0.307938	0.163722	0.489597	0.130398	-0.24093
0.259587	0.331132	0.670411	-0.0495	0.480551	0.433015	-0.3672	-0.20858	-0.52984
0.002221	-0.04517	0.049903	-0.42037	0.264794	-0.07495	0.221767	-0.19989	0.040949
-0.20387	0.504643	0.293823	0.142446	-0.11069	-0.01302	-0.4276	-0.333	-0.58244
0.031336	-0.14915	-0.14003	-0.32124	-0.13798	-0.02134	0.009625	0.080215	-0.11311
0.053978	-0.30656	-0.30444	0.049163	-0.40861	-0.14048	0.290494	0.121404	-0.04782
-0.00899	-0.08625	0.136811	0.139102	0.051294	0.25674	-0.04949	0.114529	0.085655
-0.18127	-0.4002	-0.03592	-0.03863	-0.26104	-0.43302	-0.17208	0.3687	0.269242
-0.43816	0.437665	-0.09705	-0.01272	-0.57885	-0.22457	0.534295	-0.33922	0.144412
-0.10657	0.162826	-0.1759	0.096364	0.181728	0.022662	0.231833	-0.04015	0.41958
-0.17334	-0.0934	0.063913	0.135408	-0.14413	0.446711	0.000612	0.032087	-0.26134
0.181547	-0.10262	0.025337	0.385726	-0.21982	-0.29339	-0.22545	0.162821	-0.01388
0.002806	-0.50738	-0.38151	-0.14075	-0.60068	-0.51397	-0.57805	0.529568	0.272063
0.077936	-0.09676	-0.24632	-0.32447	-0.34968	-0.35364	-0.34666	0.655597	-0.44671
0.00318	0.132483	-0.10318	0.107505	-0.24537	-0.31457	0.020255	-0.07405	0.254822
-0.04559	0.051228	-0.04416	0.13311	-0.294	-0.35127	-0.02392	-0.18438	0.295539
0.01799	0.045637	-0.09671	0.224602	-0.24128	-0.30362	-0.07613	0.091058	0.302854
0.215801	-0.21834	0.251521	0.070139	-0.01157	0.22645	-0.01668	-0.02618	-0.22753
-0.29004	-0.3818	0.250992	-0.3119	-0.12652	-0.26113	-0.30929	-0.07527	-0.52979
0.169193	-0.20193	0.201517	-0.19676	-0.01949	0.463813	-0.32368	0.025995	-0.35728
0.079231	0.409505	0.2876	-0.00372	0.265753	0.506683	-0.17837	-0.3176	-0.07081
0.05359	0.001552	0.285963	0.058677	0.034302	0.273571	0.102575	-0.22606	-0.09755
0.11008	-0.17239	-0.049	0.161181	-0.12314	0.153246	0.199402	0.03314	0.11906
0.156777	0.409291	-0.00105	0.132133	0.376349	0.54617	0.215813	-0.19594	-0.02626
0.234444	-0.12966	-0.15875	0.167034	-0.22307	0.310507	-0.09746	0.150765	0.062977
0.087497	-0.15431	0.315722	0.019935	0.063474	0.576787	-0.31111	0.07378	-0.63321
0.165247	-0.07146	-0.62665	-0.08066	0.159243	0.402745	-0.58304	0.138302	-0.04859
0.36348	0.33308	-0.04803	0.228328	0.368369	0.537326	0.274105	-0.18135	-0.07394
0.211031	-0.29692	0.364055	-0.02192	0.119508	0.358651	-0.01755	0.186298	-0.38277
0.056954	-0.11744	0.137894	-0.06407	0.138911	0.056677	-0.27355	0.032942	-0.11699
0.109243	-0.11014	0.295785	-0.15068	0.300286	0.252264	-0.25232	0.018341	-0.38265

0.039996	0.003422	0.044272	-0.15132	0.282214	0.261312	-0.22803	-0.02597	-0.12478
-0.21236	-0.23683	-0.00421	0.026213	0.312304	0.297376	-0.26568	0.116298	-0.09178
0.011029	-0.168	0.240531	-0.20728	0.188154	-0.05656	-0.16462	-0.21026	-0.06702
-0.17435	-0.26144	0.144343	-0.36748	0.033848	0.29503	-0.24457	-0.08242	-0.21657
-0.21303	-0.02445	0.081269	-0.03444	-0.05648	-0.02235	-0.2183	-0.04716	-0.0115
0.131944	-0.15439	0.146317	-0.04795	0.108183	0.241481	-0.08346	-0.17544	-0.11433
0.296525	0.170019	0.256018	-0.00461	0.097769	0.416023	-0.22352	0.168224	0.070754
0.075707	-0.03697	0.204759	0.079507	0.0846	0.330425	-0.03935	0.167755	0.102259
-0.05069	-0.0545	0.220171	0.015358	-0.26853	0.241367	-0.19412	0.20342	-0.12535
0.171541	-0.25284	0.046834	0.122928	-0.07604	0.117233	-0.29812	0.379151	0.092566
-0.12005	-0.22278	-0.01538	-0.02362	0.145783	0.099877	-0.34223	-0.01883	0.32338
0.351506	0.077378	-0.05334	0.659143	-0.38953	0.137807	0.35437	0.300459	0.567882
0.166492	0.071714	0.064688	0.03133	0.028107	0.1537	-0.09932	-0.14513	0.014858
0.336129	-0.19336	0.089449	-0.14798	-0.05602	0.389048	-0.00746	0.309736	-0.19662
0.064532	0.102354	-0.00756	-0.00969	0.008352	0.184695	0.136135	-0.22387	0.001965
0.103567	-0.29466	-0.10524	-0.27304	0.204759	-0.03373	-0.177	-0.16637	-0.05794
0.424164	-0.61069	-0.03032	-0.07031	-0.42116	-0.53751	0.27973	0.326091	0.647957
0.354994	-0.16055	-0.36784	0.118414	0.192522	-0.41234	0.289233	0.084397	0.392985
-0.09979	0.193112	-0.01268	0.140839	-0.23292	-0.15312	-0.01294	-0.11514	0.060981
0.036682	-0.39197	0.205816	-0.27282	0.076403	0.167939	-0.24062	0.248829	-0.1889
-0.39826	-0.49372	0.192068	-0.42657	0.259459	0.155734	-0.33259	0.201789	-0.37598
-0.16946	-0.28222	0.084885	-0.15225	0.255693	0.128634	-0.30666	-0.0055	0.043672
-0.16264	-0.09567	0.316839	-0.09306	0.31772	0.427857	0.017686	0.099751	0.04865
-0.24282	-0.14602	0.36676	-0.06471	0.512346	-0.12644	-0.41458	-0.01307	-0.37425
0.002929	-0.13111	0.189659	-0.27452	0.243428	-0.0473	0.179619	0.100435	-0.10711
0.050893	0.390461	-0.08209	0.285281	0.314947	0.543897	0.279756	0.013323	-0.07163
0.244672	0.102384	-0.00981	0.068239	0.178114	0.527299	0.094792	0.012251	-0.39115
0.123299	-0.09155	0.22902	-0.062	0.318578	0.452816	-0.41924	0.113106	-0.43081
-0.10035	0.516719	-0.101	0.09887	0.258175	0.318567	0.198191	-0.30889	0.152011
0.070298	-0.03629	-0.27099	0.251096	-0.14412	0.289909	-0.19701	-0.03232	0.024486
0.330333	0.367756	-0.05283	0.160146	0.191687	0.600842	0.239141	0.02824	-0.12935
0.284258	0.370119	-0.15874	0.24223	0.158063	0.568962	0.271566	-0.0161	-0.00048
0.205981	0.368001	0.266673	0.122406	0.324613	0.539461	0.276048	-0.18414	-0.35286
-0.11973	0.0477	0.131595	0.08509	0.203587	0.408521	-0.05287	-0.23343	-0.51179
0.053249	-0.02866	-0.32938	0.14012	0.03012	-0.04746	-0.04105	0.050657	-0.14704
0.107614	-0.11961	0.477362	-0.21346	0.131453	0.49769	-0.66632	0.713945	0.199868
-0.37886	0.013972	0.369734	-0.33569	0.239956	-0.08186	-0.37985	0.026855	-0.27486
0.143868	-0.22812	0.236194	-0.47482	0.307326	0.420529	-0.44111	0.083265	-0.06659
-0.10379	-0.32179	0.364596	-0.40901	0.574729	0.194983	-0.44227	0.464438	-0.73872
-0.04197	0.001199	0.277868	0.089292	0.284188	-0.09775	-0.40771	0.181026	-0.13676
-0.23125	-0.6123	0.421655	-0.51363	0.347553	0.163615	-0.42015	0.298185	-0.57281
-0.02707	-0.3762	0.139017	-0.37165	0.179174	-0.43158	-0.32072	0.03739	-0.04569
0.002778	0.010761	0.029312	0.126603	0.075407	0.358365	-0.08027	0.023927	-0.02813
0.366046	0.185478	0.271549	0.133249	0.438962	0.48879	-0.04426	0.310847	-0.55135
-0.29523	-0.23602	0.086383	-0.39241	0.307471	-0.15388	-0.34567	0.204035	-0.41348
-0.25958	-0.20576	0.128039	-0.55863	0.573326	-0.08707	-0.26444	0.427114	-0.66002
-0.12851	0.097548	0.126797	-0.3201	0.58564	0.209457	0.06164	0.017271	-0.62772
-0.04117	-0.02132	-0.23456	-0.06866	-0.2431	-0.28291	0.00324	0.037964	0.22352
-0.30298	0.056684	-0.20672	-0.03637	-0.24359	-0.29068	-0.15858	-0.07535	-0.00829
-0.11844	-0.02099	-0.09789	0.099299	-0.05683	-0.18864	-0.19061	0.126279	0.277204

0.030015	-0.13786	-0.31501	-0.01493	-0.36603	-0.36161	-0.2612	0.407031	0.096219
0.009943	-0.22597	-0.27715	-0.00781	-0.31859	-0.25602	-0.27636	0.355499	0.047011
-0.15281	0.19006	-0.07132	0.236946	-0.15241	-0.10586	0.027665	0.01041	0.137101
0.085636	-0.15151	-0.12583	0.120628	-0.19305	-0.10684	-0.17557	0.239851	0.293888
-0.28463	-0.2829	0.349959	-0.34413	0.20418	-0.30162	-0.31507	-0.04032	-0.41467
-0.31309	-0.06394	0.122545	-0.09089	-0.08765	-0.20648	-0.12279	0.037435	-0.18351
0.159561	-0.0948	0.209259	-0.16558	-0.11102	-0.28813	-0.06916	0.03352	-0.20888
-0.28177	0.077694	-0.11336	-0.26033	-0.09642	-0.24472	0.034665	-0.25595	-0.18598
0.124656	-0.11961	-0.28544	-0.20527	0.193742	-0.30701	-0.08371	-0.04292	0.009141
-0.10401	-0.04551	-0.06421	0.144177	-0.31802	-0.15768	0.039514	-0.13295	-0.04859
0.1523	0.196504	-0.211	0.006902	0.07204	0.520955	0.012954	-0.17408	-0.41521
-0.33887	-0.11129	-0.10835	-0.29231	-0.31356	-0.32418	0.121486	-0.06493	0.100932
-0.11812	0.180765	-0.16159	0.003118	-0.23191	-0.20632	-0.04623	-0.00288	-0.10002
0.057766	0.051074	-0.18423	0.315275	-0.1561	-0.1387	0.050527	0.100864	0.343281
-0.09756	-0.05586	-0.14175	-0.06913	-0.20421	-0.10312	0.085662	-0.04309	0.184095
-0.13792	0.234964	-0.13202	0.252298	-0.391	-0.34849	0.273871	-0.27745	0.107758
0.301376	-0.15972	0.003222	0.255241	-0.29521	-0.04117	0.116929	-0.28004	-0.03837
0.080215	-0.0379	0.007398	0.266772	-0.28688	-0.12502	0.008439	-0.14237	0.339811
0.028849	-0.1617	-0.19827	0.085974	-0.35367	-0.47261	0.200466	-0.23118	0.395702
-0.10447	-0.02668	-0.29731	0.007478	-0.43663	-0.18583	-0.08023	-0.1217	0.174489
-0.05916	0.130475	-0.11105	0.043281	-0.32585	-0.09203	0.084979	0.048696	0.12524
0.078638	-0.20445	-0.26755	0.234722	-0.44281	-0.42425	0.031882	-0.11542	0.279787
0.304473	-0.35407	-0.24677	0.21388	-0.49866	-0.5136	0.182986	-0.03358	0.316995
0.016061	-0.35705	-0.37173	0.18899	-0.49824	-0.48474	-0.25117	0.437002	0.202593
-0.1293	0.034621	-0.32518	0.033261	-0.4019	-0.33152	0.217549	-0.2412	0.168254
-0.04741	0.081054	-0.11326	0.218015	-0.23937	-0.01186	0.225369	-0.28519	0.031912
0.050076	-0.0252	-0.21453	0.280151	-0.19053	-0.26395	-0.03505	-0.05483	0.299075
0.119864	-0.31436	-0.24311	0.238794	-0.37853	-0.07496	-0.09199	0.415795	0.370834
-0.16402	-0.1515	-0.28179	0.081915	-0.37222	-0.56168	-0.20689	0.384295	-0.26153
-0.1986	-0.09916	-0.29195	-0.02601	-0.33486	-0.3787	-0.20068	0.246913	-0.13085
0.029208	-0.1665	-0.23659	-0.26653	-0.34027	-0.51828	-0.37955	0.130543	-0.21016
-0.25672	0.074046	0.01213	-0.12291	-0.02114	-0.30244	0.239875	0.052319	-0.29362
0.064275	0.151295	-0.08911	-0.20655	-0.03535	-0.05405	-0.24517	0.399938	-0.10537
-0.20152	0.217139	-0.18769	0.053363	-0.2927	-0.36697	-0.28074	0.285943	0.137905
-0.07898	-0.27205	-0.24532	0.02785	-0.42719	-0.45943	-0.38022	0.538433	0.072903
-0.16282	0.077724	-0.19999	-0.19283	-0.3924	-0.42984	-0.08631	0.242232	-0.22867
-0.09688	0.136009	-0.23378	-0.11374	-0.38365	-0.39639	-0.18578	0.229225	-0.011
-0.12707	0.025026	-0.23608	-0.1111	-0.43969	-0.34965	-0.15859	0.347137	-0.07517
-0.16142	0.030057	-0.17981	0.098227	-0.22109	-0.22814	-0.09256	0.156259	-0.01561
0.163244	-0.01033	-0.14279	0.249704	-0.28723	-0.15799	-0.05472	0.183308	0.041067
0.010667	-0.09068	-0.28567	0.224853	-0.47049	-0.27125	-0.04054	0.120119	-0.01686
-0.25418	-0.10487	-0.39367	-0.09635	-0.54741	-0.4645	-0.57077	0.740576	0.146891
-0.07494	0.205425	-0.1559	-0.0427	0.061162	-0.0603	0.117103	-0.13721	0.149604
-0.1599	-0.03984	-0.25603	-0.0595	-0.22762	-0.36007	-0.028	0.004695	0.117976
-0.12759	0.058969	-0.05937	0.057996	-0.21993	-0.1426	0.023071	-0.11211	0.291844
0.134447	0.195648	0.050804	0.190305	0.263407	-0.1167	0.163351	0.091124	0.181741
0.062867	-0.09191	-0.15692	0.079807	-0.32388	-0.26732	-0.12711	0.019013	0.12419
-0.20973	0.007837	-0.00337	-0.03177	-0.17525	-0.15763	0.047219	-0.05796	0.082601
-0.10058	0.097388	-0.20524	0.195384	0.004128	-0.0496	0.084465	-0.11041	0.102819
-0.37367	-0.18537	0.026483	-0.23597	0.004294	-0.28956	-0.0716	-0.00818	-0.05891

0.086486	-0.05016	-0.17883	0.230975	-0.21636	-0.01392	0.07432	-0.00494	0.302873
-0.27875	-0.09172	-0.21934	-0.12336	-0.00051	-0.09723	0.106961	-0.11363	0.180306
-0.13472	-0.03193	-0.15023	-0.06758	-0.10964	-0.13417	-0.14124	-0.01037	0.278868
-0.34796	-0.22331	-0.25692	0.231726	-0.37747	-0.60294	-0.38537	0.564909	-0.18155
-0.05238	0.301476	-0.24669	0.586542	0.117461	0.544618	0.119424	0.056036	0.421615
-0.25768	0.019843	-0.36695	-0.1432	0.009387	-0.12261	0.159683	0.088541	0.186824
0.024047	-0.02086	-0.21258	0.278186	-0.22046	-0.13926	-0.20392	-0.15663	0.291996
0.04358	-0.1286	0.156583	-0.14196	0.082619	0.153485	-0.09196	-0.02718	-0.30751
0.087171	-0.33092	0.214891	-0.18411	0.187703	0.317293	-0.05281	0.168999	-0.32614
0.097861	0.048322	-0.03767	-0.16437	-0.01056	-0.03349	0.106507	-0.12225	0.018939
0.097024	-0.25565	0.267621	-0.11124	0.142129	0.266348	-0.08352	-0.02631	-0.34229
0.104963	-0.33828	-0.23973	0.05503	-0.3447	-0.34713	-0.35739	0.289305	0.335767
0.119118	-0.0246	-0.03595	0.251901	-0.04852	0.047808	0.259061	-0.01334	-0.15232
-0.22129	-0.09012	-0.1679	-0.00638	-0.32635	-0.17858	0.102006	-0.06804	-0.15874
-0.01186	-0.35067	-0.22861	0.08131	-0.53424	-0.55401	-0.45153	0.460789	-0.05831
-0.04449	-0.11897	-0.17196	0.037353	-0.32398	-0.25527	-0.03113	-0.01766	0.087252
-0.18165	-0.05971	-0.05054	-0.20772	-0.22396	-0.3118	-0.05196	0.063639	0.122816
-0.27658	0.029253	-0.33068	-0.02786	-0.3533	-0.4192	-0.05052	-0.16307	-0.02471
-0.29434	-0.01955	-0.16067	-0.18771	-0.29306	-0.33392	-0.12241	-0.10888	-0.08271
-0.23135	-0.08723	-0.18372	-0.12372	-0.31928	-0.28159	-0.13417	0.056123	0.162541
-0.23569	-0.08557	-0.2076	0.044817	-0.24305	-0.43761	-0.14325	-0.00668	0.117254
-0.16363	-0.03659	-0.21021	-0.1011	-0.3049	-0.36289	-0.04793	0.036137	0.164186
0.123481	0.16008	0.099658	0.120047	-0.24813	-0.31973	-0.31621	-0.03597	0.013775
0.215486	-0.31463	-0.25602	0.178609	-0.40961	-0.0918	0.100182	-0.04381	0.350171
-0.03811	-0.22638	-0.25301	-0.16903	-0.31264	-0.39861	-0.12747	-0.02721	0.234802
-0.24858	-0.13619	0.079108	0.052233	-0.24266	-0.37061	-0.04495	0.228758	-0.06453
-0.0794	-0.002	-0.04725	-0.03693	-0.32618	-0.31247	-0.15069	-0.05816	0.025326
-0.21619	-0.14996	-0.08639	-0.2083	-0.41204	-0.28634	-0.13145	0.158731	0.249141
-0.04839	-0.04273	-0.02324	0.086183	-0.26797	-0.39016	-0.03378	-0.05352	-0.03644
-0.33302	0.086693	-0.19149	-0.0246	-0.27438	-0.31423	-0.12243	0.175614	-0.0042
-0.13235	-0.35092	-0.41931	0.206417	-0.62265	-0.59151	-0.64911	0.698043	0.393112
-0.25727	-0.19359	-0.38847	0.114326	-0.52204	-0.66532	-0.40621	0.636706	-0.05376
-0.29503	-0.27246	-0.4238	0.227857	-0.52365	-0.69045	-0.38711	0.684923	-0.23721
-0.24113	-0.35173	-0.53623	-0.0652	-0.53839	-0.70878	-0.34512	0.650178	-0.22259
-0.03499	-0.26963	-0.12728	0.094429	-0.44373	-0.53923	-0.11267	0.356202	0.135966

TCGA-XD-A	TCGA-XD-A	TCGA-XD-A	TCGA-XN-A	TCGA-XN-A	TCGA-YB-A	TCGA-Z5-AAPL
-0.24599	-0.28008	-0.32987	0.095009	-0.23517	-0.26249	-0.23976
-0.39904	-0.40936	0.486866	-0.00638	-0.32098	-0.07157	-0.46586
-0.02117	-0.02613	-0.11754	-0.04033	0.069763	0.426305	-0.36357
-0.14725	-0.40601	0.277189	0.260709	0.016234	0.197986	-0.32042
-0.05224	0.074235	0.458652	0.313129	-0.19671	0.233418	-0.4136
0.083501	0.260365	0.161826	-0.01056	-0.16197	0.085047	-0.28398
-0.3594	-0.23788	-0.14511	-0.33933	-0.48363	-0.00932	-0.41986
-0.00973	0.116335	0.201255	-0.02502	-0.13982	-0.04564	-0.2279
-0.15488	-0.16897	0.093238	-0.05606	-0.04397	0.073166	-0.30122
-0.03105	0.204484	0.081529	-0.11499	0.166953	0.29892	-0.20982
-0.14876	-0.05414	0.26511	-0.34676	-0.17761	0.059457	-0.16743
-0.11647	0.139936	0.111396	-0.09603	-0.20594	0.133009	-0.11179
-0.10209	-0.0926	0.290044	-0.35282	-0.16269	0.261071	-0.5449
-0.08886	0.02019	0.168976	-0.15553	-0.15769	0.065146	-0.38797
-0.1407	-0.44927	-0.04818	-0.29147	0.290696	0.182802	-0.4562
0.125265	-0.35001	0.047777	0.014073	0.330084	-0.10793	-0.17086
0.137728	-0.38572	-0.00594	-0.26141	-0.28786	0.24681	0.016067
0.106916	-0.15957	-0.23146	-0.34152	0.013097	0.238404	0.090879
-0.25251	-0.33339	0.126198	0.061131	-0.47712	0.144582	-0.16618
-0.51942	-0.40898	-0.40869	-0.43975	-0.62176	-0.57498	0.018741
-0.12183	-0.10407	-0.01912	-0.48059	-0.19038	-0.2446	0.33695
-0.15379	-0.40054	-0.60186	-0.543	-0.0879	-0.3	0.57689
-0.11808	0.27005	0.113999	-0.30979	-0.17834	0.15403	0.008205
-0.07193	-0.21928	0.316283	-0.03211	-0.27047	0.312801	-0.40159
0.058108	0.328478	-0.17705	-0.08298	0.192891	0.176675	-0.08855
0.146833	-0.36352	-0.24523	-0.21672	0.253376	0.189842	-0.16232
0.36034	0.045296	0.274076	0.292196	-0.27601	0.300952	-0.47891
0.28887	0.352091	-0.05573	0.257346	0.210424	0.194979	0.301527
0.069874	0.269895	-0.31953	0.074566	-0.08981	0.078442	-0.30533
-0.10346	0.391808	0.498238	0.281128	0.313599	0.287598	-0.01892
0.287579	0.098462	0.168397	0.202491	0.400872	0.514229	0.422608
0.410142	0.388959	-0.28703	-0.03966	0.506187	-0.04849	0.753796
0.094075	0.270394	0.161576	-0.12124	0.258739	0.322435	-0.20627
0.136808	0.265272	0.340422	0.006333	0.294464	0.389003	-0.27039
0.22029	0.245644	0.151773	-0.06726	0.386529	0.354377	-0.202
-0.16141	0.352252	-0.09598	-0.19696	-0.23552	0.362249	-0.37397
-0.05288	0.27956	-0.51766	-0.19914	-0.38259	0.130101	-0.23682
-0.37087	0.328464	0.02786	0.056744	-0.15565	0.352164	-0.26215
-0.34779	0.04292	0.402664	-0.17072	-0.43127	0.181862	-0.32486
-0.16988	0.082285	-0.10244	-0.30466	-0.32618	0.423273	-0.33745
-0.09939	0.230053	0.408326	0.108736	-0.33838	0.35609	-0.23377
-0.23084	-0.01473	0.333535	-0.04743	-0.34236	0.115633	-0.46849
0.100352	-0.24566	-0.33361	-0.1534	-0.28745	0.161868	-0.39925
-0.20382	0.63651	0.063784	0.075026	-0.54123	0.451468	-0.46258
-0.07943	-0.21035	-0.26401	-0.12393	-0.03173	0.446786	-0.43543
-0.35499	0.05586	0.487801	-0.2401	-0.24954	0.230233	-0.38006
-0.20233	0.27057	-0.3	-0.09995	-0.11954	-0.20759	-0.17954
-0.16026	-0.10507	-0.37723	-0.14182	-0.14452	0.068142	0.0279
-0.35232	-0.12614	-0.37943	0.03136	-0.27695	-0.20336	0.094996

-0.14075	0.241973	-0.24747	-0.32702	-0.15893	-0.11452	-0.17523
-0.14934	0.250941	-0.30723	-0.0665	-0.19802	-0.08492	-0.36863
-0.3579	0.20635	-0.15907	-0.15747	-0.18632	-0.18245	0.189996
-0.02266	-0.21092	-0.55417	-0.19432	-0.49377	-0.12923	-0.38043
-0.11708	-0.13908	-0.05077	0.092263	-0.40227	-0.32709	-0.11382
-0.26811	0.22026	-0.20715	-0.32897	-0.13241	0.125709	-0.41966
-0.04445	-0.05341	-0.05366	-0.26079	-0.19057	-0.21081	-0.29012
-0.02661	0.136632	0.065077	-0.13674	-0.06482	0.060383	-0.22016
0.107118	0.187523	0.110422	0.025315	-0.01717	0.13379	-0.20062
0.27407	0.207542	-0.0055	-0.13907	-0.04632	0.419897	-0.14321
0.080246	-0.12113	-0.45258	-0.2327	-0.09174	-0.00639	-0.24555
0.097578	0.279421	-0.02077	0.553901	0.374526	0.207163	-0.42463
-0.22252	-0.02273	-0.27462	-0.21429	-0.33185	-0.21892	-0.12426
-0.17629	0.289616	-0.19804	0.0032	-0.0703	0.239035	-0.2955
-0.13215	0.12082	0.256772	-0.04035	-0.32055	0.304282	-0.30489
-0.12059	0.079456	-0.03178	-0.19226	-0.24668	0.19994	-0.33297
0.051765	-0.11386	0.610368	0.383784	0.42859	0.510028	-0.346
0.077559	-0.32811	0.396871	0.275661	0.04222	0.256762	-0.26439
0.146008	0.171324	0.041291	-0.07335	0.052918	0.01247	0.010597
0.221127	0.359861	-0.3051	-0.27263	-0.27175	0.195456	-0.28772
0.180173	0.301059	-0.58416	0.04204	-0.13368	-0.00859	0.092931
0.038244	0.055767	-0.52952	-0.26513	-0.34241	-0.11526	-0.37813
0.163304	0.215855	-0.37931	-0.24047	-0.42908	0.168408	-0.35217
-0.41997	-0.09901	-0.60147	-0.27516	-0.54009	-0.57808	0.152056
-0.1283	-0.06837	-0.32095	-0.03878	0.118358	0.136127	-0.23175
-0.18805	-0.11123	0.43197	-0.37603	-0.13608	0.345589	-0.25303
-0.42315	0.064647	0.39179	-0.27843	-0.38542	0.330845	-0.30853
0.136605	0.660054	-0.13614	-0.37772	-0.3582	0.153254	-0.32394
0.039552	0.056394	-0.01812	-0.40671	-0.22433	-0.2885	-0.02091
-0.09008	-0.26481	0.152291	-0.07472	-0.36037	0.077009	-0.24692
-0.11153	0.214456	0.358835	-0.29266	-0.18351	0.222313	-0.40221
-0.15394	0.120744	0.353616	-0.28974	-0.23509	0.333482	-0.37015
-0.33976	0.107568	0.411842	-0.20053	-0.31421	0.125669	-0.35313
-0.06058	0.243471	0.084558	0.335016	-0.33674	0.079157	-0.47096
0.105349	0.096969	-0.09658	-0.15695	0.068973	0.074227	-0.13567
0.55324	0.375817	-0.77792	-0.53692	0.588974	-0.46514	0.695082
-0.0719	-0.01072	-0.49368	-0.14445	-0.04404	-0.38236	0.513861
0.136256	-0.19896	-0.50756	-0.18137	0.020015	-0.44325	0.296431
-0.66787	0.133855	-0.39723	0.369155	-0.50108	-0.4626	0.59094
0.260961	0.287484	-0.37078	0.179064	0.168146	-0.22639	0.587591
-0.34638	0.682853	-0.33977	0.127962	0.279702	-0.36995	0.297053
0.130151	-0.54904	-0.64755	-0.24869	-0.31674	-0.48241	0.122385
0.28726	0.212962	0.007141	-0.11634	-0.18269	0.263875	-0.29655
-0.5437	0.019041	-0.23229	0.303264	-0.45486	-0.18118	0.388034
-0.35948	-0.10308	-0.36691	0.078324	-0.32821	-0.4533	0.443915
-0.62682	0.073481	-0.37082	0.094471	-0.52119	-0.44066	0.575027
-0.62139	-0.00403	-0.24816	-0.23201	-0.47765	-0.49875	0.582487
0.230809	0.191439	0.143472	-0.12089	0.308446	0.301553	0.054794
0.05905	-0.01516	0.222741	-0.02651	0.164957	0.05413	0.010252
0.302621	0.179229	-0.08341	-0.18964	0.315076	0.273856	0.069984

0.322434	0.258848	0.047775	-0.20988	0.353558	0.416825	0.300746
0.325286	0.255732	0.159735	-0.12608	0.313318	0.371368	0.336338
0.277769	0.162188	0.073703	-0.10011	0.279001	-0.03872	0.126124
0.343342	0.063805	-0.05041	-0.18668	0.289739	0.254239	0.060317
-0.34174	0.236258	-0.32924	0.183747	-0.19354	-0.31264	0.43258
-0.0407	0.129421	0.03472	0.115452	-0.10731	-0.29231	0.235685
-0.20118	0.222628	-0.20622	0.26254	-0.15483	-0.06889	0.090727
-0.07699	-0.21102	-0.06508	0.11177	-0.18713	-0.3689	0.25473
-0.08632	-0.01991	-0.13333	-0.10825	-0.24335	0.09519	-0.04418
0.084777	0.113878	0.234873	-0.12789	0.188584	0.327812	-0.08805
-0.38437	-0.13338	-0.0434	-0.31536	-0.38277	0.082797	-0.40016
0.168534	0.052738	0.168844	0.132861	0.147698	-0.10683	0.072663
0.030829	0.207098	0.198566	-0.04366	0.228038	0.132878	0.198229
0.283445	0.165588	0.099969	-0.01468	0.335165	0.321362	-0.00523
0.209538	0.008902	0.132418	-0.0077	0.15977	0.209418	-0.06799
0.082302	0.133891	0.202288	-0.15235	0.07368	0.204207	-0.10345
-0.11326	0.169147	0.111369	0.465738	-0.18431	0.180066	-0.43454
0.051085	-0.03633	0.094219	0.165047	0.199778	0.346429	-0.33345
0.369207	-0.05637	0.293436	0.086956	0.34747	0.328625	-0.21606
0.076192	0.058785	0.270552	0.128526	0.266561	0.343224	-0.22371
0.117187	0.229404	0.283703	0.006051	0.262591	0.03138	0.097775
0.124792	0.176282	0.369871	0.279617	0.351796	0.335567	-0.22985
0.09654	0.225902	0.396755	0.338683	0.404201	0.467244	-0.38181
0.375239	0.341291	0.136024	0.007918	0.555238	0.438205	0.118488
0.125611	0.044208	0.40608	0.035585	0.022031	0.220164	-0.293
-0.01749	0.137981	0.368637	0.048559	0.039733	0.381705	-0.35537
0.044712	0.177861	0.266928	0.244589	0.263019	0.298973	-0.20912
0.308071	0.140943	0.340898	0.202249	0.330289	0.335758	-0.30443
0.282675	0.081487	-0.10436	0.102373	0.40264	0.16857	0.517817
0.041995	0.092994	0.230557	-0.27293	0.143456	0.261688	0.402426
0.132886	0.059293	0.168866	-0.42652	0.293249	0.277033	0.364665
-0.00814	0.18568	0.221104	-0.11515	0.143851	0.15227	0.477363
0.228156	-0.02057	-0.16163	-0.27034	0.20287	-0.03632	0.533717
0.378769	0.109683	0.081677	-0.2167	0.317758	0.185089	0.403157
0.423396	0.375895	-0.02351	-0.25562	0.44382	0.426808	0.475659
0.20518	0.170283	0.201958	-0.15179	0.388884	0.101502	0.409531
0.272274	0.173579	0.205806	-0.15013	0.317068	0.212069	0.479375
0.373794	0.299111	0.155701	-0.24946	0.403235	0.232182	0.530871
0.239386	0.122475	0.060892	-0.19804	0.238509	0.070385	0.308579
-0.01578	0.135964	0.19953	0.092928	0.212622	0.301776	0.081899
0.267334	0.349009	0.285645	0.074509	0.349103	0.342147	0.079696
0.246402	0.123051	-0.08751	-0.06812	0.567225	0.393603	0.663365
0.246652	0.048881	0.107329	-0.00357	0.186077	0.238629	0.256669
0.172822	0.127156	0.246618	0.085673	0.26058	0.017376	0.201378
0.207674	-0.07293	0.085522	-0.05898	0.283436	0.224799	-0.05967
0.141362	0.200684	0.126538	0.053948	0.180437	0.173778	0.229501
0.064228	0.217556	0.298445	0.219803	0.307928	0.335169	-0.05945
0.154643	0.190074	0.129177	0.073898	0.072356	0.006361	0.048764
0.133877	-0.00016	0.13796	-0.04446	0.223141	0.11426	-0.07173
-0.03263	0.093178	0.082421	0.14021	-0.10099	-0.23119	0.159974

0.128124	0.043378	0.148817	0.073667	0.311866	0.340244	-0.17447
0.171825	0.216444	0.213136	-0.00643	-0.08399	0.264087	-0.2266
0.213055	0.101876	0.16543	0.09699	-0.04852	0.305116	-0.14444
0.439546	0.283744	-0.1264	-0.2483	0.494362	0.44252	0.579432
0.481848	0.107614	-0.26038	0.291031	-0.43327	0.35529	-0.51139
0.012842	0.158206	0.26593	0.058671	0.156731	0.136224	-0.19817
0.058537	0.116378	0.217145	-0.07067	0.209187	0.307249	-0.32491
-0.12946	0.230061	-0.25369	-0.15726	-0.10083	-0.0798	-0.14448
-0.28423	0.314329	-0.34048	-0.11644	-0.10192	-0.24727	0.03116
0.173353	0.287016	0.09726	-0.24914	0.134608	0.157119	0.095483
-0.25004	0.167002	-0.32736	-0.1612	-0.18954	-0.12713	-0.04879
0.35202	0.286835	-0.08073	-0.02046	0.483485	0.354415	0.107881
0.004763	0.073485	-0.12226	0.028562	-0.01832	0.276888	-0.30311
0.114618	0.076356	0.341342	-0.28743	0.148059	0.30048	-0.02652
0.266193	0.278676	0.377471	0.024902	0.356835	0.472757	0.472166
0.051746	0.124228	0.219004	0.135675	0.199848	0.270496	-0.09742
0.04419	0.035301	0.202657	0.138171	0.220773	0.091747	0.155294
0.056934	0.077782	0.285633	0.234493	0.151045	0.17173	0.044076
-0.07027	0.083761	0.308898	-0.022	0.193163	0.107651	-0.00668
0.127491	0.086627	0.452722	0.176735	0.242082	-0.04604	0.016259
0.166028	0.090274	0.14512	0.162811	0.282074	0.00098	0.02524
0.05353	-0.01304	0.312204	0.208036	0.205946	0.138186	0.074002
0.222367	0.225098	0.207576	0.025505	0.186002	0.044243	0.016225
-0.05751	0.018196	0.331891	0.347468	0.368507	0.438975	-0.36319
0.10848	0.094016	0.217674	0.130424	0.325999	0.172067	-0.13561
0.091144	0.372791	0.085646	-0.03763	0.22481	0.02921	0.028491
0.12822	0.21122	0.268338	-0.03808	0.321155	0.034089	0.031447
0.248998	0.195707	0.468642	-0.02597	0.273122	0.207662	0.294535
-0.02089	0.271785	0.311734	0.182981	0.060959	0.052884	-0.06209
0.0581	0.099398	0.313608	0.117881	0.153693	-0.0098	0.098158
0.572719	-0.11619	0.085897	0.222311	0.588194	0.623094	0.607904
0.325296	0.022148	0.016194	-0.00605	0.533536	0.412493	0.638063
0.351559	0.092773	-0.05044	-0.07364	0.554258	0.454023	0.698105
0.443768	0.070595	-0.11125	-0.12132	0.587304	0.233157	0.682433
0.279812	0.267829	0.204625	-0.10843	0.414867	0.474981	0.350781
