# Supplementary figures and images for: Development of a Prognostic Model Based on Pyroptosis-Related Genes in Pancreatic Adenocarcinoma
Source: Dis Markers. 2022 May 29;2022:9141117. doi: 10.1155/2022/9141117 (PMC9169203; doi:10.1155/2022/9141117)

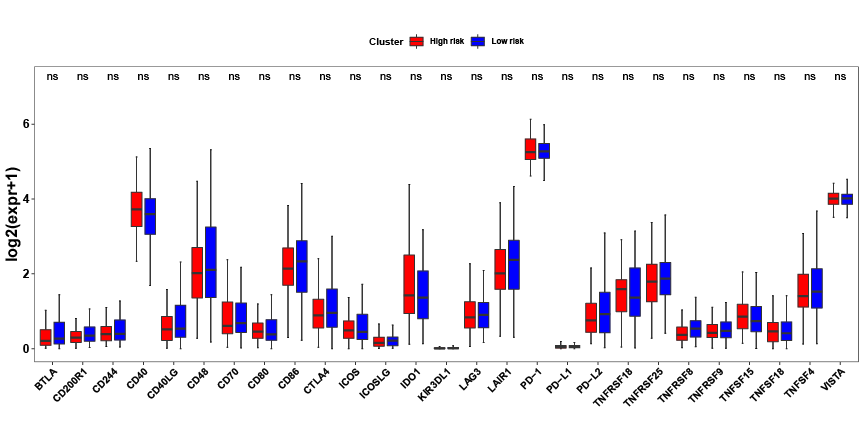

Supplement: Supplementary 4 — Figure S1: expression difference of other 27 immune checkpoints between high- and low-risk group. [file 9141117.f4.png]

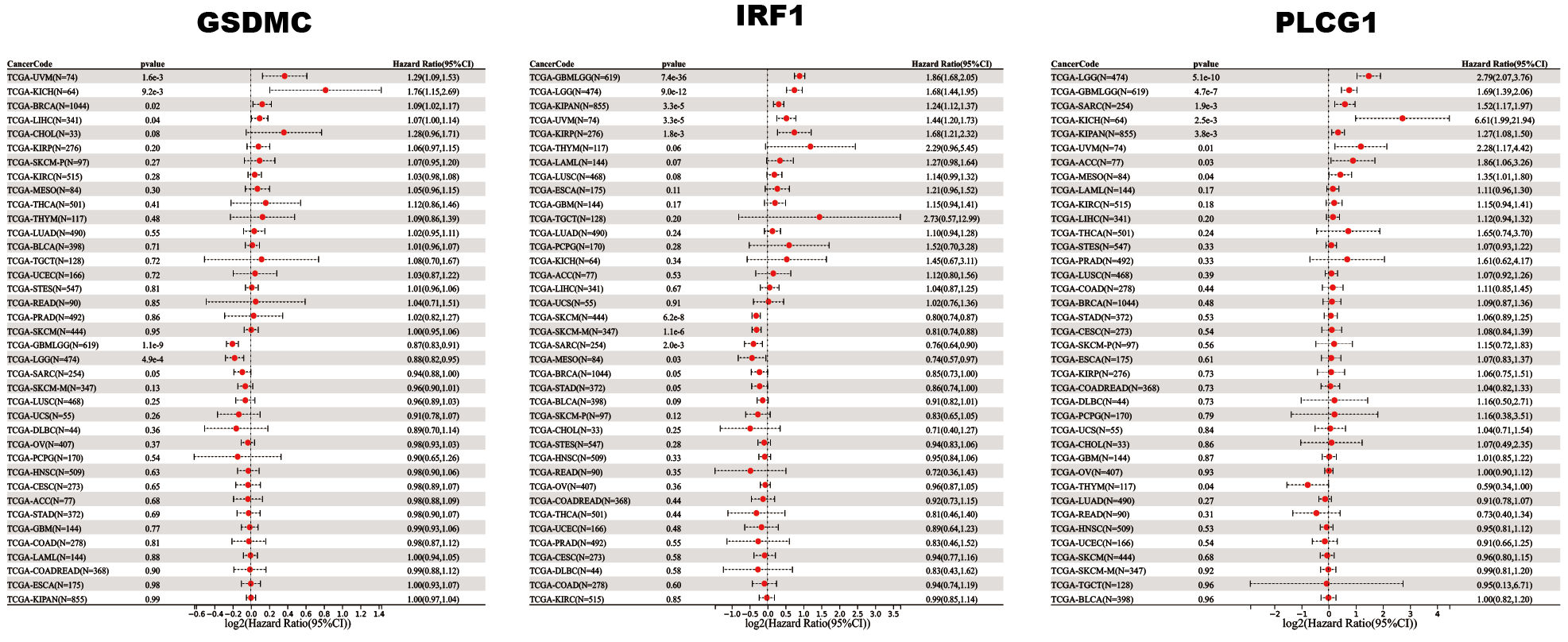

Supplement: Supplementary 5 — Figure S2: the predictive power of these three genes in other cancers. [file 9141117.f5.png]
